# Telerehabilitation for Motor Recovery in Adults with Neurological Disorders: A Systematic Review and Meta-Analysis Across Motor Domains, Delivery Models, and Follow-Up Effects

**DOI:** 10.3390/brainsci16090972

**Published:** 2026-09-14

**Authors:** Rocco Salvatore Calabrò, Arnold Fredrick D’Souza, Sanaz Pournajaf, Francesca Baglio, Carl Froilan D. Leochico, Alon Kalron

**Affiliations:** 1 IRCCS Centro Neurolesi Bonino Pulejo, 98124 Messina, Italy; roccos.calabro@irccsme.it; 2 Symbiosis College of Physiotherapy, Symbiosis International (Deemed University), Pune 412115, India; arnold.dsouza@scop.siu.edu.in; 3 Research Area in Neuromotor & Robotic Rehabilitation, Department of Neurological and Rehabilitation Sciences, IRCCS San Raffaele, 00163 Rome, Italy; 4 Department of Mental and Physical Health and Preventive Medicine, University of Campania ‘Luigi Vanvitelli’, 80138 Naples, Italy; 5 IRCCS Fondazione don Carlo Gnocchi ETS, 20148 Milano, Italy; 6 Division of Physical Medicine and Rehabilitation, Department of Medicine, University of Toronto, Toronto, ON M5S 3H2, Canada; 7 Department of Physical Medicine and Rehabilitation, St. Luke’s Medical Center, Global City, Taguig 1634, Philippines; 8 Department of Rehabilitation Medicine, Philippine General Hospital, Manila 1000, Philippines; 9 Department of Physical Therapy, School of Health Professions, Gray Faculty of Medical and Health Sciences, Tel Aviv University, Tel Aviv 6997801, Israel; alonkalr@tauex.tau.ac.il; 10 Multiple Sclerosis Center, Sheba Medical Center, Tel HaShomer 52621, Israel

**Keywords:** telerehabilitation, neurorehabilitation, motor recovery, neurological disorders, stroke, Parkinson’s disease, multiple sclerosis, meta-analysis

## Abstract

**Highlights:**

**What are the main findings?**

- Across 87 comparative studies, telerehabilitation effects varied according to neurological diagnosis, motor domain, delivery model, and comparator.
- Both primary meta-analyses included only three studies, had confidence intervals crossing the null, and provided very-low-certainty evidence; comparative efficacy therefore remains uncertain.

**What are the implications of the main findings?**

- Telerehabilitation should not be regarded as a universally effective substitute for conventional neurorehabilitation; interpretation should remain diagnosis-, domain-, and comparator-specific.
- Supervision, progression, adherence support, and safety monitoring are plausible implementation considerations, but their effects on efficacy have not been formally established.

**Abstract:**

**Background/Objectives:** Telerehabilitation may extend neurorehabilitation beyond conventional settings, but comparative effects across neurological diagnoses and motor domains remain uncertain. This systematic review and meta-analysis synthesized motor outcomes while distinguishing International Classification of Functioning, Disability and Health activity domains, delivery models, and comparator questions. **Methods:** Five databases were searched from inception to 8 June 2026 using two database-specific Boolean search strategies. Comparative studies of adults with neurological disorders receiving active remote, digital, virtual, wearable, or hybrid rehabilitation were eligible. Random-effects models used restricted maximum likelihood estimation; Hartung–Knapp confidence intervals were primary when at least three studies were available, and DerSimonian–Laird models were sensitivity analyses. **Results:** Eighty-seven studies included stroke, Parkinson’s disease, multiple sclerosis, spinal cord injury, acquired brain injury, or ataxia. For walking and mobility activities versus usual care, no additional therapy, or waitlist, the point estimate favoured telerehabilitation but was imprecise and compatible with no difference (standardized mean difference 1.06, 95% confidence interval −0.23 to 2.35; three studies; 121 participants; I^2^ = 57.6%). Global motor-functional and activities-of-daily-living outcomes showed similar uncertainty (0.83, −0.35 to 2.00; three studies; 304 participants; I^2^ = 73.6%). Both findings had very-low-certainty evidence. No pooled comparison included enough studies to assess publication bias. Two-study datasets for balance-related activity/postural control and upper-limb impairment were highly heterogeneous and interpreted descriptively. **Conclusions:** Comparative evidence for telerehabilitation across neurological diagnoses and motor domains remains uncertain. Programme characteristics are plausible implementation considerations, not established efficacy modifiers. Small evidence sets, wide intervals, clinical heterogeneity, risk of bias, and inconsistent adverse-event ascertainment preclude conclusions about clinical importance or established safety.

## 1. Introduction

Neurological disorders are now recognized as one of the largest sources of disability, death, and long-term health loss worldwide, with the Global Burden of Disease 2021 analysis showing that disorders affecting the nervous system account for a substantial and increasing proportion of global disability-adjusted life-years [1]. This epidemiological pressure intersects with a broader rehabilitation gap, since more than two billion people are estimated to live with conditions that may benefit from rehabilitation, and neurological disorders represent a major contributor to this unmet need [2]. Rehabilitation has therefore moved from a discretionary post-acute service to a core health strategy focused on functioning, participation, and continuity of care [3]. Health systems, however, remain poorly prepared to provide timely, intensive, and equitable rehabilitation, particularly when workforce shortages, fragmented services, transportation difficulties, and out-of-pocket costs limit access after hospital discharge [4,5].

Motor impairment is central to the clinical burden of many adult neurological disorders. Stroke, Parkinson’s disease (PD), multiple sclerosis (MS), spinal cord injury (SCI), traumatic brain injury (TBI), acquired brain injury (ABI), cerebellar ataxia, and related conditions frequently compromise upper-limb use, gait, balance, postural control, endurance, and independence in activities of daily living (ADL). These impairments influence participation and caregiver needs, even when survival improves and disease-specific pharmacological care is optimized. Contemporary rehabilitation research has increasingly emphasized that recovery should be judged not only by impairment-level change, but also by meaningful functional gains, participation outcomes, and individualized goals [6,7]. This perspective is especially relevant for adults with chronic or progressive neurological disorders, who often require repeated treatment cycles rather than isolated episodes of care. Telerehabilitation has emerged as a pragmatic response to this mismatch between need and service capacity. Broad overviews of the physical therapy and rehabilitation literature suggest that remotely delivered rehabilitation can be feasible and clinically useful in several populations, while also requiring careful attention to clinical suitability, patient preference, safety, digital literacy, and professional support [8,9]. Telehealth models in physical medicine and rehabilitation have been described as tools for extending assessment, education, monitoring, exercise supervision, and follow-up beyond conventional facilities [10]. In neurorehabilitation, remote delivery has gained additional momentum because digital platforms, videoconferencing, wearable sensors, virtual reality (VR), exergaming, and app-based programmes may support structured home practice, feedback, progression, and adherence [11,12]. Implementation research also shows that these technologies cannot be treated as neutral add-ons. They interact with clinical workflows, professional roles, infrastructure, reimbursement, accessibility, data governance, and patients’ confidence in using technology [13]. Existing syntheses support the plausibility of telerehabilitation in neurological populations, but they also reveal important limitations. A recent review of telerehabilitation for neurological motor impairment focused on quality of life (QoL), satisfaction, and technology acceptance in stroke, MS, and PD, leaving motor-domain-specific effects less clearly resolved [14]. Evidence from low- and middle-income countries suggests potential benefit, yet heterogeneity in intervention content, dose, delivery method, comparator, and follow-up prevented robust quantitative synthesis and limited conclusions on durability [15]. Stroke-focused reviews and umbrella reviews report encouraging results, but they remain diagnosis-specific and often combine diverse outcomes, technologies, and comparators [16,17,18]. Reviews of interactive telerehabilitation for balance and gait highlight remote monitoring and guidance as promising components, although eligible populations often include older adults without a neurological diagnosis, and outcome domains are not always separated with sufficient granularity [19]. Recent appraisal of telerehabilitation guidance in physical therapist practice also highlights its relevance for clinical integration, while emphasizing patient selection, safety, communication quality, and monitoring capacity [20]. A systematic review and meta-analysis organized around motor domains and delivery models can address these gaps more directly. Pooling all neurological diagnoses and all functional outcomes into a single estimate risks obscuring clinically meaningful differences between upper-limb recovery, gait and mobility, balance, global motor function, and disease-specific motor outcomes. Similarly, synchronous therapist-led videoconferencing, asynchronous app-based programmes, hybrid care, VR, wearable-assisted training, and caregiver-supported models may not have equivalent mechanisms or implementation requirements. Comparator type is equally important, because superiority over usual care has different implications from non-inferiority to in-person rehabilitation. The present systematic review and meta-analysis therefore aimed to synthesize evidence on telerehabilitation effects on motor outcomes in adults with neurological disorders, with specific attention to motor domain, delivery model, comparator type, intervention dose, and follow-up effects. This structure also supports clinically interpretable recommendations for trials, services, and future implementation in neurorehabilitation pathways while preserving diagnostic nuance.

## 2. Materials and Methods

### 2.1. Protocol Registration and Reporting Framework

The protocol for this systematic review and meta-analysis was prospectively registered in the International Prospective Register of Systematic Reviews (PROSPERO) under the registration number CRD420261416563 on 5 June 2026. Reporting followed the Preferred Reporting Items for Systematic Reviews and Meta-Analyses 2020 (PRISMA 2020) statement and the PRISMA literature search extension (PRISMA-S) [21,22]. The methodological plan was developed before study selection and followed the principles of the Cochrane Handbook for Systematic Reviews of Interventions [23]. The Supplementary Material is organized sequentially as follows: protocol registration and search records (Supplementary Tables S1–S3), PRISMA flow and exclusion reasons (Supplementary Tables S4–S6), eligibility and screening procedures (Supplementary Tables S7 and S8), data extraction and outcome hierarchy (Supplementary Tables S9 and S10), and quantitative synthesis safeguards (Supplementary Table S11).

The review question was structured to determine whether telerehabilitation improves motor outcomes in adults with neurological disorders and whether effects differ according to motor domain, delivery model, comparator type, intervention dose, and follow-up duration. This framework was selected to preserve clinical interpretability, because motor recovery after neurological disease is influenced by the target impairment, the intensity and supervision of practice, the technology used to deliver feedback, and the context in which remote rehabilitation is compared with usual care or conventional in-person rehabilitation. The protocol also defined decision rules for multiple outcomes, multiple intervention arms, missing variance data, and follow-up time points before data extraction, so that analytical flexibility would be minimized.

### 2.2. Population, Intervention, Comparator, Outcome (PICO) Framework and Eligibility Criteria

Eligibility criteria were operationalized according to a PICO framework. Adults aged 18 years or older with neurological disorders associated with motor impairment were eligible when they received active telerehabilitation delivered fully or partly through remote, digital, telehealth, or hybrid models. Eligible comparators included usual care, no additional intervention, waiting list, unsupervised home exercise, conventional in-person rehabilitation, or another active rehabilitation approach. Eligible outcomes were validated measures of upper-limb impairment or activity, walking and other mobility activities, balance-related activity and postural control, transfers, global motor function, disease-specific motor impairment, or activities of daily living (ADL). Within the International Classification of Functioning, Disability and Health (ICF), walking and transfers were classified as activities within mobility, balance tasks were treated as activity-level assessments of postural control, and ADL represented broader activity performance and independence. These categories were related but were not treated as interchangeable. Motor-functional outcomes were operationalized as validated measures of motor-dependent task performance or independence, including mobility, transfers, self-care, and other ADL, rather than isolated impairment measures alone. Eligibility therefore required an adult neurological population, an active remote motor-rehabilitation component, an eligible comparator, and at least one relevant motor or motor-functional outcome.

#### 2.2.1. Inclusion Criteria

Studies were included when they enrolled adults aged 18 years or older with a diagnosed neurological disorder associated with motor impairment. Neurological conditions of interest included stroke, PD, MS, SCI, TBI, ABI, cerebellar ataxia, and other adult neurological disorders when the telerehabilitation intervention targeted motor rehabilitation and at least one motor or motor-functional outcome was reported. Studies including mixed adult and pediatric samples were eligible only when adult data were separately extractable or when the adult subgroup could be clearly identified. Eligible interventions included active motor telerehabilitation delivered fully or partly through remote communication, videoconferencing, digital platforms, home-based connected technologies, VR, exergaming, wearable or sensor-assisted systems, or hybrid models combining in-person and remote care. The intervention had to involve structured motor training, therapeutic exercise, task practice, balance training, gait or mobility training, upper-limb rehabilitation, or another active motor-rehabilitation component. Eligible comparators included usual care, no additional intervention, waiting list, unsupervised home exercise, conventional in-person rehabilitation, or another active rehabilitation comparator. The primary eligible designs were randomized controlled trials (RCTs), quasi-RCTs, and controlled clinical trials published as full-text peer-reviewed articles in English. Non-randomized comparative observational studies—including prospective controlled studies, matched case–control studies, retrospective cohorts, and propensity-score-matched analyses—were eligible for narrative synthesis and sensitivity analyses when they provided relevant comparative data, but they were not planned for pooling with randomized trials in the primary quantitative analysis unless clinical and methodological justification was compelling.

#### 2.2.2. Exclusion Criteria

Studies were excluded when they focused exclusively on pediatric or adolescent populations, healthy older adults, musculoskeletal or orthopedic conditions without a neurological diagnosis, cardiopulmonary-only rehabilitation, frailty without a neurological diagnosis, or non-motor neurological symptoms without a motor-rehabilitation component. Interventions were excluded when they consisted only of teleconsultation without active motor training, passive telemonitoring without a therapeutic programme, cognitive-only rehabilitation, speech-only or swallowing-only telerehabilitation, psychological support alone, health education alone, or generic fitness applications without neurological rehabilitation intent. Case reports, case series, single-arm pre–post studies, protocols, editorials, narrative/scoping/systematic reviews, conference abstracts without sufficient outcome data, theses, non-peer-reviewed records, and articles not published in English were excluded. No restriction was placed on publication year. When a study included more than one neurological diagnosis, it was excluded if data from adults with neurological disorders were not separately extractable and the overall sample did not clearly meet the eligibility criteria for motor telerehabilitation.

### 2.3. Information Sources, Search Strategy and Study Selection

Electronic searches were run in PubMed, Web of Science, Cochrane Library, Embase, and Scopus on 8 June 2026, covering each database from inception to that date. Two conceptually harmonized Boolean strategies were implemented using the syntax accepted by each database interface, with two complete queries per database. The exact PubMed, Web of Science, Embase, Cochrane Library, and Scopus strings are reproduced verbatim in Supplementary Table S2. Web of Science used ALL = field syntax; the remaining strings were executed without explicit field tags and therefore used the respective interfaces’ default search behaviour. The searches did not specify controlled-vocabulary headings, truncation, or proximity operators. This database-level reporting makes the searches reproducible as executed, while the absence of controlled vocabulary and advanced operators may still have affected sensitivity and specificity. No citation tracking, reference-list searching, or other supplementary searching was undertaken, and the strategy was not formally peer reviewed by a librarian or information specialist. Eligibility was limited to English-language, peer-reviewed full-text reports. These restrictions were prespecified and are considered substantive limitations. No lower publication-date limit was imposed because telerehabilitation terminology and technologies have changed over time and early telephone-, web-, virtual-reality-, and home-technology trials could otherwise have been missed. Running all database searches on the same date minimized temporal discrepancies between sources. Two reviewers, RSC and SP, independently screened titles and abstracts and assessed potentially eligible full texts without automated prioritization or classification. The same reviewers recorded reasons for exclusion using predefined categories; disagreements were resolved by discussion and, when needed, consultation with AFD. Cohen’s kappa was 0.78 for title/abstract screening and 0.76 for full-text assessment [24,25]. Study selection was summarized in a PRISMA flow diagram with source-specific yields, duplicate removal, screening, full-text assessment, exclusion reasons, and final inclusion. When multiple reports described the same population, the most complete dataset was primary and companion reports supplemented methodological or outcome information. Database name, search date, exact query, retrieval count, and exported record set were archived for auditability. Full texts unavailable through institutional access were sought through library services or author contact when appropriate.

### 2.4. Data Extraction and Outcome Hierarchy

Data were extracted independently by RSC and SP using a standardized, piloted form. Extracted items included design, country, setting, recruitment source, sample size, diagnosis, age, sex, disease duration or time since neurological event, impairment severity, intervention content and delivery mode, technology, therapist and caregiver involvement, session frequency and duration, cumulative dose, adherence, comparator characteristics and intensity, outcomes, assessment time points, adverse events, funding, and conflicts of interest. The remote component was coded as synchronous, asynchronous, or hybrid and by dominant technology family. Unclear information was checked against supplementary files, trial registrations, and companion reports when available. Eligible outcomes were grouped a priori within an ICF-informed hierarchy: upper-limb impairment or activity; walking and mobility activities, including transfers; balance-related activity/postural control; global motor-functional or ADL activity; and disease-specific motor outcomes. Walking tests and mobility scales were not treated as interchangeable with balance tests, transfers, or broad ADL instruments. Examples included the Fugl-Meyer Assessment, FMA-UE, ARAT, BBT, 9-HPT, 10MWT, 6MWT, TUG, gait speed, BBS, Mini-BESTest, FIM motor subscale, BI, MBI, SIS, SCIM III, MDS-UPDRS III, MS walking outcomes, and SCI motor scores. Extraction retained the instrument, scale range, direction, analysis population, baseline and follow-up values, change scores, variance measure, and adjusted estimate when reported. Secondary outcomes included participation, falls, adherence, adverse events, satisfaction, usability, quality of life, caregiver burden, and resource use. Outcomes were extracted post-intervention and at prespecified short-term (≤3 months), medium-term (>3 to 6 months), and long-term (>6 months) follow-up. When several eligible outcomes occurred within one domain, the measure most directly aligned with the hierarchy and most frequently used across compatible studies was selected. One independent effect was retained per study, domain, comparison, and time point to prevent outcome or participant double-counting. Scale direction was harmonized before synthesis. Medians, interquartile ranges, ranges, or graphical data were converted only when recommended methods and transparent assumptions were available; otherwise, the study remained in narrative synthesis.

To improve clinical interpretability and to address the expected heterogeneity of the evidence base, each included study was additionally coded according to neurological population, primary and secondary motor-outcome domains, dominant delivery mode, and technology family. Delivery mode was categorized as synchronous, asynchronous, or hybrid, whereas technology family was categorized as standard digital/app/web/video/telephone delivery, VR/exergaming, wearable or sensor-assisted telerehabilitation, or robotic/FES/device-assisted telerehabilitation. Outcome-domain categories were not mutually exclusive because individual studies could report more than one eligible motor domain. This coding supported the evidence-distribution matrix presented after the robustness analyses.

### 2.5. Risk of Bias and Certainty of Evidence

Risk of bias was assessed independently by RSC and SP, with disagreements resolved by consensus. Randomized trials were evaluated with RoB 2 across the randomization process, deviations from intended interventions, missing outcome data, outcome measurement, and selection of the reported result [26]. Non-randomized controlled studies were assessed with ROBINS-I across confounding, participant selection, intervention classification, deviations, missing data, outcome measurement, and selective reporting [27]. These judgments directly constrained causal interpretation and informed sensitivity analyses. Certainty was assessed at the comparison-outcome level using GRADE [28]. Randomized evidence began at high certainty and was downgraded when limitations in risk of bias, inconsistency, indirectness, imprecision, or publication bias reduced confidence; non-randomized evidence began at low certainty and was not combined with randomized evidence for primary judgments. Final ratings of high, moderate, low, or very low incorporated the number and design of contributing studies, clinical coherence, interval width, and the ability to assess small-study effects. Publication bias was recorded as not assessable when fewer than 10 studies contributed, rather than interpreted as absent. Clinically distinct questions, including superiority versus minimal/usual care and non-inferiority versus dose-matched in-person rehabilitation, received separate judgments. Detailed study- and comparison-level inputs, model specifications, risk-of-bias linkages, and finalized evidence profiles are reported sequentially in Supplementary Tables S12–S17.

### 2.6. Data Synthesis and Meta-Analysis

Narrative synthesis described populations, interventions, delivery models, comparators, ICF-informed outcome domains, follow-up, adherence, and adverse events. Quantitative synthesis was considered only when population, intervention purpose, comparator question, outcome construct, and assessment time point were clinically exchangeable. Use of a standardized mean difference did not itself establish exchangeability. Walking/mobility activities, balance-related activity/postural control, transfers, upper-limb impairment or activity, and broader ADL outcomes were therefore evaluated as distinct constructs unless a prespecified clinical rationale supported grouping. Random-effects models were primary because clinical and methodological heterogeneity was expected [23]. Mean differences were used for a common scale, and standardized mean differences with Hedges’ g correction were used for different validated scales measuring the same construct [29,30]. Between-study variance was estimated by restricted maximum likelihood (REML), and heterogeneity was described with Cochran’s Q, I^2^, and τ^2^, with cautious interpretation for small k [31]. Hartung–Knapp adjustment was used for confidence intervals when at least three studies were available [23,32]. DerSimonian–Laird (DL) random-effects models were sensitivity analyses only; no fixed-effect model was used for the reported pooled results. Analyses with two studies were retained as exploratory model outputs, and high-heterogeneity two-study datasets were interpreted primarily as descriptive paired comparisons rather than generalizable pooled effects. For multi-arm trials, clinically similar intervention arms were combined when justified; otherwise, shared comparators were split to prevent participant double-counting. Cluster-randomized and crossover trials required adjusted estimates for clustering or period effects when available. Lower-is-better scales were reversed so that positive values consistently favoured telerehabilitation. Change scores were preferred when available; final values were used when change scores were unavailable and were not mixed without clinical justification. When several measures represented one domain, the prespecified hierarchy selected one effect and secondary measures remained narrative, preventing selective choice of the largest result. Missing variances were derived from standard errors, confidence intervals, *p* values, or other reported statistics when possible [23]; assumptions and derived values were retained in the Supplementary Material. Each pooled dataset was checked against the original report for sample size, group definition, assessment time, scale direction, and independence of effects. Prediction intervals and small-study tests were reserved for analyses with at least 10 studies [33,34]. Analyses used Review Manager and R with validated meta-analytic packages [35]. Primary pooling was restricted to randomized or quasi-randomized eligible comparisons; non-randomized evidence was not combined with randomized trials, and feasibility, delivery-model-only, or clinically incompatible studies informed narrative or sensitivity analyses.

### 2.7. Subgroup, Sensitivity and Additional Analyses

Subgroup analyses by diagnosis, motor domain, delivery model, comparator, intervention dose, supervision, technology, and follow-up were prespecified but were to be undertaken only when study numbers supported credible estimation. Comparator categories distinguished superiority versus usual care, no additional therapy, or waitlist; equivalence or non-inferiority versus conventional or dose-matched in-person rehabilitation; and comparative effectiveness between remote technologies or delivery models. Sensitivity analyses excluded high-risk or non-randomized evidence, studies requiring key variance imputation, and individual studies in leave-one-out analyses when at least three studies were available. Diagnostic restrictions were considered for stroke, Parkinson’s disease, multiple sclerosis, spinal cord injury, acquired/traumatic brain injury, ataxia, and mixed populations; time-point restrictions distinguished post-intervention from later follow-up. Alternative random-effects variance estimators were compared, with REML/Hartung–Knapp retained as primary and DL as sensitivity. Subgroup findings required sufficient studies, a coherent clinical rationale, and separation from confounding by comparator intensity or rehabilitation dose; otherwise, they were labelled hypothesis-generating. The sparse comparison-specific evidence precluded reliable meta-regression or formal moderator analyses for supervision, progression, safety monitoring, dose, or technology. These intervention features were therefore summarized qualitatively and were not interpreted as demonstrated effect modifiers. Unplanned analyses were identified as exploratory, and analyses with fewer than three studies were interpreted descriptively.

## 3. Results

### 3.1. From Search Yield to Included Evidence

**Figure 1:**
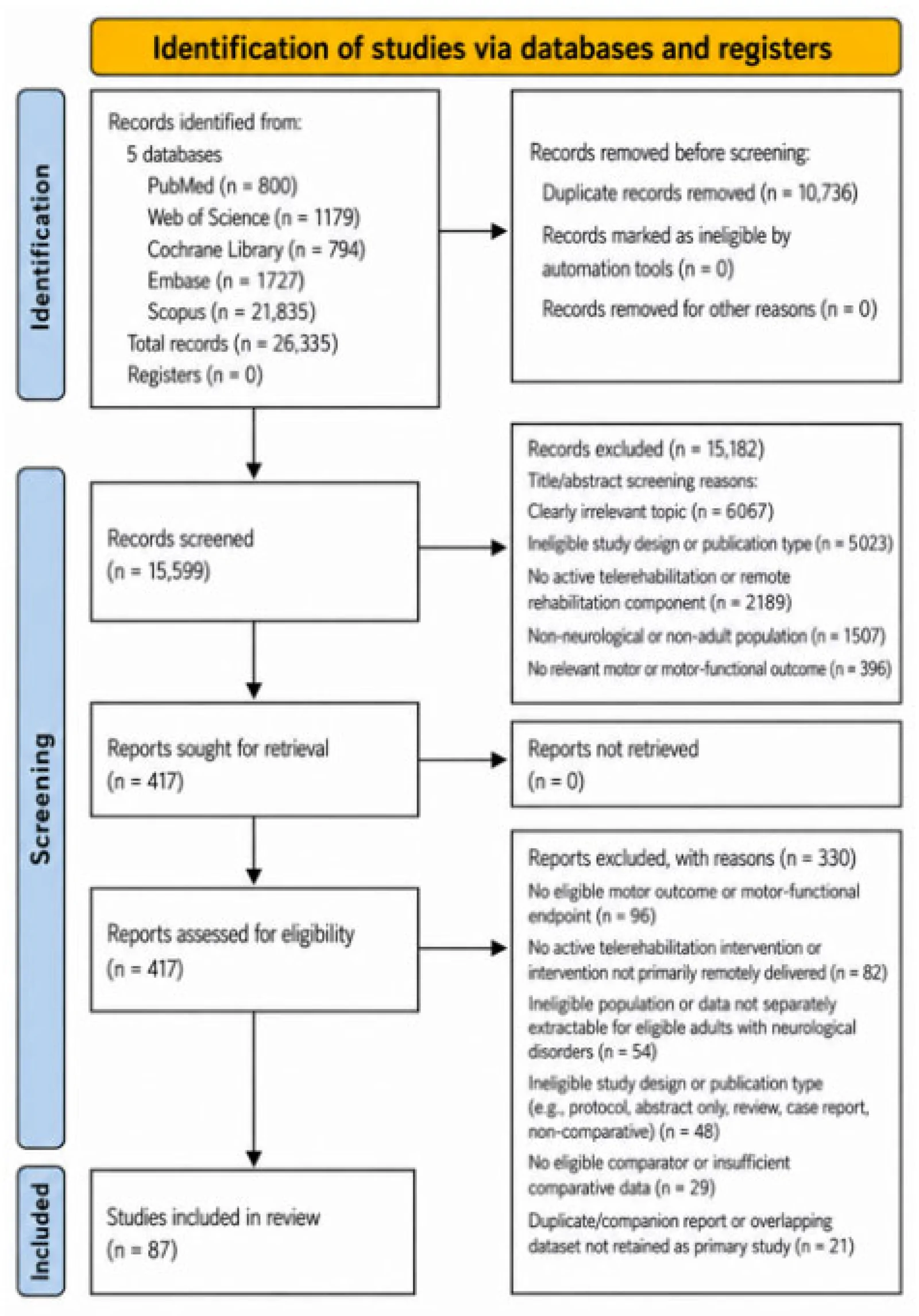
The PRISMA 2020 flow diagram for study identification, screening, eligibility assessment, and inclusion. The diagram summarizes the records identified from five databases, duplicate removal, title/abstract screening, full-text assessment, exclusion reasons, and the final set of 87 included studies.

The search identified 26,335 records across five databases, with no additional register records. After removal of 10,736 duplicates, 15,599 records underwent title and abstract screening. A total of 15,182 records were excluded at this stage, mainly because they addressed irrelevant topics, ineligible designs, non-neurological or non-adult populations, interventions without an active telerehabilitation component, or outcomes outside the motor and motor-functional scope of the review. Four hundred and seventeen reports were retrieved and assessed in full text; 330 were excluded with reasons, leaving 87 studies for qualitative synthesis (Figure 1). Included studies covered a broad chronological and methodological spectrum, from early home-based and VR telerehabilitation studies to recent wearable, app-supported, videoconferencing, and hybrid-care trials [36,37,38,39,40,41,42,43,44,45,46,47,48,49,50,51,52,53,54,55,56,57,58,59,60,61,62,63,64,65,66,67,68,69,70,71,72,73,74,75,76,77,78,79,80,81,82,83,84,85,86,87,88,89,90,91,92,93,94,95,96,97,98,99,100,101,102,103,104,105,106,107,108,109,110,111,112,113,114,115,116,117,118,119,120,121,122]. Across this evidence base, the dominant clinical focus was post-stroke rehabilitation, but PD, MS, SCI, severe ABI, mixed neurological populations, and hereditary or cerebellar ataxia were also represented. Study designs included RCTs, randomized pilot or feasibility trials, crossover trials, non-inferiority trials, prospective controlled studies, matched case–control designs, retrospective cohort studies, and propensity-matched observational analyses. Consequently, evidence synthesis required a staged approach in which all studies informed the qualitative map, but only a subset met the prespecified requirements for quantitative pooling. Study-level characteristics, populations, comparators, motor domains, and analysis classification are summarized in Table 1.

### 3.2. A Neurological Evidence Map Built Around Motor Domains

The 87 studies were clinically diverse and were mapped to five related but non-interchangeable domains: upper-limb impairment or activity, walking and mobility activities, balance-related activity/postural control, global motor-functional or ADL activity, and disease-specific motor outcomes [36,37,38,39,40,41,42,43,44,45,46,47,48,49,50,51,52,53,54,55,56,57,58,59,60,61,62,63,64,65,66,67,68,69,70,71,72,73,74,75,76,77,78,79,80,81,82,83,84,85,86,87,88,89,90,91,92,93,94,95,96,97,98,99,100,101,102,103,104,105,106,107,108,109,110,111,112,113,114,115,116,117,118,119,120,121,122]. In ICF terms, walking and transfers were treated as mobility activities; balance tasks represented activity-level performance of postural control; and ADL instruments captured broader activity performance or independence. Stroke contributed most evidence across domains, whereas Parkinson’s disease studies mainly addressed walking, mobility, balance, disease-specific motor symptoms, and exercise capacity; multiple sclerosis studies emphasized walking, balance, and physical performance; and spinal cord injury studies emphasized mobility, transfers, independence, and upper-limb capacity. Measures, diagnoses, time points, and comparators varied substantially. Table 1 therefore separates primary pooling candidates from non-inferiority, active-delivery, feasibility, non-randomized, and narrative evidence rather than assuming that statistical standardization made these constructs clinically exchangeable.

### 3.3. Remote Rehabilitation as a Treatment Ecosystem

Telerehabilitation comprised heterogeneous delivery models: synchronous videoconferencing, asynchronous applications, VR/exergaming, wearable/sensor-assisted systems, device-assisted training, and hybrid or caregiver-supported programmes. Table 2 details technology, content, dose, supervision, caregiver involvement, and motor domain. Programmes ranged from 2–4 weeks to 3–6 months and from twice-weekly sessions to daily practice. Feedback ranged from live therapist correction to automated or delayed review; caregiver involvement ranged from equipment setup to physical assistance, and comparator contact from waitlist to dose-matched specialist rehabilitation. Monitoring, progression, technical support, and caregiver presence were reported implementation features, but their independent effects were not formally tested.

### 3.4. Clinical Questions and Motor-Domain Evidence

The narrative evidence addressed three different clinical questions. In superiority comparisons versus usual care, waitlist, or minimal additional treatment, between-group effects varied across motor domains; favourable within-group change did not establish added benefit. Apparent advantages were often accompanied by small samples, greater intervention contact, multiple outcomes, or short follow-up. Comparisons with conventional or dose-matched active rehabilitation addressed substitution, but non-significant differences alone did not establish equivalence. The Cramer et al. trial supported the non-inferiority of home telerehabilitation to dose-matched in-clinic upper-limb rehabilitation under its prespecified framework [56]. Comparisons between remote models, including synchronous versus asynchronous formats, multi-user versus single-user VR, and alternative feedback configurations, addressed comparative delivery and engagement rather than telerehabilitation-versus-control efficacy [37,41,59,65,89]. Upper-limb evidence was the largest cluster, while walking/mobility and balance evidence spanned different diagnoses and measures. Global motor-functional and ADL outcomes often combined motor and non-motor programme components, while disease-specific scales addressed narrower questions. Durability remained uncertain because follow-up windows varied and many trials ended at post-treatment assessment. Table 3 retains study-level detail; interpretation rests on comparator question, clinical construct, risk of bias, and between-group evidence.

### 3.5. Risk of Bias and Certainty: Where the Evidence Was Most Fragile

**Figure 2:**
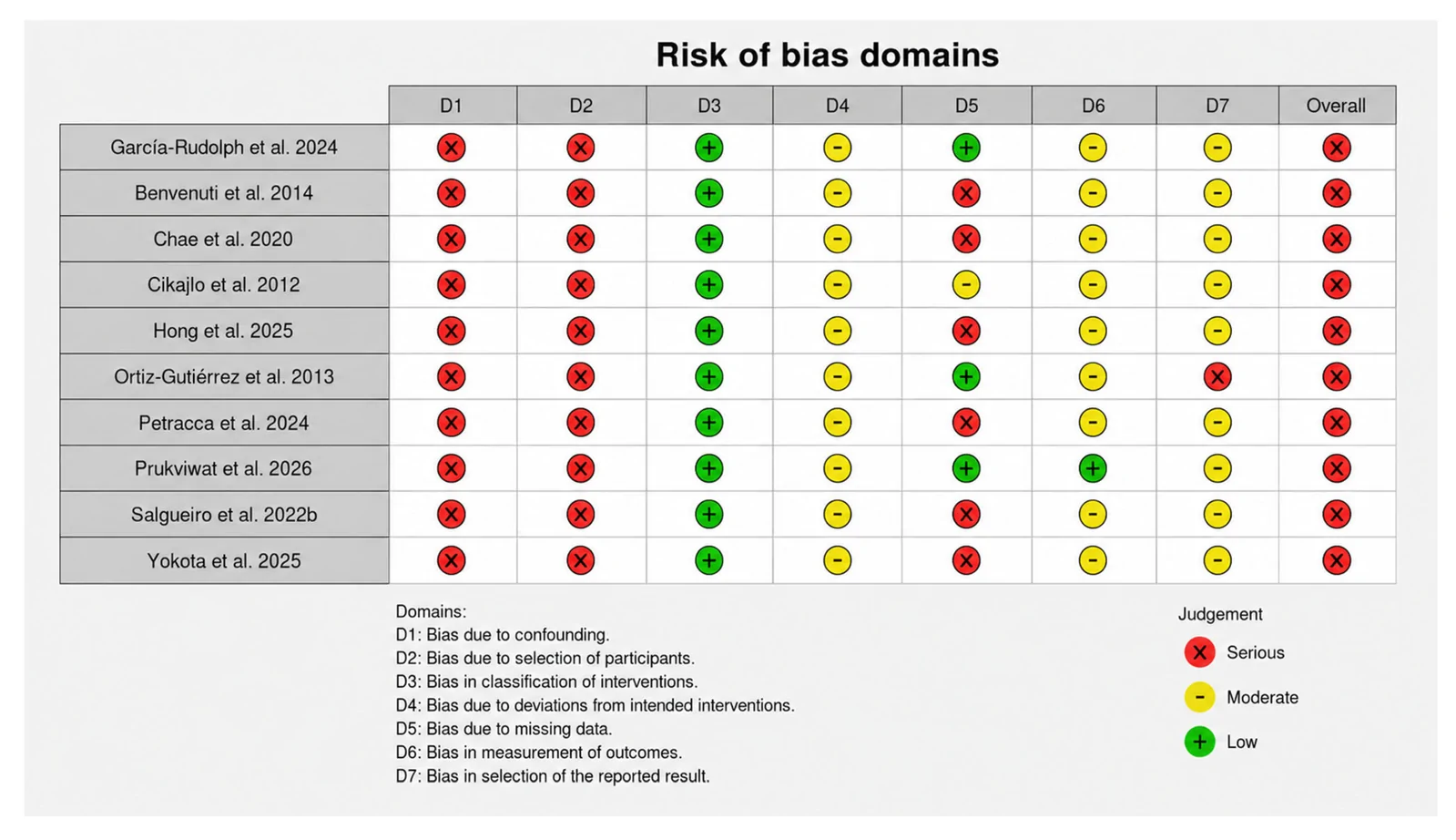
ROBINS-I risk-of-bias domain judgments for non-randomized controlled studies [36,43,46,53,76,90,95,98,101,121]. Domains: D1, bias due to confounding; D2, bias due to selection of participants; D3, bias in classification of interventions; D4, bias due to deviations from intended interventions; D5, bias due to missing data; D6, bias in measurement of outcomes; D7, bias in selection of the reported result. Green indicates low risk, yellow moderate risk, and red serious risk.

**Figure 3:**
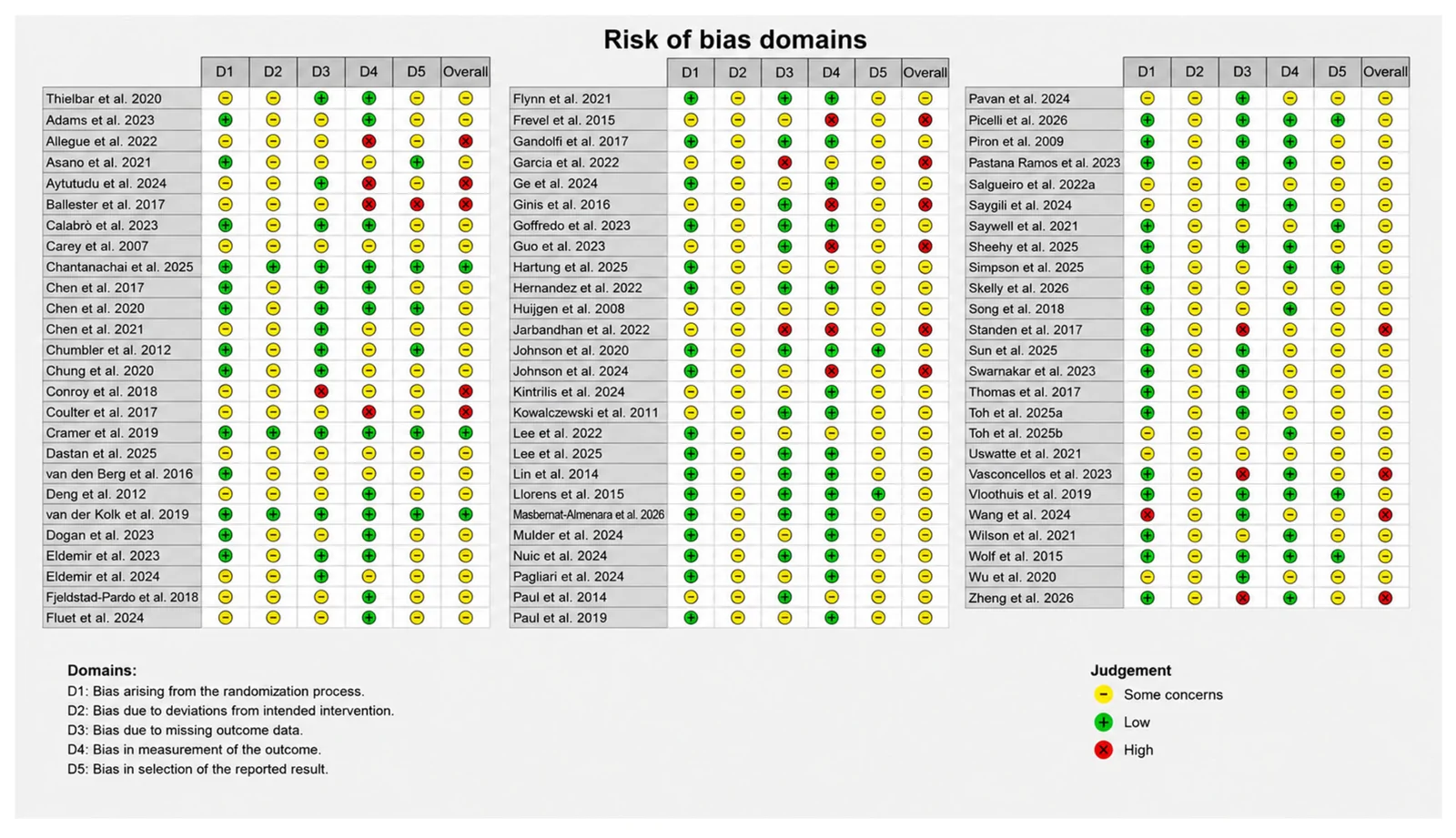
RoB 2 risk-of-bias domain judgments for randomized trials [37,38,39,40,41,42,44,45,47,48,49,50,51,52,54,55,56,57,58,59,60,61,62,63,64,65,66,67,68,69,70,71,72,73,74,75,77,78,79,80,81,82,83,84,85,86,87,88,89,91,92,93,94,96,97,99,100,102,103,104,105,106,107,108,109,110,111,112,113,114,115,116,117,118,119,120,122]. Domains: D1, bias arising from the randomization process; D2, bias due to deviations from the intended intervention; D3, bias due to missing outcome data; D4, bias in measurement of the outcome; D5, bias in selection of the reported result. Green indicates low risk, yellow some concerns, and red high risk.

Risk-of-bias assessments materially constrained causal interpretation. The ROBINS-I and RoB 2 summaries are shown in Figure 2 and Figure 3, respectively. Randomized evidence frequently involved small samples, pilot or feasibility objectives, unclear or incompletely reported randomization and allocation concealment, and limited precision [37,39,45,50,53,54,55,57,59,64,67,69,78,80,83,84,85,92,93,99,104,106,108,111,113,122]. In behavioural rehabilitation, the inability to blind participants and therapists was expected, but it became consequential when experimental groups received more contact, feedback, monitoring, motivation, or technical support and when outcomes were self-reported or assessor blinding was absent. These limitations were not treated as background features: they reduced confidence in treatment-effect estimates and contributed directly to GRADE downgrading.

Missing outcome data and attrition represented another important limitation. This issue was particularly relevant in feasibility trials, long home-based programmes, crossover designs, studies affected by pandemic-related constraints, and trials requiring sustained technology use or caregiver support [39,40,46,54,55,58,64,65,66,69,78,80,84,88,93,104,108,113,115,122]. Attrition was clinically informative, because dropout often reflected the real-world burden of telerehabilitation, including usability problems, connectivity barriers, fatigue, medical instability, reduced motivation, or difficulty maintaining adherence at home. However, incomplete follow-up also reduced confidence in the estimated motor effects, especially when analyses relied on completers or when imputation methods were insufficiently described.

Outcome-measurement bias was also common. Although several trials used blinded assessors or objective instruments, others relied on self-reported function, telephone-based follow-up, therapist-collected outcomes, wearable-derived measures with incomplete wear-time information, or performance outcomes assessed in contexts where blinding was difficult [40,51,52,54,55,73,80,88,103,105,117]. This was especially relevant for global motor-functional, ADL, adherence, physical-activity, and patient-reported outcomes, where treatment expectations and differential contact could influence responses. Selection of the reported result and outcome multiplicity were additional concerns in studies reporting numerous motor, functional, cognitive, psychological, adherence, and QoL outcomes without clearly prespecified primary endpoints or hierarchical analysis plans [39,44,50,63,65,69,73,74,80,91,106,117,122].

The non-randomized evidence assessed with ROBINS-I was generally judged at serious risk of bias (Figure 2). The main concerns were residual confounding, historical or geographic controls, patient-preference allocation, retrospective grouping, pandemic-era selection effects, and incomplete adjustment for baseline clinical differences [36,43,46,76,90,95,98,101,121]. These studies were valuable for feasibility, implementation, access, and contextual interpretation, but they were not combined with randomized evidence in primary meta-analyses.

Only a minority of studies had more robust features, including clearer randomization, assessor blinding, matched intervention dose, complete follow-up, objective outcomes, or prespecified non-inferiority frameworks [47,56,60,96]. Study-level GRADE inputs, comparison-level considerations, and risk-of-bias linkage are reported in Supplementary Tables S12–S14; final comparison-outcome ratings are reported in Supplementary Table S17. Certainty was very low for both primary pooled outcomes and for the clinically heterogeneous exploratory two-study estimates, reflecting risk of bias, small k, imprecision, inconsistency, indirectness across diagnoses, comparators, scales, and time points, and inability to assess publication bias. The downgrading rationale was outcome-specific rather than attributed to heterogeneity alone.

### 3.6. Quantitative Synthesis: Uncertain Comparative Effects

**Figure 4:**
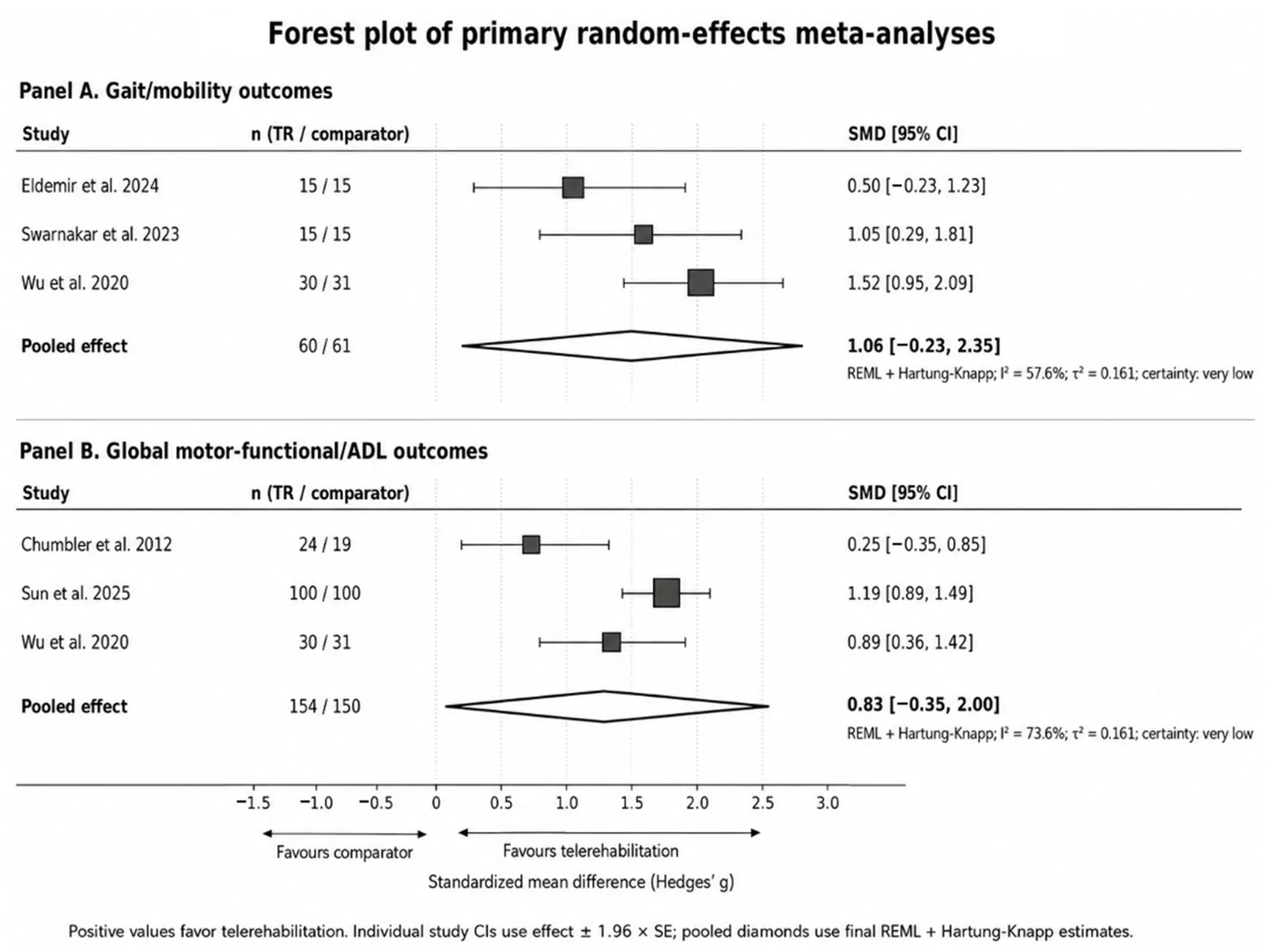
A forest plot of the two primary random-effects analyses comparing telerehabilitation with usual care, no additional therapy, or waitlist. Panel (**A**) reports walking/mobility activity outcomes, and Panel (**B**) reports global motor-functional or activities-of-daily living activity outcomes. The effect sizes are Hedges’ g standardized mean differences, with positive values favouring telerehabilitation. The restricted maximum likelihood estimated between-study variance, and Hartung–Knapp confidence intervals were used. Both intervals cross the null and are compatible with no between-group difference. The high-heterogeneity two-study datasets are reported descriptively in the text and transparently in Table 4 and the Supplementary Material [51,63,109,110,120].

Five datasets met the rules for a primary model or an exploratory quantitative summary (Table 4). The two primary analyses, shown in Figure 4, used REML random-effects models with Hartung–Knapp confidence intervals. For walking and mobility activities versus usual care, no additional therapy, or waitlist, the point estimate favoured telerehabilitation, but the interval was compatible with no difference (SMD 1.06, 95% CI −0.23 to 2.35; k = 3; n = 121; I^2^ = 57.6%; very low certainty) [63,110,120]. Global motor-functional or ADL activity outcomes showed similar uncertainty (SMD 0.83, 95% CI −0.35 to 2.00; k = 3; n = 304; I^2^ = 73.6%; very low certainty) [51,109,120]. An exploratory two-study active-comparator summary for upper/global motor function yielded SMD 0.67 (95% CI 0.23 to 1.10; n = 87), but outcome constructs and intervention contrasts differed and certainty was very low [42,49]. Two-study datasets for balance-related activity/postural control (I^2^ = 92.5%) [91,120] and upper-limb impairment (I^2^ = 83.5%) [38,112] were interpreted descriptively. Study estimates and exploratory outputs remain in Table 4 and Supplementary Tables S15–S17.

### 3.7. Robustness and Inferential Fragility

Sensitivity analyses showed inferential fragility despite stable point-estimate direction (Table 5). DL models retained the primary point estimates but produced narrower confidence intervals excluding the null: 0.45 to 1.66 for walking/mobility and 0.30 to 1.35 for global motor-functional/ADL outcomes. With only three studies, variance estimation and the reference distribution changed whether intervals crossed the null. Leave-one-out estimates remained positive but reduced each primary analysis to two studies and were treated as influence diagnostics, not confirmatory results. Excluding the SCI study did not resolve heterogeneity in walking/mobility; excluding the six-month Chumbler outcome reduced heterogeneity but also left only two studies. Restrictions on the original two-study datasets generally left one study and could not be pooled. Supplementary Tables S15–S17 provide inputs, model outputs, and finalized GRADE ratings. Comparative efficacy therefore remains uncertain.

Table 6 maps the evidence by neurological population, motor-outcome domain, delivery mode, and technology family. Outcome-domain counts are non-mutually exclusive; delivery mode and technology are coded at the dominant study level. These counts describe evidence distribution, not comparative effectiveness or clinical exchangeability.

## 4. Discussion

### 4.1. Comparative Effects Across Clinical Questions and Delivery Models

Across 87 comparative studies, telerehabilitation’s comparative efficacy remains uncertain across neurological diagnoses and motor domains. The broad literature did not translate into large clinically coherent quantitative comparisons because diagnoses, technologies, motor constructs, comparator intensity, and assessment times differed. This does not establish ineffectiveness; the comparison-specific evidence cannot distinguish benefit from no difference with adequate confidence. Stroke-focused reviews also require interpretation by outcome, content, comparator, and study quality [123,124]. Home-based technologies represent multiple intervention models rather than a single homogeneous treatment [125].

Stroke predominance and sparse evidence for SCI, ABI, mixed populations, and ataxia limit transdiagnostic inference (Table 6). Differences in recovery trajectories, functional goals, and intervention mechanisms further constrain generalization.

ICF-informed distinctions prevent conflation of impairment, mobility activities, postural-control performance, and broader ADL. Related upper-limb syntheses also require interpretation by system, dose, severity, comparator, and study quality [126,127,128]. Wearables may extend measurement beyond clinic testing, but current systems remain insufficiently standardized for strong cross-study inference [129,130]. Large standardized point estimates cannot be assumed clinically important without outcome-specific meaningful thresholds.

Comparator choice separates superiority over usual or minimal care, substitution for dose-matched active rehabilitation, and comparisons between remote formats. Non-inferiority applies only to the tested population, endpoint, margin, and conditions; it cannot establish generalized superiority. Cost-effectiveness is a separate question when comparable outcomes are established [131]. The related literature in PD [132,133], MS [134,135,136], and SCI [137] provides diagnosis-specific context, but cannot resolve the uncertainty of this review’s heterogeneous comparisons.

### 4.2. Uncertainty, Risk of Bias, and Certainty of Evidence

The primary REML/Hartung–Knapp intervals crossed the null and GRADE certainty was very low. The narrower DL sensitivity intervals demonstrate dependence on analytical assumptions with only three studies, rather than robust statistical confirmation. High-heterogeneity two-study datasets support descriptive interpretation. These findings preclude confident conclusions about comparative benefit or clinical importance.

Risk of bias was an equally important constraint. Small pilot and feasibility trials, incomplete allocation concealment, unavoidable lack of participant and therapist blinding, differential contact or dose, missing data, outcome multiplicity, and selective reporting limited randomized evidence. Self-reported or therapist-collected outcomes were particularly vulnerable when treatment expectations and contact differed. Non-randomized studies were generally at serious ROBINS-I risk because of confounding, selection, historical controls, and incomplete adjustment, and they were not used to support primary causal estimates. Adverse-event ascertainment was inconsistent. Some studies reported no events, but others did not specify systematic collection, definitions, surveillance periods, or denominators. The absence of reported events therefore cannot establish safety. Broader safety evidence may inform programme design [138], but this review cannot confirm safety across diagnoses, technologies, or home contexts.

The implementation literature and clinical guidance identify access, usability, clinician readiness, patient selection, caregiver availability, workflow integration, and safety planning as relevant to delivery [139,140,141]. Within this review, structured motor practice, therapist guidance, progressive difficulty, feedback, monitoring, adherence support, and safety procedures are plausible implementation considerations. They were not formally tested as moderators, and the evidence does not establish that programmes incorporating these characteristics are more effective.

### 4.3. Strengths, Limitations, and Future Directions

Strengths include prospective registration, duplicate screening and extraction, explicit outcome and comparator hierarchies, RoB 2 and ROBINS-I assessment, finalized GRADE profiles, and separation of primary, exploratory, single-study, non-inferiority, and delivery-model evidence. Study-level extraction tables preserve population, intervention, dose, comparator, outcome, adherence, safety, and follow-up detail. The search covered five major databases and the executed strings and yields are reported transparently. Domain-specific synthesis and independent-effect rules also reduced, but could not eliminate, inappropriate clinical aggregation and outcome multiplicity. No single pooled analysis was allowed to dominate interpretation of the broader evidence map.

Several limitations are substantive. The two conceptually harmonized strategies were represented by separate, verbatim strings for each database (Supplementary Table S2). Web of Science used ALL = field syntax, whereas the PubMed, Embase, Cochrane Library, and Scopus strings did not specify explicit field tags and therefore relied on each interface’s default search behaviour. No controlled-vocabulary headings, truncation, or proximity operators were specified. Database-level reporting resolves ambiguity about the searches and permits exact reproduction, but it does not eliminate potential losses in sensitivity for indexed concepts or specificity for broad technology terms; the large Scopus yield remains consistent with limited precision. The search was not peer reviewed by an information specialist. No citation tracking, reference-list searching, or supplementary searching was performed, and restriction to English-language, peer-reviewed full texts may have introduced language and publication bias. PRISMA and PRISMA-S improve reporting transparency but do not remedy these design limitations. Eligible studies may therefore have been missed, and comprehensiveness cannot be assumed from the number of records retrieved. The evidence itself was clinically and methodologically heterogeneous, with many underpowered or feasibility-oriented designs, unclear allocation concealment, incomplete blinding, comparator imbalance, missing data, and short follow-up. Most quantitative questions contained only two or three studies. Publication bias, small-study effects, prediction intervals, and meta-regression could not be assessed. REML/Hartung–Knapp reduced overconfidence for k = 3 but did not remove small-sample instability, and the high-heterogeneity k = 2 datasets support descriptive rather than generalizable inference.

SMDs enabled mathematical synthesis of different scales but did not establish clinical exchangeability or clinical importance. The primary walking/mobility activity analysis combined different diagnoses and measures, and the global motor-functional/ADL activity analysis combined related but non-identical instruments and time points. Large SMDs in small heterogeneous samples can be unstable and do not map directly to minimal clinically important differences. Interpretation also depends on baseline severity, scale reliability, within-study variance, comparator dose, and whether change scores or final values were available. Some studies were excluded from pooling because variance data, time points, comparators, or constructs were incompatible. Multicomponent programmes also made it difficult to isolate remote delivery from motor, cognitive, educational, psychological, caregiver, or adherence support. These constraints limit certainty and generalizability.

Future trials should move toward larger multicentre designs, concealed allocation, prospectively registered protocols, prespecified primary motor outcomes, intention-to-treat analyses, matched-contact comparators, standardized adverse-event reporting, and transparent handling of missing data. Intervention reporting should follow structured frameworks such as TIDieR, including telehealth-specific extensions such as TIDieR-Telehealth where appropriate, and exercise-specific guidance such as CERT, because dose, progression, feedback, therapist contact, caregiver role, safety monitoring, and technology support strongly influence reproducibility [142,143,144]. Core outcome sets and standardized measurement recommendations should also be adopted for stroke sensorimotor recovery, upper-limb quality, balance, and mobility, so that future trials can be pooled without losing clinical meaning [145,146]. The next generation of telerehabilitation research should therefore clarify not only whether remote rehabilitation can work, but for whom, through which delivery model, at what dose, and with what level of clinical supervision.

## 5. Conclusions

Comparative evidence for telerehabilitation across neurological diagnoses and motor domains remains uncertain. Both three-study primary analyses yielded point estimates favouring telerehabilitation, yet REML/Hartung–Knapp confidence intervals crossed the null and certainty was very low. High-heterogeneity two-study datasets for balance-related activity/postural control and upper-limb impairment were descriptive and do not support generalizable pooled effects.

Superiority over usual or minimal care, non-inferiority to dose-matched in-person rehabilitation, and comparisons between remote models address distinct questions. Structured motor practice, therapist guidance, progressive difficulty, monitoring, adherence support, and safety procedures are plausible implementation considerations; they were not formally tested as moderators and cannot be identified as characteristics associated with greater efficacy.

Small samples, wide intervals, clinical heterogeneity, risk of bias, comparator imbalance, and inconsistent adverse-event ascertainment preclude claims of established clinical benefit or safety. The absence of reported adverse events cannot establish that harms were systematically sought or excluded. Interpretation must remain specific to population, outcome construct, comparator, and technology. Adequately powered, prospectively registered trials with concealed allocation, matched-contact comparators, prespecified domain-specific outcomes, standardized adverse-event definitions and collection, and longer follow-up are required.

## Figures and Tables

**Table 1:** Included study characteristics and analysis classification.

Study/Country	Setting	Neurological Disorder	Design	Population/Stage and Sample	Comparator	Main Motor Domain/Outcomes	Analysis Classification
García-Rudolph et al. 2024 [36] Spain	Institut Guttmann, Barcelona; home teleSCI vs. inpatient rehabilitation unit	Spinal cord injury	Matched case–control	Sample/analysis: teleSCI n = 42; 1:1 matched historical controls n = 42; full historical cohort n = 613 Adults with traumatic or non-traumatic SCI admitted within 2 months post-injury; medically stable, predominantly paraplegia and motor-incomplete SCI	Matched historical in-person inpatient rehabilitation	Global motor-functional and mobility: FIM, SCIM III, WISCI II; gains, efficiency and effectiveness	Narrative/sensitivity only; non-randomized matched case–control; median/IQR data
Thielbar et al. 2020 [37] USA	Home-based intervention after rehabilitation-hospital orientation	Stroke	Randomized two-arm crossover trial	Sample/analysis: 24 enrolled; 20 completed Chronic stroke survivors with moderate upper-extremity impairment; mean 6.5 years post-stroke	Single-user VERGE VR mode	Upper-limb training activity and impairment: arm displacement/session, session time, FMA-UE, compliance	Delivery-model comparison only; both phases were home VR telerehabilitation
Adams et al. 2023 [38] USA	Regional health-system recruitment with home intervention and outpatient assessments	Stroke	Rater-blinded RCT	Sample/analysis: 21 randomized; 18 completed Adults with stroke affecting hand function and sufficient upper-limb active movement to use the GRASP system	Usual and customary care	Upper limb: FMUE, WMFT, BBT, MAL	Primary RCT candidate
Allegue et al. 2022 [39] Canada	Montreal community/home-based chronic stroke rehabilitation	Stroke	Two-arm randomized feasibility clinical trial	Sample/analysis: 11 randomized; 9 analyzed Chronic stroke survivors with residual upper-extremity impairment after discharge from rehabilitation services	Conventional home GRASP programme	Upper limb: FMA-UE, MAL-30; motor-related SIS-16 hand function and mobility	Sensitivity/narrative only; small feasibility trial
Asano et al. 2021 [40] Singapore	Community/home after acute hospital recruitment	Stroke	Parallel two-arm evaluator-blinded RCT	Sample/analysis: 124 randomized; 98 analyzed at 3 months Adults aged ≥40 years with recent stroke within 4 weeks and planned home/community discharge; caregiver required during exercises	Usual rehabilitation care/centre-based outpatient rehabilitation as needed	Global function and mobility: LLFDI disability, timed 5 m walk, 2MWD, modified BI, ABC scale	Effect-level RCT candidate; self-reported primary outcome
Aytutuldu et al. 2024 [41] Turkey	Outpatient Parkinson’s disease cohort with synchronous home telerehabilitation	Parkinson’s disease	Single-blind RCT	Sample/analysis: 34 randomized/ITT; 32 completed Parkinson’s disease, Hoehn–Yahr stage 1–3, age 40–72 years, independent ambulation	Progressive structured mobility training via synchronous telerehabilitation	Balance/gait: Mini-BESTest, TUG, Kinovea gait parameters, Biodex postural stability, ABC-SF, PAS	Delivery-model comparison only; both arms received synchronous telerehabilitation
Ballester et al. 2017 [42] Spain	Barcelona rehabilitation units with home-based intervention	Stroke	Parallel-group randomized controlled trial	Sample/analysis: 39 assigned; 35 analyzed Chronic first-ever stroke > 12 months with mild-to-moderate upper-limb hemiparesis	Home-based conventional occupational-therapy task	Upper limb: UE-FM, CAHAI; BI, MRC, grip force; NBS markers of cortical reorganization	RCT/sensitivity candidate; home VR vs. matched occupational-therapy task
Benvenuti et al. 2014 [43] Italy	Tuscany community/home and kiosk-based programme	Stroke	Longitudinal cohort with geographic control	Sample/analysis: treatment n = 143; usual care n = 45 Chronic stroke with upper-limb paresis; stroke onset ≥ 3 months; age ≥ 40 years	Usual care in municipalities without kiosks	Upper limb and ADL: Motricity Index, WMFT, 9-HPT, Barthel Index, NEADL, SPPB, SIS	Sensitivity/narrative only; non-randomized geographic control
Calabrò et al. 2023 [44] Italy	IRCCS Centro Neurolesi Bonino Pulejo and IRCCS San Camillo with home training	Severe acquired brain injury	Multicentre RCT	Sample/analysis: 40 randomized; 20 teleneuro-VRRS, 20 UTRT Adults aged 18–75 years with severe acquired brain injury of vascular or traumatic etiology, ≥6 months post-event, with caregiver available	Usual territorial rehabilitative treatment at home	Global motor-functional/balance: BI, Tinetti Scale, MAS; cognitive, QoL and caregiver burden measures	Narrative/effect-size candidate; mixed motor-cognitive VRRS intervention
Carey et al. 2007 [45] USA	Home-based computerized training with laboratory assessments	Stroke	Randomized trial with crossover component	Sample/analysis: 25 consented; 20 analyzed at post-test Chronic stroke ≥ 12 months; age 30–80 years; at least 10 degrees voluntary index-finger extension	Simple finger/wrist movement telerehabilitation; crossover to tracking	Upper limb: Box and Blocks Test, Jebsen–Taylor test, finger ROM, fMRI activation	Delivery-model comparison only; tracking vs. simple movement telerehabilitation
Chae et al. 2020 [46] South Korea	Community health centres with home-based training	Stroke	Prospective comparative study	Sample/analysis: HBR n = 17 and control n = 6 analyzed at 12 weeks Chronic stroke > 6 months with mild-to-moderate hemiplegia; age 40–70 years; K-MMSE ≥ 24	Conventional home exercise handout with weekly calls	Upper limb: WMFT, FMA-UE, grip power, shoulder ROM; BDI	Sensitivity/narrative only; non-randomized prospective comparative study
Chantanachai et al. 2025 [47] Thailand	Home intervention with assessments at Mahidol University	Spinal cord injury	Double-blind randomized sham-controlled trial	Sample/analysis: 30 randomized; 15 active tDCS, 15 sham tDCS Adults with traumatic or non-traumatic SCI, AIS A-D, 1–30 months post-injury, age 18–70 years	Sham tDCS plus the same tele-supervised exercise programme	SCI motor/sensory and function: ISNCSCI UEMS/LEMS/sensory, SCIM-III, TAI, H-reflex/m-MAS, HHD	Adjunct neuromodulation/sensitivity only; not a primary telerehabilitation effect
Chen et al. 2017 [48] China	Shanghai home-based tele-supervision vs. outpatient rehabilitation	Stroke	Assessor-blinded RCT	Sample/analysis: 54 randomized; 27 HTR, 27 COR Stroke survivors with hemiplegia, 14–90 days post-stroke, NIHSS 2–20 and mRS 1–5	Conventional outpatient rehabilitation with the same exercises and ETNS	ADL/balance and neuromuscular function: MBI, BBS, mRS, RMS of ECRL/TA, CSI	Primary RCT candidate; telerehabilitation vs. outpatient rehabilitation
Chen et al. 2020 [49] China	Shanghai home-based motor telerehabilitation vs. outpatient conventional rehabilitation	Stroke	Randomized controlled trial	Sample/analysis: 52 randomized; 26 TR, 26 CR First-onset subcortical stroke involving the motor pathway, 1–3 weeks after onset, NIHSS 2–20	Conventional rehabilitation in the outpatient department	Global motor and ADL: FMA, MBI; MRI markers including M1-M1 rsFC, M1 GMV and CST integrity	Primary RCT candidate; telerehabilitation vs. conventional rehabilitation
Chen et al. 2021 [50] Taiwan	Hospital-based simulated-home telerehabilitation environment	Stroke	Prospective case–control pilot randomized study	Sample/analysis: 30 enrolled; 15 interactive telerehabilitation, 15 conventional physiotherapy Chronic stroke > 6 months with motor deficits; Brunnstrom stage II–V	Conventional one-on-one physiotherapy	Balance/mobility: BBS, TUG, MFES, Motricity Index, FAC	Sensitivity/narrative; simulated-home pilot with active comparator
Chumbler et al. 2012 [51] USA	Three Veterans Affairs medical centres and participants’ homes	Stroke	Multisite single-blinded RCT	Sample/analysis: 52 randomized; 48 baseline assessments Community-dwelling veterans with ischemic or hemorrhagic stroke within the preceding 24 months	Usual care/routine rehabilitation as prescribed	Global motor-functional/disability: Motor FONEFIM, LLFDI function and disability domains	Primary RCT candidate; multifaceted telerehabilitation vs. usual care
Chung et al. 2020 [52] Hong Kong	Post-discharge home exercise after inpatient stroke rehabilitation	Stroke	Single-blind RCT	Sample/analysis: 56 analyzed; 27 video-guided, 29 paper-based Post-discharge stroke patients with MFAC 2–5 and access to a smartphone/tablet or caregiver support	Paper-based home exercise programme	Mobility/ADL and adherence: MFAC gain, MBI gain, exercise adherence, SEE	Sensitivity/digital home exercise; video-guided vs. paper-based delivery
Cikajlo et al. 2012 [53] Slovenia	University Rehabilitation Institute Ljubljana, with smart-home/home continuation after clinical training	Stroke	Pilot controlled comparative study	Sample/analysis: VRBT n = 6; balance training control n = 20 analyzed Adults after stroke able to stand and walk at least 10 m; VRBT participants were 2–8 months post-stroke and required balance rehabilitation	Standing-frame balance training without VR or telerehabilitation support	Balance/mobility: BBS, TUG, 10 m walk test, single-limb stance and VR task performance metrics	Narrative/sensitivity only; very small pilot and shared/previous-study control group
Conroy et al. 2018 [54] USA	VA Maryland Health Care System/community home-based MS rehabilitation	Multiple sclerosis	Randomized single-blind controlled trial	Sample/analysis: 51 consented; MS HAT n = 26 and control n = 25 randomized; completers n = 16 and n = 8 Adults with MS, PDDS 2–6, able to complete T25FW, mostly progressive MS with ambulatory disability	Routine individualized home exercise programme without HAT access	Gait/balance: T25FW, 6MWT, BBS and MSWS-12	Sensitivity/narrative only; RCT with high attrition and small completer sample
Coulter et al. 2017 [55] Scotland, UK	Queen Elizabeth National Spinal Injuries Unit and community home programme	Spinal cord injury	Pilot RCT with 2:1 allocation	Sample/analysis: 24 randomized; analyzed intervention n = 15 and usual-care control n = 6 Adults with SCI living in central/west Scotland, using manual wheelchair or walking with/without aids, and not exercising regularly twice weekly	Usual care/self-management	Mobility/physical capacity: 6MWT or 6MPT; HR/RPE, muscle strength, HADS and WHOQOL-BREF	Sensitivity/narrative only; pilot RCT with heterogeneous SCI mobility status and small control group
Cramer et al. 2019 [56] USA	11 NIH StrokeNet sites, home telerehabilitation versus outpatient clinic therapy	Stroke	Assessor-blinded non-inferiority randomized clinical trial	Sample/analysis: 124 randomized; 62 telerehabilitation and 62 in-clinic therapy Adults 4–36 weeks after ischemic or hemorrhagic stroke with arm motor deficit, FM arm score 22–56/66	Dose-matched and intensity-matched in-clinic arm motor therapy plus stroke education	Upper limb: FM arm motor score, Box and Blocks Test, SIS hand domain and stroke knowledge	Delivery-model/core non-inferiority comparison; telerehabilitation versus dose-matched in-clinic therapy
Dastan et al. 2025 [57] Turkey	Home-based MS telerehabilitation with university/neurology recruitment	Multiple sclerosis	Single-blind randomized study	Sample/analysis: 30 included; 25 analyzed after dropouts, synchronized n = 12 and asynchronous n = 13 Adults with MS and self-reported restriction in hand–arm function/fine-motor skills during daily activities	Asynchronous telerehabilitation using the same exercise videos	Upper limb: N-HPT, JAMAR grip strength, AMSQ; fatigue, QoL, participation, physical activity and pain	Delivery-model comparison only; synchronous versus asynchronous telerehabilitation
Van den Berg et al. 2016 [58] Australia	Adelaide stroke/rehabilitation units with home continuation after discharge	Stroke	Pragmatic randomized proof-of-concept trial	Sample/analysis: 63 randomized; intervention n = 31 and control n = 32; home telerehabilitation per-protocol n = 20 Early post-stroke rehabilitation phase, 24 h to 3 months post-stroke, mobility problems and available caregiver	Usual inpatient and home rehabilitation care	Mobility/ADL: SIS mobility, Rivermead Mobility Index, TUG, Barthel Index, NEADL, Fugl-Meyer lower extremity, Motricity Index, BBS	Sensitivity/hybrid caregiver-mediated intervention; e-health and telerehabilitation component evaluated in per-protocol analyses
Deng et al. 2012 [59] USA	University of Minnesota home training with laboratory gait and fMRI testing	Stroke	Pilot randomized controlled trial	Sample/analysis: 19 randomized; 16 completed, 8 complex tracking and 8 simple movement Chronic stroke, ≥5 months post-stroke, impaired paretic ankle dorsiflexion and ability to ambulate 30 m	Dose-matched simple ankle movement telerehabilitation	Walking/mobility activities: paretic ankle dorsiflexion during swing, 10 m walk test, motion-capture gait analysis and fMRI activation	Delivery-model comparison only; complex versus simple ankle telerehabilitation
Van der Kolk et al. 2019 [60] Netherlands	Radboudumc outpatient recruitment with fully home-based intervention	Parkinson’s disease	Single-centre double-blind RCT	Sample/analysis: 130 randomized; primary analysis n = 125, aerobic n = 61 and active control n = 64 Sedentary adults with mild Parkinson’s disease, Hoehn and Yahr stage ≤ 2, age 30–75 years, stable medication or untreated stable status	Home-based stretching, flexibility and relaxation active control with matched coaching/app support	Disease-specific motor and mobility: MDS-UPDRS III OFF, 6MWT, Mini-BESTest, TUG and fitness outcomes	Primary/sensitivity active-control candidate; home aerobic exergaming versus active non-aerobic control
Dogan et al. 2023 [61] Turkey	Hacettepe University, home mobile-app telerehabilitation versus clinic-based V-TOCT	Multiple sclerosis	Assessor-blinded prospective two-arm RCT	Sample/analysis: 34 randomized; 32 analyzed, TR n = 15 and V-TOCT n = 17 Adults with MS, age 18–55 years, EDSS 2–5.5, Mini-Mental State Examination ≥24 and smartphone access	Clinic-based virtual-reality-supported task-oriented circuit therapy	Trunk/upper limb: TIS, K-ICARS, ABILHAND, Minnesota Manual Dexterity Test and inertial-sensor kinematics	Delivery-model/active-comparator comparison; not primary telerehabilitation versus non-telerehabilitation pooling
Eldemir et al. 2023 [62] Turkey	Gazi University/Ankara University, home videoconference telerehabilitation	Parkinson’s disease	Evaluator-blind randomized controlled trial	Sample/analysis: 32 randomized; 30 analyzed, TOCT-TR n = 15 and control n = 15 Adults with Parkinson’s disease, age 45–70 years, Hoehn and Yahr stage I–III and MMSE ≥ 24	Home exercise programme targeting balance, walking and mobility activities	Upper limb and disease-specific motor symptoms: 9-HPT, JHFT, grip and pinch strength, UPDRS-III; ADL/QoL: UPDRS-II, PDQ-8	Primary RCT candidate; telerehabilitation task-oriented upper-limb training added to home exercise
Eldemir et al. 2024 [63] Turkey	Home videoconference Pilates telerehabilitation with university outpatient recruitment	Multiple sclerosis	Assessor-blinded RCT	Sample/analysis: 30 randomized/analyzed; Pilates-TR n = 15 and waitlist control n = 15 Adults with MS, age 18–65 years, EDSS 0–5 and relapse-free for the previous 3 months	Waitlist/no Pilates telerehabilitation during the study period	Balance/gait/global physical performance: BBS, Biodex posturography, 6MWT, G-Walk gait parameters, muscle strength, core endurance/power, FSS/FIS, MSQOL-54	Primary RCT candidate; telerehabilitation versus waitlist/no intervention
Fjeldstad-Pardo et al. 2018 [64] USA	Oklahoma Medical Research Foundation MS Center and home-based remote PT	Multiple sclerosis	Randomized three-arm evaluator-blinded feasibility pilot study	Sample/analysis: 30 randomized; final n = 29, HEP n = 10, TR n = 10 and in-person PT n = 9 Adults with MS and ambulatory deficits; cohort EDSS approximately 4.3 and mixed relapsing–remitting/progressive phenotypes	Customized unsupervised home exercise programme and in-person PT comparator arms	Gait/balance: FGA, T25FW, BBS, NeuroCom gait/balance metrics; ABC, SF-36, MFIS and MSSE	Sensitivity/multiple-comparator feasibility study
Fluet et al. 2024 [65] USA	Home-based HoVRS intervention with NJIT/Rutgers research support	Stroke	Parallel randomized controlled trial	Sample/analysis: 33 randomized, enhanced motivation/scaffolding n = 17 and algorithm-control n = 16; 28 completed Adults 20–80 years, ≥6 months post-stroke, moderate-to-mild upper-extremity hemiparesis and UEFMA 10–60/66	Algorithm-controlled home virtual rehabilitation games with the same activities	Upper limb/adherence: UEFMA, ARAT, SIS hand/ADL/participation subscales, IMI and system-recorded training time	Delivery-model/game-design comparison only; both arms received sparsely supervised home VR rehabilitation
Flynn et al. 2021 [66] Australia	University physiotherapy clinic and participants’ homes	Parkinson’s disease	Pilot randomized feasibility trial	Sample/analysis: 40 randomized after 5-week common block; home-based n = 20 and centre-based n = 20; analyzed home n = 19 Community-dwelling people with mild-to-moderate Parkinson’s disease, stable medication, mean age about 72 years	Predominantly centre-based exercise programme	Balance/gait feasibility outcomes: Mini-BESTest, 10 m walk test and New Freezing of Gait Questionnaire	Delivery-model/sensitivity analysis; feasibility and acceptability focus
Frevel et al. 2015 [67] Germany	Academic teaching hospital/home e-Training and therapeutic riding centre	Multiple sclerosis	Randomized controlled study	Sample/analysis: 18 randomized; 16 completed after one dropout per group Adults with clinically definite MS, EDSS 2.0–6.0, age 18–60 years, clinically stable and able to stand for 1 min	Hippotherapy active comparator	Balance/mobility: BBS, DGI, 2MWT; isometric strength, fatigue and QoL	Sensitivity/active-comparator trial; small sample
Gandolfi et al. 2017 [68] Italy	Four Veneto neurorehabilitation units with in-home TeleWii training	Parkinson’s disease	Multicentre single-blind RCT	Sample/analysis: 76 randomized; 38 TeleWii and 38 in-clinic SIBT; 70 completed with imputation used Adults with Parkinson’s disease, modified Hoehn and Yahr 2.5–3, stable medication and available caregiver	In-clinic sensory integration balance training	Balance/gait: BBS, DGI, 10-MWT gait speed, ABC, PDQ-8 and falls	Delivery-model/sensitivity comparison; in-home VR telerehabilitation versus in-clinic active rehabilitation
Garcia et al. 2022 [69] Spain	Universitat Politecnica de Catalunya/ADFO with home-based Muvity telerehabilitation	Stroke	Feasibility crossover study	Sample/analysis: 10 randomized; 6 completed the full crossover and follow-up analysis Chronic post-stroke adults with residual upper-limb ROM sufficient for ADL-oriented tasks and modified Rankin Scale ≤ 3	Conventional self-directed home therapy without the Muvity platform	ROM/global function and balance: shoulder/elbow/pelvis ROM, FIM, Berg Balance Scale, VAS pain, SF-36 PCS and usability	Narrative/sensitivity only; small feasibility crossover trial
Ge et al. 2024 [70] China	Department of Rehabilitation Medicine, Peking Union Medical College Hospital, Beijing; home-based HPT versus app-supported telerehabilitation	Parkinson’s disease	Single-centre home-based randomized controlled trial	Sample/analysis: 190 randomized after in-person eligibility assessment (HPT n = 100; TR n = 90); 19 withdrew during treatment and 27 completed training but missed the 4-week assessment Adults aged 60–80 years with mild-to-moderate primary Parkinson’s disease, Hoehn and Yahr stages I–III, no exercise programme in the previous 4 weeks, and adequate mobile-phone skills	Home physical therapy delivered by licenced therapists at the patient’s home, with the same overall home exercise framework	Disease-specific motor, balance, gait and ADL: UPDRS III primary outcome; BBS, TUG, FTSST, FOGQ, IDEEA gait parameters, isokinetic knee strength, UPDRS II and PDQ-39	Active-comparator RCT; delivery-model/sensitivity analysis because telerehabilitation was compared with supervised home physical therapy rather than usual care
Ginis et al. 2016 [71] Belgium and Israel	Home-based gait training after recruitment from KU Leuven and Tel Aviv Sourasky Medical Center	Parkinson’s disease	Pilot randomized controlled trial with stratified blocked allocation	Sample/analysis: 40 randomized (CuPiD n = 22; active control n = 18); 38 completed training and 39 completed the 4-week retention assessment People with Parkinson’s disease, Hoehn and Yahr stages II–III in the ON state, stable medication, MoCA ≥ 24, and able to walk continuously for 10 min; participants with and without freezing of gait were included	Personalized gait advice active control with the same visit schedule and home-practice expectations but without smartphone/wearable feedback	Gait, balance and endurance: usual and dual-task gait speed primary outcomes; 2MWT, Mini-BESTest, FSST, FOGQ, ABC and SF-36 physical health	Pilot active-comparator RCT; sensitivity candidate for wearable-feedback telerehabilitation, with limited power and non-blinded assessors
Goffredo et al. 2023 [72] Italy	Five IRCCS rehabilitation hospitals within the Italian Neuroscience and Rehabilitation Network, with home-based treatment delivery	Parkinson’s disease	Multicentre randomized controlled trial with blinded assessors	Sample/analysis: 105 randomized (TR n = 54; control n = 51); 97 analyzed (TR n = 49; control n = 48) Individuals with Parkinson’s disease, Hoehn and Yahr ≤ 3 in ON state, age ≤ 80 years, MoCA ≥ 17.54, stable medication, and 6MWT distance 200–600 m; moderate/severe dyskinesia or freezing was excluded	At-home self-administered structured conventional motor activities using a tailored written booklet, with matching intensity and duration	Balance/postural stability and mobility: Mini-BESTest primary outcome; TUG, 6MWT and MDS-UPDRS Part III secondary motor outcomes	Primary RCT candidate for telerehabilitation versus home conventional exercise; nonimmersive VR-based balance-focused intervention
Guo et al. 2023 [73] China	Tangdu Hospital and Xi’an Gaoxin Hospital rehabilitation institutions; training conducted in rehabilitation halls with remote-system data upload	Stroke	Randomized parallel-controlled two-centre pilot trial	Sample/analysis: 120 inpatients randomized (remote-system + PT n = 60; routine OT + PT n = 60); 109 completed the 3-week protocol Inpatients aged 30–75 years with CT/MRI-confirmed stroke, limb motor dysfunction, Brunnstrom upper- or lower-extremity stages II–VI, and 15–180 days since onset	Routine clinical occupational therapy plus routine clinical physical therapy and drug treatment	Global motor function: Fugl-Meyer Assessment total score, upper-extremity subscore and lower-extremity subscore at baseline and 3 weeks	Sensitivity/narrative only; rehabilitation-hall wearable remote system plus PT rather than a conventional home telerehabilitation trial
Hartung et al. 2025 [74] Germany	Eight centres in southern Germany, including rehabilitation centres, hospitals and resident neurologists; Internet-based home programme	Multiple sclerosis	Multicentre randomized waitlist-controlled study	Sample/analysis: 56 persons analyzed (intervention n = 29; waitlist/usual care n = 27), with measurements at baseline, 12 weeks and 24 weeks Adults with multiple sclerosis according to McDonald criteria, EDSS 0–6.5, no exacerbation within 30 days, wireless Internet, smartphone and basic digital skills; regular exercisers were excluded	Waitlist/usual care during the first 12 weeks, followed by delayed access to the programme	Physical activity and mobility-related outcomes: device-measured steps/day primary outcome; MVPA, sport/exercise minutes, PAHCO, T25FW, 2MWT, MSWS-12, MSIS-29, fatigue and depression	Adjacent/sensitivity only; Internet-based exercise and physical-activity promotion rather than a disorder-specific motor telerehabilitation trial
Hernandez et al. 2022 [75] Canada	Montreal, Quebec; home-based chronic stroke upper-limb programme with remote therapist monitoring	Stroke	Evaluator-blinded parallel two-arm randomized controlled trial	Sample/analysis: 53 consented, 51 completed/analyzed (Jintronix n = 26; GRASP standard care n = 25) Chronic first-time stroke > 6 months with residual mild-to-moderate upper-extremity impairment (Chedoke-McMaster arm component 2–6) and no ongoing rehabilitation services	Standardized Graded Repetitive Arm Supplementary Program (GRASP) home exercise kit/manual without ongoing therapist supervision	Upper limb: FMA-UE primary outcome; Stroke Impact Scale domains and abridged MAL-14; feasibility measures including sessions and active playing time	Active-comparator RCT/sensitivity candidate for home VR telerehabilitation versus evidence-based home exercise
Hong et al. 2025 [76] China	Yuebei People’s Hospital and Shaoguan First People’s Hospital; home-based telerehabilitation and in-person occupational-therapy comparison	Stroke	Retrospective cohort non-inferiority study using historical intervention groups	Sample/analysis: 96 recruited, 86 grouped, and 79 completed (Tele-Rehab n = 23; in-person TOT n = 28; Tele-Control n = 28) Adults aged 18–75 years with first-onset ischemic or hemorrhagic stroke, 3–24 months post-stroke, upper-limb/hand motor dysfunction, Brunnstrom stage 1–4, FAC ≥ 3, and mild-to-moderate spasticity	In-person task-oriented training with identical task content and a tele-control neurofacilitation programme delivered by pre-recorded videos with weekly check-ins	Upper limb and ADL: FMA-UE, WMFT-FAS, ARAT and IADL after 3 weeks; non-inferiority was defined using the FMA-UE change-score margin	Narrative/sensitivity only; retrospective, non-randomized non-inferiority cohort with multiple active comparators
Huijgen et al. 2008 [77] Italy, Spain and Belgium	UORIN Trevi, Institut Guttmann Badalona and National MS Centre Melsbroek, with HCAD installed at home	Mixed neurological disorders: stroke, traumatic brain injury and multiple sclerosis	Randomized multicentre clinical trial	Sample/analysis: 81 recruited and randomized (HCAD pathway n = 55; usual care n = 26); 70 analyzed after 11 losses to follow-up Adults living at home with stable stroke, MS or TBI and impaired arm/hand function (NHPT > 25 s but able to move at least one peg within 180 s), sufficient autonomous function and Internet/telephone access	Usual care and generic exercises prescribed by physicians	Upper limb/hand function: ARAT and Nine-Hole Peg Test primary outcomes; participant and therapist satisfaction with HCAD	Narrative/sensitivity only; mixed diagnostic sample and feasibility/equality design rather than diagnosis-specific RCT pooling
Jarbandhan et al. 2022 [78] Suriname	Paramaribo community/home-based SunRISe study linked to Anton de Kom University and Academic Hospital Paramaribo	Stroke	Prospective randomized controlled pilot study with 2:1 allocation	Sample/analysis: 30 chronic stroke participants randomized (intervention n = 20; usual care n = 10); 14 intervention participants completed the programme Community-dwelling chronic stroke survivors ≥ 6 months post-stroke, mild-to-moderate deficit, FAC ≥ 3, MMSE > 24, medical clearance for moderate exercise, and not already receiving regular exercise or physiotherapy	Usual care in the local low-resource context	Mobility and functional capacity: 6MWT primary efficacy outcome; BBS, DASH, paretic and non-paretic handgrip strength, exercise self-efficacy, adherence, satisfaction and safety	Pilot RCT/sensitivity candidate; hybrid home physiotherapy with telephone tele-coaching in a low-resource setting
Johnson et al. 2020 [79] Australia	Screening/testing in a university clinic, with virtual therapy delivered in community-based stroke support-group settings in Melbourne, Victoria	Stroke	Assessor-blinded RCT	Sample/analysis: 60 randomized; 58 analyzed Community-dwelling stroke survivors > 3 months post-stroke with mild-to-moderate upper-extremity impairment	Usual care/maintenance of usual activity and treatment plans	Upper limb: FMUE, ARAT, BBT, MAS, MAL-28 and EQ-5D-5L	Primary/sensitivity candidate
Johnson et al. 2024 [80] USA	Cleveland Clinic outpatient neurorehabilitation clinics in Cleveland, Ohio and Las Vegas, Nevada, with web/mobile home exercise delivery	Parkinson’s disease	Unblinded three-arm pilot randomized controlled trial	Sample/analysis: 20 randomized (clinic + TR n = 6; TR-only n = 6; usual care n = 8); 19 completed after one unrelated injurious fall withdrawal Individuals with Parkinson’s disease in early-to-moderate stages who were safe for participation (TUG < 15 s, 10 m gait speed > 0.8 m/s) and had home Internet/caregiver support when required	Usual care: weekly in-clinic physical therapy plus daily therapist-prescribed paper home exercise programme; clinic + TR was a hybrid comparator arm	Feasibility and movement-related outcomes: recruitment/retention/satisfaction primary feasibility outcomes; TUG, TUG-cognitive, 5STS, 10MWT, 6MWT, Mini-BESTest, PDQ-39 and ABC	Pilot/sensitivity only; very small feasibility RCT with three delivery arms and exploratory clinical outcomes
Kintrilis et al. 2024 [81] Greece	Neurological clinic/local third-level clinic and 414 Military Hospital of Special Diseases, Penteli; in-person and teleconferencing rehabilitation sessions	Stroke	Randomized controlled study	Sample/analysis: 90 randomized; in-person resistance training n = 30, teleconference resistance training n = 30 and usual care n = 30 Adults recovering after stroke in an acute/post-discharge rehabilitation context	In-person resistance training and usual care	Walking/mobility activities and functional capacity: TUG, BBS, Chair Stand Test, 6MWT, VAS health score and A-VO2	Sensitivity/delivery-model; teleconference versus in-person resistance training with usual-care reference group
Kowalczewski et al. 2011 [82] Canada	University of Alberta in-home teletherapy, with additional anecdotal non-pooled participants from Melbourne, Australia	Spinal cord injury	Blindly evaluated randomized crossover trial	Sample/analysis: 13 participants with C5–C7 tetraplegia; 18 hands contributed to the crossover comparison of conventional ET and ReJoyce ET Adults with chronic C5–C7 tetraplegia ≥ 9 months, able to lift/place the hand onto a table and to enhance grasp/release with FES; severe contractures, tendon transfers and FES-unresponsive hand muscles were excluded	Conventional in-home teletherapy exercise therapy comprising strength training, trackball computer games and therapeutic electrical stimulation	Upper-limb/hand function: ARAT primary outcome; grasp and pinch forces and ReJoyce Automated Hand Function Test secondary outcomes	Delivery-model comparison only; both interventions used supervised home-based telerehabilitation
Lee et al. 2022 [83] South Korea	Pusan National University Yangsan Hospital, with real-time videoconference dance therapy delivered in an independent space simulating home telerehabilitation	Stroke	Pilot randomized controlled non-inferiority study	Sample/analysis: 17 sub-acute/chronic stroke inpatients randomized; 14 analyzed (experimental n = 7; control n = 7) Sub-acute or chronic stroke 1–24 months after onset, able to sit independently, walk 10 m independently or with minimal assistive device, and tolerate 40 min of activity	Conventional physical therapy during the same period; both groups otherwise continued conventional therapy	Trunk control and balance: TIS primary outcome; BBS, TUG, FAC, K-MBI and EQ-5D secondary outcomes	Pilot/sensitivity only; small non-inferiority study of adjunct telerehabilitation dance therapy
Lee et al. 2025 [84] Taiwan	Shuang Ho Hospital, Taipei Medical University, New Taipei City; stroke inpatient wards during the pandemic period	Stroke	Prospective parallel single-blind pilot randomized controlled trial	Sample/analysis: 24 hospitalized stroke inpatients randomized (TR n = 12; bedside control n = 12); 22 included in intention-to-treat analysis after two early exclusions First-time ischemic or hemorrhagic stroke inpatients with hemiplegia, at least 5 days post-stroke, mRS 2–4, ability to follow one-step demonstrations, supported sitting balance and use of the unaffected hand to assist the affected hand	Additional bedside occupational therapy plus regular rehabilitation, matched to telerehabilitation content as clinically appropriate	ADL, postural control and mobility: MBI, PASS and FAC; PHQ-9, Borg RPE and satisfaction were also collected	Pilot/sensitivity only; inpatient telerehabilitation feasibility study with small sample and natural-recovery confounding
Lin et al. 2014 [85] Taiwan	Three long-term care facilities in Taipei and Taichung connected to a therapist-end laboratory at National Taiwan University	Stroke	Multisite blocked-randomized pilot study	Sample/analysis: 24 chronic stroke residents randomized (Tele n = 12; conventional n = 12); all 12 Tele participants retained in intention-to-treat analysis despite one post-training dropout Chronic stroke residents living in long-term care facilities for >3 months, >6 months post-stroke, Brunnstrom upper-extremity stage ≥ 3, able to sit unsupported for 30 s and follow instructions	Conventional balance training delivered in the long-term care facilities with the same small-group format and dose	Balance and functional activity: Berg Balance Scale, Barthel Index total/self-care/mobility and participant satisfaction	Pilot/sensitivity only; small long-term-care-facility balance trial
Llorens et al. 2015 [86] Spain	Hospitales NISA neurorehabilitation unit, Valencia, comparing clinic-based and home-based VR delivery	Stroke	Single-blind randomized controlled trial	Sample/analysis: 31 randomized, 30 analyzed (in-clinic control n = 15; home telerehabilitation n = 15) Chronic outpatients with stroke, residual hemiparesis, age 40–75 years, chronicity >6 months, BBA section 3 levels 7–12, MMSE > 23 and home Internet access	In-clinic delivery of the same VR balance training system, with both groups also receiving conventional clinic physical therapy on non-VR days	Balance and locomotor skill: BBS primary outcome; POMA balance and gait subscales, BBA, usability, motivation and cost outcomes	Delivery-model RCT/sensitivity; home VR telerehabilitation versus clinic-based delivery of the same VR programme
Masbernat-Almenara et al. 2026 [87] Spain	Home-based programme recruited through the Catalan Association of Hereditary Ataxia and Hospital Clinic de Barcelona	Hereditary/cerebellar ataxia	Single-blind parallel two-arm pilot randomized controlled trial	Sample/analysis: 12 randomized and completed (mixed-format n = 6; asynchronous n = 6), with 100% retention at 12-week follow-up Adults with degenerative hereditary ataxia or another neurodegenerative disease in which ataxia was dominant, Internet/mobile access, and ability to stand feet together >10 s without support	Asynchronous core-stability telerehabilitation using the same individualized exercise-video library with weekly monitoring calls	Gait, balance and ataxia: 4MWT, TUG, 30 s sit-to-stand, S-TIS 2.0, SARA sitting/standing/walking and total severity, ABC and adherence outcomes	Delivery-format/sensitivity only; rare-disorder pilot comparing mixed synchronous/asynchronous versus asynchronous telerehabilitation
Mulder et al. 2024 [88] Netherlands	Four rehabilitation centres (Reade, Roessingh, Sint Maartenskliniek and Vogellanden) with inpatient/outpatient and home-transition blended care	Stroke	Multicentre observer-blinded parallel randomized controlled trial	Sample/analysis: 41 patient–caregiver dyads randomized; 37 analyzed (intervention n = 18; usual care n = 19) after early losses Patient–caregiver dyads within 3 months post-stroke; patients lived independently pre-stroke, were planned for home discharge or already home, had MoCA > 21 and an informal caregiver able to support exercise	Usual care alone, following Royal Dutch Society for Physical Therapy guidelines, without explicit caregiver-mediated telerehabilitation module	Mobility and function: Stroke Impact Scale mobility primary outcome; RMI, FAC, 6MWD, 5 m comfortable walking speed, Motricity Index leg score, BBS, NEADL and psychosocial/transition outcomes	Hybrid/sensitivity RCT; underpowered blended-care caregiver-mediated intervention added to usual care
Nuic et al. 2024 [89] France and the Netherlands	Paris Brain Institute and Radboud University Medical Centre, with home-based exergaming after supervised initiation	Parkinson’s disease	Prospective multicentre single-blind phase II randomized controlled trial	Sample/analysis: 50 patients with Parkinson’s disease randomized 1:1 to active full-body exergaming or keyboard-control gaming People with Parkinson’s disease aged 18–80 years with dopa-resistant gait and/or balance disorders, stable dopaminergic medication, able to stand/walk independently, MMSE ≥ 24 and no impulse-control disorder	Keyboard-control version of the same Toap Run game, played without physical effort	Gait and balance: Stand–Walk–Sit Test duration primary outcome; MDS-UPDRS parts I–IV including axial subscore, GABS-B, Tinetti, NFOG-Q, ABC, gait recordings, falls and safety	VR/exergaming RCT/sensitivity; active physical exergaming versus attention/game control, not a therapist-led telerehabilitation comparator
Ortiz-Gutiérrez et al. 2013 [90] Spain	San Carlos University Hospital/Rey Juan Carlos University and home-based Kinect telerehabilitation	Multiple sclerosis	Preliminary controlled study with two parallel treatment groups	Sample/analysis: 50 enrolled and 47 completed; experimental n = 24 and control n = 23 Adults with MS, age 20–60 years, diagnosis > 2 years, EDSS 3–5, impaired balance related to cerebellar/connection lesions, medically stable and with home Internet access	Conventional physiotherapy delivered at MS rehabilitation centres	Postural control/balance: computerized dynamic posturography using Sensory Organization Test, composite equilibrium score and sensory ratios	Sensitivity/narrative candidate; preliminary non-equivalent allocation based partly on availability/accessibility rather than full randomization
Pagliari et al. 2024 [91] Italy	Five Italian IRCCS centres with home-based VRRS telerehabilitation	Multiple sclerosis	Multicentre rater-blinded active-controlled RCT	Sample/analysis: 70 randomized; 60 completed and analyzed, TR n = 30 and usual care n = 30 Adults with MS, age 25–70 years, EDSS ≤ 6.5, clinically stable and without recent relapse or steroid treatment	Home-based conventional motor and cognitive rehabilitation using a tailored booklet with the same nominal dose	Balance/motor and participation: MSQOL-54, Mini-BESTest, BBT, 9-HPT, MSWS-12, fatigue, cognitive tests and adherence	Primary RCT candidate; home VRRS telerehabilitation versus active home usual-care rehabilitation
Paul et al. 2014 [92] Scotland, UK	Douglas Grant Rehabilitation Centre and participants’ homes	Multiple sclerosis	Randomized controlled pilot study	Sample/analysis: 30 randomized; intervention n = 15 and control n = 15; 29 analyzed at week 13 Community-dwelling adults with MS moderately affected by disability, EDSS 5–6.5, stable medication and Internet access	Usual care consisting of general exercise advice and signposting to local exercise options	Gait/balance and patient-reported impact: T25FW, BBS, TUG, MSIS, Leeds MS QoL, MS symptom checklist and HADS	Sensitivity/narrative only; pilot RCT designed to inform a definitive trial
Paul et al. 2019 [93] UK	Three MS outpatient centres and home-based rehabilitation	Multiple sclerosis	Multicentre single-blind randomized feasibility study	Sample/analysis: 90 randomized, web-based n = 45 and active-comparator n = 45 People with MS, EDSS 4–6.5, with access to computer/tablet, email and Internet; excluded if exercising regularly ≥2 times/week	Standard individualized home exercise programme provided as printed exercise sheets	Mobility/balance/physical activity: 2MWT, T25FW, TUG, BBS, steps/day, MSIS, EQ-5D and healthcare resource use	Sensitivity/feasibility only; active-comparator RCT focused on feasibility and sample-size estimation
Pavan et al. 2024 [94] Italy	Fondazione Don Carlo Gnocchi, Rome, with fully home-based robotic and non-robotic telerehabilitation	Stroke	Single-blind randomized two-arm clinical trial	Sample/analysis: 119 screened; 30 randomized, non-robotic n = 14 and robotic n = 16 Adults with sub-acute ischemic or hemorrhagic stroke, upper-limb motor impairment grade > 2 on MRC, age 25–85 years, MoCA > 17.54, time since stroke ≤ 6 months	Robotic home telerehabilitation using an end-effector device, or non-robotic home telerehabilitation depending on allocation	Upper limb/global disability: WHODAS 2.0, FMA-UE motor function, BBT, cognitive tests, QoL and clinical-functional status	Delivery-model comparison only; robotic versus non-robotic telerehabilitation without a non-telerehabilitation control
Petracca et al. 2024 [95] Italy	Sant’Andrea Hospital MS centre, Rome; telerehabilitation during COVID-19-era restrictions and onsite outpatient rehabilitation	Multiple sclerosis	Single-centre interventional comparative study	Sample/analysis: 61 recruited and 51 completed; telerehabilitation n = 25 and onsite rehabilitation n = 26 Adults with MS, EDSS 2.0–6.5, clinically stable without recent relapse, treatment modification or rehabilitation in the previous 4 weeks	Onsite supervised rehabilitation with the same duration, frequency and one-to-one format	QoL/fatigue/balance/cognition: MSQoL-54, FSS, BBS and SDMT	Sensitivity/narrative only; non-randomized allocation driven by pandemic-related service constraints
Picelli et al. 2026 [96] Italy	Verona and Venice bicentric home telerehabilitation versus outpatient rehabilitation	Stroke	Bicentric assessor-blinded non-inferiority RCT	Sample/analysis: 56 randomized, 28 telerehabilitation and 28 conventional outpatient therapy; all completed Adults 2–4 months after first-ever ischemic stroke with hemiparesis, aphasia and stroke-related cognitive impairment, with home high-speed Internet/caregiver support if needed	Conventional in-person outpatient multidisciplinary rehabilitation with matched number and duration of sessions	Multidomain motor/cognitive/language/disability: FMA-UE, FMA-LE, Italian AAT, OCS orientation/memory and BI	Primary non-inferiority RCT candidate; synchronous multidomain telerehabilitation versus conventional outpatient rehabilitation
Piron et al. 2009 [97] Italy	Home-based VRRS.net telerehabilitation connected to IRCCS San Camillo Hospital versus local health-district therapy	Stroke	Randomized single-blind controlled trial	Sample/analysis: 36 randomized, telerehabilitation n = 18 and control n = 18 Adults with single ischemic MCA stroke, 7–32 months post-stroke, mild-to-intermediate upper-limb impairment with Fugl-Meyer UE 30–55	Traditional upper-limb physical therapy in the local health district	Upper limb: Fugl-Meyer Upper Extremity, ABILHAND and Ashworth scale; follow-up at 1 month post-treatment	Primary/sensitivity RCT candidate; early VR-based telerehabilitation versus traditional physical therapy
Prukviwat et al. 2026 [98] Thailand	King Chulalongkorn Memorial Hospital and home motion-sensor telerehabilitation	Parkinson’s disease	Patient-preference non-randomized controlled pilot study with blinded assessor	Sample/analysis: 46 recruited, telerehabilitation n = 23 and hospital rehabilitation n = 23 Adults with idiopathic PD, modified Hoehn and Yahr 1.5–3, stable medication and no recent rehabilitation programme	Hospital-based rehabilitation with the same exercise programme and total number of sessions	Balance/walking/mobility activities: BBS, Chula Parkinson Mobility Scale, gait speed and step length	Sensitivity/narrative only; non-randomized patient-preference comparison
Pastana Ramos et al. 2023 [99] Brazil	Belém, Brazilian Amazon, home telerehabilitation with hospital recruitment	Parkinson’s disease	Parallel-group single-centre single-blind phase 2 RCT	Sample/analysis: 19 participants, telerehabilitation n = 8 and booklet-control n = 11 People with mild PD, Hoehn and Yahr ≤ 2, age 30–80 years, stable medication and access to teleconference technology or family assistance	Booklet-based home exercise programme with weekly telephone check for training feedback/adverse effects	Mobility and motor status: TUG, 5STS, ABC scale, MDS-UPDRS III and PDQ-8	Sensitivity/feasibility RCT candidate; small phase 2 trial in an under-represented geographic context
Salgueiro et al. 2022a [100] Spain	Barcelona neurorehabilitation clinic with home Farmalarm App guidance	Stroke	Single-blind preliminary RCT	Sample/analysis: 30 randomized, experimental n = 15 and control n = 15 Chronic stroke survivors >6 months post-stroke with hemiplegia/hemiparesis, S-TIS 2.0 ≤10 and smartphone/tablet use by patient or caregiver	Conventional individualized physiotherapy alone	Trunk/balance/gait: S-TIS 2.0, S-FIST, S-PASS, BBS, falls and G-Walk gait parameters	Sensitivity/narrative only; preliminary app-guided core-stability adjunct trial with low adherence
Salgueiro et al. 2022b [101] Spain	Catalan hospitals and home Farmalarm App after discharge	Stroke	Prospective controlled extension trial	Sample/analysis: 49 recruited at hospital discharge, AppG n = 20 and usual-care control n = 29 Subacute stroke survivors after a previous 5-week trial of conventional therapy versus core-stability exercises; recruited at discharge if patient/caregiver used smartphone	Usual post-discharge care without app-guided core-stability telerehabilitation	Sitting/standing balance and gait: S-TIS 2.0, S-FIST, BBS, S-PASS, falls, BBA stepping and G-Walk	Narrative/sensitivity only; controlled extension with non-random post-discharge app allocation inherited from prior intervention group
Saygili et al. 2024 [102] Turkey	Home videoconference telerehabilitation after neurorehabilitation outpatient recruitment	Stroke	Evaluator-blind randomized controlled trial	Sample/analysis: 18 randomized, Tele-CIMT n = 10 and control n = 8 Adults with first-ever stroke at least 1 month previously, preserved minimum wrist/finger extension, MAL-28 < 2.5 and ability to stand 2 min	Home exercise programme targeting ROM, active movement, balance and walking	Upper limb/ADL: STREAM, FM-UE, WMFT, 9-HPT, grip and pinch strength, MAL-28 and FIM	Primary/sensitivity RCT candidate; m-CIMT telerehabilitation added to home exercise versus home exercise alone
Saywell et al. 2021 [103] New Zealand	Four centres with home visits, telephone and text-message support	Stroke	Assessor-blinded parallel RCT	Sample/analysis: 95 randomized, ACTIV n = 47 and usual care n = 48 Adults > 20 years with first-ever hemispheric stroke discharged home from inpatient/outpatient/community physiotherapy and with arm and/or leg physical limitations	Usual care after discharge from rehabilitation services	Physical function and performance: SIS physical subcomponent, grip strength, Step Test, SSEQ, EQ-5D VAS and SIS domains	Primary RCT candidate for augmented community telerehabilitation; hybrid low-technology programme
Sheehy et al. 2025 [104] Canada	Ottawa home-based post-discharge nonimmersive VR telerehabilitation	Stroke	Assessor-blinded parallel feasibility RCT	Sample/analysis: 20 randomized and completed, NIVRT n = 11 and iPad active control n = 9 Patients approaching discharge from inpatient or outpatient stroke rehabilitation, able to stand independently for ≥2 min and perform mild-to-moderate exercise with a study partner	iPad applications for cognition and fine-motor skills as an active attention-control intervention	Balance/gait/function/community: BBS, TUG variants, FTSTS, CB&M, SIS and CIQ; feasibility/adherence/safety	Feasibility/narrative only; active-control pilot not powered for efficacy
Simpson et al. 2025 [105] Canada	Six sites across five provinces with fully remote assessment/intervention through CanStroke Recovery Trials Platform	Stroke	Multicentre assessor-blinded waitlist-controlled RCT	Sample/analysis: 73 randomized, V-ABC n = 36 and waitlist usual care n = 37 Community-living adults < 1 year post-stroke with unilateral upper-limb impairment, residual arm movement and reduced everyday arm use	Waitlist usual care; participants could receive ≤1 formal upper-limb therapy session/week	Real-world upper-limb activity and function: TENZR reach-to-grasp counts, REACH scale, ArmCAM and QoL-related outcomes	Primary RCT candidate for activity/adherence synthesis; wearable-feedback virtual programme versus waitlist/usual care
Skelly et al. 2026 [106] UK	Two English NHS hospitals with home videoconference physiotherapy	Parkinson’s disease	Randomized controlled feasibility trial	Sample/analysis: 40 recruited, TR n = 21 and usual care n = 19; 38 analyzed after excluding two diagnosed >4 years before study start People with early PD diagnosed within 4 years, Hoehn–Yahr stages 1–3, independently mobile and without recurrent falls/freezing of gait	Usual physician/nurse advice about exercise and physical activity; physiotherapy only if clinically required	Motor/physical activity and QoL: UPDRS, Fitbit weekly step count/activity intensity, PDQ-39, SLST, FTSTS and falls	Feasibility/narrative only; preliminary efficacy estimates for definitive RCT planning
Song et al. 2018 [107] Australia	Community-based home exergame intervention with university laboratory outcome assessment	Parkinson’s disease	Single-blinded parallel RCT	Sample/analysis: 60 randomized (exergame n = 31, control n = 29); post-test analyzed n = 28 and n = 25; 6-month falls follow-up Community-dwelling adults with idiopathic Parkinson’s disease, age ≥ 40 years, stable medication, able to walk ≥30 m unaided and without substantial cognitive impairment	Usual healthcare/no study exercise intervention	Stepping and gait/falls-related outcomes: choice stepping reaction time, Functional Gait Assessment, Timed Up and Go, gait adaptability, falls, self-reported mobility/balance	Sensitivity/narrative only; home exergame step-training RCT with largely neutral primary outcomes and subgroup signals by disease severity
Standen et al. 2017 [108] UK	Patients’ homes after discharge from formal stroke rehabilitation	Stroke	Two-group feasibility RCT	Sample/analysis: 27 randomized (VR n = 17, usual care n = 10); 18 completed final outcome measures Adults with residual arm dysfunction after stroke, no longer receiving intensive rehabilitation, able to sit and follow commands, with detectable arm movement	Usual care with outcome-assessment visits only	Upper limb: Wolf Motor Function Test, Nine-Hole Peg Test, Motor Activity Log, Nottingham Extended Activities of Daily Living	Narrative/sensitivity only; feasibility RCT with substantial support requirements and limited final completer sample
Sun et al. 2025 [109] China	Post-discharge home telerehabilitation after acute ischemic stroke	Stroke	Randomized controlled trial	Sample/analysis: 200 patients randomized 1:1 (telerehabilitation n = 100; standard care n = 100); ITT analysis reported Adults aged 18–75 years discharged after first or recurrent acute ischemic stroke, Barthel Index ≤ 95, without severe cognitive impairment	Standard post-discharge care consisting of rehabilitation manual and biweekly outpatient follow-up for 12 weeks	Global function/disability and QoL: Barthel Index, modified Rankin Scale, HAMD, WHOQOL-BREF	Primary/sensitivity candidate; broad post-discharge multidisciplinary telerehabilitation with functional primary outcome
Swarnakar et al. 2023 [110] India	Tertiary rehabilitation-care centre with telephone-based home telerehabilitation during the COVID-19 pandemic	Spinal cord injury	Prospective single-centre double-blind RCT	Sample/analysis: 30 randomized; 15 telerehabilitation and 15 control; all completed 8-week follow-up Adults with traumatic or non-traumatic SCI previously receiving inpatient/outpatient rehabilitation; predominantly young males with paraplegia and complete injuries	Standard usual care as previously advised during outpatient or inpatient rehabilitation	SCI functional independence and anxiety: SCIM III total/domains, Coronavirus Anxiety Scale	Primary/sensitivity candidate; pilot RCT in SCI with pandemic-specific telerehabilitation context
Thomas et al. 2017 [111] UK	Dorset MS service with home Nintendo Wii use and hospital orientation	Multiple sclerosis	Single-centre waitlist pilot RCT	Sample/analysis: 30 randomized; outcome data available for 29 at 6 months and 28 at 12 months Ambulatory, relatively inactive adults with clinically definite MS, APDDS compatible with mild-to-moderate disability, suitable for home Wii activity	Delayed/waitlist usual care for first 6 months	Physical activity and motor performance: GLTEQ/activPAL, balance, gait, mobility, hand dexterity, i-TUG; QoL, mood, fatigue and self-efficacy	Feasibility/narrative only; pilot RCT designed to inform definitive effectiveness/cost-effectiveness trial
Toh et al. 2025a [112] Hong Kong SAR	Community self-help groups with home wearable-based upper-limb telerehabilitation	Stroke	Single-blind RCT	Sample/analysis: 40 randomized (Smart Reminder n = 20; sham n = 20); ITT analysis with two sham-group dropouts handled by LOCF Community-dwelling stroke survivors > 3 months post-stroke with unilateral hemiparesis and moderate upper-limb impairment (FTHUE-HK 3–6)	Pictorial handout-based upper-limb training with sham accelerometer device	Upper-limb impairment/activity/use: FMA-UE, ARAT, MAL-AOU/QOM, AROM, strength, accelerometer movement counts, adherence	Primary/sensitivity candidate; wearable-assisted telerehabilitation versus active sham/handout control
Toh et al. 2025b [113] Hong Kong SAR	Community stroke support groups with smartphone-linked wearable telerehabilitation	Stroke	Single-blind randomized two-period crossover feasibility pilot	Sample/analysis: 12 randomized; 9 completed both periods after 3 unrelated dropouts Community-dwelling stroke survivors > 3 months post-stroke with hemiplegic upper-limb impairment and FTHUE-HK ≥ 3	Conventional home exercise using pictorial handouts with weekly in-person consultation	Upper limb: FMA-UE, ARAT, shoulder/elbow/forearm AROM, MAL, exercise adherence	Sensitivity/delivery-model only; feasibility crossover study of wearable-smartphone delivery versus conventional handout exercises
Uswatte et al. 2021 [114] USA	In-home Tele-AutoCITE versus in-laboratory CIMT	Stroke	Proof-of-concept randomized controlled non-inferiority trial	Sample/analysis: 24 randomized; 20 completed post-treatment testing; long-term MAL follow-up available for fewer participants Adults ≥ 1 year after stroke with persistent mild-to-moderate upper-extremity hemiparesis and substantial nonuse	Standard in-laboratory, in-person Constraint-Induced Movement Therapy	Upper limb real-world use/capacity: Motor Activity Log Arm Use scale, Wolf Motor Function Test, participant satisfaction/difficulty	Delivery-model comparison only; telehealth CIMT versus standard in-lab CIMT non-inferiority framework
Vasconcellos et al. 2023 [115] Brazil	Home-based telerehabilitation after recruitment from clinics/rehabilitation centres in Natal-RN	Parkinson’s disease	Single-blind RCT	Sample/analysis: 28 randomized (trunk exercise n = 14; control n = 14); ITT with last observation carried forward; 19 completed follow-up Idiopathic Parkinson’s disease, Hoehn and Yahr II-IV, able to understand commands and walk 10 m without personal assistance	Home-based upper- and lower-limb global exercise telerehabilitation	Balance/gait: stabilometric COP measures, gait speed, hip/knee/ankle ROM during gait, MDS-UPDRS III	Delivery/content comparison only; both groups received home-based telerehabilitation
Vloothuis et al. 2019 [116] Netherlands	Hospitals, rehabilitation centres and geriatric rehabilitation departments with continuation at home when discharged	Stroke	Observer-blinded multicentre RCT	Sample/analysis: 66 stroke patient–caregiver couples randomized/analyzed Stroke patients during inpatient rehabilitation, lived independently pre-stroke, planned home discharge, FAC < 5, MMSE > 18, with eligible caregiver	Usual care according to Dutch physical therapy guidelines	Mobility/ADL and transition outcomes: SIS mobility, length of stay, FMA-LE, Motricity Index, 6MWT, 10MWT, TUG, BBS, RMI, BI, NEADL, mRS	Primary/sensitivity effect-level candidate; caregiver-mediated e-health/telerehabilitation added to usual care
Wang et al. 2024 [117] China	Post-discharge Internet remote home rehabilitation with wearable lower-limb device training	Stroke	Randomized controlled study	Sample/analysis: 80 patients allocated by odd/even numbering (observation n = 40; control n = 40) First-onset stroke patients with hemiplegia, age 45–70 years, normal cognition and ability to use the remote rehabilitation app	Routine post-hospital follow-up guidance plus home rehabilitation prescription	Global motor/balance/ADL/gait and biomarkers: FMA, MAS, BBS, MBI, HADS, stride length/speed/frequency, BDNF, NT-3, NGF	Narrative/sensitivity only; randomization/allocation quality unclear and intervention combines remote guidance with wearable training
Wilson et al. 2021 [118] Australia	Home or nursing-home EDNA-22 training after acute inpatient/outpatient stroke rehabilitation	Stroke	Parallel RCT	Sample/analysis: 19 randomized; 17 completed training and post-test (EDNA n = 10; GRASP n = 7) Acute-care stroke patients with unilateral upper-extremity dysfunction, sitting balance and minimum active shoulder/elbow/wrist movement	Active GRASP home upper-limb training	Upper limb and cognition/function: Box and Blocks Test, Nine-Hole Peg Test, MoCA, SIS, Neurobehavioural Functioning Inventory	Active-comparator/sensitivity candidate; small RCT of home virtual rehabilitation versus evidence-based home arm programme
Wolf et al. 2015 [119] USA	Multisite home-based robotic telerehabilitation in underserved post-stroke populations	Stroke	Prospective multisite single-blind RCT	Sample/analysis: 99 randomized (HMP+HEP n = 51; HEP n = 48); 92 completed post-intervention Adults < 6 months after unilateral ischemic/hemorrhagic stroke with FMA-UE 11–55 and limited access to upper-extremity rehabilitation	Dose-matched home exercise programme only	Upper limb: Action Research Arm Test, Wolf Motor Function Test, Fugl-Meyer Assessment-UE	Primary RCT candidate/sensitivity; robotic telemonitored HEP versus dose-matched HEP
Wu et al. 2020 [120] China	Post-discharge home remote rehabilitation using collaborative-care model	Stroke	Two-arm single-blind RCT	Sample/analysis: 64 randomized; 61 completed (telerehabilitation n = 30; control n = 31) Adults aged 18–80 years with ischemic or hemorrhagic stroke, NIHSS 5–15, Brunnstrom stage II–III, and home discharge with caregiver capacity	Routine telephone follow-up and rehabilitation/nursing guidance after discharge	Motor/balance/mobility/ADL/QoL: FMA, BBS, TUG, 6MWT, MBI, Stroke-Specific Quality of Life	Primary RCT candidate; collaborative-care telerehabilitation exercise training versus telephone follow-up
Yokota et al. 2025 [121] Japan	National Cerebral and Cardiovascular Center with home-based IoT ergometer telerehabilitation after direct discharge home	Stroke/TIA	Single-centre retrospective observational study with propensity-score matching	Sample/analysis: 21 TR participants matched to 63 controls Functionally independent stroke or TIA survivors discharged directly home (mRS ≤ 1), able to pedal an ergometer and medically suitable for exercise training	No home-based telerehabilitation after discharge; usual discharge guidance/self-management advice	Exercise capacity/strength and HRQOL: 6MWD, isometric knee extension strength, handgrip strength, SF-36 component scores	Narrative/sensitivity only; non-randomized PSM comparison in high-functioning stroke/TIA survivors
Zheng et al. 2026 [122] USA	Remotely delivered home/community programme with in-person laboratory assessments	Multiple sclerosis	Phase-Ib assessor-blinded parallel RCT	Sample/analysis: 51 randomized (exercise n = 26; stretching control n = 25); 41 completed the 16-week conditions Older adults with MS (≥50 years) and moderate mobility disability, ambulatory with or without a cane, without severe cognitive impairment	Active stretching programme matched for contact, timeline and behavioural coaching	Physical/cognitive function and activity: SPPB, 30STS, TUG, T25FW, 6MWT, BICAMS, GLTEQ, accelerometry	Sensitivity/feasibility RCT; remotely delivered exercise versus active attention/stretching control

Abbreviations: ABC, Activities-Specific Balance Confidence Scale; ABC-SF, Activities-Specific Balance Confidence Scale-Short Form; ADL, activities of daily living; AIS, American Spinal Injury Association Impairment Scale; AMSQ, Arm Function in Multiple Sclerosis Questionnaire; ARAT, Action Research Arm Test; BBT, Box and Blocks Test; BDI, Beck Depression Inventory; BI, Barthel Index; BBS, Berg Balance Scale; CAHAI, Chedoke Arm and Hand Activity Inventory; COR, conventional outpatient rehabilitation; CR, conventional rehabilitation; CSI, Caregiver Strain Index; CST, corticospinal tract; DGI, Dynamic Gait Index; ECRL, extensor carpi radialis longus; EDSS, Expanded Disability Status Scale; ETNS, electromyography-triggered neuromuscular stimulation; FAC, Functional Ambulation Category; FGA, Functional Gait Assessment; FIM, Functional Independence Measure; FIS/FSS, Fatigue Impact Scale/Fatigue Severity Scale; FMA, Fugl-Meyer Assessment; FMA-UE/FMUE/UE-FM/UEFMA, Fugl-Meyer Assessment for the upper extremity; fMRI, functional magnetic resonance imaging; FONEFIM, Telephone Version of the Functional Independence Measure; GMV, grey matter volume; GRASP, Graded Repetitive Arm Supplementary Program/Glove Rehabilitation Application for Stroke Patients according to study context; HADS, Hospital Anxiety and Depression Scale; HAT, home automated tele-management; HBR, home-based rehabilitation; HEP, home exercise programme; HHD, hand-held dynamometry; HTR, home telesupervising rehabilitation; IQR, interquartile range; ISNCSCI, International Standards for Neurological Classification of Spinal Cord Injury; JAMAR, JAMAR Hand Dynamometer; JHFT, Jebsen Hand Function Test; K-ICARS, kinetic function subparameter of the International Cooperative Ataxia Rating Scale; LLFDI, Late-Life Function and Disability Instrument; MAL, Motor Activity Log; MAS, Modified Ashworth Scale; MBI, Modified Barthel Index; MDS-UPDRS, Movement Disorders Society-Unified Parkinson’s Disease Rating Scale; MFAC, Modified Functional Ambulatory Category; MFES, Modified Falls Efficacy Scale; m-MAS, modified-Modified Ashworth Scale; MMSE, Mini-Mental State Examination; mRS, modified Rankin Scale; MS, multiple sclerosis; MSQOL-54, Multiple Sclerosis Quality of Life-54; MSSE, Multiple Sclerosis Self-Efficacy Questionnaire; MSWS-12, 12-item Multiple Sclerosis Walking Scale; Muvity, telerehabilitation software platform; NBS, Navigated Brain Stimulation; NEADL, Nottingham Extended Activities of Daily Living; NIHSS, National Institutes of Health Stroke Scale; N-HPT/9-HPT, Nine-Hole Peg Test; OT, occupational therapy; PAS, Parkinson’s Activity Scale; PCS, Physical Component Summary; PD, Parkinson’s disease; PDDS, Patient Determined Disease Steps; PDQ-8/PDQ-39, Parkinson’s Disease Questionnaire; PT, physical therapy; QoL, quality of life; RCT, randomized controlled trial; RMS, root mean square; ROM, range of motion; rsFC, resting-state functional connectivity; SABI, severe acquired brain injury; SCI, spinal cord injury; SCIM, Spinal Cord Independence Measure; SEE, self-efficacy for exercise; SIBT, sensory integration balance training; SIS, Stroke Impact Scale; SPPB, Short Physical Performance Battery; T25FW, Timed 25-Foot Walk; 2MWD/2MWT, 2 min walking distance/walk test; 6MPT/6MWT, 6 min push/walk test; TA, tibialis anterior; TAI, Transfer Assessment Instrument; tDCS, transcranial direct current stimulation; TIS, Trunk Impairment Scale; TOCT-TR, task-oriented circuit training-based telerehabilitation; TR, telerehabilitation; TUG, Timed Up and Go; UE, upper extremity; UEMS/LEMS, upper/lower-extremity motor scores; UTRT, usual territorial rehabilitative treatment; VAS, Visual Analogue Scale; VERGE, Virtual Environment for Rehabilitative Gaming Exercises; VR, virtual reality; VRBT, Virtual Reality Balance Trainer; VRRS, Virtual Reality Rehabilitation System; V-TOCT, virtual reality-supported task-oriented circuit therapy; WISCI, Walking Index for Spinal Cord Injury; WMFT, Wolf Motor Function Test; WHOQOL-BREF, World Health Organization Quality of Life-BREF; APDDS, Adapted Patient Determined Disease Steps; BICAMS, Brief International Cognitive Assessment for MS; BDNF, brain-derived neurotrophic factor; CAS, Coronavirus Anxiety Scale; CIMT, Constraint-Induced Movement Therapy; EDNA, Elements system; FTHUE-HK, Functional Test for Upper Extremity-Hong Kong; HMP, Hand Mentor Pro; HRQOL, health-related quality of life; IoT, Internet of Things; MAL-AOU, Motor Activity Log-Amount of Use; MAL-QOM, Motor Activity Log-Quality of Movement; Mii-vitaliSe, MS Nintendo Wii intervention package; NIVRT, nonimmersive virtual reality training; NT-3, neurotrophin-3; PSM, propensity-score matching; SR, Smart Reminder; TIA, transient ischemic attack; V-ABC, Virtual Arm Boot Camp; ACTIV, Augmented Community Telerehabilitation Intervention; AROM, active range of motion; HCAD, Home Care Activity Desk; HAMD, Hamilton Depression Rating Scale; OCS, Oxford Cognitive Screen; PASS, Postural Assessment Scale for Stroke; PHQ-9, Patient Health Questionnaire-9; STREAM, Stroke Rehabilitation Assessment of Movement Scale; TENZR, wearable reach-to-grasp feedback device; WHODAS, World Health Organization Disability Assessment Schedule; A-VO2, arteriovenous oxygen difference.

**Table 2:** Intervention taxonomy: delivery model, dose, supervision and motor domain.

Study	Delivery Model	Modality	Technology	Intervention Content	Dose	Supervision/Caregiver Support	Motor Domain and Key Measures
García-Rudolph et al. 2024 [36]	Caregiver-supported home telerehabilitation	Synchronous plus asynchronous video-supported programme	Jitsi Meet/Zoom/Teams; therapist-recorded exercise videos; home rehabilitation equipment	TeleNeuroFitness group classes, TeleNeuroRehab one-to-one PT/OT sessions and TeleNeuroMov videos for SCI-specific exercises	3.5 h/day, 5 days/week, mean 67 days; daily dose included 60 min TNF, three 30 min TNR sessions and four 15 min TNM videos	Family/caregiver always present; daily nursing phone call; weekly physician call; bimonthly MDT review	Global motor-functional and mobility: FIM, SCIM III, WISCI II
Thielbar et al. 2020 [37]	VR/exergaming-based telerehabilitation	Crossover delivery-model comparison: multi-user vs. single-user VR	VERGE system; Kinect; Unity 3D; Google Cloud server; two-way voice communication	Home upper-limb VR games using Ball Bump, Trajectory Trace and Food Fight exercises	4-week crossover; 2 weeks MU and 2 weeks SU; four 1 h SU sessions/week and 8 scheduled MU sessions	Initial 2–3 day orientation and home installation; remote technical support; no continuous therapist supervision during sessions	Upper limb: arm displacement/session, session time, FMA-UE, compliance
Adams et al. 2023 [38]	VR/exergaming-based telerehabilitation	Asynchronous home VR plus biweekly synchronous OT telehealth	GRASP/SaeboVR; instrumented SaeboGlove; Kinect-based motion capture; web provider dashboard; videoconferencing/telephone	IADL-focused upper-limb practice with reaching, grasp-release, pick-and-place and graded virtual activities	45 min/session, 4×/week for 8 weeks; synchronous OT contact every other week	OT monitored dashboard metrics remotely and provided biweekly feedback, technology support and progression	Upper limb: FMUE, WMFT, BBT, MAL
Allegue et al. 2022 [39]	VR/exergaming-based telerehabilitation	Hybrid synchronous/asynchronous feasibility model	Jintronix exergames; Reacts videoconferencing app; computer, Kinect and USB Internet key	Upper-limb exergames plus supplementary fine-motor/UE exercises and motivational interviewing	Planned 30 min exergame sessions, 5×/week for 8 weeks (20 h target); videoconference 3×/week in weeks 1–2, 2×/week in weeks 3–4 and 1×/week in weeks 5–8; some participants extended to 3 months	Clinician adjusted game difficulty remotely, monitored logs and provided synchronous Reacts sessions	Upper limb: FMA-UE, MAL-30; SIS-16 hand function and mobility
Asano et al. 2021 [40]	Home-based tele-technology aided rehabilitation	Home telerehabilitation with usual rehabilitation permitted	STARS telerehabilitation system and standardized rehabilitation programme	Standardized PT/OT programme with therapist-tailored difficulty level and movement range	3-month programme; median reported telerehabilitation exposure 295 min in the intervention group	Assigned therapist tailored exercises; therapist/therapy-aide initial training; caregiver required for exercise safety	Global function and mobility: LLFDI disability, timed 5 m walk, 2MWD, modified BI, ABC scale
Aytutuldu et al. 2024 [41]	Synchronous therapist-led telerehabilitation	Delivery-model comparison; synchronous	Zoom videoconferencing	LSVT BIG amplitude-based exercises including maximal daily exercises, functional component tasks, hierarchical tasks and gait training	60 min/day, 4 days/week, 4 weeks	Real-time physiotherapist supervision during all online sessions	Balance/gait: Mini-BESTest, TUG, Kinovea gait parameters, Biodex postural stability, ABC-SF, PAS
Ballester et al. 2017 [42]	Home TR (VR/exergaming-based)	Asynchronous home-based VR with initial caregiver training	Rehabilitation Gaming System; Kinect; data gloves; Automated Evaluation of Motor Function	RGS Spheroids training with hit, grasp and place subtasks plus automated upper-limb motor assessment	20 min RGS training plus 2.5 min AEMF/day, 5 days/week for 3 weeks; 1–3 sessions/day permitted	System delivered to home; participant and caregiver trained; intervention performed without direct supervision	Upper limb: UE-FM, CAHAI, BI, MRC, grip force; NBS subgroup
Benvenuti et al. 2014 [43]	Home/community kiosk-based telerehabilitation	Hybrid home practice plus web-based kiosk telerehabilitation	Habilis web platform; touch-screen kiosks; webcams; videoconference	Task-oriented Carr and Shepherd Motor Learning Programme for reaching, grasping, holding and manipulation	3-month individualized programme; initial three 2 h training sessions; at least 2 kiosk sessions/week plus 3 home sessions/week	Hospital physiotherapist provided weekly videoconference review at kiosks; caregiver assistance if needed; monthly clinic review when kiosk not used	Upper limb and ADL: Motricity Index, WMFT, 9-HPT, Barthel Index, NEADL, SPPB, SIS
Calabrò et al. 2023 [44]	VRRS HomeKit teleneurorehabilitation	Synchronous therapist-led and caregiver-supported home VR rehabilitation	VRRS HomeKit tablet; K-wand/K-sensors; Tele-Cockpit remote workstation	Motor and cognitive VR training including eye-hand coordination, trunk control, bimanual coordination, attention, memory and executive-function tasks	1 h/day, 5 days/week for 12 weeks; before discharge, 3 instruction meetings and 6 simulation sessions were provided	Therapist supervised remotely through Tele-Cockpit; caregivers acted as co-therapists	Global motor-functional/balance: BI, Tinetti Scale, MAS; cognitive, QoL and caregiver outcomes
Carey et al. 2007 [45]	Home-based computerized telerehabilitation	Delivery-model comparison with crossover component	Laptop with custom tracking software; electrogoniometer braces; web camera; cellular phone/Internet link	Finger/wrist tracking with accuracy feedback versus dose-matched simple finger/wrist movement training	180 trials/day for 10 days (1800 trials over 2 weeks) per phase; move group crossed over to tracking	Therapist contacted participants about 5 times over 10 sessions by video/phone; pager support available	Upper limb: Box and Blocks Test, Jebsen–Taylor test, finger ROM, fMRI activation
Chae et al. 2020 [46]	Wearable/sensor-assisted telerehabilitation	Asynchronous remote monitoring plus weekly phone support	LG W270 smartwatch IMU; Android smartphone apps; CNN-based ML model; server; therapist app	Four bilateral upper-limb home exercises: shoulder flexion, wall push, active scapular exercise and towel slide	12-week home programme; education at enrolment; assessments at 0, 6, 12 and 18 weeks; exact prescribed weekly dose not specified	Weekly therapist calls; remote exercise-data review and encouragement based on HBR system records	Upper limb: WMFT, FMA-UE, grip power, shoulder ROM; BDI
Chantanachai et al. 2025 [47]	Synchronous therapist-led telerehabilitation with home neuromodulation	Adjunct active/sham tDCS plus identical tele-exercise	Video conference; Ybrain MINDD STIM home tDCS device	TDCS followed by arm stretching, upper/lower limb resisted exercise, functional balance and wheelchair/bed transfer training	12 sessions over 4 weeks; 3 sessions/week; each session included 20 min tDCS and 1 h tele-supervised exercise	Participants and caregivers trained for home tDCS; exercise supervised by physical therapist/researcher via video conference	SCI motor/sensory and function: ISNCSCI UEMS/LEMS/sensory, SCIM-III, TAI, H-reflex/m-MAS, HHD
Chen et al. 2017 [48]	Home-based live telesupervising rehabilitation plus ETNS	Synchronous home supervision versus in-person outpatient rehabilitation	TR network data system; live video–audio; remote control; MyoNet-COW ETNS; physiological monitoring	Bobath/PNF-based physical exercises, occupational-therapy tasks and EMG-triggered neuromuscular stimulation	Physical exercises 1 h twice per working day and ETNS 20 min twice per working day for 12 weeks; total 60 sessions	Therapists supervised via live video; caregivers were trained and kept training logs; physiological/EMG data fed back to therapist end	ADL/balance and neuromuscular function: MBI, BBS, mRS, RMS of ECRL/TA, CSI
Chen et al. 2020 [49]	Home-based motor telerehabilitation plus ETNS	Synchronous live-video home telerehabilitation	Telemedicine Rehabilitation System with therapist end, network data system and patient end; ETNS	Home OT/PT and EMG-triggered neuromuscular stimulation for motor training	12 weeks; target 10 sessions/week; each session included 60 min OT/PT and 20 min ETNS	Therapists supervised OT/PT and ETNS via live video; patients/caregivers recorded delivered dose	Global motor and ADL: FMA, MBI; M1-M1 rsFC, M1 GMV and CST integrity
Chen et al. 2021 [50]	VR/exergaming-based telerehabilitation	Simulated-home interactive telerehabilitation with active comparator	Kinect camera-based LongGood exergaming system; wireless sensor network; cloud database	Target-oriented stepping, multidirectional reaching and Tai Chi exercises for balance, strength, weight shifting and walking	12 sessions; 40 min/session, 3×/week for 4 weeks; each session included warm-up, 3 game tasks and cool-down	Remote therapist monitoring/review; family or caregiver stood by to prevent falls	Balance/mobility: BBS, TUG, MFES, Motricity Index, FAC
Chumbler et al. 2012 [51]	Remote exercise/tele-coaching	Multifaceted telephone, televisit and in-home messaging intervention	In-home messaging device; telephone calls; camcorder-supported home televisits	Functionally based strength/balance exercises, adaptive strategies and home-environment recommendations	3-month intervention with three 1 h televisits, daily IHMD use and five telephone calls	Teletherapist monitored IHMD weekly and advanced the programme by phone; trained assistant supported home televisits	Global motor-functional/disability: Motor FONEFIM, LLFDI function and disability domains
Chung et al. 2020 [52]	Mobile video-guided home exercise delivery	Asynchronous digital home exercise compared with paper handout	Smartphone/tablet QR-code exercise videos	Home programme including arm control, leg control, trunk control and mobility exercises	3–5 exercises prescribed; daily to 3×/day; 10–30 min/day for 3 months	Pre-discharge 10–15 min training; phone follow-up assessments; no remote therapist progression reported	Mobility/ADL and adherence: MFAC gain, MBI gain, exercise adherence, SEE
Cikajlo et al. 2012 [53]	VR-supported balance telerehabilitation	Hybrid clinic-to-smart-home model with synchronous remote supervision	Balance Trainer standing frame with tilt sensor; VRML environment in Internet Explorer; web browser and videoconference	Dynamic standing-frame balance training using weight shifting to navigate a virtual path and avoid virtual obstacles	Up to 20 min/session, 5 days/week for about 3 weeks; approximately 2 weeks in clinic plus 1 week in smart-home/home setting	Physiotherapist supervised progress remotely through web browser and videoconference and advised on posture/hand placement	Balance-related activity/postural control: BBS, TUG, 10 m walk test, single-limb stance and VR task time/collisions
Conroy et al. 2018 [54]	Internet-based home automated tele-management	Asynchronous web-supported home exercise	MS HAT platform with patient unit, server and clinical unit; written/pictorial/video exercises, diary, messages, calendar and feedback graphs	Individualized core and lower-extremity home exercise programme targeting gait, strength, flexibility and functional activities	Daily home exercise prescribed over 6 months; repetitions, sets and progression individualized by therapist	Baseline PT instruction; HAT participants had asynchronous text messaging and exercise updates; no live online exercise supervision	Walking/mobility activities: T25FW, 6MWT, BBS and MSWS-12
Coulter et al. 2017 [55]	Web-based physiotherapy with remote progression	Asynchronous web platform with scheduled remote review	www.webbasedphysio.com; exercise videos, written explanations, audio descriptions and online diary	Individualized aerobic, strengthening, stretching and balance exercises adapted for people with SCI	Approximately 30 min/session, at least 2 sessions/week for 8 weeks	Physiotherapist reviewed online diaries and contacted participants by email or phone every 2 weeks to progress exercises	Walking/mobility activities or wheelchair mobility: 6MWT or 6MPT; strength, HADS and WHOQOL-BREF
Cramer et al. 2019 [56]	Home-based therapist-supported upper-limb telerehabilitation	Hybrid supervised and unsupervised delivery-model comparison	Internet-enabled computer, table/chair, videoconferencing software and 12 gaming input devices	Activity-based arm training with exercises, functional games matched to clinic-based functional tasks and stroke education	36 sessions of 70 min over 6–8 weeks; 18 supervised and 18 unsupervised sessions	Supervised sessions began with 30 min therapist videoconference; therapists reviewed electronic data and adjusted treatment plans	Upper limb: FM arm motor score, Box and Blocks Test, SIS hand domain and stroke knowledge
Dastan et al. 2025 [57]	Synchronous therapist-led telerehabilitation	Delivery-model comparison: synchronous versus asynchronous telerehabilitation	Skype videoconferencing for synchronized sessions; instructional exercise videos for asynchronous delivery	Upper-extremity exercise programme with warm-up, elastic-band exercises, loading/play-dough tasks, fine-motor activities and stretching cool-down	2 sessions/week for 8 weeks; 40–60 min/session; progression from 2 sets of 8 repetitions to 3 sets of 10 repetitions, updated every 2 weeks	Real-time physiotherapist supervision in the synchronized group; asynchronous group received videos and meetings every 2 weeks	Upper limb: N-HPT, JAMAR grip strength and AMSQ; fatigue, QoL and participation outcomes
Van den Berg et al. 2016 [58]	Caregiver-supported hybrid telerehabilitation	Hybrid in-hospital caregiver-mediated training plus home e-health/video support	Customized iPad exercise app with 37 standardized exercises; Vidyo secure videoconferencing; Fitbit Zip activity monitor	Caregiver-mediated gait and gait-related mobility exercises including standing, turning, transfers and walking tasks	8 weeks; at least 5 sessions/week, 30 min/session; continued at home when discharge occurred before programme completion	Caregiver delivered exercises; weekly physiotherapist evaluation; home visits and videoconferencing support after discharge	Walking/mobility activities and ADL: SIS mobility, TUG, Rivermead Mobility Index, Barthel Index, NEADL, Fugl-Meyer lower extremity, BBS
Deng et al. 2012 [59]	Home computerized ankle telerehabilitation	Delivery-model comparison: complex tracking versus simple movement	Laptop with customized ankle training software; electrogoniometer; webcam; LogMeIn; Skype; cellular modem; automated email of performance files	Complex paretic ankle dorsiflexion/plantarflexion tracking with accuracy feedback versus simple ankle movement practice	60 blocks/day, 3 trials/block, 180 trials/day for 20 days; total 3600 trials over 4 weeks	Therapist contacted participants twice weekly by telecommunication and monitored automatically emailed daily performance records	Walking/mobility activities: paretic ankle dorsiflexion during swing, 10 m walk test, gait kinematics and fMRI activation
Van der Kolk et al. 2019 [60]	Home-based remotely supervised aerobic exergaming	Asynchronous home exercise with remote coaching and motivational app	Stationary home-trainer with VR/real-life video exergaming software; tablet motivational app; heart-rate monitor; secure website	High-intensity aerobic cycling in a target heart-rate zone versus stretching/flexibility/relaxation active control	30–45 min/session, 3 sessions/week for 6 months; target intensity progressed within approximately 50–80% heart-rate reserve	One home visit and remote telephone coaching every 2 weeks; coaches tracked exercise data online	Disease-specific motor symptoms and mobility: MDS-UPDRS III OFF, 6MWT, Mini-BESTest and TUG
Dogan et al. 2023 [61]	Asynchronous app-based telerehabilitation	Delivery-model comparison: home app telerehabilitation versus clinic-based V-TOCT	Fizyo mobile app with exercise videos and patient feedback; weekly online meetings; inertial sensors used for outcome kinematics	Home balance, strengthening, coordination and stretching exercises with patient-specific progression	60 min/session, 3 sessions/week for 8 weeks	Participants reported session difficulty through the app; programme updated through weekly online meetings with physiotherapist	Trunk and upper limb: TIS, K-ICARS, ABILHAND, MMDT and inertial-sensor kinematics
Eldemir et al. 2023 [62]	Synchronous task-oriented circuit training telerehabilitation	Synchronous videoconference telerehabilitation plus home exercise	Videoconferencing; home exercise booklet/diary for balance, walking and mobility activities exercises	Fifteen upper-limb workstations using ADL-related tasks such as writing, eating, grasping, reaching, buttoning, dressing and grip-strength exercise	TOCT-TR 60 min/session, 3 sessions/week for 6 weeks; each task 3 min with 1 min rest intervals; both groups also performed home exercises 3x/week	Physiotherapist supervised TOCT-TR via videoconference; participants received telephone contact twice weekly for home exercise queries	Upper limb/disease-specific motor: 9-HPT, JHFT, grip and pinch strength, UPDRS-III; UPDRS-II and PDQ-8
Eldemir et al. 2024 [63]	Synchronous Pilates-based telerehabilitation	Synchronous videoconference telerehabilitation versus waitlist	Zoom or WhatsApp videoconferencing; resistance bands, balls and home exercise equipment	Clinical Pilates in supine, side-lying, quadruped, sitting and standing positions with warm-up, strengthening/core stability and cool-down	60 min/session, 3 sessions/week for 6 weeks; 10 repetitions in weeks 1–3 and 20 repetitions in weeks 4–6	Certified Pilates physiotherapist supervised all sessions remotely; baseline in-clinic education session before home programme	Balance/gait/global physical performance: BBS, Biodex balance measures, G-Walk gait analysis, 6MWT, strength, core endurance/power, fatigue and MSQOL-54
Fjeldstad-Pardo et al. 2018 [64]	Synchronous therapist-led telerehabilitation	Three-arm comparison: remote PT, unsupervised HEP and in-person PT	Audio/visual real-time telecommunication; NeuroCom Smart Balance Master used for outcome testing	Customized physical therapy focused on gait and balance deficits	8 weeks; TR group received remote PT supervision twice weekly; HEP control trained 5 days/week; PT arm attended in-person PT twice weekly	Physical therapist supervised TR sessions in real time through audio/visual communication	Gait/balance: FGA, T25FW, BBS, NeuroCom walk/tandem/limits-of-stability/sensory organization metrics and ABC
Fluet et al. 2024 [65]	Sparsely supervised home VR/game-based rehabilitation	Delivery-model/game-design comparison: scaffolding versus algorithm-controlled difficulty	HoVRS system; Leap Motion Controller; Unity 3D rehabilitation games; cloud-based data pipeline	Two to five individualized games targeting shoulder/elbow, forearm, wrist and finger movements; progressive scaffolding or adaptive algorithm control	12-week home programme; participants were encouraged to train at least 20 min/day but could train as much as desired	Initial home setup and training by PT/technologist; online or in-person support and periodic updating of game routines as needed	Upper limb/adherence: UEFMA, ARAT, SIS hand/ADL/participation, IMI and system-recorded training time
Flynn et al. 2021 [66]	Hybrid in-person plus remotely monitored home exercise	Delivery-model feasibility comparison after common centre-based block	PhysioTherapy eXercises website/app or paper format; telephone monitoring	Individualized Parkinson-specific balance and gait exercises, everyday mobility tasks, dual-task/cueing exercises and self-management training	10-week programme. Weeks 1–5: all participants 2 centre sessions/week plus 1 home session/week. Weeks 6–10: home group 3 home sessions/week, 45–60 min; phone monitoring in weeks 7 and 9	Physiotherapist monitored/progressed home programme by telephone; centre-based arm had supervised group exercise	Balance/gait feasibility: Mini-BESTest, 10 m walk test and New Freezing of Gait Questionnaire
Frevel et al. 2015 [67]	Asynchronous Internet-based home training	Web-supported home exercise with therapist feedback versus hippotherapy	MotionNET e-Training platform with individualized training plan, written/pictorial exercise instructions and Borg-scale feedback	Balance, postural-control and strength exercises using body weight, mats, elastic bands, gym balls, unstable surfaces, reduced base of support and visual-control manipulation	45 min/session, 2 sessions/week for 12 weeks; 5–8 exercises/session, Borg 11–14, 8–15 repetitions and 2–3 sets	Exercise therapist reviewed feedback online and adjusted the programme; participants received an initial supervised instruction meeting	Balance/mobility: BBS, DGI, 2MWT, isometric strength, fatigue and QoL
Gandolfi et al. 2017 [68]	Caregiver-supported in-home VR telerehabilitation	Synchronous delivery-model comparison: home TeleWii versus in-clinic SIBT	Nintendo Wii Fit console and balance board; laptop; high-resolution webcam; Skype	Ten graded TeleWii exergames targeting postural stability, weight shifting, ankle/hip/stepping strategies, dual-tasking and gait-related balance	21 sessions, 50 min/session, 3 days/week for 7 consecutive weeks	Physiotherapist supervised each full session by Skype, often two patients simultaneously; caregiver was always present for safety	Balance/gait: BBS, DGI, 10-MWT gait speed, ABC, PDQ-8 and falls
Garcia et al. 2022 [69]	Nonimmersive VR telerehabilitation	Asynchronous home-based crossover telerehabilitation	Muvity application; Intel RealSense D415 depth camera; NuiTrack SDK; household computer; external data server	Six ADL-like games and five exercises training shoulder flexion/extension, shoulder abduction/adduction, elbow flexion/extension and mediolateral pelvis translation	30 min/session, 3 days/week for 8 weeks per treatment phase; two 8-week phases separated by 2-week washout	Physiotherapist performed initial home setup and instruction; server-side data allowed asynchronous progress review, with no continuous supervision reported	ROM/global function: shoulder/elbow/pelvis ROM, FIM, Berg Balance Scale, VAS pain, SF-36 PCS and usability
Ge et al. 2024 [70]	Asynchronous app-based home telerehabilitation	Active-comparator delivery model; asynchronous TR versus supervised home physical therapy	Dedicated mobile app with patient information, training plans, instructions, tips, exercise-demonstration videos and adverse-event reporting	Home balance, resistance/strength, gait/LSVT BIG-style, stretching and aerobic exercises selected and progressed by the physiatrist; TR participants trained using the mobile app	40–60 min/session, 5 days/week for 4 consecutive weeks; prescription progressed from 200 min/week in weeks 1–2 to 270 min/week in weeks 3–4	Physiatrist adjusted the programme weekly; therapists sent reminders and could be contacted; caregiver supervision was recommended to prevent adverse events	Disease-specific motor, balance, gait and ADL: UPDRS III, BBS, TUG, FTSST, FOGQ, IDEEA gait parameters, isokinetic strength, UPDRS II and PDQ-39
Ginis et al. 2016 [71]	Wearable/sensor-assisted smartphone telerehabilitation	Home-based automated feedback gait training with active advice comparator	CuPiD system: Samsung Galaxy S3-mini smartphone, docking station, two EXLs3 inertial measurement units, ABF-gait app and FOG-cue app	Auditory biofeedback on cadence, stride length, symmetry or gait speed; participants with freezing of gait also practiced turning, cluttered-space walking and figure-of-eight tasks with intelligent cueing	At least 30 min of walking, 3×/week for 6 weeks; participants with freezing of gait performed an additional 30 min, 3×/week using the FOG-cue app	Weekly home visits by the researcher, telephone support, individualized calibration and adjustment of gait/FOG parameters during the 6-week programme	Walking/mobility activities and balance: usual and dual-task gait speed, 2MWT, Mini-BESTest, FSST, FOGQ, ABC and SF-36 physical health
Goffredo et al. 2023 [72]	Nonimmersive VR-based telerehabilitation	Asynchronous home VR training with weekly synchronous physiotherapist session	VRRS Tablet system (Khymeia) with inertial sensors, serious-game environment, visual and auditory feedback, tablet-based daily reports	Balance and lower-limb motor-performance exercises, including one-leg stance, marching in place, standing on tiptoe and squatting, customized by therapists and progressed through software parameters	30 sessions of approximately 45 min; 3–5 sessions/week over 6–10 weeks; same frequency and duration as control booklet exercises	Weekly synchronous physiotherapist session plus asynchronous monitoring of tablet reports; control adherence monitored by paper diary with caregiver help when needed	Balance/postural stability and mobility: Mini-BESTest, TUG, 6MWT and MDS-UPDRS Part III
Guo et al. 2023 [73]	Wearable/sensor-assisted remote rehabilitation system	Autonomous human–computer-interaction training with remote cloud data upload in rehabilitation halls	Wearable IMU modules for upper/lower limbs, rehabilitation glove with IMU and five flex sensors, ZigBee receiver, PC rehabilitation games/software, cloud management platform and mobile app	Interactive upper-limb, hand and lower-limb exercises including Bobath-based upper-limb movements, wrist/finger/ball-grip tasks and squat/knee movement training, with game-based visual/auditory feedback	3 weeks; system-guided training or routine OT 30 min twice/day plus conventional PT 30 min twice/day; at least 10 sessions/week and at least 30 sessions total	Training was performed without therapist participation except for safety; uploaded data allowed physicians to review scores and update exercise prescriptions remotely	Global motor function: Fugl-Meyer Assessment total, upper-extremity and lower-extremity scores
Hartung et al. 2025 [74]	Internet-based exercise and physical-activity promotion	Hybrid asynchronous app/e-learning plus telephone/video coaching	MS bewegt platform using Microsoft Azure/proMX-motionNET therapist control centre, smartphone/web app, ILIAS e-learning modules and consumer PA monitor (Fitbit Inspire or Garmin vivofit 4)	Tailored home endurance and resistance exercise prescription combined with e-learning on technology, MS symptoms, motivation/volition and PA planning/monitoring	12-week intervention; endurance 1–2×/week for 10–60 min at RPE 11–15; resistance 1–2×/week with 6–8 exercises, 8–20 repetitions and 2–3 sets; followed by 12-week usual-care follow-up	One-to-one therapist coaching by two calls in weeks 1 and 7 and group video calls in weeks 4 and 11; app chat, diary and progress monitoring; dose progressed semi-automatically using RPE	Physical activity and mobility: steps/day, MVPA, sport/exercise, T25FW, 2MWT, MSWS-12, MSIS-29, fatigue and PAHCO outcomes
Hernandez et al. 2022 [75]	VR/exergaming-based home telerehabilitation	Asynchronous remote therapist monitoring and adjustment	Jintronix software, computer, large screen, Microsoft Kinect depth camera and web-based performance server	Interactive serious games for repeated unilateral and bilateral upper-extremity reaching, grasping, carrying, dropping and target-directed movements	Recommended ≥ 20 min/session, 5×/week for 4 weeks; programme customized to baseline UE function	Physiotherapist provided initial tutorial; technician installed the home system; therapist monitored remotely once or twice weekly and adjusted difficulty, speed and trajectories asynchronously	Upper limb: FMA-UE primary outcome; SIS domains and MAL-14; feasibility by sessions, active playing time, pain/fatigue, dizziness and falls
Hong et al. 2025 [76]	Synchronous therapist-led telerehabilitation	Real-time video task-oriented upper-limb training with asynchronous quality-control logs	WeChat videoconferencing, dual camera angles (wide and close-up), household objects, daily video logs and therapist audit procedures	Task-oriented upper-limb practice using household activities such as cup grasping, drawer pushing and turning keys; task complexity progressed weekly according to performance	60 min/session, 6 sessions/week for 3 weeks (18 h total)	Real-time therapist correction, error screening every 15 min, daily video-log review, caregiver supervision in tele-control arm and in-person reassessment if deviations exceeded 30%	Upper limb and ADL: FMA-UE, WMFT-FAS, ARAT and IADL
Huijgen et al. 2008 [77]	Home-based HCAD telerehabilitation	Daily asynchronous sensorized practice plus weekly synchronous videoconference	Home Care Activity Desk portable unit with seven sensorized tools (key, light bulb, book, jar, writing, checkers and keyboard), two webcams and hospital server	Functional upper-limb activities targeting reaching, grasping, lateral pinch, pinch grip, holding, manipulation and finger dexterity	After one month of usual care and approximately four hospital training sessions, the home HCAD phase lasted one month with at least one 30 min session/day, 5 days/week	Exercise videos and results were uploaded to the hospital server; therapist used the information during weekly videoconferences; control received usual care and generic exercises	Upper limb/hand function: ARAT and Nine-Hole Peg Test; user satisfaction with HCAD
Jarbandhan et al. 2022 [78]	Hybrid home-based physiotherapy with telephone tele-coaching	In-person home supervision followed by tele-supervised self-management	Garmin Forerunner 225 heart-rate monitor, Yamax Digiwalker SW-200 pedometer, Borg scale, blood-pressure monitoring, exercise diary and weekly telephone contact	Holistic lower-limb strengthening/endurance, stair climbing, sit-to-stand, walking on varied surfaces, upper-limb PNF/mobility exercises and patient/family education	8 weeks, 3 days/week; first 4 weeks supervised at home for 70 min/session, second 4 weeks continued independently with weekly telephone encouragement and instructions	Physiotherapist supervised the first phase, reassessed progress at 4 weeks, adapted intensity and provided weekly tele-coaching; caregiver support was permitted when needed	Mobility/function: 6MWT, BBS, DASH, paretic/non-paretic handgrip strength, exercise self-efficacy, adherence, satisfaction and safety
Johnson et al. 2020 [79]	VR/exergaming-based telerehabilitation	Community-based virtual therapy with remote monitoring versus usual care	Jintronix Rehabilitation System with Microsoft Xbox Kinect camera, standard laptop and television display	Tailored upper-limb virtual-therapy games/exercises targeting shoulder, elbow and wrist range of motion, motor control, strength and dexterity	Approximately 45 min/session, 2 sessions/week for 8 weeks	Onsite exercise physiologist provided technical/safety support only; remote exercise physiologist reviewed clinician-interface data weekly and progressed exercises when performance targets were met	Upper limb: FMUE, ARAT, BBT, MAS, MAL-28 and EQ-5D-5L
Johnson et al. 2024 [80]	Web/mobile telerehabilitation platform for Parkinson’s disease	TR-only and clinic-plus-TR hybrid delivery compared with usual care	WizeCare Technologies web platform/mobile app, video conferencing, pre-recorded PD-specific exercise-video library, tablet/computer/phone access	Customized therapist-prescribed PD-specific home exercise programmes, with live video visits in the TR-only arm and platform-based home exercise in TR-only and clinic-plus-TR arms	4-week intervention; weekly 1 h sessions (virtual for TR-only, in-clinic for clinic-plus-TR and usual care) plus daily home exercise; usual care used paper home exercise handouts	Therapists individualized and adjusted exercises through the platform; patients completed automated safety checks after sessions; one unrelated injurious fall led to withdrawal	Feasibility and motor/mobility: TUG, TUG-cognitive, 5STS, 10MWT, 6MWT, Mini-BESTest, PDQ-39 and ABC
Kintrilis et al. 2024 [81]	Synchronous therapist-led telerehabilitation	Delivery-model comparison, synchronous	Skype v8 online telecommunication platform; elastic resistance bands	Strength/resistance training with elastic bands delivered either face to face or via teleconference	30 min/session, 3 sessions/week for 12 weeks	Therapist-led real-time sessions; relatives/caregivers assisted participants with online connection/setup when needed	Walking/mobility activities and functional capacity: TUG, BBS, Chair Stand Test, 6MWT, VAS health score and A-VO2
Kowalczewski et al. 2011 [82]	Supervised home-based telerehabilitation	Randomized crossover delivery-model comparison	Laptop, webcam, Internet connection, VNC, Skype, ReJoyce workstation, FES garment and tooth-click wireless trigger	ReJoyce FES-assisted workstation training with ADL-like manipulanda and computer games; comparator conventional teletherapy used strength training, trackball games and therapeutic electrical stimulation	Each treatment 1 h/day, 5 days/week for 6 weeks, separated by a 1-month washout	Tele-supervisors remotely supervised sessions from the host laboratory or home; physical therapist prescribed individualized exercises and trained supervisors	Upper-limb/hand function: ARAT primary outcome, grasp/pinch forces and ReJoyce Automated Hand Function Test
Lee et al. 2022 [83]	Synchronous dance-therapy telerehabilitation	Real-time videoconference adjunct to conventional physical therapy	Zoom desktop videoconferencing, desktops/TV monitors, video cameras and adapted dance for PD-based stroke dance protocol	Sitting warm-up, chair/standing choreography and dance-skill practice reconstructed to target trunk control, gait and balance in hemiplegic stroke	40 min/session, 2 sessions/week for 3 weeks, in addition to conventional physical therapy	Experienced dance instructor guided sessions remotely with two-way audio-visual feedback; caregiver participated for safety and researcher monitored for falls outside the room	Trunk control and balance: TIS primary outcome; BBS, TUG, FAC, K-MBI and EQ-5D
Lee et al. 2025 [84]	Synchronous inpatient telerehabilitation	Real-time tele-occupational therapy plus regular rehabilitation	Google Meet, ASUS notebook on rolling laptop table, therapy objects (resistance bands, bottles, coins), telerehabilitation manual and LINE links/messages	Upper-limb, lower-limb and balance training at three graded levels, plus education, ADL-related strategies, assistive-device use, mental-health support and discharge preparation	10 sessions, 30 min/session as additional ward therapy; pre-, mid- and post-evaluations after sessions 0, 5 and 10	Research assistant set up equipment and trained patients/caregivers; tele-OT set goals, demonstrated tasks, provided feedback, shared videos via LINE and adjusted plans session by session	ADL/mobility/postural control: MBI, PASS, FAC, Borg RPE, PHQ-9 and satisfaction
Lin et al. 2014 [85]	Bidirectional multi-user telerehabilitation in long-term care facilities	Synchronous small-group balance telerehabilitation with remote vital-sign monitoring	WSN system with therapist-end notebook/webcam/VoIP, client-end PC, standard screen, touch-screen, webcam, Adobe Media Server, ZigBee pulse oximeter and blood-pressure sensors	Standing balance training progressing body position, environment and upper-limb manipulation; 3D animation exercise videos and 3D interactive touch-screen games in standing posture	Approximately 50 min/session, 3 sessions/week for 4 weeks; telerehabilitation focused on about 10 min of standing exercise videos and about 10 min of interactive games within each session	Therapist supervised two participants remotely at the same time, monitored HR/SpO2/BP and Borg 12–14 intensity; volunteer/nonmedical assistant ensured safety at the client end	Balance/function: Berg Balance Scale, Barthel Index total/self-care/mobility and satisfaction
Llorens et al. 2015 [86]	Home-based VR telerehabilitation	Delivery-model comparison: home versus in-clinic use of the same VR balance system	Kinect motion sensor, laptop/television at home, personal computer/screen in clinic and adaptive VR stepping environment	Balance training requiring participants to step on virtual floor targets with the nearest foot while maintaining the other foot in the central circle; difficulty adapted by item location, distance, size, lifetime and number	Twenty 45 min sessions, 3×/week over 8 weeks; each session included six 6 min repetitions with 90 s breaks; conventional clinic PT continued on non-VR days	Blinded physical therapist adjusted and remotely reviewed weekly progress; technical support included weekly brief interviews with home participants	Balance and locomotor skills: BBS, POMA-B, POMA-G, BBA, usability, motivation and cost
Masbernat-Almenara et al. 2026 [87]	Home-based core-stability telerehabilitation	Delivery-format comparison: mixed synchronous/asynchronous versus asynchronous only	Cloud-based exercise-video library, web platform with personal diary, email video links and videoconference calls	Individualized core-stability exercise programme with predefined regressions/progressions prioritizing neutral spine, pain ≤ 3/10, safety and movement quality	7 weeks, 5 days/week, 30–40 min/session; mixed group: one weekly 45 min live PT session plus 4 asynchronous sessions; asynchronous group: 5 independent sessions/week plus weekly follow-up call	Initial in-person physiotherapist visit; weekly synchronous session or monitoring call; repetition/exercise adjustments based on symptoms, technique, fatigue and safety	Gait, trunk balance and ataxia: 4MWT, TUG, 30 s sit-to-stand, S-TIS 2.0, SARA, ABC and adherence
Mulder et al. 2024 [88]	Caregiver-mediated blended telerehabilitation	Hybrid in-person support plus asynchronous telerehabilitation platform	Telerevalidatie.nl platform with exercise videos, personal instructions, mobility milestones, messaging environment and training diary	Progressive task-specific caregiver-mediated mobility exercises focused on balance, transfers, walking and stair-related mobility, added to usual care	8-week programme; minimum 5 × 30 min/week caregiver-mediated exercises; at least four in-person sessions scheduled every other week plus asynchronous platform support	Informal caregiver delivered exercises; physiotherapist tailored the programme, trained dyads, reviewed fidelity/barriers and modified goals; weekly platform use and falls recorded	Mobility/function: SIS mobility primary outcome; RMI, FAC, 6MWD, 5 m walking speed, Motricity Index leg score, BBS, NEADL and psychosocial/transition outcomes
Nuic et al. 2024 [89]	Home-based tailored exergaming	Active full-body VR/exergaming versus seated keyboard-control gaming	Toap Run exergame, Microsoft Kinect v2 RGB-D motion sensor, television screen, Curapy web platform and automated performance recording	Full-body large-amplitude rapid movements of limbs, pelvis and trunk with lateral, vertical and forward leg displacements, rhythmic music, visual/auditory cues and feedback; control used the same game via keyboard without physical effort	18 sessions over 6–9 weeks, 2–3 sessions/week; first two sessions at the institute, next two at home with a research assistant, then independent home sessions	Game difficulty progressed across three phases and could be individually tailored from automated session data; participants could contact investigators by telephone if needed	Gait/balance: Stand–Walk–Sit Test primary outcome; MDS-UPDRS axial/motor outcomes, GABS-B, Tinetti, NFOG-Q, ABC, gait recordings, falls and safety
Ortiz-Gutiérrez et al. 2013 [90]	VR/exergaming-based telerehabilitation	Synchronous real-time videoconference monitoring versus conventional physiotherapy	Xbox 360 with Kinect; home television; online videoconferencing; Smart Equitest computerized dynamic posturography for assessment	Commercial Kinect Sports, Kinect Joy Ride and Kinect Adventures tasks selected to challenge sensory integration, weight shifting, multidirectional movement, postural reactions and hand/foot–eye coordination	40 sessions over 10 weeks; 4 sessions/week; up to 20 min/session; control received conventional physiotherapy 40 min twice/week for 10 weeks	Physiotherapist monitored and supervised all home gaming sessions in real time by videoconference; patients advised to perform sessions with another person at home for safety	Postural control/balance: SOT composite equilibrium and sensory ratios
Pagliari et al. 2024 [91]	VRRS home-based telerehabilitation	Asynchronous home VRRS with offline remote monitoring versus active home usual care	VRRS home-based kit (Khymeia); digital motor and cognitive rehabilitation contents; remote therapist monitoring	Integrated dual-domain motor and cognitive VR rehabilitation activities tailored to pwMS; comparator used conventional motor/cognitive exercises in booklet form	30 sessions over 6 weeks; 5 sessions/week; 45 min/session in both groups	Therapist remotely monitored performance offline in the TR group; usual-care group self-administered tailored booklet exercises	Balance/motor/participation: MSQOL-54, Mini-BESTest, BBT, 9-HPT and adherence
Paul et al. 2014 [92]	Web-based physiotherapy with remote progression	Asynchronous web programme plus weekly telephone review	www.webbasedphysio.com with videos, text, audio descriptions, timer, exercise diary and advice pages	Individualized cardiovascular, strengthening and balance exercises, plus warm-up, cool-down and stretching at multiple difficulty levels	12 weeks; participants advised to complete the programme at least twice/week; exercises progressed remotely as required	Physiotherapist monitored online diaries weekly, telephoned participants weekly and remotely changed exercises/difficulty/repetitions	Gait/balance and MS impact: T25FW, BBS, TUG, MSIS, QoL and HADS
Paul et al. 2019 [93]	Web-based individualized home physiotherapy	Asynchronous web-based physiotherapy versus printed exercise-sheet active comparator	www.webbasedphysio.com; exercise videos, audio/text descriptions, online diary and email notification; comparator used www.physiotherapyexercises.com printed sheets	Individualized cardiovascular, strengthening, balance, warm-up, cool-down and stretching programme prescribed from standardized physiotherapy assessment and goals	6 months; both groups asked to exercise twice/week (52 expected diary entries)	Physiotherapist reviewed electronic diaries every 2 weeks and remotely altered web programmes; weekly phone/email support for both groups during first 2 weeks	Mobility/balance/physical activity: 2MWT, T25FW, TUG, BBS, steps/day and MSIS
Pavan et al. 2024 [94]	Home robotic or non-robotic telerehabilitation	Hybrid synchronous/asynchronous delivery-model comparison	Telemedicine platform; ICONE end-effector robot for the robotic group; digital exercise booklet/notebook for the non-robotic group	Upper-limb reaching, grasping, lifting and ADL-simulation exercises; RG used planar reaching exergames with assistive/resistive/adaptive robot modes, NRG used digitally supported non-robotic exercises	20 sessions over 5 weeks; 4 days/week; 45 min/session; 1 synchronous therapist session and 3 asynchronous autonomous sessions/week	Weekly synchronous therapist session assessed progress, checked performance and planned following week; robot logs or patient notebook supported asynchronous monitoring	Upper limb/global disability: WHODAS 2.0, FMA-UE, BBT and clinical-functional status
Petracca et al. 2024 [95]	Synchronous therapist-led telerehabilitation	Real-time one-to-one videoconference versus onsite one-to-one rehabilitation	Xbox One with Kinect v2, Skype for Kinect and preconfigured online account; onsite programme used comparable equipment as needed	Individualized aerobic/anaerobic exercise, balance, core stability, flexibility, mobility, strengthening, gait, task-oriented training and PNF-based techniques	18 sessions over 6 weeks; 3 sessions/week; 45 min/session in both TR and onsite rehabilitation	One-to-one physiotherapist supervision in real time for TR and in-person for onsite rehabilitation; participants trained for console assembly/use at baseline	QoL/fatigue/balance/cognition: MSQoL-54, FSS, BBS and SDMT
Picelli et al. 2026 [96]	Synchronous multidomain VRRS telerehabilitation	Real-time therapist-supervised telerehabilitation versus in-person outpatient multidisciplinary therapy	VRRS system with K-Wand and Khymu sensors; bidirectional telehealth platform, video and motion tracking	Integrated motor, cognitive and speech-language therapy; motor training used virtual trajectories; cognitive/language modules targeted attention, executive function and aphasia domains	20 sessions over 4 weeks; 1 h/day; 5 days/week in both groups	Therapists supervised, adapted and monitored exercises in real time; adherence/fidelity tracked with therapist logs and VRRS platform logs	Motor/cognitive/language/disability: FMA-UE, FMA-LE, IT-AAT, OCS and BI
Piron et al. 2009 [97]	VR-based home telerehabilitation	Synchronous therapist-supervised VR teletherapy	VRRS.net; two PC workstations; broadband Internet/ADSL; integrated videoconference; 3D Polhemus motion tracking	Five virtual upper-limb reaching/movement tasks with virtual teacher trajectory feedback and therapist feedback via videoconference	1 h/day, 5 days/week for 4 weeks in both groups	Therapist remotely viewed the virtual task and patient performance and could control camera/commands; patients trained before starting the programme	Upper limb: Fugl-Meyer UE, ABILHAND and Ashworth
Prukviwat et al. 2026 [98]	Motion-sensor real-time telerehabilitation	Synchronous remote supervision after two initial hospital sessions	3D camera with motion sensors, monitor, microphone/speaker and real-time posture feedback interface	Same strengthening and balance exercise programme as hospital rehabilitation; system displayed exercise videos and posture feedback using green/red movement indicators	16 total 45 min sessions, twice/week; tele group received 2 hospital sessions plus 14 home telerehabilitation sessions; hospital group received 16 in-person sessions	Remote PT supervision with real-time feedback during home sessions; hospital group supervised one-to-one in person	Balance/walking/mobility activities: BBS, Chula PMS, gait speed and step length
Pastana Ramos et al. 2023 [99]	Synchronous individual telerehabilitation	Real-time videoconference telerehabilitation versus booklet-based home exercise	Smartphone, tablet or computer using free teleconference platforms such as WhatsApp or Google Meet; illustrated exercise booklet for control	Warm-up, mobility, strength, balance and cool-down exercises; progression from familiarization to postural control, unstable surfaces, weight transfer and dynamic balance	12 sessions over 4 weeks; 3 sessions/week; 60 min/session; control instructed to train 3 times/week using booklet	Physiotherapist supervised TR sessions remotely with caregiver present for safety; weekly telephone checks for feedback and adverse effects in both groups	Mobility/motor status: TUG, 5STS, ABC, MDS-UPDRS III and PDQ-8
Salgueiro et al. 2022a [100]	App-guided home core-stability telerehabilitation	Asynchronous app-based adjunct to conventional physiotherapy	Farmalarm App adapted for rehabilitation; exercise descriptions, photos, videos, performance confirmation and researcher administration panel	Thirty-two home core-stability exercises from supine to sitting and unstable-sitting positions; participants encouraged to perform as many exercises as safely possible	12 weeks; target 5 days/week; 10 repetitions of each proposed exercise; both groups continued conventional physiotherapy about 1 h twice/week	Initial in-person app training and short practice; researcher monitored app use and contacted participants by phone regularly	Trunk/balance/gait: S-TIS 2.0, S-FIST, S-PASS, BBS and G-Walk gait parameters
Salgueiro et al. 2022b [101]	App-guided post-discharge core-stability telerehabilitation	Asynchronous on-demand app guidance plus usual care	Farmalarm App with exercise guide, photo/video instructions, self-evaluation and researcher administration panel	Home core-stability exercises previously learned during inpatient rehabilitation, offered after discharge as a guide to continued practice	3-month post-discharge programme; voluntary on-demand app access; usual care frequency averaged about 2.5 sessions/week depending on prescription/availability	Principal researcher monitored app panel and contacted users by phone to encourage use and clarify doubts; experienced physiotherapist available for video calls through the app	Sitting/standing balance and gait: S-TIS 2.0, S-FIST, BBS, S-PASS, BBA and G-Walk
Saygili et al. 2024 [102]	Modified-CIMT telerehabilitation	Synchronous videoconference task practice plus home exercise	Zoom or Skype videoconferencing; mitt for less affected upper limb; task-practice materials delivered to home	M-CIMT with shaping, task practice, behavioural contract, home diary, daily schedule and patient-selected ADL tasks; both groups also performed basic home exercise programme	Tele-CIMT 90 min/day, 5 weekdays/week for 3 weeks; mitt worn 5 h/day, 5 weekdays/week; home exercise in both groups 5 weekdays/week for 3 weeks	Same physiotherapist supervised all online sessions; caregiver present for safety and task assistance; phone guidance for technical issues	Upper limb/ADL: STREAM, FM-UE, WMFT, 9-HPT, grip/pinch strength, MAL-28 and FIM
Saywell et al. 2021 [103]	Hybrid augmented community telerehabilitation	Limited in-person visits plus telephone calls and personalized text messages	Standard mobile phones/text messaging; telephone contact; paper exercise charts and home visits	Patient-centred goal setting and exercises in two functional categories: staying upright and using the arm; exercise parameters individualized and progressed remotely	6-month programme with four in-person home visits, five structured phone calls and personalized text-message reminders/support	Physical therapists delivered ACTIV after training; phone calls addressed adherence, goal strategies and exercise progression; text messages encouraged practice	Physical function: SIS physical subcomponent, grip strength, Step Test, SSEQ and EQ-5D VAS
Sheehy et al. 2025 [104]	Home nonimmersive VR telerehabilitation	Asynchronous home exergaming with weekly telephone support	Jintronix platform; Kinect camera; TV screen; iPad apps for active control; web-based usage data/logbooks	Interactive balance, stepping, strength, reaching and aerobic-capacity games/exercises customized to goals and abilities; iPad control targeted cognition/fine-motor skills	30 min/day, 5 days/week for 6 weeks in both groups	Initial home visit for installation/training; study partner present for technical/motivational support; intervention physiotherapist phoned weekly and progressed NIVRT remotely	Balance/gait/function: BBS, TUG variants, FTSTS, CB&M, SIS and CIQ; feasibility/adherence/safety
Simpson et al. 2025 [105]	Virtually delivered wearable-feedback upper-limb programme	Synchronous virtual therapist sessions plus home exercise and real-time wearable feedback	TENZR wrist-worn reach-to-grasp sensor and app; Microsoft Surface Go tablet; TeamViewer data transfer; H-GRASP materials	Virtual Arm Boot Camp combining adapted H-GRASP exercises, daily task use, reach-to-grasp feedback/targets and problem-solving using COM-B principles	3-week intervention with six virtual treatment sessions; participants asked to perform GRASP twice daily or at least 2 h/weekday and wear TENZR during waking hours	Trained PT/OT delivered virtual sessions, set daily reach-to-grasp targets and progressed exercises; caregiver assistance optional	Upper-limb activity/function: TENZR reach-to-grasp counts, REACH, ArmCAM and QoL-related outcomes
Skelly et al. 2026 [106]	Synchronous individualized physiotherapy telerehabilitation	Real-time video physiotherapy with brief video/telephone reviews	NHS Attend Anywhere or DrDoctor videoconferencing; Fitbit Inspire HR for outcome activity monitoring; tablet loan available if needed	Individualized early-PD physiotherapy addressing physical capacity, transfers, manual dexterity, balance, gait, flexibility, amplitude, strengthening and activity targets	Within 12 weeks: one 60 min video assessment, four 30 min video treatment calls and two 10 min catch-up calls/reviews	Experienced Parkinson physiotherapists delivered individualized prescriptions; participants received education/signposting and informal psychological support as needed	Motor/physical activity/QoL: UPDRS, weekly step count, activity intensity, PDQ-39, SLST, FTSTS and falls
Song et al. 2018 [107]	Home-based exergame step training	Asynchronous home exergaming with periodic therapist support	Modified Stepmania dance/exergame software, small computer connected to home TV/monitor, custom six-target step mat	Visually cued multidirectional step training with inhibition and timing demands; targets included standard arrows, tailed arrows and no-step bomb stimuli	Minimum 15 min/session, 3 sessions/week for 12 weeks; two initial home visits, additional 6-week visit and fortnightly telephone calls	Physiotherapist set up equipment, trained safe use, monitored progress and phoned every 2 weeks; participants kept logbooks and adverse-event records	Stepping/gait/falls: choice stepping reaction time, Functional Gait Assessment, TUG, falls, perceived mobility/balance
Standen et al. 2017 [108]	Low-cost home-based virtual-reality upper-limb rehabilitation	Asynchronous self-directed home VR with therapist setup and ongoing support	Virtual glove with infrared LEDs on fingertips, Nintendo Wiimotes, computer monitor and three purpose-built games	Reaching, grasp/release, pronation–supination and hand-opening practice through Spacerace, Spongeball and Balloonpop games	8-week home programme; patients advised to build up to 20 min, 3 times/day	Therapist installed equipment, provided repeated training as needed, weekly/fortnightly home support, telephone troubleshooting and instruction manual	Upper limb: WMFT, 9-HPT, MAL amount/quality of movement, NEADL
Sun et al. 2025 [109]	Post-discharge multidisciplinary telerehabilitation	Hybrid platform-based remote rehabilitation with scheduled video consultation	Dedicated telerehabilitation platform with video tutorials, interactive games, self-assessment tools and online consultation functions	Individualized physical, occupational and speech-therapy plan plus health education and mental-health support	12 weeks; daily platform-based training plus at least weekly video consultation	Rehabilitation physicians/therapists reviewed progress, adjusted plans and provided live/recorded guidance; psychological counselling available	Global ADL/disability/QoL: Barthel Index, mRS, HAMD, WHOQOL-BREF
Swarnakar et al. 2023 [110]	Telephone-based telerehabilitation and tele-coaching for SCI	Synchronous/telephone telerehabilitation plus prescribed home practice	Minimum phone-calling facility; remote consent/interview procedures; telephone sessions	PT and OT demonstrations, problem-solving for ADL, SCI-specific advice and COVID-19 precautions; ROM, stretching and strengthening exercises tailored to needs	Biweekly 30 min telerehabilitation sessions for 8 weeks; participants advised PT and OT practice twice daily for 30 min each	Remote therapist contact; data and safety monitoring board available by telephone; control continued previous usual care	SCI independence/anxiety: SCIM III domains and Coronavirus Anxiety Scale
Thomas et al. 2017 [111]	Physiotherapist-facilitated home Nintendo Wii programme	Hybrid home exergaming with structured in-person and telephone/email support	Nintendo Wii console, Wii Balance Board, Wii Fit Plus, Wii Sports, Wii Sports Resort, activity workbook and play log	Personalized active gaming and behaviour-change support to increase activity, balance, fitness and well-being	Immediate group used Mii-vitaliSe for approximately 12 months; comparison at 6 months. Early phase included 2 hospital orientation sessions, home installation at week 3, review visits and ongoing support	Senior physiotherapists provided orientation, risk assessment, home setup, goal setting, monitoring, feedback and monthly support from week 20 onward	Physical activity, balance/walking/mobility activities and dexterity: GLTEQ, activPAL, physical assessments including i-TUG and hand dexterity
Toh et al. 2025a [112]	Wearable-assisted Smart Reminder upper-limb telerehabilitation	Asynchronous home telerehabilitation with remote monitoring and weekly in-person review	Smart Reminder wristwatch with accelerometer/gyroscope, vibration/beep reminders, smartphone telerehabilitation app, encrypted web portal and triaxial accelerometer	Customized upper-limb ROM and functional task practice with real-time visual feedback, repetitions and ROM tracking; affected-arm use monitoring outside training	1 h/day, 5 days/week for 4 weeks; accelerometer worn 3 h/day, 5 days/week outside prescribed training	Occupational therapist prescribed exercises, set reminders, reviewed portal data remotely and provided weekly 45 min laboratory consultation	Upper limb: FMA-UE, ARAT, MAL, AROM, strength, accelerometer movement counts, adherence
Toh et al. 2025b [113]	Smart Reminder wearable plus smartphone telerehabilitation	Two-period crossover delivery comparison: wearable telerehabilitation versus conventional handout therapy	Smart Reminder wristwatch, smartphone/tablet telerehabilitation app, encrypted web portal	Shoulder, elbow and forearm ROM exercises delivered by app video with auditory/vibration prompts and real-time ROM feedback	Each 4-week period: 15 min/session, 3 sessions/day, 5 days/week; 3-week washout between periods	Therapist remotely monitored daily exercise progress, sent reminders and provided weekly 30 min in-person consultation to review and modify exercises	Upper limb: FMA-UE, ARAT, shoulder/elbow/forearm AROM, MAL, adherence
Uswatte et al. 2021 [114]	In-home telehealth Constraint-Induced Movement Therapy	Synchronous remote therapist-supervised delivery-model comparison	Tele-AutoCITE automated upper-extremity workstation with built-in sensors, video cameras, touch-screen, Internet audio-visual/data links	CIMT with shaping-based more-affected-arm task practice, transfer package and restraint of less-affected arm	35 h over 10 consecutive weekdays; 3.5 h/day including 3 h motor training and 30 min transfer-package procedures	Trainer remotely supervised all treatment sessions from laboratory, controlled workstation tasks and completed follow-up transfer-package phone calls	Upper limb: MAL Arm Use, WMFT, satisfaction/difficulty
Vasconcellos et al. 2023 [115]	Home-based exercise telerehabilitation for PD	Asynchronous home exercise with daily telephone/instant-message monitoring	Exercise booklets and videos delivered by email or mobile messaging; phone/instant messaging for monitoring	Experimental trunk/pelvic-floor strengthening versus control global upper/lower-limb exercises	Home exercises 3 times/day for 3 consecutive weeks	Initial in-person instruction for participants/caregivers; study therapists contacted participants/caregivers daily to monitor adherence and clarify doubts	Balance/gait: COP stabilometry, gait speed, hip/knee/ankle ROM, MDS-UPDRS III
Vloothuis et al. 2019 [116]	Caregiver-mediated e-health supported stroke telerehabilitation	Hybrid caregiver-mediated programme with app-based exercise support and optional telecontact	E-health application with 37 standardized mobility exercises; telephone, video conferencing or email for contact	Patient-tailored progressive caregiver-mediated mobility/gait-related exercises added to usual care	8 weeks; target additional 150 min/week (5 × 30 min/week), continued at home if discharged before completion	Trained physical therapist selected/progressed exercises in weekly sessions; caregiver executed exercises; dyads could contact therapist between sessions	Mobility/ADL: SIS mobility, LOS, FMA-LE, Motricity Index, 6MWT, 10MWT, TUG, BBS, RMI, BI, NEADL
Wang et al. 2024 [117]	Internet remote home rehabilitation plus wearable lower-limb device	Hybrid app-based remote guidance with wearable-device training	Cloud Ward app, point-to-point video communication, Bluetooth blood-pressure/heart-rate monitoring, smart heart-rate belt, lower-limb wearable device	Individualized remote home rehabilitation plan plus lower-limb wearable walking training and real-time monitoring of vital signs and training data	Remote training guidance and wearable walking training twice daily for 30 min, 6 days/week; outcomes at 4 and 12 weeks after discharge	Internet + home rehabilitation team reviewed training weekly, adjusted plans dynamically and monitored vital signs/exercise data in real time	Motor/balance/ADL/gait and biomarkers: FMA, MAS, BBS, MBI, HADS, gait parameters, BDNF/NT-3/NGF
Wilson et al. 2021 [118]	Home-based EDNA-22 virtual rehabilitation	Asynchronous home virtual rehabilitation with cloud performance monitoring and weekly phone support	EDNA-22 portable tablet-based virtual rehabilitation system, tangible interface/objects, cloud data storage	Tailored upper-limb virtual movement tasks with scaled motor-cognitive complexity and performance feedback	30 min/session, 3–4 sessions/week for 8 weeks	Therapy schedule delivered via Internet; performance/adherence data stored in cloud and relayed to therapist; weekly phone contact	Upper limb/cognition/function: BBT, 9-HPT, MoCA, SIS, NFI
Wolf et al. 2015 [119]	Telemonitored robotic-assisted home upper-limb rehabilitation	Store-and-forward robotic telerehabilitation with weekly therapist monitoring	Hand Mentor Pro pneumatic robotic wrist/hand device with touch-screen games, web-based monitoring and remote data transmission	HMP spasticity-reduction/basic/advanced motor-control modules plus home exercise programme	Target 3 h/day, 5 days/week for 8 weeks: experimental 2 h HMP + 1 h HEP; control 3 h HEP with matched dose	Study therapist trained participants at home, reviewed secure web data, adjusted HMP/HEP settings and contacted participants weekly by phone/email	Upper limb: ARAT, WMFT, FMA-UE
Wu et al. 2020 [120]	Collaborative-care remote rehabilitation exercise training	Synchronous Internet-based remote rehabilitation guidance after discharge	TCMeeting v6.0 videoconferencing system with computer, projector, camera and data storage	Collaborative-care rehabilitation plan including education, limb positioning, joint activity, turning, balance, walking, antispasmodic training, active limb activity and ADL training	Remote rehabilitation instruction twice/week after discharge; assessments at discharge and 4, 8 and 12 weeks	Team of neurologists, nurses, rehabilitation therapists, counsellors and caregivers; rehabilitation engineer/nurse provided personalized remote instructions	Motor/balance/mobility/ADL/QoL: FMA, BBS, TUG, 6MWT, MBI, SS-QOL
Yokota et al. 2025 [121]	IoT ergometer-based home telerehabilitation	Synchronous real-time supervised home aerobic exercise	Integrated Remohab platform with IoT-equipped ergometer, Android tablet and wireless ECG monitor	Home aerobic exercise using stationary ergometer plus resistance exercises (sitting calf raises and sit-to-stand squats) with healthcare advice	3-Month programme, 1–3 sessions/week; session duration targeted at 60 min; exercise intensity Borg 11–13	Experienced nurse supervised sessions in real time via platform; heart rate/ECG transmitted by Wi-Fi; patients reported BP, weight and condition before each session	Exercise capacity/strength and HRQOL: 6MWD, IKEMS, handgrip strength, SF-36
Zheng et al. 2026 [122]	Remotely delivered home/community exercise training with behavioural coaching	Asynchronous home/community exercise with scheduled remote coaching	Training manuals with instruction videos, pedometers, resistance bands, yoga mats, Zoom coaching, action-planning calendars and self-monitoring logs	Aerobic walking plus resistance exercises for exercise group; active stretching control matched for contact and behavioural content	16 weeks; 3 days/week. Aerobic 10–30 min moderate walking plus resistance training 1–2 sets of 10–15 repetitions across 5–10 exercises	One-on-one behavioural coaching via Zoom, SCT-based newsletters, logbooks and action-planning; 16-week no-contact follow-up	Physical/cognitive/activity: SPPB, 30STS, TUG, T25FW, 6MWT, BICAMS, GLTEQ, accelerometry

Abbreviations: ABC, Activities-Specific Balance Confidence Scale; AEMF, Automated Evaluation of Motor Function; ADL, activities of daily living; ARAT, Action Research Arm Test; ATTG/STTG, asynchronous/synchronized telerehabilitation treatment group; BBS, Berg Balance Scale; BI, Barthel Index; BBT, Box and Blocks Test; CAHAI, Chedoke Arm and Hand Activity Inventory; CNN, convolutional neural network; CST, corticospinal tract; DGI, Dynamic Gait Index; EDSS, Expanded Disability Status Scale; EM/AC, enhanced motivation/algorithm-controlled; ETNS, electromyography-triggered neuromuscular stimulation; FAC, Functional Ambulation Category; FGA, Functional Gait Assessment; FIM, Functional Independence Measure; FMA/FMA-UE/FMUE/UE-FM/UEFMA, Fugl-Meyer Assessment/upper extremity; fMRI, functional magnetic resonance imaging; FONEFIM, Telephone Version of the Functional Independence Measure; HADS, Hospital Anxiety and Depression Scale; HAT, home automated tele-management; HBR, home-based rehabilitation; HEP, home exercise programme; HoVRS, Home Virtual Rehabilitation System; HRR, heart-rate reserve; IHMD, in-home messaging device; IMI, Intrinsic Motivation Inventory; ISNCSCI, International Standards for Neurological Classification of Spinal Cord Injury; JHFT, Jebsen Hand Function Test; K-ICARS, kinetic function subparameter of the International Cooperative Ataxia Rating Scale; LLFDI, Late-Life Function and Disability Instrument; MAL, Motor Activity Log; MAS, Modified Ashworth Scale; MBI, Modified Barthel Index; MDS-UPDRS, Movement Disorders Society-Unified Parkinson’s Disease Rating Scale; MFAC, Modified Functional Ambulatory Category; MFES, Modified Falls Efficacy Scale; MMDT, Minnesota Manual Dexterity Test; MS, multiple sclerosis; MSQOL-54, Multiple Sclerosis Quality of Life-54; MSWS-12, 12-item Multiple Sclerosis Walking Scale; MU/SU, multi-user/single-user; NBS, Navigated Brain Stimulation; NEADL, Nottingham Extended Activities of Daily Living; N-HPT/9-HPT, Nine-Hole Peg Test; OT/PT, occupational/physical therapy; PCS, Physical Component Summary; PDQ-8/PDQ-39, Parkinson’s Disease Questionnaire; QoL, quality of life; RGS, Rehabilitation Gaming System; ROM, range of motion; SCI, spinal cord injury; SCIM, Spinal Cord Independence Measure; SEE, self-efficacy for exercise; SIBT, sensory integration balance training; SIS, Stroke Impact Scale; T25FW, Timed 25-Foot Walk; 2MWT, 2 min walk test; 6MPT/6MWT, 6 min push/walk test; tDCS, transcranial direct current stimulation; TIS, Trunk Impairment Scale; TOCT-TR, task-oriented circuit training-based telerehabilitation; TR, telerehabilitation; TUG, Timed Up and Go; UE, upper extremity; UEMS/LEMS, upper/lower-extremity motor scores; UTRT, usual territorial rehabilitative treatment; VAS, Visual Analogue Scale; VERGE, Virtual Environment for Rehabilitative Gaming Exercises; VR, virtual reality; VRBT, Virtual Reality Balance Trainer; VRRS, Virtual Reality Rehabilitation System; V-TOCT, virtual reality-supported task-oriented circuit therapy; WISCI, Walking Index for Spinal Cord Injury; WMFT, Wolf Motor Function Test; WHOQOL-BREF, World Health Organization Quality of Life-BREF; APDDS, Adapted Patient Determined Disease Steps; BICAMS, Brief International Cognitive Assessment for MS; BDNF, brain-derived neurotrophic factor; CAS, Coronavirus Anxiety Scale; CIMT, Constraint-Induced Movement Therapy; EDNA, Elements system; FTHUE-HK, Functional Test for Upper Extremity-Hong Kong; HMP, Hand Mentor Pro; HRQOL, health-related quality of life; IoT, Internet of Things; MAL-AOU, Motor Activity Log-Amount of Use; MAL-QOM, Motor Activity Log-Quality of Movement; Mii-vitaliSe, MS Nintendo Wii intervention package; NIVRT, nonimmersive virtual reality training; NT-3, neurotrophin-3; PSM, propensity-score matching; SR, Smart Reminder; TIA, transient ischemic attack; V-ABC, Virtual Arm Boot Camp; ACTIV, Augmented Community Telerehabilitation Intervention; AROM, active range of motion; HCAD, Home Care Activity Desk; HAMD, Hamilton Depression Rating Scale; IT-AAT, Italian version of the Aachen Aphasia Test; OCS, Oxford Cognitive Screen; PASS, Postural Assessment Scale for Stroke; PHQ-9, Patient Health Questionnaire-9; STREAM, Stroke Rehabilitation Assessment of Movement Scale; TENZR, wearable reach-to-grasp feedback device; WHODAS, World Health Organization Disability Assessment Schedule; A-VO2, arteriovenous oxygen difference.

**Table 3:** Study-level motor outcomes, main findings, and synthesis interpretation.

Study	Comparison/Time Point	Main Motor Outcome(s)	Telerehabilitation Result	Comparator Result	Between-Group Finding	Adherence, Safety and Follow-Up	Synthesis Interpretation
García-Rudolph et al. 2024 [36]	TeleSCI versus matched historical in-person inpatient rehabilitation; discharge assessment	FIM, SCIM III, WISCI II; gains, efficiency and effectiveness	The teleSCI cohort achieved functional and mobility gains across FIM, SCIM III and WISCI II during a mean teleSCI duration of 67 days.	Matched historical controls received conventional inpatient rehabilitation with comparable discharge outcomes and gain metrics.	No significant differences were observed between teleSCI and matched controls in FIM, SCIM III or WISCI II admission/discharge scores, gains, efficiency or effectiveness.	Home teleSCI was delivered with daily caregiver support, nursing/physician monitoring and MDT review; safety concerns requiring systematic exclusion from home care were not emphasized.	Supports teleSCI as a feasible alternative to inpatient rehabilitation in medically stable, supported patients with SCI; use narrative/sensitivity interpretation because of matched observational design.
Thielbar et al. 2020 [37]	Multi-user versus single-user home VR telerehabilitation; post-intervention after 4-week crossover	Arm displacement/session, session duration, FMA-UE, compliance and IMI	Multi-user VERGE sessions generated greater arm displacement than single-user sessions (414.6 m vs. 327.0 m; *p* = 0.019) and participants trained longer within sessions (*p* = 0.04).	Single-user VERGE was also feasible and had high compliance, but training engagement was lower than in multi-user mode.	The delivery mode significantly favoured multi-user VR for movement amount and session time; FMA-UE improved across all participants by 3.2 points (*p* = 0.001), without a conventional-care comparator.	Compliance was very high in both modes (99% multi-user; 89% single-user); no long-term follow-up was reported.	Suggests social/multi-user VR may enhance engagement within home telerehabilitation; use as a delivery-model comparison rather than primary telerehabilitation-versus-control efficacy evidence.
Adams et al. 2023 [38]	GRASP home VR telerehabilitation versus usual and customary care; post-intervention at 8 weeks	FMUE; WMFT; BBT; MAL	GRASP participants showed a significant FMUE improvement of 10.1 points (95% CI 6.7 to 13.5; *p* < 0.001) and clinically/statistically meaningful gains in MAL scores.	Usual and customary care participants showed no significant FMUE change (mean change 1.6 points; 95% CI −1.9 to 5.0; *p* = 0.351).	The between-group FMUE change favoured GRASP by 8.6 points (95% CI 3.7 to 13.4; *p* = 0.002), remaining significant after adjustment (*p* < 0.001).	No adverse incidents or harm were reported; intervention combined independent home sessions with biweekly synchronous OT support.	Provides supportive RCT evidence for IADL-focused home VR telerehabilitation for upper-limb recovery after stroke; candidate for primary quantitative synthesis if outcome/time point is compatible.
Allegue et al. 2022 [39]	VirTele exergame telerehabilitation versus conventional home GRASP programme; post-intervention and 1- to 2-month follow-up	FMA-UE; MAL-30; SIS-16; TSRQ-15; feasibility metrics	VirTele was feasible for most participants; more than half of participants improved on FMA-UE and MAL-30, and 75% of the experimental group increased autonomous motivation after the intervention.	The conventional GRASP group also showed improvement in more than half of participants on FMA-UE and MAL-30; SIS-16 improvements were more consistently reported for some control domains.	Between-group efficacy conclusions were limited by the very small feasibility sample and COVID-19-related protocol extension; outcome patterns were mixed rather than definitively superior.	Technical issues included passwords, Internet problems, software updates and avatar problems; most participants considered the technology useful/easy to use, except one participant.	Useful feasibility evidence for home exergame telerehabilitation with clinician monitoring, but peer-review-safe synthesis should emphasize preliminary and underpowered findings.
Asano et al. 2021 [40]	STARS home telerehabilitation versus usual rehabilitation care; primary endpoint at 3 months	LLFDI frequency and limitation total scores; 5 m walk; 2MWD; MBI; ABC; EQ-5D	The telerehabilitation group improved from baseline in self-reported function and secondary physical outcomes over 3 months.	Usual-care participants showed similar improvements and reported a comparable overall amount of rehabilitation/exercise time.	No significant between-group differences were found for LLFDI frequency (MD −3.30; 95% CI −7.81 to 1.21) or limitation scores (MD −6.90; 95% CI −15.02 to 1.22), or for secondary outcomes.	Modified ITT included 50 telerehabilitation and 48 usual-care participants at 3 months; telerehabilitation exposure was lower than total reported exercise/rehabilitation time.	Suggests comparable functional improvement rather than superiority for early post-stroke telerehabilitation; relevant to usual-care comparator synthesis and implementation discussion.
Aytutuldu et al. 2024 [41]	Synchronous LSVT BIG versus synchronous progressive structured mobility training; post-intervention at 4 weeks	Mini-BESTest; TUG; gait parameters; Biodex postural stability; ABC-SF; PAS; PDQ-39	Both synchronous telerehabilitation protocols improved TUG, gait parameters and quality of life; LSVT BIG produced additional gains in dynamic balance-related outcomes.	Progressive structured mobility training also improved mobility and gait outcomes when delivered synchronously online.	Group-by-time interactions favoured LSVT BIG for Mini-BESTest (*p* = 0.042), ABC-SF (*p* = 0.029) and PAS (*p* = 0.022); other mobility/gait outcomes improved in both groups.	Two randomized participants did not complete training, but ITT analyses produced similar results; no durability follow-up beyond post-treatment was reported.	Supports synchronous telerehabilitation feasibility in early-to-mid PD and suggests possible added value of amplitude-focused LSVT BIG; use as delivery/content comparison only.
Ballester et al. 2017 [42]	Home-based RGS VR therapy versus matched home occupational-therapy task; post-intervention at 3 weeks and 12-week follow-up	UE-FM; CAHAI; BI; MRC; grip force; spasticity/pain; NBS markers	RGS training produced significantly greater functional recovery than the control task on CAHAI (reported between-group difference 1.53, SD 2.4) and was accompanied by neurophysiological changes.	Home occupational-therapy task practice was designed to match movement content but without the adaptive VR environment.	RGS favoured the experimental group statistically, although functional improvements did not reach clinical significance; cortical-map displacement correlated with clinical-scale improvements.	Participants/caregivers were trained to use the system at home; formal clinical assessments were repeated at 12 weeks.	Suggests that adaptive home VR may induce modest functional gains and neuroplastic changes, but clinical relevance should be interpreted cautiously.
Benvenuti et al. 2014 [43]	Home/community exercise plus kiosk telerehabilitation versus usual care; post-intervention at 3 months	Motricity Index; WMFT; 9-HPT; BI; NEADL; SPPB; SIS	The intervention group improved more than usual care on upper-extremity impairment/function and disability outcomes, including Motricity Index (+6.5 vs. +0.1), WMFT (+8.9 vs. +0.3) and 9-HPT (−3.4 vs. +0.8).	Usual care produced minimal or no change across the primary upper-limb outcomes over 3 months.	Between-group changes favoured the intervention for Motricity Index (*p* < 0.0001), WMFT (*p* < 0.0001), 9-HPT (*p* < 0.009), BI (*p* = 0.0001) and NEADL (*p* < 0.002).	Satisfaction was high and no adverse events were reported; only 30% attended kiosks regularly, and kiosk users did not differ clearly from home-only users.	Supports home/community task-oriented exercise for chronic post-stroke upper-limb paresis, with the added kiosk telerehabilitation component showing limited incremental adherence signal.
Calabrò et al. 2023 [44]	Teleneuro-VRRS HomeKit versus usual territorial rehabilitative treatment; post-intervention after 12 weeks	BI; Tinetti Scale; MAS; MoCA; FAB; BDI-II; SF-36; PGWBI; CBI	The teleneuro-VRRS group improved significantly in functional independence and multidomain cognitive/psychological outcomes; BI, FAB and BDI-II showed the strongest improvements (all *p* < 0.001).	Usual territorial rehabilitative treatment involved home motor and/or cognitive therapy without the VRRS HomeKit remote platform.	Between-group effects favoured teleneuro-VRRS for selected well-being/QoL subdomains, including PGWBI anxiety (ES = 0.85; *p* < 0.02), PGWBI self-control (ES = 0.40; *p* < 0.03) and SF-36 social role functioning (ES = 0.85; *p* < 0.02).	Caregivers acted as co-therapists; caregiver burden improved significantly in the teleneuro-VRRS group (CBI; *p* < 0.004).	Suggests integrated motor-cognitive VR telerehabilitation may improve functional and psychosocial outcomes after severe acquired brain injury; motor-specific pooling may be limited by multidomain intervention/outcomes.
Carey et al. 2007 [45]	Finger/wrist tracking telerehabilitation versus simple movement telerehabilitation; post-intervention after 2 weeks plus crossover	Box and Blocks Test; Jebsen–Taylor test; finger ROM; fMRI activation	Tracking training improved all four behavioural tests within group after 1800 home-based telerehabilitation trials.	Simple movement training improved Box and Block and Jebsen–Taylor performance but not all behavioural measures.	Tracking did not clearly outperform simple movement practice on key functional tests, and no consistent group pattern of cortical reorganization was demonstrated.	The move group did not show further significant gains after crossing over to tracking; 2-week dose may have been insufficient for differential learning effects.	Indicates that home computerized telerehabilitation can improve hand performance, but accuracy-based tracking was not demonstrably superior to dose-matched movement practice.
Chae et al. 2020 [46]	Wearable/smartwatch HBR system versus conventional home exercise handout; 12-week post-intervention and 6-week post-system follow-up	WMFT; FMA-UE; grip power; shoulder ROM; BDI; exercise-recognition accuracy	The HBR group showed significant improvement in mean WMFT score (*p* = 0.02) and shoulder ROM for flexion (*p* = 0.004) and internal rotation (*p* = 0.001).	The control group showed a significant change only in shoulder internal rotation (*p* = 0.03), with high attrition limiting interpretation.	Comparative inference is limited by non-randomized/prospective comparative design and small control sample; technology-supported monitoring appeared to facilitate participation.	Dropout was lower in HBR than control at 12 weeks (22% vs. 40%) and 18 weeks (45% vs. 100%); ML exercise recognition reached 99.80% using personalized accelerometer plus gyroscope data.	Provides supportive but non-randomized evidence that wearable-assisted home rehabilitation can improve upper-limb function and adherence; use narrative/sensitivity only.
Chantanachai et al. 2025 [47]	Active home tDCS plus tele-exercise versus sham tDCS plus identical tele-exercise; post-intervention and 1-month follow-up	ISNCSCI UEMS/LEMS and sensory scores; SCIM-III; TAI; H-reflex/m-MAS; HHD; WHOQOL-BREF	Active tDCS plus tele-exercise improved UEMS at 1-month follow-up within the active group (Bonferroni post hoc *p* = 0.002).	Sham tDCS plus the same tele-supervised exercise did not show comparable UEMS improvement.	A group-by-time interaction was observed for UEMS (F(1,18) = 4.49; *p* = 0.043), but no significant effects were found for sensory, spasticity, functional, transfer or QoL outcomes.	All participants completed the protocol; 27% needed assistance setting up tDCS; adverse effects were mild cutaneous sensations only.	Relevant as adjunct neuromodulation evidence embedded in telerehabilitation, not as a primary test of telerehabilitation versus non-telerehabilitation.
Chen et al. 2017 [48]	Home-based telesupervising rehabilitation versus conventional outpatient rehabilitation; post-intervention at 12 weeks and follow-up at 24 weeks	MBI; BBS; mRS; CSI; RMS of ECRL and TA	Home telesupervising rehabilitation improved MBI, BBS, ECRL/TA RMS and caregiver strain over time.	Conventional outpatient rehabilitation showed similar within-group improvements over the same time points.	No significant between-group differences were detected; both groups improved across MBI, BBS, RMS measures and CSI (within-group time effects *p* < 0.001).	Caregivers kept training logs and therapists supervised remotely via live video; follow-up effects were assessed 12 weeks after treatment completion.	Supports home tele-supervision as a likely equivalent alternative to outpatient rehabilitation for hemiplegic stroke recovery and caregiver burden.
Chen et al. 2020 [49]	Home-based motor telerehabilitation versus outpatient conventional rehabilitation; post-intervention at 12 weeks and follow-up at 24 weeks	FMA; MBI; M1-M1 rsFC; M1 GMV; CST integrity	TR produced larger FMA improvement than CR at 12 weeks (mean change 11.115 vs. 5.307) and was associated with increased M1-M1 rsFC (0.424 vs. 0.219).	CR improved motor/ADL outcomes but less than TR for FMA; no superiority was shown for MBI.	TR was non-inferior for FMA and MBI and superior for FMA (*p* = 0.011); M1-M1 rsFC favoured TR (MD 0.204; 95% CI 0.074 to 0.336; *p* = 0.031).	No study-related adverse events were reported; week-24 primary outcomes were no longer significantly different.	Strong RCT candidate for primary motor synthesis; durability should be interpreted cautiously because post-intervention between-group differences were not maintained at follow-up.
Chen et al. 2021 [50]	Interactive Kinect-based telerehabilitation versus conventional one-to-one physiotherapy; post-intervention at 4 weeks	BBS; TUG; MFES; Motricity Index; FAC	Interactive telerehabilitation significantly improved BBS (*p* = 0.01; ES = 0.70) and TUG completion time (*p* = 0.005; ES = 0.70).	Conventional physiotherapy also significantly improved BBS (*p* = 0.01; ES = 0.49).	No significant between-group differences were found at post-intervention for any outcome.	The hospital-based simulated-home setup required caregiver/family standby for fall prevention; no longer-term follow-up was reported.	Supports interactive telerehabilitation as comparable to conventional physiotherapy for chronic stroke balance training; pilot design warrants cautious synthesis.
Chumbler et al. 2012 [51]	Multifaceted STeleR versus usual care; outcomes at 3 months and 6 months	Motor FONEFIM; LLFDI function and disability domains	STeleR participants improved on complementary primary function outcomes by 6 months and showed favourable changes in several LLFDI disability domains.	Usual-care participants tended to decline on primary function outcomes over the same period.	Primary outcomes did not reach statistical significance (LLFDI Function *p* = 0.25; Motor FONEFIM *p* = 0.316); 4 of 5 LLFDI disability subscales favoured STeleR at 6 months (*p* < 0.05).	The intervention used three televisits, daily in-home messaging and five telephone calls; no serious study-related adverse events were reported.	Suggests potential functional/disability benefit of multifaceted stroke telerehabilitation, but primary null findings require cautious interpretation in quantitative synthesis.
Chung et al. 2020 [52]	Mobile video-guided home exercise versus paper-based home exercise; follow-up at 1 day, 1 month and 3 months	Exercise adherence; SEE; MFAC gain; MBI gain	Video-guided delivery achieved higher 3-month adherence (75.6%) and higher SEE scores at 1 month (58.4) and 3 months (62.2).	Paper-based home exercise had lower 3-month adherence (55.2%) and lower SEE scores at 1 month (43.3) and 3 months (45.6).	Video-guided exercise was superior for 3-month adherence, SEE and MFAC gain (1.7 vs. 1.0), but not for MBI gain.	Intervention required smart device/QR-code access; outcome data were collected by blinded telephone follow-up.	Useful evidence that video-based delivery can improve adherence and mobility gain after stroke discharge; may be more relevant to adherence/digital-delivery synthesis than motor effect pooling.
Cikajlo et al. 2012 [53]	VR-supported balance telerehabilitation versus balance training without VR/telerehabilitation support; post-intervention and follow-up	BBS; TUG; 10 m walk test; single-limb stance; VR task time/collisions	VR-supported balance training improved BBS by 15%, TUG by 29%, 10 m walk by 26%, and affected/unaffected stance time by 200% and 67%, respectively.	Conventional standing-frame balance training without VR/telerehabilitation achieved comparable functional progress.	No statistical differences were found between VR telerehabilitation and conventional balance training in overall level or mean improvement.	Gains were preserved at follow-up; VR task time and collisions decreased by 45% and 68%, respectively.	Pilot evidence supports feasibility and comparable effects of VR-supported balance telerehabilitation, but small sample and shared control group limit inferential strength.
Conroy et al. 2018 [54]	MS HAT Internet-supported home exercise versus routine home exercise; outcomes at 3 and 6 months	T25FW; 6MWT; BBS; MSWS-12; adherence	MS HAT participants largely maintained gait outcomes over 6 months but did not show significant improvement on T25FW or 6MWT.	Routine home exercise showed small, non-significant improvements on some walking measures in a smaller completer sample.	No significant difference was found for T25FW change at 6 months (*p* = 0.44) or 6MWT; BBS change differed in a negative direction for MS HAT (*p* = 0.04).	Attrition was high but lower in MS HAT than control (38% vs. 68%); adherence was associated with higher baseline disability and self-reported walking ability.	Suggests that asynchronous self-directed tele-management alone may be insufficient for clear walking gains in mostly progressive MS; use cautiously in narrative synthesis.
Coulter et al. 2017 [55]	Web-based physiotherapy versus usual care/self-management; post-intervention at 8 weeks	6MWT or 6MPT; HR/RPE; muscle strength; HADS; WHOQOL-BREF	The intervention group improved on 6MPT/6MWT; the mean 6MWT gain was 58 m, exceeding the reported minimal detectable change of 45.8 m.	Usual-care participants reported lower exercise frequency and less pronounced physical-capacity gains.	Between-group differences were not statistically significant, although the largest signal favoured web-based physiotherapy for 6MWT.	Participants logged in 1.4 ± 0.8 times/week; no adverse events were reported; satisfaction was high and participants rated the programme good/excellent.	Feasibility evidence supports acceptability of web-based physiotherapy after SCI, but the small heterogeneous pilot sample limits efficacy conclusions.
Cramer et al. 2019 [56]	Home-based telerehabilitation versus dose- and intensity-matched in-clinic therapy; primary motor endpoint 30 days after therapy	FM arm motor score; Box and Blocks Test; SIS hand; stroke knowledge; adherence	The TR group achieved a mean FM improvement of 7.86 points (SD 6.68; *p* < 0.001) and completed 35.4/36 assigned sessions (98.3%).	The in-clinic group achieved a mean FM improvement of 8.36 points (SD 7.04; *p* < 0.001) and completed 33.6/36 sessions (93.3%).	The adjusted FM change was 0.06 points higher in TR (95% CI −2.14 to 2.26; *p* = 0.96). The non-inferiority margin was 2.47 and fell outside the CI, supporting the non-inferiority of home TR.	Technical issues decreased over time. Serious adverse events were unrelated to study procedures; nonserious related events were mainly fatigue or arm/shoulder pain.	Strong non-inferiority evidence that intensive upper-limb telerehabilitation can deliver comparable motor gains to clinic-based therapy after stroke; candidate for quantitative synthesis.
Dastan et al. 2025 [57]	Synchronous videoconference telerehabilitation versus asynchronous video-based telerehabilitation; post-intervention at 8 weeks	N-HPT; JAMAR grip strength; AMSQ; MFIS; PBMSI; IPA; BPI; PA/SWA	Synchronous telerehabilitation improved dominant and non-dominant N-HPT, dominant JAMAR grip strength, AMSQ, MFIS physical/total, PBMSI fatigue/total and IPA out-of-home activities (*p* < 0.05).	The asynchronous group performed the same exercises through instructional videos; only IPA social life/relationships improved within group, and upper-limb changes were less pronounced.	Between-group change favoured synchronous treatment for N-HPT in both dominant and non-dominant hands (*p* < 0.05); other domains should be interpreted as exploratory.	Of 30 randomized PwMS, 25 were analyzed; dropouts reflected transportation, incomplete assessment, inability to adapt to the system and COVID-19. Pain did not change significantly.	Supports therapist-guided synchronous telerehabilitation as more effective than asynchronous video delivery for manual dexterity in PwMS, but sample size remains pilot-level.
Van den Berg et al. 2016 [58]	Caregiver-mediated exercise with e-health/telerehabilitation support versus usual rehabilitation care; outcomes at 8 and 12 weeks plus 12-month readmission follow-up	SIS mobility; NEADL; LOS; readmissions; caregiver fatigue/self-efficacy and burden	Intention-to-treat analysis showed no primary SIS mobility benefit, but caregivers reported less fatigue and higher self-efficacy at week 12. In the home telerehabilitation per-protocol subgroup, mobility showed a favourable trend and extended ADL improved.	Usual care consisted of interdisciplinary stroke rehabilitation without the structured caregiver-mediated e-health programme.	ITT SIS mobility was neutral (*p* = 0.6). Caregiver fatigue (4.6, 95% CI 0.3 to 8.8; *p* = 0.04) and self-efficacy (−3.3, 95% CI −5.7 to −0.9; *p* = 0.01) favoured the intervention. Per-protocol analyses showed improved NEADL at week 8 (*p* = 0.01) and week 12 (*p* = 0.03), 9-day shorter LOS (*p* = 0.046), and fewer readmissions (*p* < 0.05).	No adverse events were reported. Of 31 intervention participants, 20 received the home telerehabilitation component after discharge.	Proof-of-concept evidence suggests caregiver-mediated e-health support may improve transition outcomes and caregiver measures, but the primary ITT motor/mobility outcome was neutral.
Deng et al. 2012 [59]	Complex ankle tracking telerehabilitation versus simple ankle movement telerehabilitation; post-intervention after 4 weeks	Ankle dorsiflexion during gait; 10 m walk/gait analysis; fMRI activation during ankle tracking	Complex tracking training produced significantly greater dorsiflexion during gait than simple movement training and was feasible for home-based use.	Simple movement training used dose-matched ankle dorsiflexion/plantarflexion practice without tracking accuracy demands.	Gait dorsiflexion favoured the complex tracking group. fMRI activation volume, percent volume and intensity did not show significant continuous changes, but the frequency of participants showing increases versus decreases differed between groups.	Sixteen participants completed the trial. No follow-up assessment was conducted, and the study was limited by small sample size.	Suggests that complex task-specific telerehabilitation may improve ankle control after chronic stroke; best used as delivery/content-comparison evidence rather than broad efficacy evidence.
Van der Kolk et al. 2019 [60]	Home-based remotely supervised aerobic exergaming versus home-based stretching active control; post-intervention at 6 months	Off-state MDS-UPDRS motor score; Mini-BESTest; TUG; 6MWT; cardiovascular fitness; adherence and adverse events	The aerobic exergaming group showed attenuation of motor worsening, with mean off-state MDS-UPDRS motor change of 1.3 points (SE 1.8).	The active control group performed home stretching/flexibility/relaxation with the same motivational app and remote supervision; mean off-state MDS-UPDRS motor change was 5.6 points (SE 1.9).	The between-group adjusted difference was 4.2 points (95% CI 1.6 to 6.9; *p* = 0.0020) in favour of aerobic exercise.	A total of 130 participants were randomized and 125 analyzed. Twenty did not complete the assigned programme. Potentially related adverse events occurred in 7 aerobic and 4 control participants; unrelated serious adverse events occurred in 3 and 4, respectively.	High-quality evidence that remotely supervised home aerobic exercise can attenuate motor signs in mild PD; synthesis should note that the comparator was also home-based and remotely supported.
Dogan et al. 2023 [61]	Mobile-app-based telerehabilitation versus clinic-based VR-supported task-oriented circuit therapy; post-intervention at 8 weeks	TIS; K-ICARS; ABILHAND; MMDT; UL and trunk kinematics (FRoM, LDJ, SPARC)	TR improved trunk impairment, ataxia severity, UL and hand function, with increased trunk FRoM in coronal/transverse planes.	V-TOCT also improved trunk impairment, ataxia severity, UL and hand function, and showed UL FRoM/smoothness gains during functional reaching.	Dynamic TIS and K-ICARS improved more with V-TOCT than with TR (*p* < 0.05); no between-group differences were found for ABILHAND, MMDT, most UL/trunk kinematic metrics or static/coordination TIS components.	Thirty-four PwMS were randomized and 32 completed the study; two TR participants were lost/discontinued because of week-4 loss to follow-up and COVID-19 hospitalization.	Shows that app-based TR can improve UL/trunk function in MS, but clinic-based VR task-oriented circuit therapy may provide stronger dynamic trunk and kinetic function benefits.
Eldemir et al. 2023 [62]	Task-oriented circuit training-based telerehabilitation plus home exercise versus home exercise alone; post-intervention at 6 weeks	9-HPT; JHFT; grip and pinch strength; UPDRS-III; UPDRS-II; PDQ-8	TOCT-TR significantly improved dexterity, hand function, grip/pinch strength and motor symptom severity, with additional improvements in ADL and QoL.	The control group completed home exercises targeting balance, walking and mobility activities; ADL and QoL also improved over time but upper-limb gains were smaller.	Group-by-time interactions favoured TOCT-TR for 9-HPT (dominant *p* = 0.002; non-dominant *p* = 0.010), JHFT (*p* < 0.001), grip strength (*p* < 0.001), pinch strength (*p* ≤ 0.015), and UPDRS-III (*p* = 0.007). Interactions for UPDRS-II and PDQ-8 were not significant.	Thirty-two PwPD were included; medication did not change during the study. Sessions were delivered by videoconference 3 days/week.	Supports synchronous task-oriented upper-limb telerehabilitation in PD, especially for dexterity and strength outcomes; candidate for motor-domain narrative or quantitative synthesis if compatible.
Eldemir et al. 2024 [63]	Pilates-based videoconference telerehabilitation versus waitlist control; post-intervention at 6 weeks	Extremity strength; core endurance/power; BBS/BioSway balance; gait speed/cadence; 6MWT; FSS/FIS; MSQOL-54	Pilates-TR improved most upper/lower-extremity strength measures, core endurance/power, functional balance, posturography, walking speed, cadence, functional exercise capacity, fatigue and QoL (*p* < 0.05).	The waitlist control group showed no meaningful physical performance or QoL changes and had increased fatigue severity (*p* < 0.005).	Between-group interpretation favours Pilates-TR across physical performance, fatigue and QoL domains, although the study primarily reports within-group changes across multiple outcomes.	Thirty PwMS were analyzed. No harmful events were reported, and session participation averaged 97.34% (range 88.89–100).	Supports videoconference Pilates as a feasible and well-adhered home intervention for MS; use cautiously for quantitative pooling because comparator was waitlist/no active treatment.
Fjeldstad-Pardo et al. 2018 [64]	Tele-supervised PT versus in-person PT versus unsupervised home exercise; post-intervention at 8 weeks	FGA; T25FW; BBS; NeuroCom gait/balance metrics; ABC; MFIS; SF-36; MSSE	The TR group improved selected outcomes, including tandem-walk sway and SF-36 mental component; FGA improved from baseline in all groups.	In-person PT improved T25FW, tandem-walk width, MFIS and SF-36 physical/mental components; unsupervised HEP improved ABC, FGA and MSSE.	Pairwise comparisons showed no significant differences between TR and in-person PT for gait/balance outcomes; only selected QoL comparisons favoured in-person PT over control/TR.	Thirty participants were randomized; one PT participant dropped out due to MS relapse. No barriers to TR participation were identified.	Pilot feasibility evidence indicates that real-time TR can provide gait/balance outcomes broadly comparable to in-person PT in MS, but precision is limited by the small three-arm sample.
Fluet et al. 2024 [65]	Scaffolded game-based home rehabilitation versus algorithm-controlled game difficulty; post-intervention after 12 weeks	Training time/sessions; UEFMA; ARAT; SIS hand/ADL/participation; IMI	The scaffolded group trained independently at home with discrete levels of increasing difficulty; clinical motor outcomes improved, but not more than algorithm control.	The algorithm-controlled group performed the same activities with incremental, less visible difficulty modification and achieved similar adherence and motor outcomes.	No significant group-by-time interactions were detected. Across both groups, UEFMA improved by 5.85 points (95% CI 4.73 to 6.98), and 21 participants exceeded the 4.25-point MCID threshold.	There were five dropouts and no adverse events. Overall adherence was modest, and training time was not significantly correlated with clinical improvement.	Shows that sparsely supervised game-based home rehabilitation can yield meaningful upper-limb gains after stroke, but scaffolding alone did not add clear adherence or efficacy benefit.
Flynn et al. 2021 [66]	Home-based exercise monitored by telehealth versus continued centre-based exercise after an initial 5-week centre/self-management block; post-intervention at 10 weeks	Feasibility/adherence; Mini-BESTest; 10 m walk speed; NFOGQ	Home-based exercise with telehealth was feasible, with 84% adherence during Block 2 and only 6.4 therapist hours required (mean 10 min per participant).	Continued centre-based exercise had 93% adherence during Block 2 but required 32.5 therapist hours (98 min per participant).	No between-group differences were found for secondary clinical outcomes; preferred walking speed mean difference was −0.04 m/s (95% CI −0.12 to 0.05).	Participants found home exercise helpful and could follow the programme, although it was less satisfying than group-based exercise. One home participant withdrew because of an unrelated medical complication.	Supports feasibility and therapist-efficiency of telehealth-monitored home exercise in PD, but not powered to demonstrate superior motor effects.
Frevel et al. 2015 [67]	Internet-based home balance/postural-control training versus hippotherapy; post-intervention after 12 weeks	BBS; DGI; 2MWT; TUG; knee/trunk strength; FSS/MFIS; HAQUAMS	The e-Training group improved dynamic and static balance, with significant gains in DGI (*p* = 0.016) and BBS (*p* = 0.011).	Hippotherapy also improved DGI (*p* = 0.011) and BBS (*p* = 0.011), and showed significant improvements in fatigue and QoL outcomes.	No significant between-group difference was observed for balance improvement; strength changes were mild and non-significant in both groups.	Eighteen participants were enrolled and 16 completed the study; one e-Training participant could not work with the computer and one hippotherapy participant had an MS relapse.	Supports feasibility of Internet-based home balance training in MS, with balance effects comparable to hippotherapy but weaker fatigue/QoL signal.
Gandolfi et al. 2017 [68]	Home TeleWii VR balance telerehabilitation versus in-clinic sensory integration balance training; post-treatment at 7 weeks and 1-month follow-up	BBS; ABC; DGI; 10-MWT; PDQ-8; fall frequency; satisfaction; costs	TeleWii improved BBS by 3.74 points at post-treatment and 3.21 points at follow-up, with improvements over time across most balance/mobility outcomes.	SIBT improved BBS by 4.21 points at post-treatment and 4.05 points at follow-up; DGI gains reached the MCID at post-treatment.	Between-group differences were found for BBS (*p* = 0.04; post hoc post-treatment *p* = 0.02), and a Time × Group interaction was found for DGI (*p* = 0.04). Secondary outcome differences were otherwise not significant.	Seventy-six participants were randomized and 70 completed the study. No adverse events were reported. Total rehabilitation cost per patient was lower for TeleWii (EUR 383.55) than SIBT (EUR 602.10).	Supports home VR telerehabilitation as a feasible lower-cost alternative to in-clinic balance training for PD patients with caregiver support.
Garcia et al. 2022 [69]	Muvity telerehabilitation versus conventional self-directed rehabilitation in an 8-week/8-week crossover design; 6-month follow-up	FIM; VAS pain; BBS; SF-36 PCS; shoulder/elbow/waist ROM; usability	With Muvity, four of six completers maintained or improved FIM scores and five of six reported lower pain than after conventional treatment; most ROM averages were higher with the application except shoulder flexion.	Conventional self-directed treatment also produced some balance gains, and there were no clear trends for BBS or SF-36 PCS favouring Muvity.	Observed differences between treatments were not statistically significant, reflecting feasibility-level precision.	Ten participants entered the crossover study and six completed all phases. Participants generally found Muvity more motivating than conventional rehabilitation.	Preliminary feasibility evidence suggests Muvity may help maintain ADL performance without increasing pain, but larger powered studies are needed.
Ge et al. 2024 [70]	Telerehabilitation versus home physical therapy; post-intervention at 4 weeks	UPDRS3; BBS; TUG; FTSST; FOGQ; gait parameters; isokinetic strength; UPDRS2; PDQ-39; adherence/safety	TR improved UPDRS3 by 1.87 points and significantly improved BBS, TUG, FTSST, FOGQ, gait parameters, lower-limb strength, UPDRS2 and PDQ-39 over 4 weeks.	HPT also improved the same domains and showed larger gains in older participants.	In participants aged 70 years or older, UPDRS3 decreased more with HPT than TR (−3.38 vs. −1.45 points; *p* = 0.021); no significant between-group difference was seen in younger participants (*p* = 0.416). BBS, TUG, step velocity and extension average torque also favoured HPT.	Exercise completion was higher with HPT: 98% attended at least 50% of sessions versus 87.78% in TR (*p* = 0.04). No serious adverse events were reported; adverse events were fatigue, pain or dizziness.	Shows that TR is feasible and effective for mild-to-moderate PD, but therapist-delivered home physical therapy may improve compliance and outcomes in older patients.
Ginis et al. 2016 [71]	CuPiD smartphone/wearable-feedback gait training versus personalized gait advice; post-intervention after 6 weeks and 4-week follow-up	Single- and dual-task gait speed; Mini-BESTest; endurance; SF-36 physical health; feasibility/user acceptance	CuPiD participants improved single- and dual-task gait speed at post-test and follow-up; Mini-BESTest improved from 24.8 to 26.1 at post-test.	The active control group also improved gait speed after personalized gait advice, but SF-36 physical health deteriorated at follow-up (50.4 to 48.3).	Primary gait-speed outcomes improved in both groups without significant between-group differences. CuPiD showed greater Mini-BESTest improvement at post-test and maintained SF-36 physical health at follow-up.	The CuPiD system was well tolerated and rated user-friendly; 40 participants were randomized and 38 completed training.	Supports wearable/smartphone feedback as a feasible home gait-training adjunct in PD, with comparable gait gains and possible balance/QoL advantages.
Goffredo et al. 2023 [72]	Nonimmersive VR-based telerehabilitation versus at-home self-administered conventional motor activities; post-intervention after 30 sessions	Mini-BESTest; TUG; 6MWT; MDS-UPDRS III	The telerehabilitation group improved Mini-BESTest by 1.7 ± 0.33 points and showed significant gait/endurance gains.	The control group improved Mini-BESTest by 0.70 ± 0.34 points after structured self-administered exercises.	Mini-BESTest improved significantly more with telerehabilitation (TIME *p* < 0.001; Time × Group *p* = 0.029). Gait and endurance improved in the telerehabilitation group only, with significant within- and between-group differences.	Of 105 randomized participants, 97 were analyzed; dropout was due to drug changes, medical complications or discontinued treatment. The intervention was described as feasible and well tolerated.	Provides multicentre RCT evidence that nonimmersive VR telerehabilitation improves static/dynamic balance in PD more than self-administered home exercises.
Guo et al. 2023 [73]	Wearable remote rehabilitation training system plus routine PT/drug therapy versus routine OT plus routine PT/drug therapy; post-intervention after 3 weeks	FMA total; FMA upper extremity; FMA lower extremity; adverse events	The experimental system group improved total FMA by 17.56 points (SD 11.65; 95% CI 14.37 to 20.74), UE FMA by 11.28 points and LE FMA by 6.28 points.	The control group improved total FMA by 11.98 points (SD 8.46; 95% CI 9.69 to 14.27), UE FMA by 7.45 points and LE FMA by 4.53 points.	Between-group change favoured the remote rehabilitation system for total FMA (*p* = 0.005) and UE FMA (*p* = 0.01); LE FMA did not reach significance (*p* = 0.06).	Completion rates were 90% in the experimental group and 92% in controls. Adverse events were not significantly different (36.67% vs. 46.67%; *p* = 0.27) and were judged unrelated to the system.	Supports efficacy and safety of wearable human–computer-interaction training for stroke motor recovery, although the trial was conducted in rehabilitation institutions rather than fully unsupervised home settings.
Hartung et al. 2025 [74]	Internet-based exercise and physical-activity promotion versus waitlist/usual care; post-intervention at 12 weeks and follow-up at 24 weeks	Device-measured steps/day and MVPA; self-reported sport/exercise and leisure PA; PAHCO; T25FW; 2MWT; MSWS-12; fatigue; QoL	The intervention did not significantly change the primary outcome steps/day, but improved MVPA, sport/exercise, control competence, fatigue and physical QoL. Fatigue decreased from median 41.0 to 29.0 (*p* = 0.002; d = 0.68), and control competence increased from 39.9 to 51.8 (*p* = 0.001; d = 0.98).	Waitlist/usual care did not improve steps/day and showed no significant fatigue or physical QoL change; leisure/transportation PA decreased during the first 12 weeks.	Between-group changes were significant for MVPA, sport/exercise activity, control competence, fatigue and physical QoL, but not for steps/day, psychological QoL, depression, walking ability or self-concordance.	Adherence averaged 1.9 training sessions/week and declined over time; 65.5% completed all four therapist calls. Eleven adverse events occurred during the first 12 weeks, mostly falls, relapses, respiratory infections, orthopedic issues or medication side effects.	Relevant for physical-activity promotion rather than impairment-specific motor recovery; useful narrative evidence that Internet-based coaching can improve selected activity, fatigue and QoL outcomes in MS.
Hernandez et al. 2022 [75]	Home-based Jintronix VR serious-game telerehabilitation versus standardized GRASP home exercise programme; post-intervention at 4 weeks and 4-week follow-up	FMA-UE; SIS; MAL-14; active playing time; feasibility metrics	The VR group showed upper-limb improvement over time, with 9/26 participants reaching or exceeding the FMA-UE MCID. Among participants who actively engaged with the system for at least the recommended 400 min, 56% reached the MCID.	The GRASP home-programme group also improved over time; 5/25 participants reached or exceeded the FMA-UE MCID overall, with fewer responders than in the VR group.	No statistically significant between-group differences were found across clinical measures. A significant overall time effect was observed for FMA-UE (*p* = 0.045), especially from pre- to post-intervention (*p* = 0.03).	No adverse events, falls or dizziness episodes were reported. Two participants reported technology difficulties, mainly related to mouse control and navigation of the gaming interface.	Suggests that remotely monitored home VR training is feasible and may be comparable to evidence-based home exercise in chronic stroke; responder analyses suggest a dose-engagement signal rather than clear between-group superiority.
Hong et al. 2025 [76]	Telerehabilitation task-oriented training versus in-person task-oriented training and telerehabilitation neurofacilitation; post-intervention after 3 weeks	FMA-UE; WMFT-FAS; ARAT; IADL	Telerehabilitation task-oriented training improved FMA-UE by a mean of 9.4 points and improved WMFT, ARAT and IADL scores after treatment.	In-person task-oriented training improved FMA-UE by 6.4 points, while tele-neurofacilitation improved FMA-UE by 6.7 points; all three groups improved significantly after treatment.	Telerehabilitation task-oriented training was non-inferior to in-person task-oriented training for FMA-UE change (mean difference 3.29; 95% CI −0.81 to 7.39), with the upper CI below the 12.4-point non-inferiority margin.	Seventy-nine participants completed the trial (tele-rehab n = 23; in-person n = 28; tele-control n = 28); no adverse reactions were reported. Disease duration differed across groups and was adjusted using ANCOVA.	Supports non-inferiority of remotely guided task-oriented upper-limb training after stroke, but the retrospective cohort design and baseline duration imbalance require cautious interpretation.
Huijgen et al. 2008 [77]	HCAD home telerehabilitation for arm/hand function versus usual care/general exercises; outcomes after 1 month of HCAD or usual care	ARAT; NHPT; user satisfaction	HCAD users generally maintained or improved arm/hand function; the MS subgroup showed a tendency toward NHPT improvement, and MS ARAT scores were clinically equivalent to usual care.	Usual care and generic exercises also maintained or slightly improved arm/hand function in patients with stroke, TBI or MS.	No significant between-group differences were found for ARAT or NHPT. Equivalence conclusions varied by diagnosis: MS ARAT and TBI NHPT remained within prespecified equality bounds, while stroke estimates had wide confidence intervals.	Eighty-one participants were randomized and 70 were analyzed. HCAD was used for a mean of 19 days, about 30 min/day, producing a monthly training exposure similar to usual care (9.5 vs. 9 h); patients and therapists were generally satisfied.	Provides early multicentre feasibility evidence for home arm/hand telerehabilitation across neurological diagnoses, with maintenance/comparable effects rather than demonstrated superiority.
Jarbandhan et al. 2022 [78]	Home-based semi-supervised physiotherapy with tele-supervision versus usual care; post-intervention at 8 weeks	6MWT/6MWD; BBS; DASH; paretic/non-paretic handgrip strength; satisfaction and safety	The intervention group improved 6MWD by 57.2 ± 67.3 m and DASH score by 9.8 ± 15.2 points, indicating better walking tolerance and upper-extremity function.	The usual-care group showed minimal change in 6MWD (0.6 ± 29.2 m) and no comparable DASH improvement; BBS showed a ceiling effect and handgrip strength did not significantly change.	Significant group-by-time effects favoured the intervention for 6MWD (F(1,28) = 6.36; *p* = 0.018) and DASH (F(1,28) = 4.54; *p* = 0.042).	Fourteen of 20 intervention participants completed the programme (70%). No physical-activity-related adverse events were reported, and participants were generally satisfied; adherence was affected by rainy-season logistics, medical status and motivation.	Supports feasibility and preliminary mobility/upper-limb benefits of semi-supervised home physiotherapy with tele-coaching in a low-resource stroke setting; larger powered trials are needed.
Johnson et al. 2020 [79]	STRIVE community-based virtual therapy versus usual care; post-intervention after 8 weeks	FMUE; ARAT; BBT; MAS; MAL-28; EQ-5D-5L	The virtual-therapy group showed greater FMUE improvement than usual care, increased MAL-28 amount of use and quality of movement, and reduced shoulder and elbow spasticity.	Usual-care participants maintained their usual activities and did not show significant FMUE improvement over time.	Between-group differences favoured virtual therapy for FMUE (group main effect F = 5.37; *p* = 0.02; d = 0.41), but not ARAT. Post-intervention MAL-28 quality of movement also favoured virtual therapy (*p* = 0.02).	Two participants dropped out of the virtual-therapy group; the remaining 28 participants completed all 16 sessions, averaging 43.4 ± 5.4 min/session. No adverse events were reported.	Supports community-delivered, remotely monitored virtual therapy as a feasible option for chronic stroke upper-limb rehabilitation, with motor-impairment gains exceeding usual care but limited functional superiority on ARAT.
Johnson et al. 2024 [80]	Clinic plus telerehabilitation, telerehabilitation-only and usual care physical therapy; discharge after 4-week therapy episode	TUG; TUG-cognitive; 5STS; 10 m walk test; 6MWT; Mini-BESTest; ABC; PDQ-39; feasibility/satisfaction	Clinic plus TR improved Mini-BESTest from baseline to discharge; TR-only showed no clear within-group clinical superiority in this small pilot sample.	Usual care improved 5STS time and Mini-BESTest score; final clinical outcomes did not differ across the three study groups.	No statistically significant between-group differences were observed in clinical outcomes at discharge; a pairwise ABC difference favoured usual care over clinic plus TR but baseline disease-severity differences limit interpretation.	Of 389 screened patients, 20 were enrolled and 19 completed the study. One TR-only participant was withdrawn after a non-study-related injurious fall; patients and therapists generally rated care delivery as good or very good.	Provides pilot feasibility evidence that telerehabilitation can be acceptable in early-to-moderate PD, but the sample is underpowered for comparative effectiveness conclusions.
Kintrilis et al. 2024 [81]	Teleconference-delivered resistance training versus in-person resistance training and usual care; reassessment 3 months after discharge	VAS health score; TUG; BBS; Chair Stand Test; 6MWT; A-VO2	The teleconference resistance-training group showed significant improvement in VAS health score (*p* = 0.044), while functional-test changes were not consistently significant within the teleconference subgroup.	In-person resistance training produced similar post-intervention results to teleconference delivery. The usual-care group also improved on selected functional tests, particularly TUG and 6MWT.	No significant differences were observed between in-person and teleconference delivery across VAS, functional tests or A-VO2. Comparisons with usual care were mixed and did not establish superiority of resistance training.	Both resistance-training groups trained 3 times/week for 12 weeks using elastic bands; relatives/caregivers assisted with videoconference setup when needed. Adverse-event reporting was not emphasized in the results.	Suggests teleconference resistance training may be deliverable with effects similar to in-person delivery after stroke, but efficacy versus usual care should be interpreted cautiously because control-group improvements were substantial.
Kowalczewski et al. 2011 [82]	ReJoyce FES-assisted in-home teletherapy versus conventional in-home tele-exercise therapy; crossover, post-treatment after 6 weeks	ARAT; RAHFT; grasp and pinch force	ReJoyce FES-assisted teletherapy improved ARAT scores by 13.0% ± 9.8% and RAHFT scores by 16.9% ± 8.6%.	Conventional tele-exercise therapy improved ARAT by 4.0% ± 9.6% and RAHFT by 3.3% ± 10.2%.	ReJoyce produced significantly greater improvement than conventional tele-exercise for ARAT (F = 10.6; *p* < 0.01) and RAHFT (F = 20.4; *p* < 0.01).	Participants trained 1 h/day, 5 days/week for 6 weeks under Internet supervision. Thirteen participants entered the study; 18 hands were analyzed, and no dropouts occurred at 30-week follow-up.	Supports feasibility and possible superiority of FES-assisted, ADL-like tele-supervised workstation training for tetraplegic hand function in selected SCI participants.
Lee et al. 2022 [83]	Telerehabilitation dance therapy plus conventional physical therapy versus conventional physical therapy; post-intervention after 3 weeks	TIS; BBS; TUG; FAC; K-MBI; EQ-5D	The remote dance-therapy group significantly improved TIS score (*p* = 0.017), and also showed improvements in K-MBI (*p* = 0.004) and TUG (*p* = 0.033).	The control group received conventional physical therapy over the same period and served as the active comparator for non-inferiority testing.	TIS results demonstrated non-inferiority of dance telerehabilitation versus conventional treatment within the predefined 3.5-point margin (difference −0.86; 95% CI −2.21 to 0.50).	The analyzable pilot sample was small after dropouts. No adverse events were observed in either group; safety was supported by obstacle removal, caregiver participation and researcher monitoring.	Suggests remotely delivered dance therapy may provide trunk-control effects comparable to conventional rehabilitation after stroke, but findings remain preliminary because of the small pilot sample.
Lee et al. 2025 [84]	Inpatient telerehabilitation occupational therapy versus bedside occupational therapy; 10 additional 30 min sessions during hospitalization	MBI; PASS; FAC; PHQ-9; RPE; satisfaction	The telerehabilitation group significantly improved MBI and PASS scores (both *p* < 0.01), and FAC also improved significantly (*p* = 0.01).	The bedside-therapy control group significantly improved MBI (*p* < 0.01), PASS (*p* = 0.01) and PHQ-9 (*p* < 0.01).	No significant between-group differences were detected for the outcome measures, indicating no clear efficacy advantage of inpatient TR over bedside therapy in this pilot trial.	Twenty-four inpatients were randomized and 22 were included in the analysis. No falls or adverse effects were observed during the study; the TR group used a laptop, therapy objects and Google Meet with caregiver/research-assistant setup support.	Supports inpatient telerehabilitation as a feasible substitute or adjunct when in-person therapy access is constrained, but effectiveness remains preliminary and may be influenced by natural recovery.
Lin et al. 2014 [85]	Multi-user bidirectional telerehabilitation versus conventional group balance therapy in long-term care facilities; post-intervention after 4 weeks	BBS; BI total, self-care and mobility subscores; participant satisfaction	The telerehabilitation group significantly improved BBS and BI total/self-care scores after training.	The conventional therapy group showed similar significant improvements in BBS and BI total/self-care scores.	No significant between-group differences were demonstrated for balance, functional activity or satisfaction. BI mobility did not change significantly.	Twenty-four participants were randomized; one telerehabilitation participant dropped out because of unwillingness to continue. Vital signs were monitored remotely, and a volunteer/nonmedical assistant supported safety at the patient end.	Supports feasibility of bidirectional multi-user telerehabilitation for chronic stroke residents in long-term care facilities, with effects comparable to conventional balance therapy.
Llorens et al. 2015 [86]	Home-based VR telerehabilitation versus in-clinic VR balance training; post-treatment at 8 weeks and 12-week follow-up	BBS; POMA-B; POMA-G; BBA; SUS; IMI; cost	Home-based VR telerehabilitation improved BBS, POMA-B, POMA-G and BBA from baseline to post-treatment, and gains were broadly comparable to in-clinic delivery.	The in-clinic VR group also showed significant improvement on the same balance and mobility measures.	No significant between-group differences were found for any balance scale or feedback questionnaire; significant time effects were observed for BBS (partial eta squared = 0.68; *p* = 0.001), POMA-B (*p* = 0.006) and POMA-G (*p* = 0.001).	Thirty chronic stroke participants were analyzed. Both groups considered the VR system usable and motivating; home-based delivery reduced costs by $654.72 per person compared with in-clinic delivery.	Provides RCT evidence that VR-based home telerehabilitation can achieve balance recovery similar to in-clinic VR training after stroke, with potential cost savings.
Masbernat-Almenara et al. 2026 [87]	Mixed synchronous/asynchronous core-stability telerehabilitation versus asynchronous telerehabilitation; post-intervention at 7 weeks and follow-up at 12 weeks	S-TIS 2.0; SARA sitting/standing/walking and total score; 4MWT; 30 s sit-to-stand; TUG; ABC; EARS	The mixed-format group showed significant short-term improvement in gait performance at 7 weeks (*p* = 0.002), but this was not maintained at follow-up. Balance confidence unexpectedly declined in the mixed-format group.	The asynchronous group received the same core-stability exercise programme with weekly monitoring calls; clinical effects were generally smaller or less distinct than mixed delivery.	A significant time-by-group interaction favoured mixed delivery for short-term gait performance (*p* = 0.003). No other clinical outcome reached statistical significance.	Twelve participants completed the study with 100% retention. Programme delivery was described as feasible and safe; asynchronous diary adherence was 80%, while mixed-group platform logging became incomplete after the first weeks.	Suggests home core-stability telerehabilitation is feasible in hereditary ataxia and that mixed supervision may transiently improve gait, but evidence is pilot-level and adherence-monitoring methods need refinement.
Mulder et al. 2024 [88]	Caregiver-mediated exercises with telerehabilitation plus usual care versus usual care alone; post-intervention at 8 weeks and 6-month follow-up	SIS mobility; RMI; FAC; 6MWD; 5 m walking speed; Motricity Index leg; BBS; NEADL; mRS; psychosocial and transition outcomes	The Armed4Stroke group achieved the intended caregiver-mediated practice contrast but did not show a significant advantage on SIS mobility after 8 weeks.	Usual care alone produced similar self-reported mobility outcomes; self-reported muscle strength at 6 months favoured controls.	No significant difference was found for the primary outcome SIS mobility at 8 weeks (B = 0.8; 95% CI −6.8 to 8.5; *p* = 0.826). Significant secondary benefits favoured the intervention for caregiver QoL and depression post-intervention and leisure activities at 6 months.	Forty-one dyads were randomized and 37 analyzed. Caregiver-mediated exercise minutes were higher with Armed4Stroke (median 1260 vs. 435 min; *p* = 0.006); falls were similar (4 vs. 5). Recruitment stopped after futility analysis.	Confirms limited added value for self-reported mobility but suggests caregiver-centred psychosocial benefits; useful for implementation and caregiver-mediated telerehabilitation synthesis rather than primary motor efficacy pooling.
Nuic et al. 2024 [89]	Active full-body home exergaming versus keyboard-controlled gaming; post-training after 18 sessions over 6–9 weeks and optional open-label follow-up	SWST; MDS-UPDRS I-IV and axial score; GABS-B; Tinetti; NFOGQ; ABC; gait parameters; falls/adverse events	Active exergaming improved SWST duration numerically (−3.71 ± 18.06 s) and reduced clinical severity of gait/balance disorders, with 32% classified as responders.	Keyboard-controlled gaming produced a smaller SWST change (−0.71 ± 3.41 s), and 8% were classified as responders.	The primary SWST change did not differ significantly between groups (*p* = 0.61), but responder rate favoured active training (32% vs. 8%; *p* = 0.03) and gait/balance severity improved more after active training (*p* = 0.0082).	Fifty participants were randomized. Home-based training produced no serious adverse events; the first sessions were supported before independent home use, and the system automatically recorded adherence and success metrics.	Shows safe home implementation of tailored exergaming for dopa-resistant gait/balance problems in PD, with secondary responder and gait/balance signals despite a null primary endpoint.
Ortiz-Gutiérrez et al. 2013 [90]	Kinect-based home telerehabilitation versus conventional physiotherapy; post-treatment after 10 weeks	Computerized dynamic posturography/SOT; composite equilibrium score; sensory ratios	The experimental group improved overall balance and showed significant gains in sensory organization, particularly visual preference and vestibular contribution measures.	The conventional physiotherapy group also improved general balance after the 10-week programme.	Both interventions improved postural control; the telerehabilitation programme showed specific advantages in optimizing sensory information integration rather than a uniform superiority across all SOT domains.	Forty-seven of 50 participants completed the study; home sessions were monitored by videoconference and another person was recommended to be present for safety.	Supports Kinect-based VR telerehabilitation as a feasible alternative for balance/postural-control training in MS when conventional therapy access is limited; synthesis should remain cautious because allocation was partly pragmatic.
Pagliari et al. 2024 [91]	Home VRRS telerehabilitation versus home conventional rehabilitation/usual care; post-treatment at 6 weeks	MSQOL-54; Mini-BESTest; BBT; 9-HPT; adherence	TR improved the physical domain of QoL in a greater proportion of participants, with 63.3% showing improvement in MSQOL-54 physical domain.	Usual care also delivered a same-dose conventional home motor/cognitive programme, but showed smaller gains in balance-related outcomes.	Time-by-group effects favoured TR for MSQOL-54 physical domain (*p* = 0.045), Mini-BESTest balance domain (*p* = 0.014), anticipatory postural control (*p* = 0.024) and dynamic walking (*p* = 0.020).	Adherence was higher in TR than usual care (86.7% vs. 80.0%); SUS usability score was above cut-off; five participants dropped out in each group.	Provides multicentre RCT evidence that asynchronous home VRRS can improve patient-relevant physical QoL and balance domains in pwMS; candidate for quantitative synthesis where outcomes align.
Paul et al. 2014 [92]	Web-based physiotherapy versus usual care; post-intervention at 12 weeks/week 13	T25FW; BBS; TUG; MSIS; Leeds MS QoL; HADS	The web-based group showed a non-significant increase in gait speed on the T25FW and a significant improvement in the physical subscale of the MSIS.	Usual care consisted of general exercise advice and signposting; one control participant withdrew by week 13.	No significant between-group effect was detected for the primary T25FW outcome (*p* = 0.170); MSIS physical subscale favoured the intervention (*p* = 0.048).	Participants logged in an average of 1.3 times/week, declining from 2.1 to 0.9; three adverse events occurred but were deemed unrelated to the intervention; participants rated the website good/excellent.	Useful pilot evidence for acceptability and potential symptom impact of web-based MS physiotherapy, but motor efficacy should be interpreted as preliminary.
Paul et al. 2019 [93]	Six-month web-based physiotherapy versus printed home exercise programme; outcomes at 3, 6 and 9 months	2MWT; T25FW; TUG; BBS; steps/day; MSIS; EQ-5D	The web-based group maintained walking and balance outcomes over 6 months without clear superiority over the active comparator.	The printed exercise-sheet group showed similar stability; EQ-5D decreased at 6 months in the active-comparator group.	No significant between-group changes were found for most outcomes, including 2MWT; the study supported feasibility rather than efficacy.	Adherence ranged 40–63% in the web group and 53–71% in the comparator; no intervention-related adverse events were recorded; the definitive trial sample estimate was about 160 participants.	Supports feasibility and acceptability of long-duration web-based physiotherapy in MS, but should be used as feasibility/sensitivity evidence rather than pooled superiority evidence.
Pavan et al. 2024 [94]	Robotic versus non-robotic home telerehabilitation; post-intervention after 20 sessions/5 weeks	WHODAS 2.0; FMA-UE; BBT; Trail Making Test-A; QoL and clinical-functional status	Both telerehabilitation models produced significant within-group improvement across analyzed domains.	The robotic group also improved after home telerehabilitation using the ICONE end-effector, but with smaller mean WHODAS improvement than the non-robotic group.	Between-group patterns favoured the non-robotic group for perceived disability and selected motor/cognitive outcomes; WHODAS 2.0 decreased by 6.09 ± 2.62% in NRG versus 0.76 ± 2.21% in RG, with greater NRG improvement in FMA-UE motor function, BBT and TMT-A.	All 30 randomized participants completed baseline and post-intervention evaluations; the programme relied on one weekly synchronous session and three autonomous sessions/week.	Relevant to delivery-model selection within telerehabilitation after sub-acute stroke; not a primary telerehabilitation-versus-usual-care efficacy comparison.
Petracca et al. 2024 [95]	Telerehabilitation versus onsite rehabilitation; post-intervention after 6 weeks	MSQoL-54; FSS; BBS; SDMT	TR participants improved across QoL, fatigue, balance and cognition domains after the 6-week programme.	Onsite rehabilitation participants also improved significantly across the same domains.	Across the entire cohort, QoL improved (*p* = 0.005), fatigue and balance improved (both *p* < 0.001), and cognition improved (*p* = 0.003); TR and onsite rehabilitation showed comparable clinically meaningful improvements.	Fifty-one of 61 recruited pwMS completed the study; TR discontinuations included Internet connectivity issues and non-compliance; sessions were one-to-one and remotely supervised.	Supports the clinical comparability and feasibility of synchronous TR and onsite rehabilitation in moderately disabled pwMS, but non-random allocation requires cautious narrative interpretation.
Picelli et al. 2026 [96]	Synchronous multidomain telerehabilitation versus conventional outpatient therapy; post-treatment at 4 weeks and 1-month follow-up	FMA-UE motor; FMA-LE motor; IT-AAT naming/written language/comprehension; OCS orientation/memory; BI	The telerehabilitation group improved on upper- and lower-extremity FMA scores and maintained comparable multidomain trajectories through follow-up.	The conventional outpatient group showed similar motor, language, cognitive and ADL improvements.	Primary non-inferiority was met for FMA-UE: adjusted between-group change was 2.23 points (95% CI −3.63 to 8.09), with the lower CI above the prespecified −5.2 margin; ITT sensitivity was consistent.	All participants completed all 20 sessions and follow-up assessments; no adverse events were reported; fidelity was tracked using therapist logs and VRRS platform logs.	Strong non-inferiority evidence for short-term multidomain synchronous telerehabilitation after sub-acute stroke; candidate for primary non-inferiority/delivery-model synthesis.
Piron et al. 2009 [97]	Home VRRS telerehabilitation versus traditional upper-limb physical therapy; post-treatment at 4 weeks and 1-month follow-up	Fugl-Meyer UE; ABILHAND; Ashworth	The telerehabilitation group significantly improved after treatment and showed greater Fugl-Meyer UE gains than the control group.	The traditional physical-therapy group also improved significantly after treatment.	Both groups improved, but between-group comparison favoured telerehabilitation for Fugl-Meyer UE motor performance; benefits were substantially maintained at 1-month follow-up.	All 36 participants completed the study; patients did not have problems handling VRRS.net, although intermittent broadband quality reductions and connection interruptions were reported.	Early RCT evidence suggests VR-based teletherapy can improve post-stroke upper-limb motor performance; synthesis should note the small sample and older technology context.
Prukviwat et al. 2026 [98]	Motion-sensor telerehabilitation versus hospital-based rehabilitation; post-intervention after 16 sessions	BBS; Chula PMS; gait speed; step length	The tele group significantly improved BBS (+3.50, *p* < 0.001) and Chula PMS (+3.45, *p* < 0.001).	The hospital group also significantly improved BBS (+4.35, *p* < 0.001) and Chula PMS (+5.70, *p* < 0.001).	No significant between-group differences were found for BBS (mean difference −0.85, *p* = 0.454) or Chula PMS (mean difference −2.25, *p* = 0.086).	Attendance exceeded 90% in both groups; feasibility was high; BBS improvement did not reach the minimal clinically important difference.	Suggests motion-sensor TR may be a feasible alternative to hospital rehabilitation for PD balance/mobility, but non-randomized preference allocation and sub-MCID balance change require cautious synthesis.
Pastana Ramos et al. 2023 [99]	Individual telerehabilitation versus booklet-based exercise; post-intervention at 4 weeks and 4-week washout follow-up	TUG; 5STS; ABC; MDS-UPDRS III; PDQ-8	The telerehabilitation programme had high adherence and reduced TUG completion time after the intervention.	The booklet group also reduced TUG time after the same 4-week exercise period.	Both telerehabilitation and booklet orientation improved dynamic mobility as measured by TUG; the small phase 2 sample limits definitive between-group efficacy claims.	Minimal adverse effects were reported; sessions were caregiver-supported and weekly phone calls checked feedback/adverse effects in both groups.	Feasibility evidence supports remote individual rehabilitation for mild PD in an underserved Amazonian context; use as feasibility/sensitivity evidence.
Salgueiro et al. 2022a [100]	App-guided core-stability exercises plus conventional physiotherapy versus conventional physiotherapy alone; 12-week post-intervention	S-TIS 2.0; S-FIST; S-PASS; BBS; G-Walk gait parameters	The experimental group improved trunk performance, with a 2.76-point S-TIS 2.0 gain (*p* = 0.001), and showed small improvements in balance/gait measures.	The control group continued conventional physiotherapy and showed less marked change in trunk outcomes.	The clearest effect was within-group trunk improvement in the app group; between-group evidence for balance and gait was small/preliminary.	Adherence to app use was low; the study occurred during COVID-19 restrictions and used phone/video-call assessment when needed.	Supports app-guided CSE as a potentially useful adjunct for trunk control in chronic stroke, but adherence and preliminary sample size limit inference.
Salgueiro et al. 2022b [101]	Post-discharge app-guided core-stability exercises plus usual care versus usual care; 3-month follow-up	S-TIS 2.0; S-FIST; BBS; S-PASS; falls; BBA stepping; G-Walk	The AppG showed greater numerical improvement in sitting balance, standing balance and gait than usual care; S-TIS 2.0 improved by 0.95 points.	The usual-care group also improved slightly on sitting-balance measures; S-TIS 2.0 improved by 0.65 points.	Between-group differences did not reach statistical significance; direction of effect generally favoured AppG but remained exploratory.	Follow-up data were available for 13 AppG and 25 control participants; app adherence remained a key feasibility challenge.	Provides post-discharge continuity evidence for app-guided CSE after sub-acute stroke, but should be treated as controlled extension/narrative evidence.
Saygili et al. 2024 [102]	Tele-CIMT plus home exercise versus home exercise alone; post-intervention after 3 weeks	STREAM; FM-UE; WMFT; 9-HPT; grip/pinch strength; MAL-28; FIM	Tele-CIMT produced significant gains in upper-limb motor function, functional ability, grip/pinch strength, affected-arm use and ADL independence.	The control group performed the same basic home exercise programme without m-CIMT videoconference sessions.	Significant time-by-group interactions favoured Tele-CIMT for STREAM, FM-UE, WMFT, 9-HPT, grip/pinch strength, MAL-28 and FIM.	Tele-CIMT participants attended all sessions; control participants completed home exercises; no technical problems prevented session delivery.	Provides small RCT evidence that m-CIMT-based telerehabilitation can enhance upper-limb recovery beyond home exercise alone; candidate for sensitivity/meta-analysis if outcome/time point compatible.
Saywell et al. 2021 [103]	ACTIV augmented community telerehabilitation versus usual care; 6-month post-intervention and 12-month follow-up	SIS physical subcomponent; grip strength; Step Test; SSEQ; EQ-5D VAS	ACTIV showed a favourable but non-significant ITT effect on physical function at 6 months.	Usual care after discharge typically involved no further formal rehabilitation, although additional care was not restricted.	ITT difference in SIS physical function was 4.51 (*p* = 0.07); planned per-protocol analysis favoured ACTIV by 5.28 (*p* = 0.04), but improvements were not maintained at 12 months.	Monthly adverse-event calls were conducted; the programme used 4 home visits, 5 phone calls and text reminders over 6 months.	Suggests possible benefit among adherent participants, but primary ITT finding was not statistically significant and durability was limited.
Sheehy et al. 2025 [104]	Home NIVRT versus iPad active control; post-intervention after 6 weeks	Feasibility; BBS; TUG variants; FTSTS; CB&M; SIS; CIQ	NIVRT participants completed an average of 26 sessions and 700 min; most learned and progressed, enjoyed the intervention and perceived benefit.	iPad participants completed an average of 33 sessions and 1241 min of cognitive/fine-motor app use.	Most physical assessments improved over time in both groups, but the feasibility trial was not designed or powered to establish efficacy.	All 20 participants completed the study; no serious adverse events occurred; space constraints were observed in 5 homes.	Supports safety and feasibility of asynchronous home NIVRT for standing balance and gait after discharge; efficacy requires a larger definitive RCT.
Simpson et al. 2025 [105]	Virtual Arm Boot Camp versus waitlist/usual care; primary endpoint at 4 weeks	TENZR daily reach-to-grasp counts; REACH; ArmCAM; upper-limb use/QoL outcomes	V-ABC increased real-world affected upper-limb activity measured objectively by reach-to-grasp counts.	Waitlist/usual-care participants could receive limited usual therapy but did not receive V-ABC during the initial comparison period.	V-ABC produced higher average daily reach-to-grasp counts than control at 4 weeks (mean difference 368 counts/day; 95% CI 6 to 730; *p* = 0.046).	The intervention combined home exercise, wearable feedback and six virtual therapist sessions; outcome wear-time criteria were prospectively defined.	Provides RCT evidence that a virtually delivered wearable-feedback programme can increase real-world upper-limb activity after stroke; relevant to activity/adherence synthesis.
Skelly et al. 2026 [106]	Individualized real-time physiotherapy telerehabilitation versus usual exercise advice; follow-up at 3 and 6 months	UPDRS; weekly step count; PDQ-39; Fitbit activity; SLST; FTSTS; falls	The TR group showed favourable preliminary changes in motor symptoms and weekly step count at 6 months.	The usual-care group received physician/nurse advice about exercise and physical activity and had less favourable exploratory motor/activity changes.	Median UPDRS change at 6 months was −3.5 in TR versus +7 in usual care (Cohen d = −0.537); weekly step count changed by +4215 versus −2185 (Cohen d = 0.198).	Feasibility progression criteria were met: 74% eligibility among screened, 90% study-instrument completion, and 80% completion of intervention/assessments; participants found the intervention acceptable.	Supports feasibility of a definitive RCT of individualized TR in early PD; preliminary effects are hypothesis-generating and not confirmatory.
Song et al. 2018 [107]	Home-based exergame step training versus usual care/no study intervention; post-intervention at 12 weeks and falls follow-up to 6 months	Choice stepping reaction time; Functional Gait Assessment; TUG; gait adaptability; falls; perceived mobility/balance	Intervention participants completed a mean of 31/36 prescribed sessions (86%). Primary stepping and FGA outcomes did not improve versus control, but participants reported perceived mobility improvement.	Control participants continued usual healthcare and showed no structured exergame exposure; TUG results unexpectedly favoured controls.	No between-group differences were detected for primary outcomes or most secondary outcomes. TUG favoured control (*p* = 0.02), while perceived mobility favoured intervention (between-group difference 0.9 on an 11-point scale, *p* = 0.03). Subgroup interactions suggested possible benefit in lower disease severity and possible adverse functional signals in higher severity.	Six intervention participants discontinued; one non-injurious fall occurred during exergame training and several pre-existing pain problems were exacerbated but not attributed to training.	Overall efficacy was neutral; home exergame stepping may be acceptable and potentially useful for selected lower-severity PD participants, but should be synthesized cautiously.
Standen et al. 2017 [108]	Low-cost home-based virtual-reality arm/hand rehabilitation versus usual care; midpoint at 4 weeks and final assessment at 8 weeks	WMFT; 9-HPT; MAL amount/quality of movement; NEADL	Participants allocated to home VR used a virtual glove system for arm/hand games targeting reaching, grasp/release and forearm rotation; some favourable changes were observed in grip and MAL outcomes.	Usual-care participants received no study intervention beyond outcome assessments.	The feasibility trial found significantly greater change from baseline in the intervention group for midpoint Wolf grip strength and two final Motor Activity Log subscales; other outcomes were not reported as definitively superior.	Recruitment was low, only 18/27 randomized participants completed final outcomes, and training/support demands were substantial (median 230 min support per intervention patient).	Feasibility evidence suggests possible upper-limb activity benefit, but definitive efficacy requires a larger trial with enhanced recruitment and support planning.
Sun et al. 2025 [109]	Post-discharge telerehabilitation versus standard post-discharge care; post-intervention at 12 weeks	Barthel Index; mRS; HAMD; WHOQOL-BREF	The telerehabilitation group improved markedly in ADL, disability, mood and QoL; Barthel Index increased from 65.4 ± 12.3 to 88.7 ± 9.6.	Standard care improved Barthel Index from 65.6 ± 12.1 to 74.9 ± 13.2 with manual-based rehabilitation and biweekly outpatient follow-up.	Between-group results favoured telerehabilitation for Barthel Index (*p* < 0.001), mRS 0–2 status (75% vs. 62%; *p* = 0.003), HAMD reduction (*p* < 0.001), and all WHOQOL-BREF domains (*p* < 0.05).	The programme included personalized plans, platform training, weekly video consultations, health education and mental-health support; adverse-event detail was limited in the available report.	Supports post-discharge telerehabilitation after AIS for ADL, disability and QoL, but synthesis should consider broad multimodal content and the need to verify reporting quality.
Swarnakar et al. 2023 [110]	Biweekly telerehabilitation plus home PT/OT practice versus standard usual care; outcomes at 4 and 8 weeks	SCIM III total/domains; Coronavirus Anxiety Scale	The telerehabilitation group improved functional independence, particularly self-care and mobility domains, and coronavirus-related anxiety.	The control group continued previously advised standard usual care after outpatient/inpatient rehabilitation.	Between-group analysis favoured telerehabilitation for SCIM III self-care (*p* = 0.03) and mobility (*p* = 0.01); CAS also improved in the intervention group versus control.	All 30 participants completed the 8-week trial; the intervention was described as safe and feasible in SCI during pandemic-related access restrictions.	Provides small RCT evidence that telephone-based telerehabilitation can support self-care and mobility in SCI, but longer and larger studies are needed.
Thomas et al. 2017 [111]	Immediate Mii-vitaliSe plus usual care versus 6-month delayed waitlist/usual care; feasibility outcomes at 6 and 12 months	Physical activity; balance/walking/mobility activities and hand dexterity; QoL; mood; fatigue; self-efficacy; adherence	Participants used the home Wii programme on average twice weekly for 27 min/day during the initial 6-month intervention period and qualitative feedback indicated acceptability.	Delayed-group participants received usual MS service care and were asked to refrain from Wii use during the first 6 months.	The pilot was not powered for clinical efficacy; its primary contribution was feasibility, acceptability, adherence and cost information rather than definitive motor effects.	Recruitment rate was 31%; outcome data were available for 97% at 6 months and 93% at 12 months; no serious adverse events were reported. Mean delivery cost was GBP 684/person.	Supports feasibility of a definitive trial of physiotherapist-supported home exergaming in MS; use as feasibility/narrative evidence rather than motor-efficacy pooling.
Toh et al. 2025a [112]	Smart Reminder wearable telerehabilitation versus sham device plus pictorial handouts; post-intervention at 4 weeks and follow-up at 8 weeks	FMA-UE; ARAT; MAL; AROM; muscle strength; accelerometer movement counts; adherence	The Smart Reminder group showed greater FMA-UE improvement and very high adherence to training.	The sham group performed comparable handout-guided exercises and wore an accelerometer without active app feedback/reminders.	FMA-UE change favoured Smart Reminder (mean difference 2.05; *p* = 0.036). Training adherence was higher with Smart Reminder (97.0% vs. 82.3%; *p* = 0.038), but daily affected-arm use was not significantly enhanced.	Two sham-group participants dropped out; the intervention combined remote data review with weekly therapist consultations.	Suggests wearable-assisted telerehabilitation may modestly improve upper-limb impairment and adherence after stroke, while transfer to daily arm use remains uncertain.
Toh et al. 2025b [113]	Smart Reminder wearable-smartphone telerehabilitation versus conventional handout therapy in a two-period crossover design; each treatment lasted 4 weeks	FMA-UE; ARAT; shoulder/elbow/forearm AROM; MAL; adherence	Wearable telerehabilitation significantly improved hemiplegic shoulder AROM, including flexion (+8.9 ± 12.7; *p* = 0.022) and abduction (+12.2 ± 14.3; *p* = 0.018).	Conventional therapy used similar exercises presented as pictorial handouts with weekly consultation; no significant upper-limb improvements were detected.	Between-treatment comparison indicated greater shoulder AROM improvement after Smart Reminder training, while FMA-UE, ARAT, MAL and other ROM outcomes were not significantly different.	Three participants dropped out for medical reasons unrelated to the study; no adverse intervention-related effects were reported. Compliance was 78.25% for Smart Reminder and 70.5% for conventional therapy.	Feasibility evidence supports wearable-smartphone delivery for shoulder ROM, but sample size and crossover attrition limit efficacy conclusions.
Uswatte et al. 2021 [114]	In-home Tele-AutoCITE CIMT versus in-laboratory in-person CIMT; post-treatment after 35 h and 1-year follow-up	MAL Arm Use scale; WMFT; satisfaction and perceived difficulty	Tele-AutoCITE produced very large immediate improvements in real-world arm use comparable to in-lab CIMT.	In-lab CIMT delivered the same amount, intensity and transfer package in-person in the laboratory.	Across both groups, MAL Arm Use improved by 2.5 points immediately after treatment (*p* < 0.001; d = 3.1) and by 1.8 points at 1 year (*p* < 0.001; d = 2.0). Telehealth outcomes were not inferior to in-lab outcomes post-treatment; long-term non-inferiority could not be ruled in because of follow-up dropout.	Ten participants per group completed treatment; satisfaction and perceived difficulty were similar between delivery modes. No adverse events were related to study participation.	Strong proof-of-concept evidence that automated in-home CIMT can deliver immediate benefits comparable to in-lab CIMT, with long-term precision limited by attrition.
Vasconcellos et al. 2023 [115]	Home-based trunk exercise telerehabilitation versus home-based upper/lower-limb exercise telerehabilitation; post-training at 3 weeks and 4-week follow-up	COP stabilometry; gait speed; hip/knee/ankle ROM; MDS-UPDRS III	The trunk-exercise group did not show significant improvements in static balance or gait variables compared with baseline or control.	The control group performed global upper- and lower-limb exercises at home with the same telerehabilitation monitoring structure.	No significant time-by-group interactions were found for COP displacement/velocity/range, gait speed, hip extension, or knee/ankle ROM; no intragroup differences were detected.	One non-serious spinal-pain event was reported; non-adherence/losses were high (9/28; 33%), potentially limiting treatment effects.	Indicates no added benefit of remotely monitored trunk exercises over global exercises for PD balance/gait in this small trial; content-comparison evidence only.
Vloothuis et al. 2019 [116]	CARE4STROKE caregiver-mediated exercises with e-health support plus usual care versus usual care; outcomes at 8 and 12 weeks	SIS mobility; LOS; FMA-LE; Motricity Index; 6MWT; 10MWT; TUG; BBS; RMI; BI; NEADL	CARE4STROKE added caregiver-mediated task-specific mobility exercises with e-health support but did not improve primary mobility or length of stay.	Usual-care participants received guideline-based stroke rehabilitation without the structured caregiver-mediated e-health programme.	No significant between-group differences were found for SIS mobility at 8 weeks (beta 6.21; *p* = 0.229), 12 weeks (beta 0.14; *p* = 0.961), or LOS (*p* = 0.818). Secondary functional outcomes were also neutral, although patient anxiety and caregiver depression improved.	The intended additional dose was 150 min/week for 8 weeks; the authors suggested treatment contrast may have been insufficient. Caregiver burden did not increase.	Proof-of-concept evidence is neutral for mobility/LOS but relevant for transition support and caregiver-mediated implementation.
Wang et al. 2024 [117]	Internet remote home rehabilitation guidance plus wearable lower-limb device training versus routine post-hospital follow-up; outcomes at 4 and 12 weeks after discharge	FMA; MAS; BBS; MBI; HADS; gait parameters; BDNF, NT-3, NGF	The observation group showed greater improvements in motor function, balance, ADL, gait parameters and neurotrophic biomarkers, with lower spasticity and HADS scores.	The control group received routine medication guidance, home rehabilitation prescription and monthly/telephone follow-up advice.	At 4 and 12 weeks, FMA, BBS, MBI, stride length, gait speed/frequency and BDNF/NT-3/NGF were higher in the observation group, while MAS and HADS were lower (all *p* < 0.05).	Remote monitoring included app/video guidance, vital-sign monitoring and wearable walking training; allocation by odd/even numbering and limited methodological detail warrant caution.	Suggests potential benefit of Internet plus wearable rehabilitation after stroke, but should be treated as high-risk-of-bias narrative/sensitivity evidence.
Wilson et al. 2021 [118]	Home-based EDNA-22 virtual rehabilitation versus active GRASP control; post-intervention after 8 weeks	BBT; 9-HPT; MoCA; SIS; NFI	EDNA produced a significant and large improvement on BBT for the more-affected hand (g = 0.90) and improved MoCA performance (g = 0.70).	GRASP active control also targeted upper-limb function and showed mild-to-moderate 9-HPT improvement (g = 0.42).	EDNA effects appeared larger for BBT and cognition; 9-HPT improved mildly to moderately in both groups (EDNA g = 0.55; control g = 0.42). Functional self/informant measures improved only tentatively.	Seventeen participants completed training; one participant in each arm withdrew before intervention for personal reasons; no adverse events were reported.	Small active-comparator RCT suggests home virtual rehabilitation may improve upper-limb dexterity and cognition after stroke; effect estimates remain preliminary.
Wolf et al. 2015 [119]	Hand Mentor Pro plus home exercise programme versus dose-matched home exercise programme only; post-intervention after 8–12 weeks	ARAT; WMFT; FMA-UE	Robotic plus HEP was feasible and remotely monitored, with upper-limb gains across outcomes.	HEP-only participants completed dose-matched home exercises and functional activities with weekly therapist contact.	Both groups improved across upper-extremity outcomes, but there was no significant between-group difference in motor-function change over time.	Ninety-two of 99 randomized participants completed the trial; weekly phone/email monitoring supported compliance and progression in both groups.	Provides RCT evidence that telemonitored robotic therapy is feasible but not superior to dose-matched HEP in this underserved post-stroke population.
Wu et al. 2020 [120]	Collaborative-care telerehabilitation exercise training versus routine telephone follow-up; outcomes at discharge and 4, 8 and 12 weeks	FMA; BBS; TUG; 6MWT; MBI; SS-QOL	The collaborative telerehabilitation group showed greater recovery in motor function, balance, functional walking/endurance, ADL and stroke-specific QoL.	The control group received regular treatment during hospitalization and routine weekly telephone follow-up after discharge.	At 12 weeks, reported scores favoured intervention for FMA (83.70 ± 4.44 vs. 75.29 ± 2.89), BBS (43.13 ± 2.32 vs. 38.29 ± 2.70) and SS-QOL (190.57 ± 5.09 vs. 175.90 ± 5.78), with significant group-time interactions.	Sixty-one of 64 randomized participants completed the study; remote guidance was delivered twice weekly by a collaborative care team.	Supports collaborative-care telerehabilitation for post-discharge stroke recovery in China; include as an RCT candidate while noting single-blind design and complex intervention content.
Yokota et al. 2025 [121]	Home-based IoT ergometer telerehabilitation versus no home-based telerehabilitation after direct discharge home; 3-month assessment after stroke/TIA onset	6MWD; isometric knee extension strength; handgrip strength; SF-36 component scores	The TR group had a larger increase in 6 min walking distance after the 3-month home programme.	Matched controls received usual discharge guidance but no home-based TR platform.	After propensity-score matching, 6MWD increased more in TR than control (least-square mean change 57.9 m, 95% CI 38.9 to 76.9 vs. 16.7 m, 95% CI 5.6 to 27.7). HRQOL did not differ significantly between groups.	From 29 participants initially agreeing to TR, 21 were analyzed after exclusions/dropout and PSM; sessions were real-time nurse-supervised using ECG/heart-rate monitoring.	Suggests home TR can improve exercise capacity in high-functioning stroke/TIA survivors, but non-randomized PSM design limits causal inference.
Zheng et al. 2026 [122]	Remotely delivered aerobic/resistance exercise training versus active stretching control; post-intervention at 16 weeks and no-contact follow-up at 32 weeks	SPPB; 30STS; TUG; T25FW; 6MWT; BICAMS; GLTEQ; accelerometry	The exercise group showed moderate-to-large improvements in walking speed, functional mobility, lower-extremity function and verbal learning/memory.	The active control group completed remotely supported stretching matched for contact, coaching and behavioural content, but did not show comparable improvements.	Scientific feasibility analyses showed significant improvements in the exercise condition (*p* < 0.05; |d| = 0.58–0.80) but not control; the study was phase-Ib and not powered as a definitive efficacy trial.	Feasibility was strong: 51 randomized, 41 completed the 16-week conditions (80.4%), adherence and compliance exceeded 80%, and the intervention was described as safe and well received.	Supports feasibility and initial efficacy of theory-based remote exercise training for older adults with MS; appropriate for feasibility/sensitivity synthesis and future phase-II rationale.

Abbreviations: ABC, Activities-Specific Balance Confidence Scale; ABC-SF, Activities-Specific Balance Confidence Scale-Short Form; ABF, auditory biofeedback; AC, algorithm-controlled; ADL, activities of daily living; AMSQ, Arm Function in Multiple Sclerosis Questionnaire; APB, abductor pollicis brevis; ARAT, Action Research Arm Test; BBS, Berg Balance Scale; BBT, Box and Blocks Test; BDI-II, Beck Depression Inventory-II; BI, Barthel Index; BPI, Brief Pain Inventory; CAHAI, Chedoke Arm and Hand Activity Inventory; CBI, Caregiver Burden Inventory; CG, control group; CI, confidence interval; COR, conventional outpatient rehabilitation; CR, conventional rehabilitation; CSI, Caregiver Strain Index; CuPiD, smartphone-delivered cueing/biofeedback gait-training system; DGI, Dynamic Gait Index; EDSS, Expanded Disability Status Scale; EM, enhanced motivation/scaffolded gaming; ES, effect size; ETNS, electromyography-triggered neuromuscular stimulation; FAB, Frontal Assessment Battery; FAC, Functional Ambulation Category; FES, functional electrical stimulation; FGA, Functional Gait Assessment; FIM, Functional Independence Measure; FIS/FSS, Fatigue Impact Scale/Fatigue Severity Scale; FM/FMA, Fugl-Meyer Assessment; FMA-UE/FMUE/UEFMA, Fugl-Meyer Assessment for the Upper Extremity; FOG, freezing of gait; FOGQ, Freezing of Gait Questionnaire; FONEFIM, Telephone Version of the Functional Independence Measure; FRoM, functional range of motion; FTSST, Five Times Sit-to-Stand Test; GRASP, Graded Repetitive Arm Supplementary Program or Glove Rehabilitation Application for Stroke Patients according to study context; HADS, Hospital Anxiety and Depression Scale; HAQUAMS, Hamburg Quality of Life Questionnaire in Multiple Sclerosis; HBR, home-based rehabilitation; HEP, home exercise programme; HHD, hand-held dynamometry; HPT, home physical therapy; HTR, home-based telesupervising rehabilitation; IC, in-clinic therapy; IADL, instrumental activities of daily living; IMI, Intrinsic Motivation Inventory; IPA, Impact on Participation and Autonomy Questionnaire; ISNCSCI, International Standards for Neurological Classification of Spinal Cord Injury; ITT, intention-to-treat; K-ICARS, kinetic function subparameter of the International Cooperative Ataxia Rating Scale; LDJ, log dimensionless jerk; LEMS, lower-extremity motor score; LLFDI, Late-Life Function and Disability Instrument; LOS, length of stay; MAL, Motor Activity Log; MAL-30, Motor Activity Log-30; MAS, Modified Ashworth Scale; MBI, Modified Barthel Index; MDT, multidisciplinary team; MFAC, Modified Functional Ambulatory Category; MFES, Modified Falls Efficacy Scale; MFIS, Modified Fatigue Impact Scale; ML, machine learning; Mini-BESTest, mini-Balance Evaluation Systems Test; MDS-UPDRS, Movement Disorder Society-Unified Parkinson’s Disease Rating Scale; MMDT, Minnesota Manual Dexterity Test; MoCA, Montreal Cognitive Assessment; m-MAS, modified-Modified Ashworth Scale; MRI, magnetic resonance imaging; MS, multiple sclerosis; MS HAT, multiple sclerosis home automated tele-management; MSQOL-54, Multiple Sclerosis Quality of Life-54; MSSE, Multiple Sclerosis Self-Efficacy Questionnaire; MSWS-12, Multiple Sclerosis Walking Scale-12; MU, multi-user; MVPA, moderate-to-vigorous physical activity; NBS, Navigated Brain Stimulation; NEADL, Nottingham Extended Activities of Daily Living; NFOGQ, New Freezing of Gait Questionnaire; N-HPT/9-HPT, Nine-Hole Peg Test; OT, occupational therapy; PA, physical activity; PAHCO, physical-activity-related health competence; PAS, Parkinson’s Activity Scale; PBMSI, Preference-Based Multiple Sclerosis Index; PD, Parkinson’s disease; PDQ-8/PDQ-39, Parkinson’s Disease Questionnaire-8/39; PGWBI, Psychological General Well-Being Index; Pilates-TR, Pilates-based telerehabilitation; PwMS, people with multiple sclerosis; PwPD, people with Parkinson’s disease; QoL, quality of life; RCT, randomized controlled trial; RGS, Rehabilitation Gaming System; ROM, range of motion; RMS, root mean square; SABI, severe acquired brain injury; SCI, spinal cord injury; SCIM, Spinal Cord Independence Measure; SEE, Self-Efficacy for Exercise; SF-36, Short Form Health Survey-36; SIBT, sensory integration balance training; SIS, Stroke Impact Scale; SPARC, Spectral Arc Length; SPPB, Short Physical Performance Battery; STeleR, stroke telerehabilitation intervention; SU, single-user; SWA, SenseWear Armband; T25FW, Timed 25-Foot Walk; TA, tibialis anterior; TAI, Transfer Assessment Instrument; teleSCI, telerehabilitation in spinal cord injury; TIS, Trunk Impairment Scale; TOCT, task-oriented circuit training; TOCT-TR, task-oriented circuit training-based telerehabilitation; TR, telerehabilitation; TS, Tinetti Scale; TSRQ-15, Treatment Self-Regulation Questionnaire-15; TUG, Timed Up and Go; UE, upper extremity; UEMS, upper-extremity motor score; UL, upper limb; UTRT, usual territorial rehabilitative treatment; V-TOCT, virtual reality-supported task-oriented circuit therapy; VERGE, Virtual Environment for Rehabilitative Gaming Exercises; VR, virtual reality; VRBT, Virtual Reality Balance Trainer; VRRS, Virtual Reality Rehabilitation System; WHOQOL-BREF, World Health Organization Quality of Life-BREF; WISCI, Walking Index for Spinal Cord Injury; WMFT, Wolf Motor Function Test; 2MWD, 2 min walking distance; 2MWT, 2 min walk test; 6MPT, 6 min push test; 6MWT, 6 min walk test; 10-MWT, 10 m walk test.; A-VO2, arteriovenous oxygen difference; AG, asynchronous group; BBA, Brunel Balance Assessment; BS-OT, bedside occupational therapist; CSE, core-stability exercises; DASH, Disabilities of the Arm, Shoulder and Hand; EARS, Exercise Adherence Rating Scale; EQ-5D/EQ-5D-5L, EuroQol 5 Dimension/5-Level; FTF, in-person; GABS-B, Gait and Balance Scale Part B; G1/G2/G3, study group 1/study group 2/control group; HCAD, Home Care Activity Desk; IHT, in-home teletherapy; K-MBI, Korean Modified Barthel Index; LTCF, long-term care facility; MG, mixed-format group; NHPT, Nine-Hole Peg Test; PASS, Postural Assessment Scale for Stroke; PHQ-9, Patient Health Questionnaire-9; POMA-B/POMA-G, Performance-Oriented Mobility Assessment balance/gait subscale; RAHFT, ReJoyce Automated Hand Function Test; ReJoyce, Rehabilitation Joystick for Computerized Exercise; RMI, Rivermead Mobility Index; SARA, Scale for the Assessment and Rating of Ataxia; S-TIS, Spanish Trunk Impairment Scale; STRIVE, STRoke Interactive Virtual thErapy; SUS, System Usability Scale; SWST, Stand–Walk–Sit Test; TBI, traumatic brain injury; tele-OT, tele-occupational therapist; UC, usual care; VAS, Visual Analogue Scale; VT, virtual therapy; 4MWT, 4-Metre Walk Test; 5STS, Five Times Sit-to-Stand Test; APDDS, Adapted Patient Determined Disease Steps; BICAMS, Brief International Cognitive Assessment for MS; BDNF, brain-derived neurotrophic factor; CAS, Coronavirus Anxiety Scale; CIMT, Constraint-Induced Movement Therapy; EDNA, Elements system; FTHUE-HK, Functional Test for Upper Extremity-Hong Kong; HMP, Hand Mentor Pro; HRQOL, health-related quality of life; IoT, Internet of Things; MAL-AOU, Motor Activity Log-Amount of Use; MAL-QOM, Motor Activity Log-Quality of Movement; Mii-vitaliSe, MS Nintendo Wii intervention package; NIVRT, nonimmersive virtual reality training; NT-3, neurotrophin-3; PSM, propensity-score matching; SR, Smart Reminder; TIA, transient ischemic attack; V-ABC, Virtual Arm Boot Camp; ACTIV, Augmented Community Telerehabilitation Intervention; AROM, active range of motion; CARE4STROKE, caregiver-mediated exercises with e-health support after stroke; HAMD, Hamilton Depression Rating Scale; IT-AAT, Italian version of the Aachen Aphasia Test; MCID, minimal clinically important difference; OCS, Oxford Cognitive Screen; STREAM, Stroke Rehabilitation Assessment of Movement Scale; TENZR, wearable reach-to-grasp feedback device; WHODAS, World Health Organization Disability Assessment Schedule.

**Table 4:** Primary and exploratory meta-analysis results by comparator type and motor domain.

Comparator Type	Motor Domain	Outcome/Time Point	Studies, k	Participants, n	Effect Measure	Model	Pooled Effect	95% CI	Heterogeneity	Certainty of Evidence	Interpretation
Telerehabilitation vs. usual care/no additional therapy/waitlist	Walking/mobility activities	Post-intervention	3 [63,110,120]	121	SMD, Hedges’ g	REML random-effects + Hartung–Knapp	1.06	−0.23 To 2.35	I^2^ = 57.6%; τ^2^ = 0.161	Very low	No clear between-group difference. Interpret cautiously because of heterogeneity.
Telerehabilitation vs. usual care/no additional therapy/waitlist	Global motor-functional/ADL activity	Post-intervention to 6 months	3 [51,109,120]	304	SMD, Hedges’ g	REML random-effects + Hartung–Knapp	0.83	−0.35 To 2.00	I^2^ = 73.6%; τ^2^ = 0.161	Very low	No clear between-group difference. Interpret cautiously because of heterogeneity.
Telerehabilitation vs. conventional/in-person or active rehabilitation	Upper/global motor function	Change score at post-intervention	2 [42,49]	87	SMD, Hedges’ g	REML random-effects, exploratory	0.67	0.23 To 1.10	I^2^ = 0.0%; τ^2^ = 0.000	Very low	Exploratory active-comparator summary; limited clinical exchangeability and no confirmatory inference.
Telerehabilitation vs. usual/active home rehabilitation	Balance-related activity/postural control	Post-intervention	2 [91,120]	121	SMD, Hedges’ g	REML random-effects, exploratory	1.15	−0.29 To 2.59	I^2^ = 92.5%; τ^2^ = 1.000	Very low	Descriptive two-study comparison; high heterogeneity precludes generalizable pooled inference.
Telerehabilitation vs. usual care/sham or handout control	Upper-limb motor impairment	Change score at post-intervention	2 [38,112]	58	MD	REML random-effects, exploratory	4.93	−1.44 To 11.30	I^2^ = 83.5%; τ^2^ = 17.914	Very low	Descriptive two-study comparison; high heterogeneity precludes generalizable pooled inference.

Positive values favour telerehabilitation unless otherwise stated. The primary random-effects models used restricted maximum likelihood estimation for between-study variance, with Hartung–Knapp confidence intervals when k ≥ 3. The DerSimonian–Laird random-effects models were sensitivity analyses only; no reported pooled result used a fixed-effect model. The two-study model outputs are exploratory, and those with high heterogeneity are interpreted primarily as descriptive paired comparisons. Prediction intervals, funnel plots, and Egger-type tests were not estimated because k < 10. Certainty was finalized at the comparison-outcome level using GRADE. Abbreviations: ADL, activities of daily living; CI, confidence interval; GRADE, Grading of Recommendations Assessment, Development and Evaluation; HK, Hartung–Knapp; MD, mean difference; REML, restricted maximum likelihood; SMD, standardized mean difference; TR, telerehabilitation; τ^2^, between-study variance.

**Table 5:** Sensitivity, subgroup, robustness, and certainty-of-evidence summary.

Primary Analysis/Domain	Sensitivity or Subgroup Analysis	Eligibility Rule	Studies/Participants	Effect Estimate (95% CI)	Heterogeneity	Change from Primary Analysis	Risk-of-Bias/GRADE Implication	Robustness Interpretation
Walking/mobility activities (TR vs. usual care/no additional therapy/waitlist)	DerSimonian–Laird versus REML	Same 3 studies; DL τ^2^ estimator used as sensitivity only.	k = 3; n = 121	1.06 (0.45 to 1.66)	I^2^ = 57.6%; τ^2^ = 0.164	Direction unchanged; DL CI was narrower than the primary REML/HK CI.	No change to certainty; DL retained only as sensitivity.	Sensitivity estimate favoured TR, but did not override the more conservative REML/HK primary inference.
Walking/mobility activities (TR vs. usual care/no additional therapy/waitlist)	Leave-one-out influence analysis	Each contributing study removed in turn; performed because k = 3.	k = 2 in each iteration; n = 60–91	Omitting Eldemir et al. study 2024 [63] 1.35 (0.90 to 1.81), n = 91, I^2^ = 0.0%, τ^2^ = 0.000; omitting Swarnakar et al. 2023 [110]: 1.04 (0.04 to 2.04), n = 91, I^2^ = 78.6%, τ^2^ = 0.408; omitting Wu et al. 2020 [120]: 0.76 (0.23 to 1.30), n = 60, I^2^ = 3.0%, τ^2^ = 0.005	I^2^ range 0.0% to 78.6%; τ^2^ range 0.000 to 0.408	All leave-one-out estimates remained positive; magnitude ranged from 0.76 to 1.35.	Influence analysis supports the GRADE concerns for inconsistency and imprecision.	No single study reversed the direction of effect; interpretation remains cautious because each leave-one-out estimate is k = 2 exploratory.
Walking/mobility activities (TR vs. usual care/no additional therapy/waitlist)	Excluding SCI study	Removed Swarnakar et al. 2023 [110] because SCIM mobility differs from stroke/MS walking tests.	k = 2; n = 91	1.04 (0.04 to 2.04)	I^2^ = 78.6%; tau^2^ = 0.408	Direction remained positive; sensitivity estimate remained exploratory.	Does not remove imprecision; informs indirectness related to diagnosis/scale heterogeneity.	Effect direction was unchanged, but heterogeneity remained substantial; primary inference remains very low certainty.
Global motor-functional/ADL activity (TR vs. usual care/no additional therapy/waitlist)	DerSimonian–Laird versus REML	Same 3 studies; DL τ^2^ estimator used as sensitivity only.	k = 3; n = 304	0.83 (0.30 to 1.35)	I^2^ = 73.6%; τ^2^ = 0.158	Direction unchanged; DL CI was narrower than the primary REML/HK CI.	No change to certainty; DL retained only as sensitivity.	Sensitivity estimate favoured TR, but did not override the more conservative REML/HK primary inference.
Global motor-functional/ADL activity (TR vs. usual care/no additional therapy/waitlist)	Leave-one-out influence analysis	Each contributing study removed in turn; performed because k = 3.	k = 2 in each iteration; n = 104–261	Omitting Chumbler et al. 2012 [51]: 1.12 (0.86 to 1.38), n = 261, I^2^ = 0.0%, τ^2^ = 0.000; omitting Sun et al. 2025 [109]: 0.59 (−0.03 to 1.21), n = 104, I^2^ = 58.6%, τ^2^ = 0.118; omitting Wu et al. 2020 [120]: 0.76 (−0.16 to 1.68), n = 243, I^2^ = 86.5%, τ^2^ = 0.381	I^2^ range 0.0% to 86.5%; τ^2^ range 0.000 to 0.381	All leave-one-out estimates remained positive; magnitude ranged from 0.59 to 1.12.	Influence analysis supports the GRADE concerns for inconsistency and imprecision.	No single study reversed the direction of effect; certainty remains limited by broad constructs and small k.
Global motor-functional/ADL activity (TR vs. usual care/no additional therapy/waitlist)	Excluding 6-month outcome	Removed Chumbler et al. 2012 [51] to align post-intervention/12-week ADL outcomes.	k = 2; n = 261	1.12 (0.86 to 1.38)	I^2^ = 0.0%; tau^2^ = 0.000	Magnitude increased and heterogeneity decreased, but the estimate became k = 2 exploratory.	Informs time-point indirectness; imprecision remains.	Post-intervention-only evidence was directionally consistent but remains exploratory.
Upper/global motor function (TR vs. conventional/in-person or active rehabilitation)	DerSimonian–Laird versus REML	Same 2 studies; DL τ^2^ estimator used as sensitivity only.	k = 2; n = 87	0.67 (0.23 to 1.10)	I^2^ = 0.0%; τ^2^ = 0.000	Direction unchanged.	No change to certainty; exploratory k = 2 evidence.	DL result was identical to REML because τ^2^ = 0; inference remains exploratory.
Upper/global motor function (TR vs. conventional/in-person or active rehabilitation)	Excluding active home-OT pilot study	Removed Ballester et al. 2017 [42]; restriction left Chen et al. 2020 [49] only.	k = 1; n = 52	Not pooled after restriction.	Not estimable.	Non-estimable after exclusion.	Reinforces indirectness and imprecision.	The comparison should not be used for confirmatory inference.
Balance-related activity/postural control (TR vs. usual/active home rehabilitation)	DerSimonian–Laird versus REML	Same 2 studies; DL τ^2^ estimator used as sensitivity only.	k = 2; n = 121	1.15 (−0.29 to 2.59)	I^2^ = 92.5%; τ^2^ = 1.000	Direction unchanged.	No change to certainty; exploratory k = 2 evidence.	High heterogeneity remained; interpretation remains exploratory and very low certainty.
Balance-related activity/postural control (TR vs. usual/active home rehabilitation)	Excluding EMM/SE-derived SD study	Removed Pagliari et al. 2024 [91]; restriction left Wu et al. 2020 [120] only.	k = 1; n = 61	Not pooled after restriction.	Not estimable.	Non-estimable after exclusion.	Reinforces imprecision and data-source concerns.	Balance evidence remains exploratory and sensitive to data-source assumptions.
Upper-limb motor impairment (TR vs. usual care/sham or handout control)	DerSimonian–Laird versus REML	Same 2 studies; DL τ^2^ estimator used as sensitivity only.	k = 2; n = 58	4.93 (−1.44 to 11.30)	I^2^ = 83.5%; τ^2^ = 17.914	Direction unchanged.	No change to certainty; exploratory k = 2 evidence.	Heterogeneity remained high; interpretation remains exploratory.
Upper-limb motor impairment (TR vs. usual care/sham or handout control)	Excluding sham/handout comparator study	Removed Toh et al. 2025a [112]; restriction left Adams et al. 2023 [38] only.	k = 1; n = 18	Not pooled after restriction.	Not estimable.	Non-estimable after exclusion.	Highlights sensitivity to comparator definition.	Upper-limb FMA-UE pooled evidence should remain exploratory.

Note: The sensitivity analyses were interpreted as robustness checks and not as independent confirmatory analyses. The leave-one-out analyses were performed only when k ≥ 3. DerSimonian–Laird models were retained as sensitivity analyses only. The rows marked not pooled indicate that the sensitivity restriction left fewer than two compatible studies. Abbreviations: CI, confidence interval; DL, DerSimonian–Laird; GRADE, Grading of Recommendations Assessment, Development and Evaluation; HK, Hartung–Knapp; k, number of studies; REML, restricted maximum likelihood; RoB, risk of bias; SMD, standardized mean difference; TR, telerehabilitation.

**Table 6:** Evidence-distribution matrix by neurological population, motor domain, delivery mode and technology family. Panel (**A**): Outcome-domain distribution by neurological population. Panel (**B**): Dominant delivery-mode and technology-family distribution by neurological population.

**(A)**								
**Population**	**Included Studies, n**	**Upper Limb, n**	**Walking/Mobility Activities, n**	**Balance-Related Activity/Postural Control, n**	**Global Motor-Functional/ADL Activity, n**	**Interpretative Implication**		
Stroke	50	28	23	19	25	Dominant evidence base; broad outcome coverage but still heterogeneous.		
Parkinson’s disease	15	1	14	13	3	Mainly gait, balance, and disease-specific motor outcomes.		
Multiple sclerosis	14	4	9	11	2	Mainly gait/balance, physical activity, and postural-control evidence.		
Spinal cord injury	5	1	2	0	3	Small functional-independence/mobility evidence; mostly narrative.		
ABI/TBI/mixed	2	1	1	2	1	Sparse mixed-diagnosis evidence; avoid diagnosis-specific inference.		
Ataxia	1	0	1	1	0	Single pilot study; feasibility-level evidence only.		
**(B)**								
**Population**	**Synchronous, n**	**Asynchronous, n**	**Hybrid, n**	**Standard Digital/App/Web/ Video/Telephone, n**		**VR/Exergaming, n**	**Wearable/Sensor-Assisted, n**	**Robotic/FES/Device-Assisted, n**
Stroke	13	18	19	17	18	12	3	
Parkinson’s disease	6	6	3	8	2	5	0	
Multiple sclerosis	4	7	3	7	4	3	0	
Spinal cord injury	3	1	1	2	0	1	2	
ABI/TBI/mixed	1	0	1	1	1	0	0	
Ataxia	0	0	1	1	0	0	0	

The counts are derived from the 87 included comparative studies. The outcome-domain counts are non-mutually exclusive because a single study could report more than one motor domain. Delivery mode and technology family were coded at the dominant study level; the studies comparing synchronous and asynchronous formats or combining structured synchronous and asynchronous components were coded as hybrid. ABI, acquired brain injury; ADL, activities of daily living; FES, functional electrical stimulation; MS, multiple sclerosis; SCI, spinal cord injury; TBI, traumatic brain injury; VR, virtual reality.

## Data Availability

All data generated or analyzed during this systematic review and meta-analysis are included in the published article and its Supplementary Materials. No individual participant-level data were collected or analyzed. Further inquiries can be directed to the corresponding author.

## Supplementary Materials

The following supporting information can be downloaded at https://www.mdpi.com/article/10.3390/brainsci16090972/s1, Table S1: PROSPERO registration and protocol record; Table S2: Database-specific electronic search strings reproduced verbatim; Table S3: Records retrieved by database and search string; Table S4: PRISMA 2020 study-selection flow and screening counts; Table S5: Title/abstract screening exclusion categories; Table S6: Full-text exclusion reasons; Table S7: PICO framework and eligibility criteria; Table S8: Study selection, deduplication, and inter-rater agreement workflow; Table S9: Data extraction framework and telerehabilitation delivery-model taxonomy; Table S10: ICF-informed outcome hierarchy and motor-domain classification; Table S11: Planned quantitative synthesis and methodological safeguards; Table S12: Study-level GRADE-domain inputs supporting certainty judgments; Table S13: Supporting GRADE comparison/outcome considerations; Table S14: Study-level synthesis role and risk-of-bias linkage; Table S15: Primary and exploratory meta-analysis input values; Table S16: Model specification, pooled results, and sensitivity outputs; Table S17: Final GRADE evidence profile for quantitative and key non-pooled outcomes.
