# Supplementary material for: Telerehabilitation for Motor Recovery in Adults with Neurological Disorders: A Systematic Review and Meta-Analysis Across Motor Domains, Delivery Models, and Follow-Up Effects
Source: Brain Sci. 2026 Sep 14;16(9):972. doi: 10.3390/brainsci16090972 (PMC13604133; doi:10.3390/brainsci16090972)
Supplement: Supplementary file 1 [file brainsci-16-00972-s001.zip › brainsci-4500230-supplementary.pdf]

Supplementary Material S1

Protocol Registration, Search Strategy, Screening Flow, Eligibility Framework, Outcome Hierarchy, and Planned Evidence Synthesis

Systematic review and meta-analysis on telerehabilitation for motor recovery in adults with neurological disorders.

Protocol registration

The protocol for this systematic review and meta-analysis was registered in PROSPERO on 5 June 2026 before completion of the electronic database searches. The registered protocol specified the review question, PICO framework, eligibility criteria, information sources, screening workflow, data-extraction domains, risk-of-bias tools, planned quantitative synthesis, sensitivity analyses, and certainty-of-evidence approach. The final review was conducted using the registered methodological framework, with subsequent search and screening details documented below.

Table S1. PROSPERO registration and protocol record

Protocol item	Details
Review title	Telerehabilitation for Motor Recovery in Adults With Neurological Disorders: A Systematic Review and Meta-Analysis Across Motor Domains, Delivery Models, and Follow-Up Effects.
Registry	PROSPERO, International Prospective Register of Systematic Reviews.
Registration date	5 June 2026.
Registration number	CRD420261416563.
Protocol version and date	Protocol version 1.0; 05 June 2026.
Review type	Systematic review and meta-analysis of controlled comparative studies.
Population	Adults aged 18 years or older with neurological disorders associated with motor impairment.
Intervention	Active motor telerehabilitation delivered fully or partly through remote, digital, telehealth, or hybrid models.
Main outcomes	Validated motor or motor-functional outcomes, including upper limb function, gait or mobility, balance or postural control, global motor function, disease-specific motor outcomes, and motor-related activities of daily living.
Planned evidence appraisal	Risk of bias using RoB 2 for randomized trials and ROBINS-I for non-randomized controlled studies; certainty of evidence using the GRADE approach.

Table S2. Database-specific electronic search strings reproduced verbatim

Search	Purpose	Complete search string	Databases	Final search date	Coverage period	Filters or limits applied
String 1	Primary balanced search for telerehabilitation, neurological diagnoses, motor outcomes, and comparative designs.	("telerehabilitation" OR "tele-rehabilitation" OR "remote rehabilitation" OR "home-based rehabilitation" OR "virtual rehabilitation" OR "web-based rehabilitation") AND (stroke OR "Parkinson disease" OR "Parkinson's disease" OR "multiple sclerosis" OR "spinal cord injury" OR "brain injury" OR ataxia) AND ("motor function" OR "upper limb" OR "lower limb" OR gait OR walking OR mobility OR balance) AND (randomized OR randomised OR controlled OR trial OR cohort OR comparative)	PubMed; Web of Science; Cochrane Library; Embase; Scopus	8 June 2026	Inception to 8 June 2026	No field tags, no truncation, and no search-string-level date limits. Eligibility criteria were applied during screening and full-text assessment.
String 2	Complementary technology/delivery-model search to improve capture of remote exercise, web-based physiotherapy, virtual reality, exergaming, and wearable rehabilitation studies.	("telerehabilitation" OR "remote exercise" OR "web-based physiotherapy" OR "virtual reality rehabilitation" OR "exergaming rehabilitation" OR "wearable rehabilitation") AND (stroke OR "Parkinson disease" OR "multiple sclerosis" OR "spinal cord injury" OR "brain injury" OR ataxia) AND ("upper limb" OR gait OR mobility OR balance OR "activities of daily living") AND (randomized OR randomised OR controlled OR pilot OR feasibility OR comparative)	PubMed; Web of Science; Cochrane Library; Embase; Scopus	8 June 2026	Inception to 8 June 2026	No field tags, no truncation, and no search-string-level date limits. Eligibility criteria were applied during screening and full-text assessment.

The two Boolean strings were applied as database-agnostic, field-free strategies. Cochrane Reviews/CDSR records were counted within Cochrane Library rather than as a separate database.

Table S3. Records retrieved by database and search string

Database	String 1 records	String 2 records	Combined records used for PRISMA identification
PubMed	422	378	800
Web of Science	687	492	1,179
Cochrane Library	427	367	794
Embase	908	819	1,727
Scopus	11,968	9,867	21,835
Total before deduplication	13,412	11,923	26,335

All database searches were completed on 8 June 2026. The combined database totals were used for the PRISMA identification box before deduplication.

Table S4. PRISMA 2020 study-selection flow and screening counts

Stage	n	Details
Records identified from databases	26,335	PubMed 800; Web of Science 1,179; Cochrane Library 794; Embase 1,727; Scopus 21,835.
Records identified from registers	0	No register records were added to the database search yield.
Duplicate records removed before screening	10,736	Cross-database and cross-string duplicates.
Records marked as ineligible by automation tools	0	No automated exclusion was applied before screening.
Records removed for other reasons before screening	0	No additional pre-screening removal reason was applied.
Records screened at title/abstract level	15,599	Calculated as 26,335 records identified minus 10,736 duplicates removed.
Records excluded at title/abstract level	15,182	Excluded as clearly irrelevant, ineligible design/publication type, no active telerehabilitation or remote rehabilitation component, non-neurological or non-adult population, or no relevant motor/motor-functional outcome.
Reports sought for retrieval	417	Potentially eligible reports after title/abstract screening.
Reports not retrieved	0	All sought full-text reports were retrieved.
Reports assessed for eligibility	417	Full-text reports assessed by the reviewers.
Reports excluded with reasons	330	Full-text exclusions were assigned one primary reason.
Studies included in review	87	Studies included in the qualitative evidence synthesis.

Table S5. Title/abstract screening exclusion categories

Primary title/abstract exclusion category	Records excluded, n	Percentage of title/abstract exclusions
Clearly irrelevant topic	6,067	40.0%
Ineligible study design or publication type	5,023	33.1%
No active telerehabilitation or remote rehabilitation component	2,189	14.4%
Non-neurological or non-adult population	1,507	9.9%
No relevant motor or motor-functional outcome	396	2.6%
Total title/abstract records excluded	15,182	100.0%

One primary exclusion category was assigned to each record excluded at title/abstract screening. Categories were aligned with the eligibility criteria and PRISMA flow diagram.

Table S6. Full-text exclusion reasons

Primary full-text exclusion reason	Reports excluded, n	Percentage of full-text exclusions
No eligible motor outcome or motor-functional endpoint	96	29.1%
No active telerehabilitation intervention or intervention not primarily remotely delivered	82	24.8%
Ineligible population or data not separately extractable for eligible adults with neurological disorders	54	16.4%

Primary full-text exclusion reason	Reports excluded, n	Percentage of full-text exclusions
Ineligible study design or publication type (e.g., protocol, abstract only, review, case report, non-comparative)	48	14.5%
No eligible comparator or insufficient comparative data	29	8.8%
Duplicate/companion report or overlapping dataset not retained as primary study	21	6.4%
Total full-text reports excluded	330	100.0%

Full-text reasons refer to the primary reason for exclusion and sum to the total number of reports excluded after full-text assessment.

Table S7. PICO framework and eligibility criteria

PICO element	Eligibility definition	Exclusion rationale
Population	Adults aged 18 years or older with a neurological disorder and motor impairment. Eligible diagnoses included stroke, Parkinson disease, Parkinson's disease, multiple sclerosis, spinal cord injury, traumatic brain injury, acquired brain injury, ataxia, and other adult neurological disorders when a motor rehabilitation outcome was assessed.	Pediatric/adolescent-only samples, healthy older adults, non-neurological samples, orthopedic-only, musculoskeletal-only, cardiopulmonary-only, or frailty-only samples without a neurological diagnosis were excluded unless adult neurological data were separately extractable.
Intervention	Active motor telerehabilitation delivered fully or partly remotely through videoconferencing, digital platforms, apps, virtual reality, exergaming, wearable or sensor systems, connected home programmes, or hybrid in-person plus remote models.	Passive telemonitoring, teleconsultation without active motor training, health education alone, psychological support alone, cognitive-only, speech-only, swallowing-only interventions, and generic fitness apps without neurological rehabilitation intent were excluded.
Comparator	Usual care, no additional intervention, waiting list, unsupervised home exercise, conventional face-to-face rehabilitation, or another active rehabilitation comparator.	Studies without a comparator were excluded from the primary meta-analysis because comparative effect estimates could not be derived.
Outcomes	At least one validated motor or motor-functional outcome. Primary domains included upper limb function, gait or mobility, balance or postural control, global motor function, and disease-specific motor outcomes. Secondary outcomes included ADL, participation, falls, adherence, adverse events, satisfaction, usability, quality of life, caregiver burden, and resource use.	Studies reporting only non-motor outcomes or feasibility outcomes without any eligible motor or motor-functional measure were excluded.
Study design	Randomized controlled trials, quasi-randomized controlled trials, and controlled clinical trials were eligible. Non-randomized controlled studies were retained for narrative synthesis or sensitivity analyses when relevant comparative data were available.	Case reports, case series, single-arm pre-post studies, protocols, editorials, narrative/scoping/systematic reviews, conference abstracts without sufficient data, theses, non-peer-reviewed records, and non-comparative reports were excluded.

Table S8. Study selection, deduplication, and inter-rater agreement workflow

Stage	Procedure	Reviewers	Documentation fields	Agreement or decision rule
Identification	Records were retrieved from PubMed, Web of Science, Cochrane Library, Embase, and Scopus using two database-agnostic Boolean search strings.	RSC and AC verified source completeness.	Database-specific and string-specific counts are reported in Tables S2-S4.	Not applicable.
Deduplication	Duplicate records were removed before title/abstract screening using electronic deduplication followed by manual verification where needed.	RSC and AC verified uncertain duplicates.	10,736 duplicate records were removed before screening.	Discrepancies were resolved by discussion.
Title and abstract screening	Titles and abstracts were screened against the eligibility criteria.	RSC and AC screened independently.	15,599 records were screened; 15,182 records were excluded with one primary title/abstract exclusion category.	Cohen's kappa = 0.78, interpreted as substantial agreement.
Full-text assessment	Potentially eligible full texts were assessed against all criteria, and reasons for exclusion were recorded.	RSC and AC assessed independently.	417 reports were assessed for eligibility; 330 full-text reports were excluded with reasons.	Cohen's kappa = 0.76, interpreted as substantial agreement.
Final inclusion	Studies meeting all criteria were included in qualitative synthesis and, when sufficiently homogeneous, quantitative synthesis.	Final inclusion was confirmed by consensus.	87 studies were included in the review.	Any unresolved disagreement was referred to a third reviewer.

Table S9. Data extraction framework and telerehabilitation delivery-model taxonomy

Extraction domain	Variables or taxonomy categories	Coding rule
Study and sample characteristics	Study design, country, setting, recruitment source, sample size, neurological diagnosis, age, sex, disease duration, time since event when relevant, impairment severity, inclusion criteria, and exclusion criteria.	Extract exactly as reported. Convert units only when needed and record assumptions.
Intervention characteristics	Intervention content, therapeutic targets, delivery mode, technology used, synchronous or asynchronous components, therapist involvement, caregiver involvement, safety monitoring, and progression rules.	Classify the model by dominant delivery feature while retaining secondary components.
Dose and adherence	Session frequency, session duration, total programme duration, cumulative rehabilitation time, prescribed dose, completed dose, adherence, attrition, and reasons for non-completion.	Prefer objectively recorded adherence when available; otherwise extract participant-reported or therapist-reported adherence.
Comparator and outcomes	Comparator content, intensity, setting, outcome measures, assessment time points, adverse events, funding source, conflicts of interest, and trial registration details.	Record whether comparator intensity is matched, lower, higher, or unclear compared with telerehabilitation.
Delivery-model taxonomy	Synchronous therapist-led videoconferencing; asynchronous app or platform-based telerehabilitation; hybrid in-person plus remote rehabilitation; VR or exergaming-based telerehabilitation; sensor or wearable-assisted telerehabilitation; caregiver-supported telerehabilitation.	Use mutually informative categories. A study may be coded with a primary and secondary delivery model when justified.

Table S10. ICF-informed outcome hierarchy and motor-domain classification

Outcome level	Domain	Examples of eligible measures	Time-point classification
Primary motor outcome	Upper limb function	Fugl-Meyer Assessment upper extremity, Action Research Arm Test, Box and Block Test, Nine-Hole Peg Test, grip or dexterity measures when validated.	Post-intervention; short-term follow-up <=3 months; medium-term follow-up >3 to 6 months; long-term follow-up >6 months.
Primary motor outcome	Gait and mobility	10-Meter Walk Test, 6-Minute Walk Test, Timed Up and Go, gait speed, walking endurance, validated mobility scales.	Same time-point hierarchy.
Primary motor outcome	Balance and postural control	Berg Balance Scale, Mini-BESTest, Functional Reach Test, postural control measures, validated balance tests.	Same time-point hierarchy.
Primary motor outcome	Global motor function	Fugl-Meyer total score, Motor Assessment Scale, Functional Independence Measure motor subscale, Barthel Index when used as a motor-functional measure.	Same time-point hierarchy.
Primary motor outcome	Disease-specific motor outcomes	Unified Parkinson's Disease Rating Scale Part III, multiple sclerosis walking outcomes, spinal cord injury motor scores, ataxia motor scales when motor-specific.	Same time-point hierarchy.
Secondary outcomes	Function, implementation, safety, and experience	ADL, participation, falls or fall risk, adherence, adverse events, satisfaction, usability, quality of life, caregiver burden, and resource use.	Extract all reported time points and map to the predefined follow-up categories when possible.

Table S11. Planned quantitative synthesis and methodological safeguards

Synthesis component	Decision rule	Reporting implication
Primary pooling set	Randomized or quasi-randomized comparisons directly evaluating telerehabilitation against usual care, no intervention, waiting list, home exercise, or conventional face-to-face rehabilitation comparator, when population, outcome domain, and time point were sufficiently similar.	Primary meta-analysis when >=2 clinically compatible studies were available; k = 2 analyses interpreted as exploratory.
Studies retained outside primary pooling	Non-randomized controlled studies, feasibility/pilot trials not designed for definitive efficacy, delivery-model-only comparisons, and studies without extractable variance data.	Narrative synthesis or sensitivity analyses when clinically relevant and data permitted.
Model specification	Random-effects models were planned because clinical and methodological heterogeneity was expected. Between-study variance was estimated using restricted maximum likelihood.	Hartung-Knapp adjustment used when k >=3; DerSimonian-Laird retained only as sensitivity analysis.
Small-study safeguards	Prediction intervals, funnel plots, and Egger-type tests were not planned unless at least 10 studies contributed to a comparison.	Leave-one-out analyses performed only when k >=3.
Effect direction	Effect estimates were harmonized so that positive values favored telerehabilitation unless the direction could not be safely transformed.	Direction of effect was recorded during extraction.

GRADE evidence profile and certainty-of-evidence inputs

These tables report GRADE-domain judgments for studies [36-122]. Table S12 provides study-level inputs used to support certainty judgments, whereas Table S13 summarizes provisional comparison/outcome-level certainty considerations. Final certainty should be confirmed after the definitive meta-analysis model, RoB consensus, and outcome-specific pooling decisions are finalized.

Table S12. Study-level GRADE-domain inputs supporting certainty judgments.

Study	Design/tool	Risk of bias	Inconsistency	Indirectness	Imprecision	Publication bias	GRADE role/certainty impact
García-Rudolph et al. 2024 [36]	Matched case-control; ROBINS-I	Very serious: historical controls and residual confounding despite propensity matching; pandemic-era selection of medically stable supported patients.	Not assessable at study level; not pooled with RCTs.	Serious for causal TR effectiveness; directly relevant to supported teleSCI but pandemic/home-support context limits generalizability.	Serious: matched n = 84 and non-significant differences.	Not assessable; single observational study.	Non-randomized narrative evidence; starts low and likely downgrades to very low.
Thielbar et al. 2020 [37]	Randomized crossover; RoB 2	Some concerns: small sample, no participant blinding, simple randomization by sequence, and delivery-mode comparison.	Not assessable; single delivery-mode trial.	Very serious for primary effectiveness because both modes were home VR telerehabilitation.	Serious: n = 20 and engagement-focused outcomes.	Not assessable.	Delivery-model evidence only; not used for primary telerehabilitation-vs-control certainty.
Adams et al. 2023 [38]	Rater-blinded RCT; RoB 2	Serious/some concerns: rater blinding and block randomization reported, but small sample, attrition, non-blinded treatment, and intellectual-property conflict.	Not assessable; single RCT in formal upper-limb comparison.	Not serious: adult stroke, home VR telerehabilitation with OT support, usual-care comparator, validated FMUE outcome.	Serious: single small RCT despite clinically favorable effect.	Not assessable.	Contributes to upper-limb GRADE; preliminary certainty low after downgrading for RoB and imprecision.
Allegue et al. 2022 [39]	Feasibility randomized clinical trial; RoB 2	Very serious: feasibility design, very small analyzed sample, attrition, COVID-related protocol extension, and partial loss of blinding during remote assessment.	Not assessable; not suitable for pooled efficacy inference.	Serious: feasibility objective and pandemic-modified delivery reduce applicability to definitive efficacy.	Very serious: n = 9 analyzed and outcome effects not powered.	Not assessable.	Narrative/feasibility evidence; very low certainty contribution.
Asano et al. 2021 [40]	Evaluator-blinded RCT; RoB 2	Serious/some concerns: randomized and evaluator-blinded, but withdrawals before 3 months, co-interventions permitted, low telerehabilitation exposure, and self-reported primary outcome.	Contributes to cross-study heterogeneity if pooled with global functional outcomes.	Serious: primary LLFDI disability is self-reported and usual rehabilitation was allowed in both arms.	Serious: 95% CIs cross the null for primary LLFDI outcomes.	Not assessable.	Contributes to global functional GRADE; likely low certainty.
Aytutuldu et al. 2024 [41]	Single-blind RCT; RoB 2	Serious: small sample, participants/therapists not blinded, and outcome assessment may be vulnerable to performance/measurement bias.	Not pooled for primary TR effectiveness.	Very serious: both arms received synchronous telerehabilitation; comparison tests exercise content rather than TR vs non-TR.	Serious: n = 34 randomized/ITT, 32 completed.	Not assessable.	Delivery/content evidence only; not primary GRADE effectiveness evidence.
Ballester et al. 2017 [42]	Parallel-group RCT; RoB 2	Very serious/serious: small chronic-stroke sample, outcome assessors not blinded, retrospective trial registration, and selective neurophysiology subgroup.	Potentially inconsistent with other upper-limb active-comparator evidence.	Serious: home VR vs matched home OT tests VR-specific content in chronic stroke rather than broad TR access effect.	Serious: small n = 35 analyzed; clinical significance uncertain.	Not assessable.	Active-comparator/sensitivity evidence; low to very low certainty.
Benvenuti et al. 2014 [43]	Longitudinal cohort with geographic control; ROBINS-I	Very serious: non-randomized geographic controls, baseline differences, uncontrolled confounding, and adherence heterogeneity.	Not pooled with RCT evidence; observational direction may not resolve inconsistency.	Serious: intervention combines home exercise, community kiosks, and optional telerehabilitation monitoring.	Not serious to serious: large treatment group, but non-randomized design limits precision interpretation.	Not assessable.	Observational supportive evidence; starts low and likely downgrades to very low for causal certainty.
Calabrò et al. 2023 [44]	Multicenter RCT; RoB 2	Serious/some concerns: assessor blinding reported and randomization used, but small sample, non-blinded	May increase inconsistency in global functional pooling because	Serious: SABI mixed vascular/traumatic etiology and combined motor-cognitive	Serious: n = 40 and wide uncertainty around motor-specific effects.	Not assessable.	Contributes to global functional/ADL GRADE only with caution; likely low/very low.

		treatment, multiple outcomes, and multidomain intervention.	diagnosis/intervention differ from stroke-only trials.	VRRS intervention.			
Carey et al. 2007 [45]	Randomized trial with crossover component; RoB 2	Serious: small sample, post-test/follow-up losses in one arm, no clear conventional comparator, and no clear superiority over movement practice.	Not suitable for broad pooled TR effectiveness; content comparison.	Very serious: both groups received home computerized telerehabilitation, so it tests tracking vs simple movement content.	Serious: n = 20 at post-test; short 2-week intervention.	Not assessable.	Delivery/content evidence only; very low for primary TR effectiveness.
Chae et al. 2020 [46]	Prospective comparative study; ROBINS-I	Very serious: non-randomized groups recruited at different centers, small control group, and substantial differential attrition.	Not pooled with RCTs.	Serious: wearable HBR monitoring compared with handout plus calls; technology-recognition outcome mixed with clinical outcome.	Very serious: analyzed n = 17 vs n = 6 and 100% control attrition by follow-up.	Not assessable.	Narrative/sensitivity evidence; very low certainty for causal effect.
Chantanachai et al. 2025 [47]	Double-blind sham-controlled RCT; RoB 2	Some concerns/low: robust blinding and no dropout, but small sample and adjunct neuromodulation design.	Not pooled for TR effectiveness; adjunct-specific comparison.	Very serious for primary TR effectiveness: both groups received identical tele-exercise and differed by active vs sham tDCS.	Serious: n = 30; effects limited to UEMS and follow-up.	Not assessable.	Adjunct tDCS evidence; not used to rate telerehabilitation vs non-telerehabilitation certainty.
Chen et al. 2017 [48]	Assessor-blinded RCT; RoB 2	Some concerns: allocation concealed and assessors/statisticians blinded; participants/caregivers/therapists unblinded as expected.	May be consistent with equivalence/non-inferiority interpretation but differs from Chen et al. 2020 [49] superiority signal.	Not serious to serious: direct home TR vs outpatient rehabilitation, but both arms included ETNS.	Serious: moderate sample and no significant between-group effects.	Not assessable.	Contributes to TR vs conventional rehabilitation GRADE; likely low/moderate depending on pooled precision.
Chen et al. 2020 [49]	Randomized controlled trial; RoB 2	Some concerns: assessor/data-analyst blinding reported, but unequal treatment exposure and LOCF handling require caution.	Potentially inconsistent with neutral trials because it showed FMA superiority at 12 weeks.	Not serious: subacute stroke, home-based motor TR vs conventional rehabilitation, validated FMA/MBI outcomes.	Serious: n = 52 and effect not maintained at 24-week follow-up.	Not assessable.	Important RCT contributor; certainty likely low/moderate after imprecision and RoB considerations.
Chen et al. 2021 [50]	Prospective case-control pilot randomized study; RoB 2	Serious: pilot design, small sample, unclear concealment details, and simulated-home hospital setting.	May increase heterogeneity in balance/mobility domain.	Serious: intervention occurred in hospital-based simulated home environment rather than true home delivery.	Serious: n = 30 and no significant between-group difference.	Not assessable.	Sensitivity/narrative evidence; low to very low certainty.
Chumbler et al. 2012 [51]	Multisite single-blinded RCT; RoB 2	Serious/some concerns: blinded outcome interview, but missing baseline/follow-up data, telephone self-report, and small veteran sample.	May be inconsistent with other global functional estimates due to neutral primary outcomes and positive secondary disability outcomes.	Not serious to serious: clinically relevant home TR, but veteran sample and self-reported functional outcomes limit generalizability.	Serious: primary outcomes not statistically significant; limited sample.	Not assessable.	Contributes to global functional/disability certainty; likely low.
Chung et al. 2020 [52]	Single-blind pilot RCT; RoB 2	Serious: self-reported adherence by telephone, pilot design, unblinded participants, and functional gains secondary.	Not assessable; single delivery-format study.	Serious: video-guided vs paper home exercise is an adherence/delivery-format question, not a full therapist-led TR efficacy comparison.	Serious: n = 56 and secondary functional outcomes limited.	Not assessable.	Implementation/adherence evidence; low to very low for motor recovery certainty.
Cikajlo et al. 2012 [53]	Pilot controlled comparative study; ROBINS-I	Serious: non-randomized VRBT cohort with a control group shared from previous work; residual confounding by cohort, baseline severity and care context remains likely.	Not assessable at study level; not pooled with randomized evidence.	Serious: directly relevant to stroke balance telerehabilitation, but smart-home/standing-frame setup and non-equivalent control limit applicability.	Serious: only 6 telerehabilitation participants and small/non-randomized effect-level evidence.	Not assessable; single small observational comparison.	Narrative/sensitivity evidence; starts low as non-randomized evidence and likely downgrades to very low.
Conroy et al. 2018 [54]	Randomized single-blind controlled trial; RoB 2	High: randomized design with blinded outcome assessment, but very high and differential attrition produced small imbalanced analyzed	Potentially serious if combined with other MS home-exercise trials because disability level, adherence and	Serious: web-based asynchronous HAT supported self-directed exercise, not live supervised telerehabilitation;	Serious to very serious: small completed sample and no clear between-group benefit for primary walking	Not assessable (k < 10).	Sensitivity evidence only; very low to low certainty for MS walking/balance outcomes.

		groups.	intervention intensity varied.	population was predominantly progressive MS.	outcome.		
Coulter et al. 2017 [55]	Pilot RCT; RoB 2	High: small 2:1 pilot RCT with unblinded outcome assessment and no published protocol/registry.	Not assessable as single SCI web-physio pilot unless combined narratively with SCI implementation studies.	Serious: SCI web-based physiotherapy was feasible, but heterogeneity of injury level and usual-care comparator limits causal certainty.	Very serious: n = 24 randomized and 21 analyzed; estimates were imprecise and mainly nonsignificant.	Not assessable (k < 10).	Feasibility evidence; very low certainty for SCI physical-capacity effectiveness.
Cramer et al. 2019 [56]	Assessor-blinded noninferiority RCT; RoB 2	Low: robust multicenter randomization, assessor blinding, matched dose/intensity, ITT analysis and prespecified noninferiority framework.	Not serious within the direct comparison; single large trial with internally consistent analyses.	Not serious: adult post-stroke upper-limb motor rehabilitation, home TR versus dose-matched in-clinic therapy, validated FM outcome.	Not serious for noninferiority: n = 124 and the adjusted CI stayed within the prespecified noninferiority margin.	Not assessable (single study; k < 10).	Important delivery-model evidence; moderate to high certainty for noninferiority of home TR versus in-clinic therapy.
Dastan et al. 2025 [57]	Single-blind randomized study; RoB 2	Some concerns: independent randomization and assessor procedures were reported, but small sample, attrition and nonblinded delivery remain concerns.	Not assessable at study level; may add heterogeneity in MS upper-extremity delivery-mode evidence.	Very serious for primary TR effectiveness: both groups received telerehabilitation; comparison is synchronous versus asynchronous delivery.	Serious: n = 25 analyzed and estimates are not sufficient for definitive efficacy certainty.	Not assessable (k < 10).	Delivery-mode evidence only; low to very low certainty for comparative delivery conclusions.
van den Berg et al. 2016 [58]	Pragmatic randomized proof-of-concept trial; RoB 2	Some concerns: allocation concealment and assessor blinding were strong, but self-reported primary outcome, unblinded delivery and per-protocol emphasis limit certainty.	Serious if pooled broadly; ITT primary mobility was neutral while per-protocol secondary outcomes were more favorable.	Serious: complex intervention combined caregiver-mediated exercises, app support, Fitbit feedback, home visits and videoconferencing, not isolated telerehabilitation.	Serious: proof-of-concept sample and confidence intervals around mobility/ADL outcomes were wide.	Not assessable (k < 10).	Hybrid e-health/caregiver-mediated evidence; low certainty for patient mobility/ADL and caregiver outcomes.
Deng et al. 2012 [59]	Pilot RCT; RoB 2	Some concerns: randomized pilot with blinded testers, but allocation concealment was limited, groups were small and baseline imbalance was present.	Not assessable at study level; not pooled for broad TR effectiveness.	Very serious for primary TR effectiveness: both groups received home telerehabilitation and differed by complex versus simple ankle task content.	Very serious: n = 16, no follow-up and multiple gait/fMRI endpoints.	Not assessable (k < 10).	Delivery/content evidence only; very low certainty for comparative content effects.
van der Kolk et al. 2019 [60]	Double-blind randomized controlled trial; RoB 2	Low: independently generated web randomization, patient masking to alternative intervention, masked assessors/analysts and ITT sensitivity analyses.	Not serious within this comparison; single large trial with coherent primary and adherence analyses.	Not serious: mild PD, home aerobic exergaming with remote supervision compared with active stretching control; directly relevant to remotely supervised motor exercise.	Not serious to serious: n = 125 with CI favoring intervention for off-state MDS-UPDRS, but single-center evidence warrants caution.	Not assessable (single study; k < 10).	Moderate certainty for home remotely supervised aerobic exercise improving PD motor signs.
Dogan et al. 2023 [61]	Assessor-blinded RCT; RoB 2	Some concerns: assessor-blinded randomization, but small sample, unblinded delivery and attrition in the TR arm.	Serious if combined with other MS technology trials because V-TOCT and mobile-app TR differ in setting, feedback and intervention content.	Very serious for primary TR effect: active technology-supported comparator; the trial compares mobile-app TR with clinic-based VR TOCT.	Serious: n = 32 analyzed and domain-specific estimates are small-study evidence.	Not assessable (k < 10).	Active-comparator/delivery evidence; low to very low certainty for TR versus VR-supported TOCT.
Eldemir et al. 2023 [62]	Evaluator-blind RCT; RoB 2	Some concerns: minimization/randomization and blinded evaluation were reported, but nonblinded delivery and multiple outcomes remain concerns.	Not assessable at study level; may be pooled only with carefully matched PD upper-extremity comparisons.	Not serious to serious: direct PD upper-extremity TOCT-TR plus home exercise versus home exercise, but the effect includes added therapy dose/content.	Serious: small RCT (about 30 PwPD) with multiple upper-extremity outcomes.	Not assessable (k < 10).	Primary candidate for PD upper-extremity TR; low certainty pending pooled evidence.
Eldemir et al. 2024 [63]	Assessor-blinded RCT; RoB 2	Some concerns: minimization and blinded assessment were reported, but waitlist/no-treatment comparator, no participant blinding and multiple self-reported outcomes introduce	Not assessable at study level; Pilates-TR differs clinically from other MS telerehabilitation approaches.	Serious: Pilates-based synchronous TR versus waitlist tests an exercise modality and comparator expectation effects, not TR delivery alone.	Serious: n = 30 and many outcomes; precision remains limited.	Not assessable (k < 10).	Primary candidate for MS physical performance versus waitlist; low certainty due comparator and imprecision.

		concerns.					
Fjeldstad-Pardo et al. 2018 [64]	Randomized three-arm feasibility pilot; RoB 2	Some concerns: evaluator-blinded pilot with limited detail on sequence/concealment and small groups.	Serious: intervention effects varied across HEP, TR and in-person PT arms and across gait, balance and PRO outcomes.	Serious: feasibility design with customized HEP, remote PT or in-person PT; not a definitive efficacy trial.	Serious to very serious: n = 29 and multiple outcomes with limited power.	Not assessable (k < 10).	Feasibility/sensitivity evidence; very low to low certainty for MS gait/balance effectiveness.
Fluet et al. 2024 [65]	Randomized controlled trial; RoB 2	Some concerns: randomized with blinded clinical assessors, but concealment was limited and dropouts were related to usability/enjoyment in some cases.	Not assessable for primary TR effectiveness; not pooled with usual-care comparisons.	Very serious: both arms received home virtual rehabilitation; comparison tested scaffolding versus adaptive algorithms, not telerehabilitation effect.	Serious: n = 33 and adherence/motivation primary outcomes, with motor outcomes secondary.	Not assessable (k < 10).	Delivery/adherence evidence only; not rated for primary telerehabilitation effectiveness.
Flynn et al. 2021 [66]	Randomized pilot trial; RoB 2	Some concerns: concealed randomization and blinded outcome assessors, but pilot design and unblinded participants/therapists limit certainty.	Not assessable as single feasibility comparison; not intended for pooled efficacy conclusions.	Very serious for primary TR effectiveness: both groups first received centre-based exercise and randomization compared follow-on home monitoring versus centre-based continuation.	Serious: n = 40 and feasibility outcomes were primary; clinical outcomes were secondary.	Not assessable (k < 10).	Feasibility/delivery-model evidence; low to very low certainty for clinical effectiveness.
Frevel et al. 2015 [67]	Randomized controlled study; RoB 2	High: randomization by lots was reported, but assessor was not blinded and sample was very small.	Serious: active comparator hippotherapy differs substantially from internet-based balance/strength training.	Serious: direct for MS balance training access, but comparator, therapist contact and intervention mechanisms are heterogeneous.	Very serious: n = 18 randomized, n = 16 completed, and confidence in effect estimates is limited.	Not assessable (k < 10).	Sensitivity active-comparator evidence; very low certainty.
Gandolfi et al. 2017 [68]	Multicenter single-blind RCT; RoB 2	Some concerns: multicenter randomized design with blinded assessors, but participants/therapists were unblinded and reporting flexibility remains possible.	Serious if pooled with other PD balance trials because comparators and VR/sensory-integration mechanisms differ.	Serious for broad TR effectiveness: home VR telerehabilitation was compared with in-clinic SIBT, so results address delivery plus modality differences.	Serious: single trial, modest sample and outcome-specific effects varied between BBS and DGI.	Not assessable (k < 10).	Delivery/sensitivity evidence for PD balance; low certainty.
Garcia et al. 2022 [69]	Feasibility crossover trial; RoB 2	High: small feasibility crossover with limited concealment/blinding details and substantial incomplete follow-up.	Not assessable; single feasibility study and not poolable.	Serious: chronic post-stroke home Muvity versus conventional self-training is relevant, but intervention is feasibility/engagement oriented.	Very serious: 10 randomized, 6 completed the full plan; no statistically robust estimates.	Not assessable (k < 10).	Narrative feasibility evidence; very low certainty.
Ge et al. 2024 [70]	Randomized controlled trial; RoB 2	Some concerns: randomization and blinded assessment/analysis reported, but participants and therapists were unblinded and supervision intensity differed between HPT and TR.	Not assessable at study level; may add heterogeneity to PD home-exercise delivery comparisons.	Serious for telerehabilitation efficacy: the comparison is TR versus in-person home physical therapy, not TR versus usual/no care; subgroup age effects also limit directness.	Serious: although sample size was comparatively large, the key between-group findings were age-dependent and adherence differed between groups.	Not assessable (k < 10).	Delivery-model evidence; certainty contribution likely low for TR versus HPT and indirect for primary telerehabilitation effectiveness.
Ginis et al. 2016 [71]	Pilot RCT; RoB 2	High: randomization was stratified, but assessors were not blinded and outcomes were performance-based gait/balance measures.	Not assessable at study level; effects were mostly comparable between groups with selected balance/QoL signals.	Serious: wearable/smartphone feedback plus weekly visits was compared with personalized gait advice, so the effect is technology-feedback specific rather than broad TR access.	Serious: pilot sample with 40 randomized participants and limited power for between-group effects.	Not assessable (k < 10).	Wearable gait-feedback TR evidence; very low to low certainty contribution, retained mainly for sensitivity/narrative synthesis.
Goffredo et al. 2023 [72]	Multicenter RCT; RoB 2	Some concerns: assessor blinding and centralized envelope randomization were reported, but participants/therapists were	Potentially not serious if analyzed with closely related PD balance TR trials, but intervention/comparator	Serious: VRRS-based TR was compared with at-home booklet-guided conventional activities, which tests both	Serious: n = 97 analyzed, but Mini-BESTest improvement was modest and below the MCID for	Not assessable (k < 10).	Primary/sensitivity evidence for PD balance; likely low to moderate certainty depending on pooled consistency and precision.

		unblinded and the monitoring/feedback intensity differed.	content differs from in-clinic SIBT studies.	technology feedback and remote monitoring.	many participants.		
Guo et al. 2023 [73]	Randomized controlled pilot trial; RoB 2	High: randomized parallel design, but allocation concealment and assessor blinding were unclear; Fugl-Meyer outcomes were scored by rehabilitation physicians in an obvious device trial.	Not assessable at study level; inpatient device-supported trial differs from home/community TR trials.	Serious: conducted in rehabilitation institutions with routine PT and drug treatment, so indirect for unsupervised home/community telerehabilitation.	Serious: pilot device trial; total Fugl-Meyer and upper-extremity scores favored the system, while lower-extremity difference was not significant.	Not assessable (k < 10).	Sensitivity/device evidence only; very low to low certainty for causal TR effectiveness.
Hernandez et al. 2022 [75]	Evaluator-blinded RCT; RoB 2	Some concerns: sealed-envelope block randomization and blinded evaluators were used, but the VR arm had remote therapist monitoring and progression while GRASP was unsupervised.	Not serious to serious: findings align with equivalence/comparable-effect interpretation but responder signals depended on active use.	Serious: chronic stroke VR system versus GRASP tests serious-game engagement and monitoring rather than telerehabilitation versus usual care.	Serious: n = 51 completed, no between-group superiority, and clinically meaningful change depended on active-use subgroup.	Not assessable (k < 10).	Sensitivity/active-comparator evidence; low certainty for comparative effectiveness and useful for engagement/adherence interpretation.
Hartung et al. 2025 [74]	Multicenter waitlist-controlled RCT; RoB 2	Some concerns: stratified block randomization was described, but the trial was retrospectively registered, participants were unblinded and missing data were imputed.	Serious if grouped with motor-recovery studies because the primary outcome was physical activity behavior rather than motor impairment/function.	Serious: internet-based exercise and PA promotion targets steps/MVPA/competence; it is adjacent to motor telerehabilitation rather than direct motor recovery therapy.	Serious: no significant primary effect on steps/day and multiple secondary outcomes in a modest sample.	Not assessable (k < 10).	Adjacent PA-promotion evidence; low certainty for PA behavior outcomes, not primary motor-effect evidence.
Hong et al. 2025 [76]	Retrospective cohort non-inferiority; ROBINS-I	Serious: retrospective historical grouping by completed intervention rather than randomization; disease duration differed significantly across groups, and only partial ANCOVA adjustment was reported; assessor blinding and adherence capture were not clearly reported.	Not assessable at study level; not pooled with randomized evidence.	Serious: directly relevant to post-stroke upper-limb telerehabilitation, but the retrospective single-center Chinese context, active-comparator/non-inferiority delivery design and absence of long-term follow-up limit broader applicability.	Serious: n = 79 analyzed with smaller Tele-Rehab group (n = 23); the FMA-UE confidence interval supported non-inferiority but remained relatively wide, and follow-up was limited to the 3-week intervention endpoint.	Not assessable; single observational study.	Non-randomized narrative/sensitivity evidence; starts low and likely downgrades to very low because of serious risk of bias and imprecision.
Huijgen et al. 2008 [77]	Randomized multicentre clinical trial; RoB 2	Some concerns: pragmatic 2:1 allocation linked to HCAD availability, unclear concealment, unblinded delivery and unclear assessor blinding.	Serious: not pooled as a single homogeneous RCT because stroke, TBI and MS were combined and diagnosis-specific effects were reported.	Serious: mixed neurological population and feasibility/equivalence framing limit directness for diagnosis-specific TR effectiveness.	Serious: modest sample after losses; equivalence bounds and subgroup analyses limit precision.	Not assessable (k < 10).	Sensitivity/narrative contribution; likely low to very low certainty for causal effectiveness.
Jarbandhan et al. 2022 [78]	Randomized controlled pilot study; RoB 2	High: no allocation blinding in practice, one investigator executed multiple trial functions, and intervention attrition was substantial.	Not assessable at study level; contributes to heterogeneous stroke mobility evidence.	Serious: LMIC home-based semi-supervised physiotherapy with telephone support is relevant but context-specific.	Very serious: pilot sample, 2:1 allocation and 70% intervention completion.	Not assessable (k < 10).	Feasibility/sensitivity evidence; very low certainty contribution for effectiveness.
Johnson et al. 2020 [79]	Assessor-blinded RCT; RoB 2	Some concerns: robust assessor-blinded design, but participants were unblinded and attention/dose differed from usual care.	Potentially serious if pooled with heterogeneous upper-limb VR/TR trials.	Not serious to serious: chronic stroke community-based VR telerehabilitation is directly relevant but delivered in support-group premises rather than unsupervised home.	Serious: moderate sample and domain-specific uncertainty, despite no adverse events.	Not assessable (k < 10).	Primary/sensitivity contributor to chronic stroke upper-limb evidence; likely low to moderate pending pooling.
Johnson et al. 2024 [80]	Pilot randomized controlled trial; RoB 2	High: unblinded delivery, very small groups and discharge outcomes collected by treating therapists.	Not assessable at study level.	Serious: feasibility comparison of clinic+TR, TR-only and usual care in early-to-moderate PD rather than a	Very serious: n = 20 randomized and clinical outcomes exploratory.	Not assessable (k < 10).	Feasibility evidence only; very low certainty for comparative motor outcomes.

				definitive efficacy trial.			
Kintrilis et al. 2024 [81]	Randomized controlled study; RoB 2	Some concerns: random assignment and blinded testing reported, but sequence/concealment and missing-data handling were incompletely described.	Serious if pooled because strength training, in-person delivery and teleconference delivery estimate related but distinct questions.	Serious: resistance-band strength training after acute stroke is modality-specific and not a broad TR effect.	Serious: no clear powered primary motor outcome and several outcomes were tested.	Not assessable (k < 10).	Delivery-mode/sensitivity contribution; low certainty for teleconference strength training equivalence.
Kowalczewski et al. 2011 [82]	Blinded randomized crossover trial; RoB 2	Some concerns: block-randomized crossover with blinded ARAT scoring, but small sample, unblinded treatment and possible period/carryover issues.	Not pooled for primary TR effectiveness because both arms used in-home teletherapy.	Very serious for primary TR-vs-control certainty; comparison tests ReJoyce/FES content versus conventional tele-ET.	Serious: small sample of hands/participants, despite complete follow-up.	Not assessable (k < 10).	Delivery/content evidence only; not used for primary TR certainty.
Lee et al. 2022 [83]	Pilot RCT/non-inferiority; RoB 2	Some concerns: computer randomization and sealed envelopes reported, but very small sample, unclear assessor blinding and analysis-set ambiguity.	Not assessable at study level.	Serious: remote dance therapy added to conventional PT is a modality-specific adjunct intervention.	Very serious: n < 20 and pilot non-inferiority interpretation.	Not assessable (k < 10).	Pilot/sensitivity contribution; very low to low certainty for trunk-control outcomes.
Lee et al. 2025 [84]	Prospective parallel single-blind pilot RCT; RoB 2	Some concerns: concealed randomization and blinded assessment were reported, but unblinded delivery and pilot sample remain limitations.	Not assessable at study level.	Serious: inpatient pandemic-era TR plus regular rehabilitation may not generalize to outpatient/home TR.	Very serious: n = 24 and no clear between-group differences.	Not assessable (k < 10).	Pilot inpatient TR evidence; very low to low certainty.
Lin et al. 2014 [85]	Multisite blocked-randomized pilot study; RoB 2	Some concerns: computer randomization and blinded rater reported, but pilot size and unblinded delivery remain concerns.	Not assessable at study level; not pooled with outpatient/home trials unless carefully stratified.	Serious: long-term care facility group telerehabilitation differs from individual home-based TR.	Serious: n = 24 and one tele-group dropout; no sample-size calculation.	Not assessable (k < 10).	Pilot/sensitivity evidence for LTCF balance TR; low to very low certainty.
Llorens et al. 2015 [86]	Single-blind RCT; RoB 2	Some concerns: concealed randomization and blinded assessment were used, but delivery was unblinded and this is a delivery-setting comparison.	Not serious to serious; one of the more directly comparable home-versus-clinic VR balance studies.	Serious for broad TR effectiveness because both arms used the same VR system with different settings.	Serious: n = 30, although results were consistent across several balance scales.	Not assessable (k < 10).	Delivery-model evidence; low to moderate certainty for home VR being comparable to in-clinic VR.
Masbernat-Almenara et al. 2026 [87]	Single-blind two-arm pilot RCT; RoB 2	Some concerns: allocation concealment and blinded assessment were used, but age imbalance and very small pilot sample limit certainty.	Not assessable at study level.	Very serious for primary TR efficacy because both groups received CSE telerehabilitation and only delivery format differed.	Very serious: n = 12 and exploratory outcomes.	Not assessable (k < 10).	Delivery-format feasibility evidence; not primary TR-vs-control certainty.
Mulder et al. 2024 [88]	Multicentre observer-blinded RCT; RoB 2	Some concerns: off-site minimization and observer blinding were strong, but unblinded dyads and early futility stopping/underpowering limit certainty.	Serious if pooled with other stroke mobility interventions because caregiver-mediated dose and TR platform are hybrid components.	Serious: estimates blended caregiver-mediated exercises, telerehabilitation support and usual-care transition context.	Serious: underpowered trial with 37 analyzed dyads and wide uncertainty around the primary mobility outcome.	Not assessable (k < 10).	Hybrid caregiver-mediated TR evidence; low certainty for mobility and psychosocial outcomes.
Nuic et al. 2024 [89]	Multicenter single-blind phase II RCT; RoB 2	Some concerns: RedCap randomization and blinded SWST scoring were used, but participants were unblinded to active full-body versus seated gaming.	Not assessable at study level; may align with PD exergaming evidence but outcome effects were mixed.	Serious: tailored exergaming without supervision targets a specific PD subgroup with dopa-resistant gait/balance disorders.	Serious: n = 50 phase-II trial; primary SWST difference was not significant, although responder and clinical-scale signals favored active training.	Not assessable (k < 10).	Sensitivity evidence for PD exergaming; low certainty pending pooled/domain synthesis.

Ortiz-Gutiérrez et al. 2013 [90]	Controlled preliminary study; ROBINS-I	Serious: intervention allocation was based partly on access/availability to conventional therapy and not fully random.	Not formally pooled with RCTs; cross-study inconsistency not estimable.	Serious: MS balance/postural-control evidence is relevant but nonrandomized, technology-specific and based on CDP/SOT endpoints.	Serious: n = 50 and multiple sensory-ratio outcomes; precision limited for causal claims.	Not assessable (single non-randomized study; k < 10).	Non-randomized sensitivity evidence; starts low and downgrades to very low for causal certainty.
Pagliari et al. 2024 [91]	Multicentre rater-blinded RCT; RoB 2	Some concerns: adequate stratified block randomization and blinded assessors, but unblinded delivery, balanced attrition and multiple outcomes.	Not assessable at study level; contributes to heterogeneous MS TR evidence.	Not serious for MS home-based VRRS telerehabilitation vs home conventional rehabilitation.	Serious: 60 analyzed participants and single-trial/domain-specific effects.	Not assessable; single study within this outcome contribution.	RCT evidence; certainty contribution downgraded mainly for risk of bias/imprecision.
Paul et al. 2014 [92]	Randomized controlled pilot study; RoB 2	Some concerns: limited concealment detail, unblinded participants and unclear assessor blinding.	Not assessable at study level.	Mostly not serious for web-based physiotherapy in moderately affected MS, but comparator was usual care/advice.	Serious: pilot n = 30 with small effects and no definitive efficacy power.	Not assessable.	Pilot RCT; contributes low-certainty supportive evidence only.
Paul et al. 2019 [93]	Multicentre single-blind feasibility RCT; RoB 2	Some concerns: robust randomization/blinded assessment but unblinded delivery and attrition across follow-up.	Not assessable at study level; outcome direction mixed.	Some concern for indirectness because comparator was active printed home exercise rather than no rehabilitation.	Serious: feasibility trial, adherence variability and imprecise estimates.	Not assessable.	Feasibility RCT; certainty contribution limited mainly by imprecision and active-comparator indirectness.
Pavan et al. 2024 [94]	Single-blind randomized two-arm clinical trial; RoB 2	Some concerns: randomized design, but randomization details limited, unblinded delivery and self/perceived disability outcome.	Not assessable; single delivery-model trial.	Very serious for primary TR efficacy because both groups received telerehabilitation and the comparison was robotic vs non-robotic TR.	Serious: n = 30 and multiple domains.	Not assessable.	Delivery-model evidence; does not directly rate telerehabilitation vs conventional/usual care certainty.
Petracca et al. 2024 [95]	Nonrandomized interventional comparative study; ROBINS-I	Serious: temporal allocation by COVID-era feasibility, residual confounding and differential discontinuation.	Not assessable at study level.	Some concern: directly relevant to MS TR vs onsite rehabilitation, but pandemic-era allocation limits causal interpretation.	Serious: n = 51 completers and nonrandomized comparison.	Not assessable.	Nonrandomized evidence starts low and is likely very low for causal effectiveness.
Picelli et al. 2026 [96]	Assessor-blinded non-inferiority RCT; RoB 2	Some concerns: strong allocation/complete follow-up/blinded assessment, but participants and therapists were unblinded.	Not assessable; single study for integrated multi-domain TR.	Not serious for synchronous multi-domain TR vs conventional outpatient therapy in subacute stroke.	Serious: one RCT and wide non-inferiority CI, despite complete follow-up.	Not assessable.	Methodologically strong RCT; certainty contribution mainly limited by single-study imprecision.
Piron et al. 2009 [97]	Randomized single-blind controlled trial; RoB 2	Some concerns: concealed envelopes and blinded neurologist, but unblinded delivery and limited protocol/reporting detail.	Not assessable at study level.	Not serious for VRRS.net telerehabilitation vs conventional upper-limb therapy after stroke.	Serious: n = 36 and one-month follow-up.	Not assessable.	Small RCT; contributes low-to-moderate certainty support for upper-limb motor outcomes.
Prukviwat et al. 2026 [98]	Patient-preference nonrandomized controlled pilot; ROBINS-I	Serious: self-selection into tele vs hospital groups creates major confounding by preference, access and motivation.	Not assessable at study level.	Some concern: relevant to PD tele vs hospital rehabilitation, but patient preference and setting differences reduce directness.	Serious: pilot n = 46 and effects below MCID for BBS.	Not assessable.	Nonrandomized pilot; certainty starts low and is downgraded for serious bias/imprecision.
Pastana Ramos et al. 2023 [99]	Single-blind phase-2 RCT; RoB 2	Some concerns: independent web randomization and blinded assessment, but small pilot sample and unblinded delivery.	Not assessable at study level.	Not serious for remote individual PD rehabilitation in an underrepresented Amazonian setting, though generalizability may vary.	Very serious: n = 19 and feasibility-focused design.	Not assessable.	Pilot RCT; certainty contribution remains low because of imprecision.
Salgueiro et al. 2022a [100]	Single-blind preliminary RCT; RoB 2	Some concerns: randomization reported, assessor blinded, but concealment limited, low app adherence and pandemic-related assessment constraints.	Not assessable at study level.	Some concern: app-guided core-stability add-on to conventional therapy; not standalone TR.	Serious: n = 30 and preliminary estimates.	Not assessable.	Preliminary RCT; certainty limited by imprecision and adherence.
Salgueiro et al. 2022b [101]	Prospective controlled extension; ROBINS-I	Serious: nonrandomized extension, prior-treatment confounding,	Not assessable at study level.	Some concern: clinically relevant post-discharge app	Serious: follow-up n = 38 with AppG attrition.	Not assessable.	Nonrandomized extension; certainty starts low and is likely

		variable usual care and attrition.		TR, but not randomized and intervention was an add-on after prior CSE exposure.			very low for causal inference.
Saygili et al. 2024 [102]	Evaluator-blind RCT; RoB 2	Some concerns: blinded assessment and complete follow-up, but concealment detail limited and Tele-CIMT delivery unblinded/intensive.	Not assessable at study level.	Not serious for Tele-CIMT plus home exercise vs home exercise in stroke upper-limb recovery.	Serious: n = 18 despite large reported interactions.	Not assessable.	Small RCT; promising but certainty downgraded for imprecision.
Saywell et al. 2021 [103]	Assessor-blinded parallel RCT; RoB 2	Some concerns: strong randomization/blinding of assessors, but self-reported primary outcome and adherence-sensitive effects.	Not assessable at study level.	Not serious for pragmatic post-discharge stroke TR using phone/text/limited visits.	Serious: CI crossed null in ITT and per-protocol findings were adherence dependent.	Not assessable.	RCT evidence; certainty downgraded for risk of bias and imprecision.
Sheehy et al. 2025 [104]	Assessor-blinded feasibility RCT; RoB 2	Some concerns: concealed randomization and blinded assessment, but feasibility design and unblinded NIVRT vs iPad control.	Not assessable at study level.	Some concern: active control addressed cognition/fine motor rather than equivalent physical rehabilitation.	Very serious: n = 20 and feasibility objective, not powered for efficacy.	Not assessable.	Feasibility RCT; very limited contribution to outcome certainty.
Simpson et al. 2025 [105]	Multicenter assessor-blinded waitlist RCT; RoB 2	Some concerns: central randomization and objective wearable primary outcome, but waitlist design and valid-wear/missing data issues.	Not assessable at study level.	Not serious for virtual upper-limb program vs usual care/waitlist after stroke; outcome directly measures real-world arm activity.	Serious: moderate sample and CI close to the null.	Not assessable.	RCT evidence; certainty limited mainly by unblinded waitlist design and imprecision.
Skelly et al. 2026 [106]	Randomized controlled feasibility trial; RoB 2	Some concerns: randomized and blinded outcome assessment, but feasibility design, baseline imbalance, attrition/protocol exclusions and unblinded delivery.	Not assessable at study level.	Some concern: early Parkinson disease, physician-advice comparator and feasibility context.	Very serious: n = 40 feasibility study with exploratory effect sizes.	Not assessable.	Feasibility RCT; certainty contribution is very limited pending definitive trial.
Song et al. 2018 [107]	Single-blinded RCT; RoB 2	Some concerns: offsite randomization and blinded assessment, but unblinded unsupervised intervention, attrition and post-hoc subgroup findings.	Not assessable at study level; results varied by severity in exploratory analysis.	Some concern: home exergame step training is a specific unsupervised VR intervention rather than therapist-led TR.	Serious: modest sample and incomplete intervention/falls data.	Not assessable.	RCT evidence for exergaming; certainty limited by attrition, indirectness and imprecision.
Standen et al. 2017 [108]	Two-group feasibility RCT; RoB 2	High: substantial attrition/nonreceipt of intervention and incomplete final outcomes in a feasibility design.	Not assessable at study level.	Some concern: home VR arm system relevant to TR, but high support needs and feasibility context limit directness.	Very serious: final analyzed n = 18 and wide uncertainty.	Not assessable.	Feasibility evidence; very low certainty for efficacy.
Sun et al. 2025 [109]	Randomized controlled trial; RoB 2	Some concerns: concealed randomization and blinded assessors, but unblinded contact-intensive intervention and limited prospective reporting detail.	Not assessable at study level.	Not serious for post-discharge AIS telerehabilitation vs standard post-discharge care, though intervention includes psychological/education components.	Some concern: n = 200, but precision should be verified after effect extraction and contact-intensity imbalance.	Not assessable.	Larger RCT; certainty contribution potentially important but downgraded for performance/reporting concerns.
Swarnakar et al. 2023 [110]	Single-center double-blind pilot RCT; RoB 2	Some concerns: concealed randomization and ITT, but participant blinding is questionable and outcomes were remotely collected.	Not assessable at study level.	Not serious for SCI telerehabilitation during COVID-19, but pandemic context may limit generalizability.	Serious: n = 30 pilot trial.	Not assessable.	Small RCT; certainty downgraded for risk of bias and imprecision.
Thomas et al. 2017 [111]	Single-centre wait-list pilot RCT; RoB 2	Some concerns: strong randomization and retention, but unblinded delivery and partially unblinded physical assessments.	Not assessable at study level.	Some concern: home Nintendo Wii active gaming targets activity/vitality in ambulatory inactive MS and is indirect for impairment-level motor recovery.	Serious: pilot n = 30, feasibility focus and no definitive primary efficacy endpoint.	Not assessable.	Feasibility RCT; certainty contribution is low/very low and mainly informs acceptability/safety and feasibility.

Toh et al. 2025a [112]	Single-blind RCT; RoB 2	Some concerns: assessor blinding and stratified randomization, but unblinded device feedback and therapist monitoring.	Not assessable at study level.	Not serious for wearable-based upper-limb telerehabilitation versus sham/handout training after stroke.	Serious: n = 40 and effects concentrated mainly on FMA-UE; confidence remains outcome-specific.	Not assessable.	RCT contribution to stroke upper-limb wearable telerehabilitation; likely low to moderate pending pooling and RoB consensus.
Toh et al. 2025b [113]	Single-blind randomized crossover feasibility pilot; RoB 2	Some concerns: randomized crossover and blinded assessment, but small sample, attrition and unblinded treatment delivery.	Not assessable at study level.	Some concern: evaluates wearable-device delivery versus pictorial conventional therapy, partly a delivery/feedback question.	Serious: n = 12 crossover pilot with incomplete follow-up and several exploratory outcomes.	Not assessable.	Supportive feasibility evidence; certainty contribution limited by imprecision and crossover pilot design.
Uswatte et al. 2021 [114]	Proof-of-concept RCT/non-inferiority delivery comparison; RoB 2	Some concerns: randomization reported, but concealment/outcome blinding details limited and delivery was unblinded.	Not assessable at study level.	Serious for primary TR efficacy because the comparison is tele-health CIMT versus in-lab CIMT, not telerehabilitation versus usual care.	Serious: 24 randomized, 20 completers and only 14 with one-year MAL follow-up.	Not assessable.	Delivery-model evidence; does not drive primary telerehabilitation-vs-control certainty.
Vasconcellos et al. 2023 [115]	Single-blind RCT; RoB 2	High: adequate randomization/blinded objective measurement, but 33% non-adherence/losses with imputation.	Not assessable at study level.	Very serious for TR efficacy because both groups received remote home exercise; the contrast mainly tests trunk versus limb exercise content.	Serious: n = 28 with substantial non-adherence and null results.	Not assessable.	Delivery/content comparison only; very limited contribution to primary certainty.
Vloothuis et al. 2019 [116]	Observer-blinded multicenter RCT; RoB 2	Some concerns: robust randomization and blinded assessment, but unblinded caregiver-mediated behavioral intervention.	Not assessable at study level.	Some concern: e-health supported caregiver-mediated exercise adds caregiver coaching to usual care and is partly an early-supported-discharge model.	Serious: single n = 66 trial with no primary mobility/LOS effect.	Not assessable.	RCT evidence for caregiver-mediated e-health exercise; certainty downgraded mainly for imprecision and complex intervention indirectness.
Wang et al. 2024 [117]	Controlled comparative/quasi-random trial; RoB 2	High: odd/even allocation and incomplete concealment/reporting, with unblinded intensive internet/wearable intervention.	Not assessable at study level.	Some concern: clinically relevant post-discharge internet/wearable stroke rehabilitation, but biomarker outcomes and setting may limit generalizability.	Serious: single-center n = 80 and multiple outcomes; precision should not be inferred from p values alone.	Not assessable.	Narrative/sensitivity evidence only; certainty contribution very low/low due to high RoB.
Wilson et al. 2021 [118]	Parallel RCT with active comparator; RoB 2	Some concerns: concealed randomization and blinded assessment, but unblinded delivery and very small proof-of-concept sample.	Not assessable at study level.	Some concern: EDNA-22 home virtual rehabilitation was compared with active GRASP, not usual care.	Serious: 19 randomized and 17 completers; effects are exploratory.	Not assessable.	Active-comparator evidence; low-certainty contribution for home virtual upper-limb rehabilitation.
Wolf et al. 2015 [119]	Prospective multisite single-blind RCT; RoB 2	Some concerns: strong randomization and blinded assessment, but unblinded home robotic/device delivery and adherence self-report.	Not assessable at study level.	Some concern: robotic telerehabilitation plus HEP versus dose-matched HEP, an active-comparator and access-focused question.	Some concern/serious: n = 99, but extractable variance for key effect estimates may be incomplete for meta-analysis.	Not assessable.	Important RCT evidence; certainty contribution may be low/moderate but effect-level pooling depends on reconstructable variance.
Wu et al. 2020 [120]	Single-blind RCT; RoB 2	Some concerns: randomization and blinded assessors reported, but concealment details and unblinded collaborative care delivery remain concerns.	Not assessable at study level.	Not serious for acute stroke telerehabilitation exercise versus telephone/usual follow-up, although conducted in one Chinese hospital system.	Serious: n = 61 completers and single study; several repeated outcomes.	Not assessable.	RCT evidence for acute stroke motor/balance/ADL outcomes; likely low/moderate pending pooling.
Yokota et al. 2025 [121]	Retrospective observational PSM study; ROBINS-I	Serious: self-selection into TR and exclusions before matching leave residual confounding despite PSM.	Not assessable at study level.	Some concern: functionally independent stroke/TIA survivors using ergometer-based home TR; indirect for more disabled neurorehabilitation populations.	Serious: analyzed n = 21 TR and 63 matched controls; single observational estimate.	Not assessable.	Non-randomized evidence starts low and is downgraded for serious confounding/selection; narrative/sensitivity only.

Zheng et al. 2026 [122]	Phase-Ib assessor-blinded RCT; RoB 2	High for efficacy: concealed allocation and blinded assessment, but feasibility design with substantial attrition at post and follow-up.	Not assessable at study level.	Some concern: older adults with MS receiving remote aerobic/resistance exercise versus stretching; partially indirect for clinical telerehabilitation motor recovery.	Serious to very serious: n = 51 randomized, 36 post-intervention assessments and exploratory efficacy analyses.	Not assessable.	Feasibility RCT; certainty contribution remains low/very low for efficacy pending a phase-II trial.

Study-level GRADE note: These rows do not assign final certainty by themselves. They document risk-of-bias, indirectness, imprecision, inconsistency and publication-bias inputs that support the comparison-outcome judgments in Table S13.

Table S13. Supporting GRADE comparison/outcome considerations.

Comparison and outcome/time point	Studies and participants	Effect estimate/data basis	Risk of bias	Inconsistency	Indirectness	Imprecision	Publication bias and overall certainty
Telerehabilitation vs usual care/no additional therapy; upper-limb function, post-intervention	1 RCT: Adams et al. 2023 [38]; n = 18 completers. Non-randomized supportive evidence from Benvenuti et al. 2014 [43] and Chae et al. 2020 [46] not combined with RCT evidence.	Single-study effect only; no pooled estimate. Primary domain: FMUE/upper-limb motor function.	Serious. Rater-blinded randomized design, but small sample, attrition from randomized cohort, non-blinded treatment, and industry/intellectual-property conflict require caution.	Not assessable because one RCT contributes to the formal comparison-outcome judgment.	Not serious for the RCT question: adult stroke, home VR telerehabilitation with OT support, usual/customary-care comparator, validated upper-limb outcome.	Serious. Single small RCT; precision depends on one study even though the direction of effect favors telerehabilitation.	Not assessable (k < 10). Overall certainty: Low, downgraded for risk of bias and imprecision.
Telerehabilitation vs usual care/no additional therapy; global motor-functional/ADL outcomes, post-intervention to 6 months	3 RCTs: Asano et al. 2021 [40], Calabrò et al. 2023 [44], Chumbler et al. 2012 [51]; 216 randomized, approximately 186 analyzed at main endpoint.	Mixed validated functional outcomes: LLFDI/MBI/BI/Motor FONEFIM and related ADL or global motor-functional measures. Pooling only if clinically/statistically compatible.	Serious. Assessor blinding was reported in key studies, but small samples, incomplete follow-up, self-reported/telephone outcomes in some studies, non-blinded participants, and multidomain interventions raise concerns.	Serious. Direction and magnitude vary across studies; clinical heterogeneity includes stroke and severe acquired brain injury, different intervention intensities, and different functional scales.	Serious. Outcomes measure related but non-identical constructs: self-reported disability, ADL independence, global functional status, and mixed motor-cognitive rehabilitation effects.	Serious. Small number of studies; several estimates cross the null or are not robustly poolable by identical scale/time point.	Not assessable (k < 10). Overall certainty: Very low to Low; likely Low if restricted to clinically coherent RCT outcomes, Very low if pooled across broad functional constructs.
Telerehabilitation vs conventional/face-to-face rehabilitation; stroke motor function, balance/mobility, and ADL, post-intervention	3 RCT/pilot RCTs: Chen et al. 2017 [48], Chen et al. 2020 [49], Chen et al. 2021 [50]; n = 136 randomized/enrolled.	Validated outcomes including FMA, MBI, BBS, TUG and related motor-functional endpoints. Change-score data preferred when available.	Serious. Allocation and assessor blinding were generally reported, but participants/therapists were unblinded; Chen et al. 2020 [49] had unequal treatment exposure adjusted analytically; Chen et al. 2021 [50] was a simulated-home pilot.	Serious. Results range from comparable effects to superiority for selected FMA outcomes; chronicity and settings differ across studies.	Serious. One study used a simulated home environment and one combined telerehabilitation with ETNS; comparators differed between outpatient conventional rehabilitation and one-to-one physiotherapy.	Serious. Small k and modest total sample; confidence in domain-specific estimates remains limited.	Not assessable (k < 10). Overall certainty: Low, downgraded for risk of bias, inconsistency/indirectness, and imprecision.
Home VR/telerehabilitation vs active home or occupational-therapy comparator; upper-limb function, post-intervention/follow-up	3 randomized or feasibility randomized studies: Ballester et al. 2017 [42], Allegue et al. 2022 [39], Carey et al. 2007 [45]; small chronic-stroke samples.	Upper-limb outcomes including UE-FM/FMA-UE, CAHAI, Box and Block, Jebsen-Taylor, MAL and related measures. Not a single homogeneous pooled comparison unless restricted by outcome/time point.	Very serious. Small samples, feasibility designs, non-blinded or incompletely blinded outcome assessment, crossover components, and COVID-related protocol changes in Allegue et al. 2022 [39].	Serious to very serious. Interventions and comparators differ substantially; efficacy patterns are mixed and not consistently confirmatory.	Serious. These trials mainly evaluate VR-specific or training-content effects against active alternatives rather than the broad effect of telerehabilitation versus usual/no care.	Very serious. Small samples and many outcomes are underpowered or not directly poolable.	Not assessable (k < 10). Overall certainty: Very low for comparative effectiveness; useful primarily for narrative/sensitivity synthesis.
Delivery-model-only or adjunct remote-rehabilitation comparisons; engagement, balance, or	4 RCT/crossover/adjunct trials: Thielbar et al.	Not a primary telerehabilitation-vs-control effect. Outcomes address multiuser vs single-user VR, LSVT BIG	Serious. Designs are randomized but small, often unblinded, and some involve crossover/content	Not pooled. Clinical and methodological inconsistency is expected	Very serious for primary effectiveness: both arms generally	Serious to very serious because each comparison is small and not designed to	Not assessable (k < 10). Overall certainty: Not formally rated for primary

neuromodulation-related motor outcomes	2020 [37], Aytutuldu et al. 2024 [41], Carey et al. 2007 [45], Chantanachai et al. 2025 [47].	vs mobility training, tracking vs movement training, or active vs sham tDCS with identical tele-exercise.	comparisons rather than independent telerehabilitation effectiveness questions.	because the question differs across studies.	received remote/telerehabilitation or the comparison tested an adjunct rather than telerehabilitation itself.	support broad effect estimates.	telerehabilitation effectiveness; very low for exploratory delivery/adjunct conclusions.
Non-randomized telerehabilitation evidence; SCI functionality and stroke upper-limb home rehabilitation	3 non-randomized comparative studies: García-Rudolph et al. 2024 [36], Benvenuti et al. 2014 [43], Chae et al. 2020 [46].	Observational effect-level evidence only; not combined with randomized evidence for primary GRADE judgments.	Very serious. Historical/geographic/prospective non-randomized controls, residual confounding, selection bias, and differential attrition are major concerns despite matching or prospective assessment.	Not formally pooled. Cross-study heterogeneity is substantial across SCI vs stroke, inpatient-to-home teleSCI vs community/kiosk/wearable models, and outcome constructs.	Serious. Evidence is relevant to implementation and feasibility, but indirect for causal efficacy compared with randomized evidence.	Serious to very serious; some samples are large but observational, while others have very small controls or incomplete follow-up.	Not assessable (k < 10). Overall certainty: Very low for causal effectiveness; retained as narrative/contextual evidence only.
Video-guided vs paper-based home exercise delivery; adherence, self-efficacy, mobility gain after stroke discharge	1 pilot RCT: Chung et al. 2020 [52]; n = 56 analyzed.	Single-study delivery-mode evidence; primary outcome was adherence, with MFAC/MBI functional outcomes.	Serious. Single-blind randomized design, but adherence was self-reported by telephone, intervention could not be blinded, and pilot design limits robustness.	Not assessable because only one study contributes.	Serious for motor-recovery certainty because the comparison primarily tests delivery format and adherence rather than supervised telerehabilitation efficacy.	Serious. Single pilot study, limited sample size, and functional outcomes are secondary.	Not assessable. Overall certainty: Low to Very low; useful for implementation/adherence evidence, not confirmatory motor-effect evidence.
Home-based telerehabilitation vs dose-matched in-clinic rehabilitation; stroke upper-limb motor function, post-intervention	1 large assessor-blinded RCT: Cramer et al. 2019 [56]; n = 124 randomized.	Noninferiority effect basis: adjusted FM change difference 0.06 points favoring TR (95% CI -2.14 to 2.26); CI within prespecified noninferiority margin.	Not serious. Robust multicenter design, blinded assessment, high adherence and ITT sensitivity analyses.	Not assessable as pooled inconsistency because one trial contributes; internal sensitivity analyses were coherent.	Not serious for the delivery-model question: home TR directly compared with dose-matched in-clinic therapy.	Not serious to serious. Single trial, but adequately powered for the noninferiority question and CI supports noninferiority.	Not assessable (k < 10). Overall certainty: Moderate to High for noninferiority of TR versus in-clinic therapy; conservative rating Moderate because evidence is single-study.
Home/remotely supervised aerobic exercise vs active stretching control; Parkinson disease motor symptoms at 6 months	1 double-blind RCT: van der Kolk et al. 2019 [60]; n = 130 randomized, 125 primary analysis.	Adjusted between-group difference in off-state MDS-UPDRS motor score favored aerobic exercise by 4.2 points (95% CI 1.6 to 6.9).	Not serious. Randomization, masking, remote supervision, adherence capture and ITT sensitivity analyses were strong.	Not assessable because one primary trial contributes.	Not serious: mild PD, home-based exergaming/aerobic exercise with remote coaching versus active non-aerobic control.	Not serious to serious. Estimate excludes the null and exceeds prespecified clinical relevance, but evidence is single-center.	Not assessable (k < 10). Overall certainty: Moderate for remotely supervised home aerobic exercise in mild PD.
Task-oriented or modality-specific TR vs home exercise/waitlist; upper-extremity or physical performance in PD/MS	3 small RCTs: Eldemir et al. 2023 [62], Eldemir et al. 2024 [63], Dastan et al. 2025 [57]; approximately n = 85 randomized/analyzed across related but distinct questions.	Effect estimates are study-specific across 9-HPT/JHFT/grip strength, Pilates physical performance and N-HPT/JAMAR/AMSQ; no single homogeneous pooled estimate.	Serious. Blinded assessment was often used, but participants/therapists were unblinded, samples were small, and comparator expectations/delivery modes varied.	Serious. Direction generally favors active synchronous/supervised intervention, but populations, scales and comparator structures differ.	Serious. Evidence combines PD and MS, different intervention modalities, and delivery-mode or added-dose contrasts.	Serious. Small samples and many outcome domains limit precision.	Not assessable (k < 10). Overall certainty: Low for modality-specific TR improving upper-extremity/physical-performance outcomes.
Technology-supported TR versus active clinic-based VR/physiotherapy comparators; MS upper-limb/trunk or gait/balance outcomes	3 randomized/pilot studies: Dogan et al. 2023 [61], Fjeldstad-Pardo et al. 2018 [64], Frevel et al. 2015 [67]; approximately n = 79 randomized/enrolled.	Data basis: TIS/K-ICARS/ABILHAND/MMDT/kinematics, FGA/T25FW/BBS/PROs, and BBS/DGI/TUG/2MWT. Pooling not recommended without strict domain separation.	Serious to very serious. Assessor blinding varied; Frevel et al. was unblinded and very small; feasibility designs were common.	Serious. Results and constructs vary across balance, gait, trunk and upper-limb outcomes.	Serious. Active comparators and mechanisms differ substantially: mobile-app TR, in-person PT, hippotherapy or VR TOCT.	Serious to very serious. Small trials and outcome multiplicity dominate.	Not assessable (k < 10). Overall certainty: Very low to Low; use mainly as narrative/sensitivity evidence.
Web-based or remotely monitored exercise vs usual/routine home care; MS/SCI walking, balance or physical	2 pilot RCTs: Conroy et al. 2018 [54] and Coulter et al. 2017 [55];	Data basis: T25FW, 6MWT, BBS, 6MPT/6MWT and QoL/symptom outcomes; no robust pooled estimate.	High/serious. Major attrition in Conroy and unblinded measurement/no registry in	Serious. Different diagnoses, intervention platforms, supervision	Serious. MS and SCI evidence address related access models but not	Very serious. Small completed samples and CIs/effects not robust.	Not assessable (k < 10). Overall certainty: Very low for effectiveness;

capacity	n = 75 randomized/consented, much smaller completed samples.		Coulter limit certainty.	intensity and outcomes.	identical rehabilitation questions.		feasibility/acceptability evidence only.
Hybrid caregiver-mediated exercise with e-health/TR support vs usual care; early stroke mobility and ADL	1 pragmatic proof-of-concept RCT: van den Berg et al. 2016 [58]; n = 63 randomized.	Data basis: SIS mobility primary outcome, NEADL, length of stay, readmissions and caregiver outcomes. ITT primary mobility effect was neutral.	Serious. Allocation/blinding were strong, but unblinded self-reported outcomes and per-protocol emphasis create concerns.	Not assessable because one trial contributes.	Serious. Intervention combines caregiver-mediated exercises, app, activity monitor, home visits and videoconferencing; TR-specific effect is not isolated.	Serious. Proof-of-concept sample and several favorable results were secondary/per-protocol.	Not assessable (k < 10). Overall certainty: Low for mobility/ADL effects; promising but not definitive.
Delivery-content-only home telerehabilitation comparisons; stroke or MS motor outcomes	4 randomized/crossover studies: Deng et al. 2012 [59], Dastan et al. 2025 [57], Fluet et al. 2024 [65], Flynn et al. 2021 [66].	Data basis: complex vs simple ankle TR, synchronous vs asynchronous upper-limb TR, scaffolding vs algorithmic games, and home monitoring vs centre continuation. Not pooled as effectiveness evidence.	Some concerns to high. Small samples, attrition and feasibility/delivery-oriented designs were common.	Not pooled because clinical and methodological inconsistency is intrinsic to delivery/content comparisons.	Very serious for primary TR efficacy: both arms usually received remote/technology-supported rehabilitation or a common exercise base.	Serious to very serious due small samples and secondary clinical outcomes.	Not assessable (k < 10). Overall certainty: Not rated for primary effectiveness; very low for exploratory delivery/content conclusions.
Home VR/telerehabilitation for postural instability vs in-clinic sensory-integration balance training; Parkinson disease balance	1 multicenter single-blind RCT: Gandolfi et al. 2017 [68]; n = 76 randomized.	Data basis: BBS primary outcome, DGI, ABC, 10MWT, PDQ-8 and falls at post-treatment and 1-month follow-up.	Serious/some concerns. Randomization and blinded assessment were used, but participants/therapists were unblinded and reporting flexibility remains possible.	Not assessable because one trial contributes.	Serious: compares home VR telerehabilitation with in-clinic SIBT, mixing delivery setting and rehabilitation modality.	Serious. Modest sample and outcome-specific effects were not uniform.	Not assessable (k < 10). Overall certainty: Low for balance outcomes.
Non-randomized or feasibility evidence for stroke balance/upper-limb telerehabilitation	2 studies: Cikajlo et al. 2012 [53] and Garcia et al. 2022 [69]; very small samples.	Data basis: BBS/TUG/10m walk/standing balance and FIM/VAS/Berg/SF-36/ROM. Not pooled.	Serious to high/very serious. Non-equivalent controls or feasibility crossover with major incomplete follow-up.	Not formally pooled; inconsistency not estimable.	Serious. Relevant to implementation and feasibility, but indirect for causal efficacy.	Very serious. Small samples and non-poolable estimates.	Not assessable (k < 10). Overall certainty: Very low; retained only as narrative/contextual evidence.
Telerehabilitation versus in-person home physical therapy; Parkinson disease motor symptoms, balance/gait and QoL	1 RCT: Ge et al. 2024 [70]; n = 190 allocated (100 HPT, 90 TR).	Data basis: UPDRS-III primary outcome plus BBS, TUG, FTSST, FOGQ, gait parameters and PDQ-39 after 4 weeks; age-stratified effects reported.	Some concerns. Blinded assessment/analysis reported, but unblinded delivery and differential therapist supervision/adherence may bias comparative effects.	Not assessable with one study; subgroup findings suggest possible effect modification by age.	Serious. The comparison estimates TR relative to in-person HPT, not relative to no care/usual care or conventional outpatient rehabilitation.	Serious. Comparatively large sample but age-dependent between-group effects and adherence differences limit precision for a general conclusion.	Not assessable (k < 10). Overall certainty: Low for TR versus HPT; not directly applicable to primary TR effectiveness versus usual care.
Smartphone/wearable feedback gait training versus personalized gait advice; Parkinson disease gait and balance	1 pilot RCT: Ginis et al. 2016 [71]; n = 40 randomized.	Data basis: single- and dual-task gait speed, MiniBESTest, 2MWT, FES-I, FOG outcomes and SF-36 at post-test and 4-week retention.	High. Randomization was used, but testers were not blinded and performance outcomes were susceptible to measurement bias.	Not assessable with one pilot study.	Serious. The intervention tests automated feedback/cueing technology added to home gait training, not telerehabilitation as a service model alone.	Serious. Pilot sample and limited between-group effects; most outcomes improved similarly across groups.	Not assessable (k < 10). Overall certainty: Very low to Low; retained for wearable-feedback sensitivity/narrative synthesis.
VR-based telerehabilitation versus at-home conventional motor activities; Parkinson disease postural stability	1 multicenter RCT: Goffredo et al. 2023 [72]; n = 105 randomized, 97 analyzed.	Data basis: Mini-BESTest primary outcome; TUG, 6MWT and MDS-UPDRS III after 30 sessions.	Some concerns. Outcome assessors were blinded, but participants were unblinded and feedback/monitoring differed between arms.	Not assessable with one study; should be interpreted alongside, but not automatically pooled with, in-clinic comparator VR/TR trials.	Serious. Comparator was self-administered at-home conventional exercise, so the effect includes VR feedback and remote monitoring differences.	Serious. Between-group Mini-BESTest improvement was statistically significant but modest and not uniformly clinically meaningful.	Not assessable (k < 10). Overall certainty: Low to Moderate pending pooled consistency with other PD balance TR evidence.

Wearable remote-rehabilitation system plus PT versus routine OT plus PT; stroke motor recovery	1 randomized controlled pilot trial: Guo et al. 2023 [73]; n = 120.	Data basis: Fugl-Meyer total, upper-extremity and lower-extremity motor scores after 3 weeks in rehabilitation institutions.	High. Allocation details and assessor blinding were unclear in an obvious device-based intervention.	Not assessable with one study.	Serious. Inpatient rehabilitation-hall trial with routine PT/drug treatment is indirect for home/community telerehabilitation after discharge.	Serious. Pilot evidence; total and upper-extremity effects favored the system, but lower-extremity effects were less certain.	Not assessable (k < 10). Overall certainty: Very low to Low; sensitivity/device evidence only.
Home VR serious-game telerehabilitation versus GRASP home exercise; chronic stroke upper-limb recovery	1 evaluator-blinded RCT: Hernandez et al. 2022 [75]; n = 53 randomized, 51 completed.	Data basis: FMA-UE primary outcome, SIS and MAL-14 at post-intervention and 4-week follow-up; feasibility/adherence metrics.	Some concerns. Evaluators were blinded, but the VR arm had remote monitoring/progression while standard care was unsupervised after instruction.	Not assessable with one study; responder effects varied by active use and baseline function.	Serious. Comparator was an evidence-based home exercise program; the contrast tests serious-game engagement/monitoring rather than TR versus usual care.	Serious. No statistically significant between-group differences; clinically meaningful gains were concentrated among active VR users.	Not assessable (k < 10). Overall certainty: Low; useful for active-comparator and engagement-sensitive synthesis.
Internet-based exercise and physical-activity promotion versus waitlist/usual care; multiple sclerosis physical activity and health outcomes	1 multicenter waitlist-controlled RCT: Hartung et al. 2025 [74]; n = 56 analyzed.	Data basis: device-measured steps/day primary outcome, MVPA, self-reported sport/exercise, PAHCO, walking ability, fatigue and MSIS-29.	Some concerns. Randomization was stratified, but the study was unblinded, retrospectively registered and used imputation for missing data.	Not assessable with one study; intervention targets PA behavior rather than motor recovery.	Serious. Indirect for motor-rehabilitation effectiveness because the core outcome is PA promotion/behavior change.	Serious. Primary steps/day effect was not significant and the sample was modest with missing data.	Not assessable (k < 10). Overall certainty: Low for PA-promotion outcomes; not used as primary motor-recovery evidence.
Task-oriented telerehabilitation versus face-to-face task-oriented training or tele-neurofacilitation; stroke upper-limb function after 3 weeks	1 retrospective cohort non-inferiority study: Hong et al. 2025 [76]; n = 79 analyzed (Tele-Rehab n = 23; FTF n = 28; Tele-Control n = 28).	Data basis: FMA-UE primary outcome plus WMFT, ARAT and IADL. FMA-UE change was 9.4 (SD 8.80) in Tele-Rehab, 6.4 (7.52) in FTF and 6.7 (6.28) in Tele-Control. ANCOVA LS mean difference: Tele-Rehab vs FTF 3.29 (95% CI -0.81 to 7.39) and Tele-Rehab vs Tele-Control 2.99 (95% CI -1.11 to 7.08); non-inferiority margin 12.4.	Serious. Retrospective historical allocation, baseline disease-duration imbalance, residual confounding, differential missing data and no reported blinded outcome assessment limit causal certainty.	Not assessable with one observational study.	Serious for primary telerehabilitation effectiveness because this is an active-comparator/delivery non-inferiority cohort, not a randomized telerehabilitation versus usual-care/no-care comparison.	Serious. Modest total sample with a smaller Tele-Rehab group; confidence intervals support non-inferiority but do not provide high-precision comparative efficacy evidence, and no long-term follow-up was available.	Not assessable (k < 10). Overall certainty: Very low; retained as non-randomized narrative/sensitivity evidence only.
Mixed neurological arm/hand telerehabilitation versus usual care; ARAT/NHPT after home HCAD training	1 randomized multicentre trial: Huijgen et al. 2008 [77]; n = 81 randomized, 70 with follow-up.	Single-study equivalence/clinical-outcome basis; ARAT and NHPT after one month of HCAD versus usual care/general exercises.	Serious. Pragmatic randomization, unclear concealment/blinding and attrition.	Not assessable; one mixed-diagnosis study.	Serious. Stroke, MS and TBI were combined, limiting diagnosis-specific directness.	Serious. Modest sample and equivalence bounds/subgroup interpretation.	Not assessable (k < 10). Overall certainty: Low to Very low.
Stroke upper-limb or global motor recovery using virtual/wearable telerehabilitation versus usual care, OT or structured home program	3 randomized trials/pilot trials: Guo et al. 2023 [73], Johnson et al. 2020 [79], Hernandez et al. 2022 [75]; plus related delivery-only SCI evidence Kowalczewski et al. 2011 [82].	Data basis: Fugl-Meyer/FMA-UE, ARAT, SIS/MAL and related motor-function outcomes; effects are study-specific and not assumed interchangeable.	Serious to high. Assessor blinding was strong in Johnson/Hernandez, but Guo had unclear assessor blinding and Kowalczewski was delivery-content only.	Serious. Intervention technologies, setting and comparator intensity differ.	Serious. Evidence spans community, home, inpatient/device trials and different comparators.	Serious. Small-to-moderate samples and several non-primary/sensitivity estimates.	Not assessable (k < 10). Overall certainty: Low to Very low depending on outcome.
Stroke mobility, balance and ADL telerehabilitation/hybrid home programs versus usual care, bedside/conventional therapy or in-clinic VR	7 RCT/pilot studies: Jarbandhan et al. 2022 [78], Kintrilis et al. 2024 [81], Lee et al. 2022 [83], Lee et al.	Data basis: 6MWT, BBS, TIS, PASS, FAC, MBI, SIS mobility and related function/QoL endpoints; pooling should be stratified by comparator and domain.	Serious to high. Several studies were pilots with unblinded delivery; Jarbandhan had no assessor blinding and high attrition.	Serious. Delivery format, dose, setting, comparator and outcome domains are heterogeneous.	Serious. Includes inpatient, LTCF, home/community, caregiver-mediated and resistance-training	Serious to very serious. Many small pilots; Mulder stopped early for futility.	Not assessable (k < 10). Overall certainty: Very low to Low; exploratory by domain.

	2025 [84], Lin et al. 2014 [85], Llorens et al. 2015 [86], Mulder et al. 2024 [88].				models.		
Parkinson disease home-based telerehabilitation, feedback or exergaming versus active delivery comparator, HPT, usual care or gaming control	5 randomized/pilot trials: Ge et al. 2024 [70], Ginis et al. 2016 [71], Goffredo et al. 2023 [72], Johnson et al. 2024 [80], Nuic et al. 2024 [89].	Data basis: UPDRS-III, Mini-BESTest, TUG, SWST, gait speed, 6MWT, balance confidence, QoL and adherence/safety outcomes.	Serious. Blinding and assessor independence varied; Johnson 2024 was high risk because outcomes were collected by treating therapists.	Serious. Effects differ across HPT comparison, wearable feedback, VR tablet, pilot feasibility and exergaming control designs.	Serious. Several comparisons are delivery-model or active-comparator questions, not pure TR versus no rehabilitation.	Serious. Samples range from very small pilot to larger single trials; outcome-specific uncertainty persists.	Not assessable (k < 10). Overall certainty: Low for more robust PD balance/gait trials; Very low for pilot feasibility outcomes.
Multiple sclerosis or hereditary ataxia remote exercise/balance/PA programs versus conventional therapy, waitlist/usual care or alternate TR format	3 studies: Hartung et al. 2025 [74], Masbernat-Almenara et al. 2026 [87], Ortiz-Gutiérrez et al. 2013 [90].	Data basis: device-measured steps/MVPA, S-TIS/SARA/4MWT/TUG/ABC, and CDP/SOT balance outcomes; not a single pooled construct.	Serious. Hartung and Masbernat were randomized but small/unblinded; Ortiz was non-randomized with serious confounding/selection risk.	Serious. Interventions target PA promotion, ataxia core stability, and MS postural control respectively.	Serious to very serious. Diagnoses and mechanisms differ substantially, and Ortiz is non-randomized.	Serious to very serious. Small samples and multiple exploratory endpoints.	Not assessable (k < 10). Overall certainty: Very low to Low; use mainly as domain-specific supportive evidence.
Teleconference resistance training versus in-person resistance training or usual care after acute stroke	1 randomized controlled study: Kintrilis et al. 2024 [81]; n = 90.	Data basis: VAS health status, TUG, BBS, CST, 6MWT and A-VO₂ at 3 months after discharge.	Serious/some concerns. Random assignment and blinded testing were reported, but concealment and missing-data handling were incomplete.	Not assessable with one study.	Serious. Modality-specific anaerobic/resistance training and acute-stroke post-discharge context limit broader TR generalization.	Serious. Several outcomes, limited prespecification and no clear pooled estimate.	Not assessable (k < 10). Overall certainty: Low to Very low.
Telerehabilitation vs usual care/home exercise; multiple sclerosis motor, balance, physical activity and QoL outcomes.	3 RCTs: Pagliari et al. 2024 [91], Paul et al. 2014 [92], Paul et al. 2019 [93]; supportive nonrandomized evidence should be kept separate.	No pooled estimate in this table; outcomes include MSQOL-54, Mini-BESTest, 2MWT/25-Foot Walk, BBS, TUG and MSIS.	Serious: most studies have some concerns from unblinded delivery, pilot/feasibility design or attrition.	Serious: direction and magnitude vary by outcome and intervention intensity.	Some concern: comparators include usual care, booklet exercise and conventional home rehabilitation; constructs differ.	Serious: mostly small or feasibility studies; publication bias not assessable (k < 10). Overall certainty: Low.	Overall certainty: Low
Telerehabilitation vs onsite rehabilitation; multiple sclerosis QoL, fatigue, balance and cognition.	1 nonrandomized comparative study: Petracca et al. 2024 [95]; n = 51 completers.	No pooled estimate; comparison based on longitudinal change and MCID analyses.	Serious: temporal COVID-era allocation and differential discontinuation.	Not assessable; single study.	Some concern: directly relevant comparison but affected by pandemic-era context.	Serious: single nonrandomized study; publication bias not assessable. Overall certainty: Very low.	Overall certainty: Low
Stroke upper-limb telerehabilitation or virtual/remote upper-limb programs vs conventional/usual care; post-intervention motor/activity outcomes.	5 RCTs/feasibility RCTs: Piron et al. 2009 [97], Saygili et al. 2024 [102], Simpson et al. 2025 [105], Standen et al. 2017 [108], Sun et al. 2025 [109].	Effect basis includes FMA-UE/STREAM/WMFT/MAL/TENZR reach-to-grasp counts/BI; formal pooling should separate scale/domain and comparator type.	Serious: mostly some concerns; Standen et al. has high risk due to attrition.	Serious: technologies, stroke stage, intervention dose and outcome constructs are heterogeneous.	Some concern: includes telerehab, virtual programs, Tele-CIMT and multicomponent post-discharge care.	Serious: several small trials and one larger trial; publication bias not assessable (k < 10 within homogeneous cells). Overall certainty: Low.	Overall certainty: Low
Integrated synchronous multi-domain stroke telerehabilitation vs conventional outpatient therapy; upper-limb motor non-inferiority and secondary domains.	1 RCT: Picelli et al. 2026 [96]; n = 56.	Non-inferiority contrast for FMA-UE; secondary language/cognitive/ADL outcomes reported without clear superiority.	Some concerns: unblinded delivery but strong randomization, complete follow-up and blinded assessment.	Not assessable; single study.	Not serious for the specified multi-domain synchronous TR question.	Serious: one study and wide CI. Publication bias not assessable. Overall certainty: Low to Moderate pending final GRADE threshold.	Overall certainty: Low
Robotic vs non-robotic telerehabilitation after stroke; upper-limb disability and motor/cognitive	1 delivery-model RCT: Pavan et al. 2024 [94]; n = 30.	No pooled estimate; both arms received telerehabilitation, with robotic vs non-robotic delivery.	Some concerns.	Not assessable; single study.	Very serious for primary telerehabilitation	Serious: small single study; publication bias not assessable. Overall	Overall certainty: Low

outcomes.					effectiveness because no non-telerehabilitation comparator was used.	certainty for delivery-model question: Low; for TR efficacy: not directly applicable.	
Stroke post-discharge/trunk/balance/gait telerehabilitation or augmented community TR; balance, mobility and participation outcomes.	4 studies: Salgueiro et al. 2022a [100], Salgueiro et al. 2022b [101], Saywell et al. 2021 [103], Sheehy et al. 2025 [104].	No pooled estimate; constructs include S-TIS/S-FIST/BBS/gait, SIS physical/mobility, TUG/FTSTS/CB&M and community integration.	Serious: one nonrandomized extension, feasibility designs and self-reported outcomes.	Serious: mixed stages, intervention formats and domains.	Serious: app-guided CSE, ACTIV phone/text program and NIVRT are related but clinically non-identical.	Serious to very serious: small samples/feasibility trials and attrition; publication bias not assessable. Overall certainty: Very low to Low.	Overall certainty: Low
Parkinson disease telerehabilitation/exergaming/remote physiotherapy vs booklet/usual care/hospital/control; gait, balance, mobility and activity outcomes.	4 studies: Pastana Ramos et al. 2023 [99], Prukviwat et al. 2026 [98], Skelly et al. 2026 [106], Song et al. 2018 [107].	No pooled estimate; outcomes include TUG, 5STS, ABC, UPDRS, BBS, Chula PMS, step count and Functional Gait Assessment.	Serious: one nonrandomized patient-preference study and several pilot/feasibility or unblinded RCTs.	Serious: effects differ across intervention technology, supervision and disease severity.	Serious: PD stage and intervention content differ; some studies evaluate exergaming rather than therapist-led TR.	Serious to very serious: small samples and exploratory analyses; publication bias not assessable. Overall certainty: Very low to Low.	Overall certainty: Low
Telerehabilitation vs usual care during COVID-19; spinal cord injury independence and anxiety outcomes.	1 pilot RCT: Swarnakar et al. 2023 [110]; n = 30.	No pooled estimate; SCIM III primary outcome and Coronavirus Anxiety Scale secondary outcome.	Serious: questionable participant blinding and remote/self-report elements despite concealed randomization.	Not assessable; single study.	Some concern: pandemic-specific SCI context may limit applicability outside crisis settings.	Serious: small pilot study; publication bias not assessable. Overall certainty: Low to Very low.	Overall certainty: Low
Home active-gaming telerehabilitation/physical-activity support versus wait-list/usual care; MS activity, balance and mobility	1 pilot RCT: Thomas et al. 2017 [111]; n = 30.	No pooled estimate; data basis includes feasibility, activity logs, GLTEQ/activPAL, balance/gait/mobility and hand dexterity outcomes.	Some concerns: unblinded delivery and partially unblinded physical assessment.	Not assessable with one pilot study.	Some concern: active gaming and activity promotion are indirect for impairment-level motor rehabilitation.	Serious: feasibility sample and exploratory outcomes.	Not assessable (k < 10). Overall certainty: Very low to Low.
Wearable/app-based upper-limb telerehabilitation versus sham/conventional home training after stroke; upper-limb impairment/activity	2 randomized studies: Toh et al. 2025a [112] and Toh et al. 2025b [113]; total n = 52 randomized/allocated across parallel and crossover designs.	No pooled estimate in this table; outcomes include FMA-UE, AROM, strength, ARAT, MAL and adherence/compliance.	Some concerns overall: blinded assessors but unblinded device feedback/monitoring and small pilot crossover evidence.	Serious: parallel RCT and crossover pilot differ in design, dose and feedback structure.	Some concern: both studies test Smart Reminder-specific wearable feedback rather than generic telerehabilitation.	Serious: small samples and exploratory outcome sets.	Not assessable (k < 10). Overall certainty: Low.
Tele-health CIMT versus in-lab CIMT after chronic stroke; upper-limb use and capacity	1 proof-of-concept RCT: Uswatte et al. 2021 [114]; n = 24 randomized, 20 completers.	Non-inferiority/delivery-comparison basis; MAL Arm Use and WMFT outcomes.	Some concerns: unblinded delivery and limited assessor-blinding detail.	Not assessable with one study.	Serious for primary efficacy because both groups received CIMT; evidence addresses delivery equivalence.	Serious: small sample and reduced one-year follow-up precision.	Not assessable (k < 10). Overall certainty for delivery equivalence: Low.
Home trunk telerehabilitation versus active home limb exercise; Parkinson disease balance/gait	1 RCT: Vasconcellos et al. 2023 [115]; n = 28.	No pooled estimate; outcomes include force-plate COP variables and gait kinematics.	High: substantial non-adherence/losses despite objective blinded measures.	Not assessable with one study.	Very serious for TR efficacy because both groups received remote home exercise; the contrast is content-specific.	Serious: small trial and null effects.	Not assessable (k < 10). Overall certainty: Very low.
Caregiver-mediated e-health supported exercise plus usual care versus usual care after stroke; mobility/ADL/discharge outcomes	1 multicenter RCT: Vloothuis et al. 2019 [116]; n = 66 patient-caregiver dyads.	No pooled estimate; primary outcomes SIS mobility and length of stay; secondary lower-limb, balance, ADL and psychosocial outcomes.	Some concerns: unblinded caregiver-mediated intervention, but observer-blinded assessment and ITT analysis.	Not assessable with one study.	Some concern: complex caregiver/e-health intervention and early-discharge context may not isolate telerehabilitation effect.	Serious: single study and no significant primary outcome effect.	Not assessable (k < 10). Overall certainty: Low.
Internet/wearable home rehabilitation versus routine follow-	2 RCT/quasi-randomized studies:	No pooled estimate; outcomes include FMA, BBS, MBI, gait parameters,	Serious to high: Wu has some concerns; Wang has high risk	Serious: different populations, delivery	Some concern: both are Chinese post-	Serious: single-country evidence, multiple	Not assessable (k < 10). Overall certainty: Very

up or home prescription after stroke; limb function, gait and ADL	Wang et al. 2024 [117] and Wu et al. 2020 [120]; n = 80 and n = 64 randomized/recruited.	TUG, 6MWT and quality of life.	from odd/even allocation and incomplete concealment.	models and outcome batteries.	discharge collaborative/Internet rehabilitation models, limiting wider generalizability.	outcomes and incomplete variance readiness for some endpoints.	low to Low.
Home virtual/robotic upper-limb rehabilitation versus active home exercise after stroke; upper-limb function	2 RCTs: Wilson et al. 2021 [118] and Wolf et al. 2015 [119]; n = 19 and n = 99 randomized.	No pooled estimate in this table; outcomes include BBT, 9HPT, ARAT, WMFT, FMA-UE and functional self-report.	Some concerns: blinded outcome assessment but unblinded device/robotic delivery and support.	Serious: EDNA virtual training and Hand Mentor robotic HEP differ substantially.	Serious: both use active home exercise comparators and address technology-enhanced delivery rather than simple TR vs usual care.	Serious: Wilson is small; Wolf has more robust sample but some effect-level variance may require reconstruction.	Not assessable (k < 10). Overall certainty: Low.
Home-based telerehabilitation versus matched/no home-based TR after functionally independent stroke/TIA; exercise capacity and HRQOL	1 observational PSM study: Yokota et al. 2025 [121]; analyzed n = 21 TR and n = 63 matched controls.	No pooled estimate; 6MWD change, knee-extension strength, handgrip strength and SF-36 component scores.	Serious: ROBINS-I serious due to self-selection, exclusions before PSM and residual confounding.	Not assessable with one observational study.	Some concern: independent stroke/TIA survivors and cardiac-style ergometer TR may not generalize to disabled neurorehabilitation cohorts.	Serious: small TR group and observational design.	Not assessable (k < 10). Overall certainty: Very low.
Remote aerobic/resistance exercise versus stretching control; older adults with MS physical and cognitive function	1 phase-Ib RCT: Zheng et al. 2026 [122]; n = 51 randomized.	No pooled estimate; outcomes include T25FW, TUG, 30STS, 6MWT, BICAMS and physical activity.	High for efficacy because of feasibility design and post/follow-up attrition despite concealed allocation and blinded assessment.	Not assessable with one study.	Some concern: exercise-training intervention in older MS is relevant but partly indirect for clinical telerehabilitation motor recovery.	Serious to very serious: exploratory phase-Ib sample and incomplete follow-up.	Not assessable (k < 10). Overall certainty: Very low to Low.

GRADE interpretation note: Randomized evidence starts as high certainty and may be downgraded for risk of bias, inconsistency, indirectness, imprecision and publication bias. Non-randomized evidence starts as low certainty and was not combined with randomized evidence for primary certainty judgments. Publication bias was judged not assessable when fewer than 10 studies contributed to a comparison. Positive effect estimates favor telerehabilitation unless otherwise stated.

Table S14. Study-level synthesis role and risk-of-bias linkage.

Study	Design	Comparator/main contrast	Main quantitative contribution	Synthesis role	RoB tool and overall judgment	Main reason/interpretive note
García-Rudolph et al. 2024 [36]	Matched case-control	Matched in-person rehabilitation	Global motor-functional/mobility outcomes	Sensitivity/narrative evidence	ROBINS-I: Serious	Non-randomized matched case-control design; narrative/sensitivity only
Thielbar et al. 2020 [37]	Randomized crossover trial	Single-user VERGE VR	Upper-limb engagement and motor outcomes	Delivery-model/narrative evidence	RoB 2: Some concerns	Delivery-model comparison; both arms received home VR telerehabilitation
Adams et al. 2023 [38]	Rater-blinded RCT	Usual and customary care	Upper limb motor impairment	Exploratory k=2 pooled analysis	RoB 2: Some concerns	Included in quantitative Table 4; interpretation follows primary/exploratory label.
Allegue et al. 2022 [39]	Two-arm feasibility clinical trial	Conventional home GRASP	Upper limb motor impairment	Sensitivity/narrative evidence	RoB 2: High	Feasibility/pilot study not used for confirmatory primary pooling
Asano et al. 2021 [40]	Evaluator-blinded RCT	Usual rehabilitation care	Gait/mobility	Single-study compatible/sensitivity evidence	RoB 2: Some concerns	Single compatible study only or not clinically/statistically compatible with selected pooled comparison
Aytutuldu et al. 2024 [41]	Single-blind RCT	Progressive structured mobility training via synchronous telerehabilitation	Balance	Sensitivity/narrative evidence	RoB 2: High	Delivery-model/content comparison or both arms received telerehabilitation
Ballester et al. 2017 [42]	Parallel controlled randomized trial	Domiciliary occupational therapy task	Upper limb activity	Exploratory k=2 pooled analysis	RoB 2: High	Included in quantitative Table 4; interpretation follows primary/exploratory label.
Benvenuti et al. 2014 [43]	Longitudinal cohort with geographic control group	Usual care	Upper limb motor function	Sensitivity/narrative evidence	ROBINS-I: Serious	Non-randomized/observational or unclear allocation; narrative/sensitivity only
Calabrò et al. 2023 [44]	Multicenter RCT	Usual territorial rehabilitative treatment	ADL	Sensitivity/narrative evidence	RoB 2: Some concerns	Missing or non-reconstructable effect variance
Carey et al. 2007 [45]	Randomized trial with crossover component	Simple movement telerehabilitation	Upper limb dexterity	Sensitivity/narrative evidence	RoB 2: Some concerns	Delivery-model/content comparison or both arms received telerehabilitation
Chae et al. 2020 [46]	Prospective comparative study	Conventional home exercise handout with weekly calls	Upper limb motor function	Sensitivity/narrative evidence	ROBINS-I: Serious	Non-randomized/observational or unclear allocation; narrative/sensitivity only
Chantanachai et al. 2025 [47]	Double-blind randomized sham-	Sham tDCS + same tele-supervised	SCI upper limb motor	Sensitivity/narrative evidence	RoB 2: Low	Not clinically/statistically compatible with primary

Study	Design	Comparator/main contrast	Main quantitative contribution	Synthesis role	RoB tool and overall judgment	Main reason/interpretive note
	controlled trial	exercise				pooled comparisons
Chen et al. 2017 [48]	Assessor-blinded RCT	Conventional outpatient rehabilitation	Balance; ADL	Single-study compatible/sensitivity evidence	RoB 2: Some concerns	Single compatible study only or not clinically/statistically compatible with selected pooled comparison
Chen et al. 2020 [49]	Randomized controlled trial	Conventional rehabilitation	Motor impairment	Exploratory k=2 pooled analysis	RoB 2: Some concerns	Included in quantitative Table 4; interpretation follows primary/exploratory label.
Chen et al. 2021 [50]	Prospective case-control pilot randomized study	Conventional one-on-one physiotherapy	Balance	Sensitivity/narrative evidence	RoB 2: Some concerns	Median/IQR or non-reconstructable variance; not primary poolable
Chumbler et al. 2012 [51]	Multisite single-blinded RCT	Usual care	ADL/motor function	Primary pooled analysis	RoB 2: Some concerns	Included in quantitative Table 4; interpretation follows primary/exploratory label.
Chung et al. 2020 [52]	Single-blind randomized controlled pilot trial	Paper-based home exercise program	Gait/mobility; ADL/motor-functional	Sensitivity/narrative evidence	RoB 2: Some concerns	Not clinically/statistically compatible with primary pooled comparisons
Cikajlo et al. 2012 [53]	Pilot controlled comparative study	Balance training without VR/telerehabilitation	Balance; Gait/mobility	Sensitivity/narrative evidence	ROBINS-I: Serious	Not clinically/statistically compatible with primary pooled comparisons
Conroy et al. 2018 [54]	Randomized single-blind controlled trial	Routine home rehabilitation	Gait/mobility; Balance	Sensitivity/narrative evidence	RoB 2: High	Not clinically/statistically compatible with primary pooled comparisons
Coulter et al. 2017 [55]	Pilot randomized controlled trial	Usual care/self-management	Gait/mobility	Sensitivity/narrative evidence	RoB 2: High	Missing or non-reconstructable effect variance
Cramer et al. 2019 [56]	Assessor-blinded noninferiority randomized clinical trial	Dose-matched in-clinic therapy	Upper limb motor impairment	Sensitivity/narrative evidence	RoB 2: Low	Delivery-model/content comparison or both arms received telerehabilitation
Dastan et al. 2025 [57]	Single-blind randomized study	Asynchronous telerehabilitation	Upper limb dexterity	Sensitivity/narrative evidence	RoB 2: Some concerns	Delivery-model/content comparison or both arms received telerehabilitation
van den Berg et al. 2016 [58]	Pragmatic randomized proof-of-concept trial	Usual rehabilitation care	Self-reported mobility; ADL; Gait/mobility	Sensitivity/narrative evidence	RoB 2: Some concerns	Missing or non-reconstructable effect variance
Deng et al. 2012 [59]	Pilot randomized controlled trial	Simple ankle movement telerehabilitation	Ankle/gait kinematics	Sensitivity/narrative evidence	RoB 2: Some concerns	Delivery-model/content comparison or both arms received telerehabilitation
van der Kolk et al. 2019 [60]	Double-blind randomized controlled trial	Home-based stretching active control	Parkinson motor symptoms; Gait/mobility; Balance	Single-study compatible/sensitivity evidence	RoB 2: Low	Single compatible study only or not clinically/statistically compatible with selected pooled comparison
Dogan et al. 2023 [61]	Assessor-blinded randomized controlled trial	Clinic-based virtual reality supported task-oriented circuit therapy	Trunk control; Ataxia kinetic function	Sensitivity/narrative evidence	RoB 2: Some concerns	Delivery-model/content comparison or both arms received telerehabilitation
Eldemir et al. 2023 [62]	Randomized controlled trial	Home exercise	Upper limb dexterity; Parkinson motor symptoms	Single-study compatible/sensitivity evidence	RoB 2: Some concerns	Single compatible study only or not clinically/statistically compatible with selected pooled comparison
Eldemir et al. 2024 [63]	Assessor-blinded randomized controlled trial	Waitlist/no Pilates-TR	Balance; Gait/mobility	Primary pooled analysis	RoB 2: Some concerns	Included in quantitative Table 4; interpretation follows primary/exploratory label.
Fjeldstad-Pardo et al. 2018 [64]	Randomized three-arm evaluator-blinded feasibility pilot study	Unsupervised home exercise and in-person PT arms	Balance; Gait/mobility	Sensitivity/narrative evidence	RoB 2: Some concerns	Feasibility/pilot study not used for confirmatory primary pooling
Fluet et al. 2024 [65]	Randomized controlled trial	Algorithm-controlled home virtual rehabilitation games	Upper limb motor impairment	Sensitivity/narrative evidence	RoB 2: Some concerns	Delivery-model/content comparison or both arms received telerehabilitation
Flynn et al. 2021 [66]	Randomized pilot trial after common initial exercise block	Centre-based exercise	Balance; Gait/mobility	Sensitivity/narrative evidence	RoB 2: Some concerns	Delivery-model/content comparison or both arms received telerehabilitation
Frevel et al. 2015 [67]	Randomized controlled study	Hippotherapy	Balance	Sensitivity/narrative evidence	RoB 2: High	Not clinically/statistically compatible with primary pooled comparisons
Gandolfi et al. 2017 [68]	Multicenter single-blind RCT	In-clinic sensory integration balance training	Balance; Gait/mobility; Balance confidence; multiple additional domains	Sensitivity/narrative evidence	RoB 2: Some concerns	Delivery-model/content comparison or both arms received telerehabilitation
Garcia et al. 2022 [69]	Feasibility crossover trial	Conventional self-directed home therapy	Upper-limb ROM/global function and balance	Sensitivity/narrative evidence	RoB 2: High	Small feasibility crossover study with incomplete follow-up; not primary poolable
Ge et al. 2024 [70]	Randomized controlled trial	Home physical therapy	Disease-specific motor symptoms; Balance; Functional mobility/fall risk; multiple additional domains	Sensitivity/narrative evidence	RoB 2: Some concerns	Delivery-model/content comparison or both arms received telerehabilitation
Ginis et al. 2016 [71]	Pilot RCT	Personalized gait advice active control	Gait/mobility; Balance; Balance/mobility; multiple additional domains	Sensitivity/narrative evidence	RoB 2: High	Not clinically/statistically compatible with primary pooled comparisons
Goffredo et al. 2023 [72]	Multicenter randomized controlled trial	At-home self-administered conventional motor activities	Balance; Functional mobility; Gait/endurance; multiple additional domains	Single-study compatible/sensitivity evidence	RoB 2: Some concerns	Single compatible study only or not clinically/statistically compatible with selected pooled comparison
Guo et al. 2023 [73]	Randomized controlled pilot trial	Routine OT + PT	Global motor impairment; Upper limb motor impairment; Lower limb motor impairment	Sensitivity/narrative evidence	RoB 2: High	Not clinically/statistically compatible with primary pooled comparisons
Hartung et al. 2025 [74]	Multicenter randomized waitlist-controlled study	Waitlist/usual care	Gait/endurance; Gait/mobility; Self-reported walking; multiple additional domains	Sensitivity/narrative evidence	RoB 2: Some concerns	Not clinically/statistically compatible with primary pooled comparisons
Hernandez et al. 2022 [75]	Evaluator-blinded RCT	GRASP standard home exercise program	Upper limb motor impairment	Sensitivity/narrative evidence	RoB 2: Some concerns	Median/IQR or non-reconstructable variance; not primary poolable
Hong et al. 2025 [76]	Retrospective cohort non-inferiority	Face-to-face task-oriented training; tele-control neurofacilitation	Upper limb motor impairment	Sensitivity/narrative evidence	ROBINS-I: Serious	Non-randomized/observational or unclear allocation; narrative/sensitivity only
Huijgen et al. 2008 [77]	Randomized multicentre clinical trial	Usual care and generic exercises	Upper limb activity	Sensitivity/narrative evidence	RoB 2: Some concerns	Feasibility/pilot study not used for confirmatory primary pooling
Jarbandhan et al. 2022 [78]	Randomized controlled pilot study	Usual care	Gait/endurance	Sensitivity/narrative evidence	RoB 2: High	Not clinically/statistically compatible with primary pooled comparisons
Johnson et al. 2020 [79]	Assessor-blinded RCT	Usual care	Upper limb motor impairment; Upper limb activity; Upper limb manual dexterity; multiple additional domains	Single-study compatible/sensitivity evidence	RoB 2: Some concerns	Single compatible study only or not clinically/statistically compatible with selected pooled comparison
Johnson et al. 2024 [80]	Pilot randomized controlled trial	Usual care in-clinic therapy	Functional mobility; Gait/mobility; Gait/endurance; multiple additional domains	Sensitivity/narrative evidence	RoB 2: High	Feasibility/pilot study not used for confirmatory primary pooling
Kintrilis et al. 2024 [81]	Randomized controlled study	In-person strength training; usual care	Quality of life/perceived health; Functional mobility; Balance; multiple	Sensitivity/narrative evidence	RoB 2: Some concerns	Delivery-model/content comparison or both arms received telerehabilitation

Study	Design	Comparator/main contrast	Main quantitative contribution	Synthesis role	RoB tool and overall judgment	Main reason/interpretive note
			additional domains			
Kowalczewski et al. 2011 [82]	Blinded randomized crossover trial	Conventional ET via in-home teletherapy	Upper limb activity; Automated hand function	Sensitivity/narrative evidence	RoB 2: Some concerns	Delivery-model/content comparison or both arms received telerehabilitation
Lee et al. 2022 [83]	Pilot RCT/non-inferiority	Conventional PT	Trunk control/balance; ADL/motor-functional; Balance; multiple additional domains	Sensitivity/narrative evidence	RoB 2: Some concerns	Feasibility/pilot study not used for confirmatory primary pooling
Lee et al. 2025 [84]	Prospective parallel single-blind pilot RCT	Bedside occupational therapy plus regular rehabilitation	ADL/motor-functional; Postural control/balance; Gait/ambulation	Sensitivity/narrative evidence	RoB 2: Some concerns	Feasibility/pilot study not used for confirmatory primary pooling
Lin et al. 2014 [85]	Multisite blocked-randomized pilot study	Conventional balance training	Balance	Sensitivity/narrative evidence	RoB 2: Some concerns	Feasibility/pilot study not used for confirmatory primary pooling
Llorens et al. 2015 [86]	Single-blind RCT	In-clinic VR balance program	Balance; Gait/mobility	Sensitivity/narrative evidence	RoB 2: Some concerns	Delivery-model/content comparison or both arms received telerehabilitation
Masbernat-Almenara et al. 2026 [87]	Single-blind two-arm pilot RCT	Asynchronous core stability telerehabilitation	Gait/mobility	Sensitivity/narrative evidence	RoB 2: Some concerns	Delivery-model/content comparison or both arms received telerehabilitation
Mulder et al. 2024 [88]	Multicentre observer-blinded RCT	Usual care alone	Self-reported mobility; Gait/endurance	Sensitivity/narrative evidence	RoB 2: Some concerns	Not clinically/statistically compatible with primary pooled comparisons
Nuic et al. 2024 [89]	Multicenter single-blind phase II RCT	Keyboard-control version of same game	Functional mobility; PD axial motor symptoms	Sensitivity/narrative evidence	RoB 2: Some concerns	Not clinically/statistically compatible with primary pooled comparisons
Ortiz-Gutiérrez et al. 2013 [90]	Controlled preliminary study	Conventional physiotherapy	Postural control/SOT	Sensitivity/narrative evidence	ROBINS-I: Serious	Non-randomized/observational or unclear allocation; narrative/sensitivity only
Pagliari et al. 2024 [91]	Multicentre rater-blinded RCT	Home-based conventional usual care	Balance	Exploratory k=2 pooled analysis	RoB 2: Some concerns	Included in quantitative Table 4; interpretation follows primary/exploratory label.
Paul et al. 2014 [92]	Randomized controlled pilot study	Usual care	Gait/mobility; Balance	Sensitivity/narrative evidence	RoB 2: Some concerns	Feasibility/pilot study not used for confirmatory primary pooling
Paul et al. 2019 [93]	Multicentre single-blind feasibility RCT	Printed sheet home exercise program	Gait/endurance	Sensitivity/narrative evidence	RoB 2: Some concerns	Not clinically/statistically compatible with primary pooled comparisons
Pavan et al. 2024 [94]	Single-blind randomized two-arm clinical trial	Robotic telerehabilitation	Upper limb motor impairment	Sensitivity/narrative evidence	RoB 2: Some concerns	Delivery-model/content comparison or both arms received telerehabilitation
Petracca et al. 2024 [95]	Single-center interventional comparative study	Onsite rehabilitation	Balance	Sensitivity/narrative evidence	ROBINS-I: Serious	Non-randomized/observational or unclear allocation; narrative/sensitivity only
Picelli et al. 2026 [96]	Bicentric assessor-blinded non-inferiority RCT	Conventional outpatient therapy	Upper limb motor impairment; Lower limb motor impairment	Single-study compatible/sensitivity evidence	RoB 2: Some concerns	Single compatible study only or not clinically/statistically compatible with selected pooled comparison
Piron et al. 2009 [97]	Randomized single-blind controlled trial	Traditional physical therapy	Upper limb motor impairment	Single-study compatible/sensitivity evidence	RoB 2: Some concerns	Single compatible study only or not clinically/statistically compatible with selected pooled comparison
Prukviwat et al. 2026 [98]	Patient-preference nonrandomized controlled pilot study	Hospital-based rehabilitation	Balance	Sensitivity/narrative evidence	ROBINS-I: Serious	Non-randomized/observational or unclear allocation; narrative/sensitivity only
Pastana Ramos et al. 2023 [99]	Parallel-group single-blind phase 2 randomized clinical trial	Booklet-based exercise program	Gait/mobility	Sensitivity/narrative evidence	RoB 2: Some concerns	Feasibility/pilot study not used for confirmatory primary pooling
Salgueiro et al. 2022a [100]	Single-blind preliminary RCT	Conventional physiotherapy	Trunk/sitting balance	Sensitivity/narrative evidence	RoB 2: Some concerns	Delivery-model/content comparison or both arms received telerehabilitation
Salgueiro et al. 2022b [101]	Prospective controlled extension trial	Usual care	Standing balance	Sensitivity/narrative evidence	ROBINS-I: Serious	Non-randomized/observational or unclear allocation; narrative/sensitivity only
Saygili et al. 2024 [102]	Evaluator-blind randomized controlled trial	Home exercise program	Upper limb motor impairment	Sensitivity/narrative evidence	RoB 2: Some concerns	Median/IQR or non-reconstructable variance; not primary poolable
Saywell et al. 2021 [103]	Assessor-blinded parallel RCT	Usual care	Physical function	Single-study compatible/sensitivity evidence	RoB 2: Some concerns	Single compatible study only or not clinically/statistically compatible with selected pooled comparison
Sheehy et al. 2025 [104]	Assessor-blinded parallel feasibility RCT	iPad cognitive/fine-motor active control	Balance	Sensitivity/narrative evidence	RoB 2: Some concerns	Feasibility/pilot study not used for confirmatory primary pooling
Simpson et al. 2025 [105]	Multicenter assessor-blinded waitlist RCT	Waitlist/usual care	Upper limb activity	Single-study compatible/sensitivity evidence	RoB 2: Some concerns	Single compatible study only or not clinically/statistically compatible with selected pooled comparison
Skelly et al. 2026 [106]	Randomized controlled feasibility trial	Usual care/exercise advice	Parkinson motor/global symptoms	Sensitivity/narrative evidence	RoB 2: Some concerns	Feasibility/pilot study not used for confirmatory primary pooling
Song et al. 2018 [107]	Single-blinded RCT	Usual care/no study intervention	Gait/balance	Sensitivity/narrative evidence	RoB 2: Some concerns	Not clinically/statistically compatible with primary pooled comparisons
Standen et al. 2017 [108]	Two-group feasibility RCT	Usual care	Upper limb use	Sensitivity/narrative evidence	RoB 2: High	Feasibility/pilot study not used for confirmatory primary pooling
Sun et al. 2025 [109]	Randomized controlled trial	Standard post-discharge care	ADL/motor-functional	Primary pooled analysis	RoB 2: Some concerns	Included in quantitative Table 4; interpretation follows primary/exploratory label.
Swarnakar et al. 2023 [110]	Single-center double-blind RCT	Standard usual care	SCI mobility	Primary pooled analysis	RoB 2: Some concerns	Included in quantitative Table 4; interpretation follows primary/exploratory label.
Thomas et al. 2017 [111]	Single-centre wait-list pilot RCT	Delayed/wait-list usual care	Mobility	Sensitivity/narrative evidence	RoB 2: Some concerns	Feasibility/pilot study not used for confirmatory primary pooling
Toh et al. 2025a [112]	Single-blind randomized controlled trial	Sham device plus pictorial handouts	Upper limb motor impairment	Exploratory k=2 pooled analysis	RoB 2: Some concerns	Included in quantitative Table 4; interpretation follows primary/exploratory label.
Toh et al. 2025b [113]	Single-blind randomized crossover feasibility pilot	Conventional home exercise with pictorial handouts	Upper limb ROM	Sensitivity/narrative evidence	RoB 2: Some concerns	Delivery-model/content comparison or both arms received telerehabilitation
Uswatte et al. 2021 [114]	Proof-of-concept RCT; non-inferiority framework	In-lab face-to-face CIMT	Upper limb use	Sensitivity/narrative evidence	RoB 2: Some concerns	Delivery-model/content comparison or both arms received telerehabilitation
Vasconcellos et al. 2023 [115]	Single-blind RCT	Home-based upper/lower limb global exercise telerehabilitation	Gait/mobility	Sensitivity/narrative evidence	RoB 2: High	Delivery-model/content comparison or both arms received telerehabilitation
Vloothuis et al. 2019 [116]	Observer-blinded multicenter RCT	Usual care	Mobility self-report	Single-study compatible/sensitivity evidence	RoB 2: Some concerns	Single compatible study only or not clinically/statistically compatible with selected pooled comparison
Wang et al. 2024 [117]	Controlled comparative trial;	Routine post-hospital follow-up	Global motor function; Balance;	Sensitivity/narrative evidence	RoB 2: High	Non-randomized/observational or unclear allocation;

Study	Design	Comparator/main contrast	Main quantitative contribution	Synthesis role	RoB tool and overall judgment	Main reason/interpretive note
	random allocation procedure incompletely robust	guidance + home rehabilitation prescription	ADL/motor-functional; multiple additional domains			narrative/sensitivity only
Wilson et al. 2021 [118]	Parallel RCT	GRASP active control home program	Upper limb motor function; Cognition/supportive	Sensitivity/narrative evidence	RoB 2: Some concerns	Not clinically/statistically compatible with primary pooled comparisons
Wolf et al. 2015 [119]	Prospective multisite single-blind RCT	Dose-matched HEP only	Upper limb motor impairment; Upper limb motor function	Sensitivity/narrative evidence	RoB 2: Some concerns	Missing or non-reconstructable effect variance
Wu et al. 2020 [120]	Single-blind RCT	Regular telephone follow-up/routine rehabilitation guidance	Global motor function; Balance; Gait/mobility; multiple additional domains	Primary pooled analysis	RoB 2: Some concerns	Included in quantitative Table 4; interpretation follows primary/exploratory label.
Yokota et al. 2025 [121]	Retrospective observational study with propensity score matching	No home-based telerehabilitation	Gait/exercise capacity	Sensitivity/narrative evidence	ROBINS-I: Serious	Non-randomized/observational or unclear allocation; narrative/sensitivity only
Zheng et al. 2026 [122]	Phase-Ib assessor-blinded RCT	Active stretching control	Gait/mobility; Lower limb function	Sensitivity/narrative evidence	RoB 2: High	Feasibility/pilot study not used for confirmatory primary pooling

Note: Table S14 is simplified to one row per study. The role column summarizes the highest-level quantitative use of each study; effect-level details are reported in Tables S15-S17 when used in a model.

Table S15. Primary and exploratory meta-analysis input values.

Analysis ID	Study	Comparator type	Motor domain	Outcome/time point	TR n	Comparator n	TR value	Comparator value	Effect size	SE; variance	Direction
A1	Eldemir et al. 2024 [63]	Telerehabilitation vs usual care/no additional therapy/waitlist	Gait/mobility	6MWT post (6 weeks)	15	15	517.93 (SD 49.69)	483.76 (SD 79.11)	0.50	SE=0.371; var=0.138	No reversal; higher values favor better outcome
A1	Swarnakar et al. 2023 [110]	Telerehabilitation vs usual care/no additional therapy/waitlist	Gait/mobility	SCIM III mobility post (8 weeks)	15	15	6.1 (SD 1.9)	4.3 (SD 1.4)	1.05	SE=0.389; var=0.152	No reversal; higher values favor better outcome
A1	Wu et al. 2020 [120]	Telerehabilitation vs usual care/no additional therapy/waitlist	Gait/mobility	6MWT post (12 weeks)	30	31	141.63 (SD 8.68)	129.45 (SD 7.06)	1.52	SE=0.291; var=0.085	No reversal; higher values favor better outcome
A2	Chumbler et al. 2012 [51]	Telerehabilitation vs usual care/no additional therapy/waitlist	Global motor-functional/ADL	Motor FONEFIM post (6 months)	24	19	83.7 (SD 9.9)	80.9 (SD 12.0)	0.25	SE=0.308; var=0.095	No reversal; higher values favor better outcome
A2	Sun et al. 2025 [109]	Telerehabilitation vs usual care/no additional therapy/waitlist	Global motor-functional/ADL	Barthel Index post (12 weeks)	100	100	88.7 (SD 9.6)	74.9 (SD 13.2)	1.19	SE=0.153; var=0.024	No reversal; higher values favor better outcome
A2	Wu et al. 2020 [120]	Telerehabilitation vs usual care/no additional therapy/waitlist	Global motor-functional/ADL	MBI post (12 weeks)	30	31	65.07 (SD 4.15)	60.81 (SD 5.24)	0.89	SE=0.268; var=0.072	No reversal; higher values favor better outcome
A3	Ballester et al. 2017 [42]	Telerehabilitation vs conventional/face-to-face or active rehabilitation	Upper/global motor function	CAHAI change (Week 3)	17	18	1.53 (SD 2.4)	-0.67 (SD 6.01)	0.46	SE=0.343; var=0.117	No reversal; higher values favor better outcome
A3	Chen et al. 2020 [49]	Telerehabilitation vs conventional/face-to-face or active rehabilitation	Upper/global motor function	FMA change (12 weeks)	26	26	11.115 (SD 8.905)	5.307 (SD 4.593)	0.81	SE=0.288; var=0.083	No reversal; higher values favor better outcome
A4	Pagliari et al. 2024 [91]	Telerehabilitation vs usual/active home rehabilitation	Balance/postural control	Mini-BESTest global post (6 weeks)	30	30	21.22 (SD 6.134492644057861)	18.58 (SD 6.134492644057861)	0.42	SE=0.261; var=0.068	No reversal; higher values favor better outcome
A4	Wu et al. 2020 [120]	Telerehabilitation vs usual/active home rehabilitation	Balance/postural control	BBS post (12 weeks)	30	31	43.13 (SD 2.32)	38.29 (SD 2.7)	1.90	SE=0.308; var=0.095	No reversal; higher values favor better outcome
A5	Adams et al. 2023 [38]	Telerehabilitation vs usual care/sham or handout control	Upper limb motor impairment	FMUE change (8 weeks)	9	9	10.1 (SD 5.4)	1.6 (SD 4.2)	8.60	SE=2.474; var=6.123	No reversal; higher values favor better outcome
A5	Toh et al. 2025a [112]	Telerehabilitation vs usual care/sham or handout control	Upper limb motor impairment	FMA-UE change (4 weeks)	20	20	2.95 (SD 3.55)	0.9 (SD 2.29)	2.05	SE=0.975; var=0.951	No reversal; higher values favor better outcome

Note: Values are rounded for publication. Calculations were performed using the unrounded extracted values.

Table S16. Model specification, pooled results, and sensitivity outputs.

Analysis ID	Analysis type	Studies, k	Participants, n	Effect measure	Primary model	Primary estimate (95% CI)	I²; τ²	Sensitivity model/result	Robustness conclusion
A1	Primary pooled analysis	3	121	SMD, Hedges' g	REML random-effects + Hartung-Knapp	1.06 (-0.23 to 2.35)	I²=57.6%; τ²=0.161	DL: 1.06 (0.45 to 1.66)	No clear between-group difference. Interpret cautiously because of heterogeneity.
A2	Primary pooled analysis	3	304	SMD, Hedges' g	REML random-effects + Hartung-Knapp	0.83 (-0.35 to 2.00)	I²=73.6%; τ²=0.161	DL: 0.83 (0.30 to 1.35)	No clear between-group difference. Interpret cautiously because of heterogeneity.
A3	Exploratory k=2 quantitative summary	2	87	SMD, Hedges' g	REML random-effects, exploratory	0.67 (0.23 to 1.10)	I²=0.0%; τ²=0.000	DL: 0.67 (0.23 to 1.10)	Effect favored telerehabilitation. Exploratory only.
A4	Exploratory k=2 quantitative summary	2	121	SMD, Hedges' g	REML random-effects, exploratory	1.15 (-0.29 to 2.59)	I²=92.5%; τ²=1.000	DL: 1.15 (-0.29 to 2.59)	No clear between-group difference. Exploratory only. Interpret cautiously because of heterogeneity.
A5	Exploratory k=2 quantitative summary	2	58	MD	REML random-effects, exploratory	4.93 (-1.44 to 11.30)	I²=83.5%; τ²=17.914	DL: 4.93 (-1.44 to 11.30)	No clear between-group difference. Exploratory only. Interpret cautiously because of heterogeneity.

Note: DerSimonian-Laird estimates were used only as sensitivity analyses. Prediction intervals and small-study effect tests were not calculated because k < 10 for all analyses.

Table S17: Final GRADE evidence profile for quantitative and key non-pooled outcomes.

Comparison/outcome	Studies and participants	Effect estimate or data basis	Risk of bias	Inconsistency	Indirectness	Imprecision	Publication bias	Overall certainty and rationale
Telerehabilitation vs usual care/no additional therapy/waitlist; Gait/mobility; Post-intervention	3 studies; n = 121. Eldemir et al. 2024 [63]; Swarnakar et al. 2023 [110]; Wu et al. 2020 [120]	1.06 (-0.23 to 2.35)	Serious: most contributing trials had some concerns/high RoB or pilot/context-specific limitations.	Serious: mixed neurological diagnoses and heterogeneous scales; I^2 considered in certainty.	Serious: broad gait/mobility construct across MS, SCI and stroke.	Serious: small k and wide confidence interval.	Not assessable because k < 10.	Very low: Downgraded for risk of bias, inconsistency/indirectness across neurological diagnoses and scales, and imprecision; publication bias not assessable (k < 10).
Telerehabilitation vs usual care/no additional therapy/waitlist; Global motor-functional/ADL; Post-intervention to 6 months	3 studies; n = 304. Chumbler et al. 2012 [51]; Sun et al. 2025 [109]; Wu et al. 2020 [120]	0.83 (-0.35 to 2.00)	Serious: unblinded delivery and self-reported or broad functional outcomes in contributing RCTs.	Serious: broad functional constructs and time-point heterogeneity.	Serious: ADL/global motor-functional scales measure related but non-identical constructs.	Serious: small k and imprecise pooled estimate.	Not assessable because k < 10.	Very low: Broad functional constructs and time points; downgraded for risk of bias, inconsistency/indirectness and imprecision; publication bias not assessable.
Telerehabilitation vs conventional/face-to-face or active rehabilitation; Upper/global motor function; Change score at post-intervention	2 studies; n = 87. Ballester et al. 2017 [42]; Chen et al. 2020 [49]	0.67 (0.23 to 1.10)	Serious: small RCTs, active comparators, and some concerns in RoB 2.	Serious: only two studies with different outcome constructs.	Serious: home VR/OT and conventional rehabilitation comparisons are clinically heterogeneous.	Serious: exploratory k = 2 summary only.	Not assessable because k < 10.	Low: Exploratory k = 2 evidence; downgraded for risk of bias, indirectness across outcome constructs and imprecision; publication bias not assessable.
Telerehabilitation vs usual/active home rehabilitation; Balance/postural control; Post-intervention	2 studies; n = 121. Pagliari et al. 2024 [91]; Wu et al. 2020 [120]	1.15 (-0.29 to 2.59)	Serious: EMM-derived data and some concerns in outcome assessment/delivery blinding.	Serious: k = 2 with heterogeneous balance scales and diagnoses.	Serious: active home rehabilitation/usual care comparators differ.	Serious: exploratory k = 2 and wide uncertainty.	Not assessable because k < 10.	Very low: Exploratory k = 2 evidence with different diagnoses, balance scales and one EMM/SE-derived SD; publication bias not assessable.
Telerehabilitation vs usual care/sham or handout control; Upper limb motor impairment; Change score at post-intervention	2 studies; n = 58. Adams et al. 2023 [38]; Toh et al. 2025a [112]	4.93 (-1.44 to 11.30)	Serious: small unblinded trials with some concerns in RoB 2.	Not serious to serious: both studies use FMA-UE change but interventions/comparators differ.	Some concern: comparator varies from usual care to sham/handout control.	Serious: exploratory k = 2 and limited sample size.	Not assessable because k < 10.	Low: Exploratory k = 2 stroke upper-limb FMA-UE change evidence; downgraded for risk of bias and imprecision; publication bias not assessable.
Home-based telerehabilitation vs dose-matched in-clinic rehabilitation; stroke upper-limb motor function	1 RCT; n = 124. Cramer et al. 2019 [56].	Adjusted FM change difference 0.06 points (95% CI -2.14 to 2.26); noninferiority margin met.	Not serious: robust randomization, assessor blinding, matched dose/intensity and ITT sensitivity analyses.	Not assessable; single study.	Not serious for delivery-model/noninferiority question.	Not serious to serious; single study but powered for noninferiority.	Not assessable because k < 10.	Moderate: strong single noninferiority RCT; downgraded conservatively for single-study evidence.
Home remotely supervised aerobic exercise vs active stretching; Parkinson disease motor symptoms	1 RCT; n = 125. van der Kolk et al. 2019 [60].	Adjusted MDS-UPDRS III difference favored remote aerobic exercise by 4.20 points (95% CI 1.60 to 6.90).	Not serious: randomization, masking and blinded outcome assessment were strong.	Not assessable; single study.	Not serious for mild Parkinson disease remote aerobic exercise.	Not serious to serious; estimate excludes null but is from one trial.	Not assessable because k < 10.	Moderate: strong RCT evidence, downgraded conservatively for single-study evidence.
Virtual upper-limb activity programme vs waitlist/usual care; real-world upper-limb activity	1 RCT; n = 66. Simpson et al. 2025 [105].	Adjusted MD 368.10 reach-to-grasp counts/day (95% CI 6.20 to 730.10).	Serious/some concerns: unblinded waitlist design despite objective wearable outcome.	Not assessable; single study.	Not serious for real-world upper-limb activity after stroke.	Serious; CI is close to null and relatively wide.	Not assessable because k < 10.	Low: downgraded for risk of bias and imprecision.

Note: GRADE certainty was assessed at the comparison-outcome level. Randomized evidence starts as high certainty and may be downgraded for risk of bias, inconsistency, indirectness, imprecision and publication bias. Non-randomized evidence starts as low certainty and was not combined with randomized evidence for primary certainty judgments. Publication bias was considered not assessable when k < 10.

Abbreviations: *2MWD, 2-minute walking distance; 2MWT, 2-minute walk test; 4MWT, 4-meter walk test; 5STS, Five Times Sit-to-Stand Test; 6MPT, 6-minute push test; 6MWD, 6-minute walking distance; 6MWT, 6-minute walk test; 9-HPT/9HPT/N-HPT/NHPT, Nine-Hole Peg Test; 10-MWT/10MWT, 10-meter walk test; 30STS, 30-second sit-to-stand test; A-VO2, arteriovenous oxygen difference; ABC, Activities-Specific Balance Confidence Scale; ABC-SF, Activities-Specific Balance Confidence Scale-Short Form; ABF, auditory biofeedback; AC, algorithm-controlled; AC/EM, algorithm-controlled/enhanced motivation; ACRM, American Congress of Rehabilitation Medicine; ACTIV, Augmented Community Telerehabilitation Intervention; ADL, activities of daily living; AEMF, Automated Evaluation of Motor Function; AIS, acute ischemic stroke; American Spinal Injury Association Impairment Scale when used for spinal cord injury classification; AMSQ, Arm Function in Multiple Sclerosis Questionnaire; ANCOVA, analysis of covariance; APB, abductor pollicis brevis; APDDS, Adapted Patient Determined Disease Steps; ARAT, Action Research Arm Test; AROM, active range of motion; ATTG/STTG, asynchronous/synchronized telerehabilitation treatment group; BBA, Brunel Balance Assessment; BBS, Berg Balance Scale; BBT, Box and Blocks Test; BDI, Beck Depression Inventory; BDI-II, Beck Depression Inventory-II; BDNF, brain-derived neurotrophic factor; BI, Barthel Index; BICAMS, Brief International Cognitive Assessment for Multiple Sclerosis; BIG, Lee Silverman Voice Treatment BIG; BMI, body mass index; BPI, Brief Pain Inventory; BS-OT, bedside occupational therapist; CAHAI, Chedoke Arm and Hand Activity Inventory; CARE4STROKE, caregiver-mediated exercises with e-health support after stroke; CAS, Coronavirus Anxiety Scale; CB&M, Community Balance and Mobility; CBI, Caregiver Burden Inventory; CDP, computerized dynamic posturography; CDSR, Cochrane Database of Systematic Reviews; CENTRAL, Cochrane Central Register of Controlled Trials; CG, control group; CI, confidence interval; CIMT, Constraint-Induced Movement Therapy; CNN, convolutional neural network; CONUT, Controlling Nutritional Status; COP, center of pressure; COR, conventional outpatient rehabilitation; COVID-19, coronavirus disease 2019; CR, conventional rehabilitation; CSE, core-stability exercise; CSI, Caregiver Strain Index; CST, corticospinal tract; DASH, Disabilities of the Arm, Shoulder and Hand; DGI, Dynamic Gait Index; DL, DerSimonian-Laird; EARS, Exercise Adherence Rating Scale; ECRL, extensor carpi radialis longus; EDNA, Elements virtual rehabilitation system; EDSS, Expanded Disability Status Scale; EMM, estimated marginal mean; EQ-5D/EQ-5D-5L, EuroQol 5 Dimension/5-Level; ES, effect size; ET, exercise therapy; ETNS, electromyography-triggered neuromuscular stimulation; FAB, Frontal Assessment Battery; FAC, Functional Ambulation Category; FES, functional electrical stimulation; FES-I, Falls Efficacy Scale-International; FGA, Functional Gait Assessment; FIM, Functional Independence Measure; FIS/FSS, Fatigue Impact Scale/Fatigue Severity Scale; FM/FMA, Fugl-Meyer Assessment; FMA-UE/FMUE/UE-FM/UEFMA, Fugl-Meyer Assessment for the upper extremity; fMRI, functional magnetic resonance imaging; FOG, freezing of gait; FOGQ, Freezing of Gait Questionnaire; FONEFIM, Telephone Version of the Functional Independence Measure; FRoM, functional range of motion; FTF, face to face; FTSST, Five Times Sit-to-Stand Test; GABS-B, Gait and Balance Scale Part B; GLTEQ, Godin Leisure-Time Exercise Questionnaire; GRADE, Grading of Recommendations Assessment, Development and Evaluation; GRASP, Graded Repetitive Arm Supplementary Program or Glove Rehabilitation Application for Stroke Patients according to study context; HADS, Hospital Anxiety and Depression*

Scale; HAMD, Hamilton Depression Rating Scale; HAQUAMS, Hamburg Quality of Life Questionnaire in Multiple Sclerosis; HAT, home automated tele-management; HBR, home-based rehabilitation; HCAD, Home Care Activity Desk; HEP, home exercise programme; HHD, hand-held dynamometry; HK, Hartung-Knapp; HMP, Hand Mentor Pro; HoVRS, Home Virtual Rehabilitation System; HPT, home physical therapy; HR, heart rate; HRQOL, health-related quality of life; HTR, home-based telesupervising rehabilitation; I^2*, inconsistency statistic; IADL, instrumental activities of daily living; IC, in-clinic therapy; IHMD, in-home messaging device; IKEMS, isometric knee extension muscle strength; IMI, Intrinsic Motivation Inventory; IPA, Impact on Participation and Autonomy Questionnaire; IoT, Internet of Things; IQR, interquartile range; ISNCSCI, International Standards for Neurological Classification of Spinal Cord Injury; IT-AAT, Italian version of the Aachen Aphasia Test; ITT, intention-to-treat; JAMAR, JAMAR Hand Dynamometer; JHFT, Jebsen Hand Function Test; k, number of studies; K-ICARS, kinetic-function subparameter of the International Cooperative Ataxia Rating Scale; K-MBI, Korean Modified Barthel Index; LDJ, log dimensionless jerk; LEMS, lower-extremity motor score; LLFDI, Late-Life Function and Disability Instrument; LMIC, low- and middle-income country; LOCF, last observation carried forward; LOS, length of stay; LS mean, least-squares mean; LSVT BIG, Lee Silverman Voice Treatment BIG; LTCF, long-term care facility; MAL, Motor Activity Log; MAL-14, abridged 14-item Motor Activity Log; MAL-30, 30-item Motor Activity Log; MAL-AOU, Motor Activity Log-Amount of Use; MAL-QOM, Motor Activity Log-Quality of Movement; MAS, Modified Ashworth Scale; MBI, Modified Barthel Index; MCID, minimal clinically important difference; MD, mean difference; MDS-UPDRS, Movement Disorders Society-Unified Parkinson's Disease Rating Scale; MDT, multidisciplinary team; MFAC, Modified Functional Ambulatory Category; MFES, Modified Falls Efficacy Scale; MFIS, Modified Fatigue Impact Scale; Mii-vitaliSe, multiple sclerosis Nintendo Wii intervention package; Mini-BESTest, mini-Balance Evaluation Systems Test; ML, machine learning; MMDT, Minnesota Manual Dexterity Test; MMSE, Mini-Mental State Examination; MoCA, Montreal Cognitive Assessment; MRI, magnetic resonance imaging; m-MAS, modified-Modified Ashworth Scale; mRS, modified Rankin Scale; MS, multiple sclerosis; MS HAT, multiple sclerosis home automated tele-management; MSIS, Multiple Sclerosis Impact Scale; MSIS-29, 29-item Multiple Sclerosis Impact Scale; MSQOL-54, Multiple Sclerosis Quality of Life-54; MSSE, Multiple Sclerosis Self-Efficacy Questionnaire; MSWS-12, 12-item Multiple Sclerosis Walking Scale; MU/SU, multiuser/single-user; MVPA, moderate-to-vigorous physical activity; Muvity, telerehabilitation software platform; NBS, Navigated Brain Stimulation; NEADL, Nottingham Extended Activities of Daily Living; NFI, Neurobehavioural Functioning Inventory; NFOGQ, New Freezing of Gait Questionnaire; NGF, nerve growth factor; NIHSS, National Institutes of Health Stroke Scale; NIVRT, nonimmersive virtual reality training; NT-3, neurotrophin-3; OCS, Oxford Cognitive Screen; OT, occupational therapy; PA, physical activity; PAHCO, physical activity-related health competence; PAS, Parkinson's Activity Scale; PASS, Postural Assessment Scale for Stroke; PBMSI, Preference-Based Multiple Sclerosis Index; PCS, Physical Component Summary; PD, Parkinson disease; PDDS, Patient Determined Disease Steps; PDQ-8/PDQ-39, Parkinson's Disease Questionnaire-8/39; PGWBI, Psychological General Well-Being Index; PHQ-9, Patient Health Questionnaire-9; PICO, Population, Intervention, Comparator, Outcomes; Pilates-TR, Pilates-based telerehabilitation; PMS, Parkinson Mobility Scale; PNF, proprioceptive neuromuscular facilitation; POMA-B/POMA-G, Performance-Oriented Mobility Assessment balance/gait subscale; PRISMA, Preferred Reporting Items for Systematic Reviews and Meta-Analyses; PRO, patient-reported outcome; PROSPERO, International Prospective Register of Systematic Reviews; PSM, propensity score matching; PT, physical therapy; PwMS, people with multiple sclerosis; PwPD, people with Parkinson disease; QoL, quality of life; RAHFT, ReJoyce Automated Hand Function Test; RCT, randomized controlled trial; ReJoyce, Rehabilitation Joystick for Computerized Exercise; REML, restricted maximum likelihood; RGS, Rehabilitation Gaming System; RMI, Rivermead Mobility Index; RoB, risk of bias; ROBINS-I, Risk Of Bias In Non-randomized Studies of Interventions; ROM, range of motion; RMS, root mean square; RPE, rating of perceived exertion; RSC, Rocco Salvatore Calabrò; SABI, severe acquired brain injury; SARA, Scale for the Assessment and Rating of Ataxia; SCI, spinal cord injury; SCIM, Spinal Cord Independence Measure; SCT, social cognitive theory; SD, standard deviation; SDMT, Symbol Digit Modalities Test; SE, standard error; SEE, Self-Efficacy for Exercise; SF-36, 36-Item Short-Form Health Survey; S-FIST, Spanish Function in Sitting Test; SIBT, sensory integration balance training; SIS, Stroke Impact Scale; SMD, standardized mean difference; SOT, Sensory Organization Test; SPARC, Spectral Arc Length; SPPB, Short Physical Performance Battery; SR, Smart Reminder; SSEQ, Stroke Self-Efficacy Questionnaire; SS-QOL, Stroke-Specific Quality of Life; STeleR, stroke telerehabilitation intervention; STREAM, Stroke Rehabilitation Assessment of Movement Scale; STRIVE, STRoke Interactive Virtual thErapy; SUS, System Usability Scale; SWA, SenseWear Armband; SWST, Stand-Walk-Sit Test; S-TIS, Spanish Trunk Impairment Scale; T25FW, Timed 25-Foot Walk; TA, tibialis anterior; TAI, Transfer Assessment Instrument; TBI, traumatic brain injury; tDCS, transcranial direct current stimulation; teleSCI, telerehabilitation in spinal cord injury; TENZR, wearable reach-to-grasp activity monitor; TIA, transient ischemic attack; TIDieR, Template for Intervention Description and Replication; TIS, Trunk Impairment Scale; TOCT, task-oriented circuit training; TOCT-TR, task-oriented circuit training-based telerehabilitation; TOT, task-oriented training; TR, telerehabilitation; TS, Tinetti Scale; TSRQ-15, Treatment Self-Regulation Questionnaire-15; TUG, Timed Up and Go; UC, usual care; UE, upper extremity; UEMS/LEMS, upper/lower extremity motor scores; UL, upper limb; UPDRS, Unified Parkinson's Disease Rating Scale; UTRT, usual territorial rehabilitative treatment; V-ABC, Virtual Arm Boot Camp; VAS, Visual Analog Scale; VERGE, Virtual Environment for Rehabilitative Gaming Exercises; V-TOCT, virtual reality-supported task-oriented circuit therapy; VR, virtual reality; VRBT, Virtual Reality Balance Trainer; VRRS, Virtual Reality Rehabilitation System; WHODAS 2.0, World Health Organization Disability Assessment Schedule 2.0; WHOQOL-BREF, World Health Organization Quality of Life-BREF; WISCI, Walking Index for Spinal Cord Injury; WMFT, Wolf Motor Function Test; WSN, wireless sensor network;* τ^2*, between-study variance.*
